# Nickel-Catalyzed Kumada Cross-Coupling Reactions of Benzylic Sulfonamides

**DOI:** 10.3390/molecules26195947

**Published:** 2021-09-30

**Authors:** Kirsten A. Hewitt, Claire A. Herbert, Alissa C. Matus, Elizabeth R. Jarvo

**Affiliations:** Department of Chemistry, University of California, Irvine, CA 92697-2025, USA; khewitt1@uci.edu (K.A.H.); caherber@uci.edu (C.A.H.); matusa@uci.edu (A.C.M.)

**Keywords:** cross-coupling reactions, sulfonamides, nickel, catalysis, hydrocarbons

## Abstract

Herein, we report a Kumada cross-coupling reaction of benzylic sulfonamides. The scope of the transformation includes acyclic and cyclic sulfonamide precursors that cleanly produce highly substituted acyclic fragments. Preliminary data are consistent with a stereospecific mechanism that allows for a diastereoselective reaction.

## 1. Introduction

Transition-metal catalyzed cross-coupling (XC) reactions have transformed modern synthetic organic chemistry by creating an arsenal of carbon–carbon bond forming reactions [1,2,3,4,5,6]. Nickel is a cost-effective metal that is capable of activating challenging electrophiles such as amine derivatives [7,8,9,10]. Intense research efforts have been employed in the development of nickel-catalyzed XC reactions of sluggish electrophiles [11,12,13]. However, the XC reaction of alkyl amine derivatives has remained a significant challenge [14,15,16,17,18]. Historically, in order to facilitate nickel-catalyzed reactions, activation of these carbon–nitrogen bonds has been achieved via incorporation into strained aziridine rings or transformation to ammonium salts [19].

**Scheme 1:**
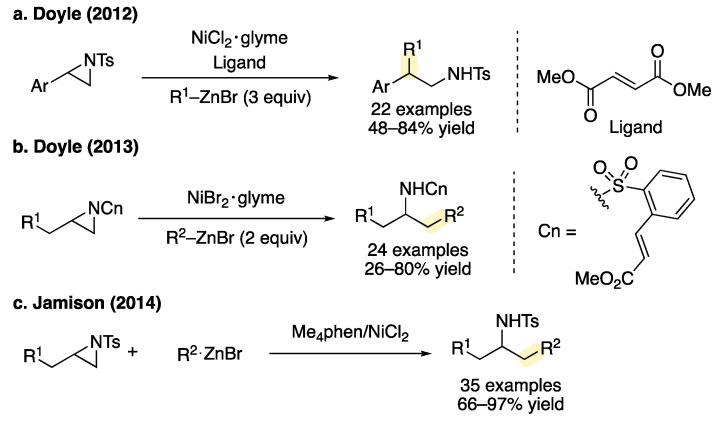
Cross-Coupling (XC) Reactions of Aziridines.

Ring-strain-promoted XC of aziridines has been accomplished [20]. Early stoichiometric work by Hillhouse established that aziridines undergo facile oxidative addition with nickel complexes [21]. Catalytic Negishi reactions of sulfonylaziridines have subsequently been established. The Doyle laboratory reported a regioselective Negishi XC reaction of styrenyl aziridines with alkylzinc reagents with substitution at the benzylic position (Scheme 1a) [22,23]. Key to their success was the use of an electron deficient fumarate ligand. Shortly thereafter, the Doyle and Jamison groups independently described a regioselective Negishi XC reaction of alkyl aziridines with alkylzinc reagents to forge the desired carbon–carbon bond (Scheme 1b,c) [24,25]. The differing regioselectivity of these reactions can be explained by comparing the oxidative addition events of the C–N bonds. Styrenyl aziridines preferentially undergo oxidative addition at the benzylic center to afford a η^3^-benzylnickel complex. In contrast, alkyl aziridines, which do not contain an aromatic ring to direct the nickel complex, preferentially undergo oxidative addition at the less hindered position [21]. These reports demonstrate the ability to activate the C–N bond in strained rings.

**Scheme 2:**
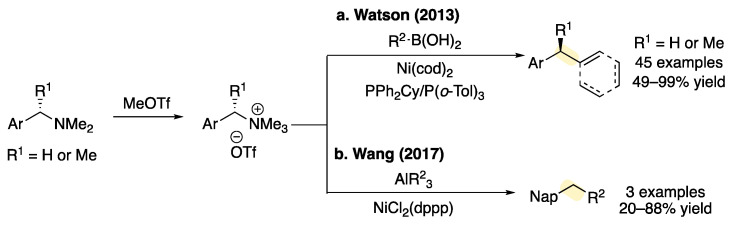
XC Reactions of Trimethylammonium salts. Nap = Naphthyl.

Development of XC reactions of acyclic benzylamine derivatives has relied upon formation of highly reactive electrophiles (i.e., charged ammonium salts) [26,27]. For example, the Watson laboratory demonstrated that benzylic trimethylammonium salts are competent electrophiles in Suzuki-Miyaura XC reactions with aryl and vinylboronic acids (Scheme 2a) [28,29]. Similarly, the Wang laboratory disclosed the XC reaction of benzylic trimethylammonium salts with organoaluminum reagents to forge the desired carbon–carbon bond (Scheme 2b) [30].

**Scheme 3:**
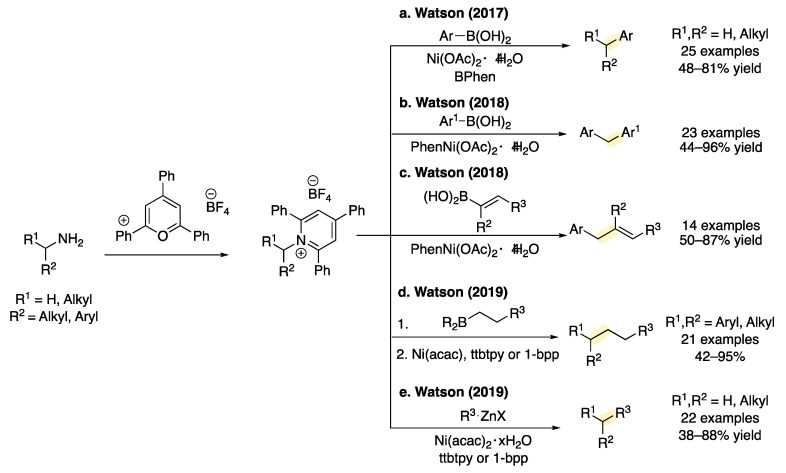
XC Reactions of Katritzky Salts.

The use of Katritzky salts to activate amines has proven to be sufficient for activation of benzylic and alkyl amines for Suzuki-Miyaura and Negishi XC reactions. Previously, it has been observed that Katritzky salts participate in S_N_2, radical, and Minisci-type reactions, and in recent years, many transition-metal catalyzed reactions have been developed [31,32,33,34,35,36,37,38,39,40]. The Watson laboratory hypothesized that these air and moisture stable salts would be suitable electrophiles in a XC reaction [41]. To test their hypothesis, primary amines were converted to Katritzky salts via a condensation reaction with 2,4,6-triphenylpyrylium tetrafluoroborate and the corresponding salts were subjected to Suzuki- Miyaura XC reactions with aryl boronic acids. The desired cross-coupled products were obtained in good yields (Scheme 3a) [42]. This strategy was amenable to the coupling of primary benzylic Katritzky salts as well (Scheme 3b) [43]. Additionally, vinyl boranes and alkylborane reagents, generated in situ by hydroboration of alkenes, participated in XC with Katritzky salts (Scheme 3c,d) [44,45]. This strategy has been extended beyond Suzuki-Miyaura reactions to include Negishi XC reactions with alkylzinc reagents (Scheme 3e) [46].

**Scheme 4:**
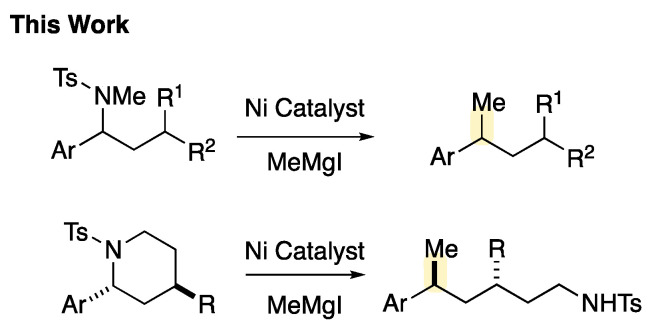
Kumada XC Reactions of Benzylic Sulfonamides.

These methods establish strain- and charge-based strategies to activate amines for use as the electrophilic partner in XC reactions; however, the requirement for aziridines or functionalization as highly reactive ammonium salts remains a major limitation in broad application of these methods. In this manuscript, we report the first nickel-catalyzed Kumada XC reaction of simple benzylic sulfonamides with methylmagnesium iodide (Scheme 4). Previously, the Jarvo laboratory disclosed the Kumada XC reaction of benzylic ethers which proceeded in excellent yields, and enantio- and diastereoselectivity [12,47,48]. Building on this work, we aimed to develop an analogous reaction that employed benzylic sulfonamides. Ethers and sulfonamides have similar leaving group abilities, as the conjugate bases have similar pK_a_’s, and we hypothesized sulfonamides would behave similarly to ethers in a XC reaction [49,50]. In addition, these moieties are appealing because they are common functional groups in synthesis. Furthermore, we demonstrate that sulfonamides undergo stereospecific XC reactions, in contrast to the stereoablative reactivity typically observed with styrenyl aziridines and Katritzky salts [22,36,37,38,39,40,51,52,53,54]. This stereospecific manifold allows for rapid diastereoselective construction of acyclic fragments bearing 1,3-substitution [55,56].

## 2. Results and Discussion

We began our investigation into the Kumada XC reaction with benzylic sulfonamide **1**, which was synthesized in three steps from the commercially available aldehyde (See Experimental Section for substrate synthesis). Previously, Kumada XC reactions of benzylic ethers employed Ni(cod)_2_ and racemic BINAP as the optimal reaction conditions [12,47,48]. Under these conditions, we were excited to observe 25% yield of the desired cross-coupled product **2** (Table 1, entry 1). Increasing the catalyst loading to 15 mol % improved the yield of the reaction (entry 2). However, it also increased the yield of the undesired styrene product **3** arising from β-hydride elimination. In an effort to improve the ratio between desired product **2** and styrene product **3**, we investigated a series of bidentate phosphine, NHC, and pyridine ligands. DPEPhos improved the yield of **2** and decreased the amount of styrene **3** (entry 3). However, all other ligands evaluated did not improve the yield of **2** (entries 4–7).

We next investigated an alternative precatalyst. Previously, the Jarvo laboratory reported the cross-electrophile coupling (XEC) reaction of benzylic and allylic sulfonamides which employed a BINAP-ligated nickel (II) precatalyst [50,57,58]. Utilizing these conditions, with 15 mol % of catalyst, we were delighted to observe the desired product in 54% yield and 40% yield of styrene **3** (entry 8). We elected to proceed with the nickel (II) precatalyst as it provided the desired product in the highest yield.

**Scheme 5:**
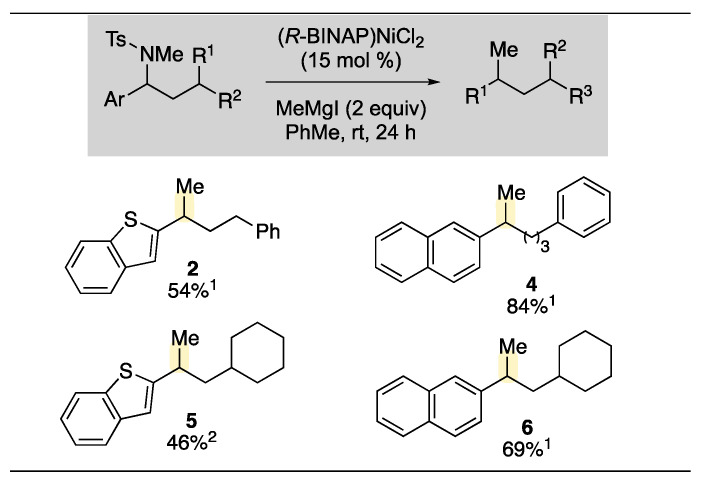
Scope of the Kumada XC Reaction of Acyclic Sulfonamides. ^1^ Yield determined by ^1^H NMR based on comparison to PhTMS as internal standard. ^2^ Isolated yield.

With optimized conditions in hand, we evaluated the scope of the Kumada XC reaction (Scheme 5). Naphthyl substrates were well tolerated under the standard reaction conditions and product **4** was observed in 84% yield. Notably, products such as **5** and **6** with branching at the β-position provided good yields of cross-coupled products with lesser amounts of styrenes formed from β-hydride elimination (20–30%) when compared to product **2**. We hypothesized that this increase in steric bulk destabilized the conformation necessary for β-hydride elimination to proceed.

**Scheme 6:**
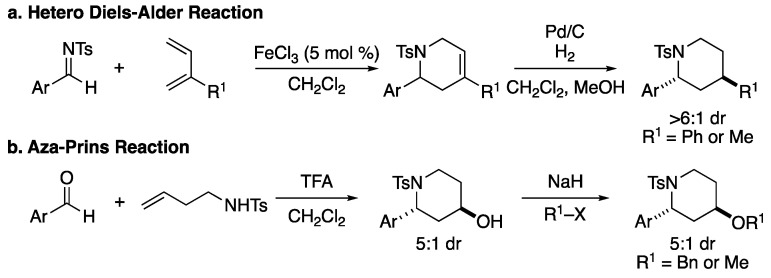
Arylpiperidine Synthesis via (**a**) Hetero Diels-Alder (HDA) Reaction and (**b**) Aza-Prins Reaction.

We also sought to evaluate a series of arylpiperidines, with the expectation that a stereospecific XC reaction at the benzylic position would provide synthetic access to highly substituted acyclic fragments. Furthermore, products would bear a pendant sulfonamide moiety, available for subsequent functionalization [50]. Rapid synthesis of the requisite cyclic sulfonamides was achieved by hetero Diels-Alder (HDA) cycloadditions or aza-Prins reactions [59,60,61]. For substrates with alkyl substituents in the 4-position, [4+2] HDA reactions provided the requisite starting materials (Scheme 6a). For substrates bearing ether groups in the 4-position, an aza-Prins reaction provided the requisite 2-aryl-4-hydroxylpiperidine that could be subsequently methylated or benzylated. (Scheme 6b).

**Scheme 7:**
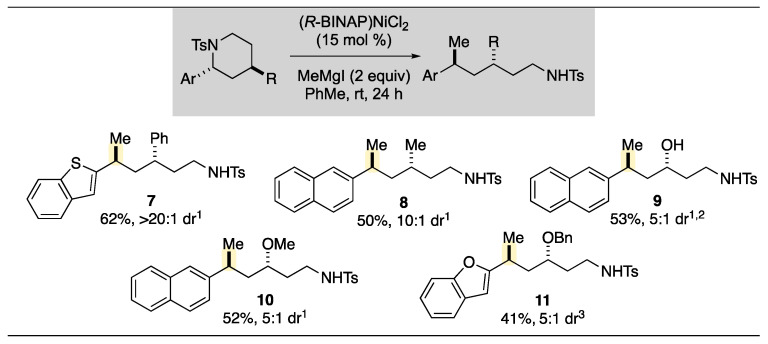
Scope of the Kumada XC Reaction of Cyclic Sulfonamides. ^1^ Isolated yield. ^2^ R = OBn in starting material and provided the free alcohol in product. ^3^ Yield determined by ^1^H NMR based on comparison to PhTMS as internal standard.

With rapid and diastereoselective access to the desired piperidines, we examined these cyclic substrates in ring-opening Kumada XC reactions (Scheme 7). Phenyl and methyl substituents (products **7** and **8**) were well tolerated and minimal amounts (<5%) of β-hydride elimination were observed. Methylated and benzylated ethers were well tolerated and provided the desired products in good yields (**9**, **10**, and **11**) [62]. It is important to note that the diastereomeric ratio observed in the products is consistent with the diastereomeric ratio of the starting material (See Materials and Methods Section). Therefore, preliminary data support a stereospecific Kumada XC reaction.

**Scheme 8:**
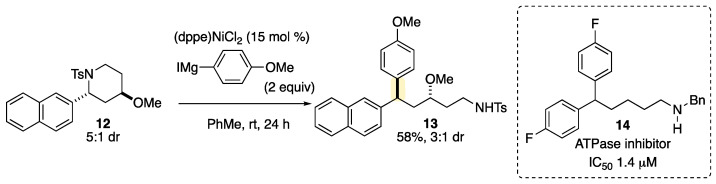
Kumada XC Reaction with Aryl Grignard Reagent.

To further develop the potential scope of this reaction, we sought to establish a ring-opening of a sulfonyl piperidine with an aryl Grignard reagent (Scheme 8). Such transformations would provide synthetic access to diarylalkanes bearing pendant sulfonamides, including rapid assembly of stereochemically-rich analogs of ATPase inhibitor **14** [63,64,65,66,67]. We have previously observed that in Kumada XC reactions of benzylic ethers employing aryl Grignard reagents, the optimal nickel catalyst is ligated by dppe [68]. We were pleased to see that this trend applied to benzylic sulfonamides: employing the commercially available precatalyst, (dppe)NiCl_2_, the XC reaction proceeded smoothly to provide the desired product **13** in 58% isolated yield [69].

**Scheme 9:**
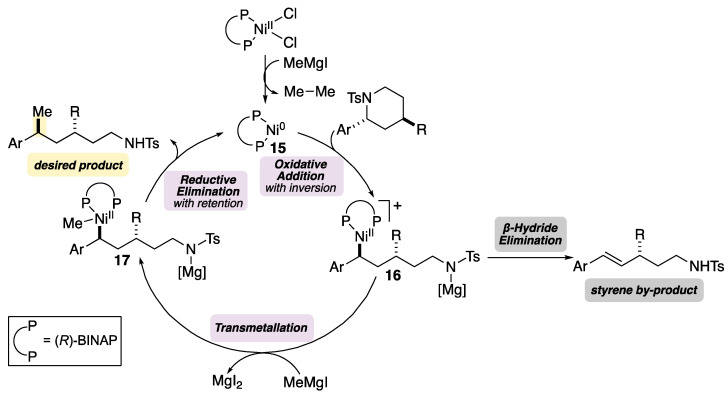
Proposed Mechanism of Kumada XC Reaction. Speciation of magnesium complexes are omitted for clarity.

We propose the following catalytic cycle for the Kumada XC reaction based on related mechanisms for the Kumada XC reaction of benzylic ethers and the XEC reaction of benzylic sulfonamides (Scheme 9) [50,70]. First, reduction of the nickel(II) precatalyst with the Grignard reagent provides the active Ni(0) catalyst **15**. Next, oxidative addition of the benzylic sulfonamide affords the Ni(II) intermediate **16**. Based on the calculated reaction coordinate diagram and transition state energies for related transformations, we hypothesize that rate-determining oxidative addition occurs with inversion of the benzylic carbon [12,47,48,70]. This step is facilitated by Lewis acidic magnesium salts that activate the sulfonamide moiety. Transmetallation with the Grignard reagent provides alkylnickel complex **17**. Subsequent reductive elimination, which occurs with retention at the benzylic center, affords the desired product and turns over the catalyst. Alternatively, intermediate **16** can undergo β-hydride elimination to afford the observed styrene by-product.

## 3. Materials and Methods

### 3.1. General Procedures

All reactions were carried out under an atmosphere of N_2_, or Ar when noted. All glassware was oven- or flame-dried prior to use. Tetrahydrofuran (THF), diethyl ether (Et_2_O), dichloromethane (CH_2_Cl_2_), and toluene (PhMe) were degassed with Ar and then passed through two 4 × 36 inch columns of anhydrous neutral A-2 alumina (8 × 14 mesh; LaRoche Chemicals; activated under a flow of argon at 350 °C for 12 h) to remove H_2_O [71]. All other solvents utilized were purchased anhydrous commercially, or purified as described. ^1^H NMR spectra were recorded on Bruker DRX-400 (400 MHz ^1^H, 100 MHz ^13^C, 376.5 MHz ^19^F), GN-500 (500 MHz ^1^H, 125.4 MHz ^13^C), or CRYO-500 (500 MHz ^1^H, 125.8 MHz ^13^C) spectrometers. Proton chemical shifts are reported in ppm (δ) relative to internal tetramethylsilane (TMS, δ 0.00). Data are reported as follows: chemical shift (multiplicity [singlet (s), broad singlet (br s), doublet (d), doublet of doublet (dd), doublet of doublet of doublets (ddd), doublet of doublet of doublet of doublets (dddd), doublet of triplet (dt), doublet of doublet of triplet (ddt), doublet of triplet of doublet (dtd), triplet (t), broad triplet (br t), triplet of doublet (td), triplet of doublet of doublet (tdd), triplet of triplet (tt), quartet (q), quartet of doublet (qd), quartet of doublet of doublets (qdd), quintet (quint), apparent quintet (appar quint), sextet, apparent sextet (appar sextet), multiplet (m)]. coupling constants [Hz], integration). Carbon chemical shifts are reported in ppm (δ) relative to TMS with the respective solvent resonance as the internal standard (CDCl_3_, δ 77.16 ppm). Unless otherwise indicated, NMR data were collected at 25 °C. Infrared (IR) spectra were obtained on a Thermo Scientific Nicolet iS5 spectrometer with an iD5 ATR tip (neat) and are reported in terms of frequency of absorption (cm^−1^). Analytical thin-layer chromatography (TLC) was performed using Silica Gel 60 F_254_ precoated plates (0.25 mm thickness). Visualization was accomplished by irradiation with a UV lamp and/or staining with KMnO_4_ or CAM. Flash chromatography was performed using SiliaFlash F60 (40–63 μm, 60 Å) from SiliCycle. Automated chromatography was carried out on a Teledyne Isco CombiFlash Rf Plus. Melting points (m.p.) were obtained using a Mel-Temp melting point apparatus and are uncorrected. High resolution mass spectrometry was performed by the University of California, Irvine Mass Spectrometry Center. See the ^1^H, ^13^C, COSY and NOE NMR detailed data in the Supplementary Materials.

Bis(1,5-cyclooctadiene)nickel was purchased from Strem, stored in a glove box freezer (–20 °C) under an atmosphere of N_2_ and used as received. All ligands were purchased from Strem or Sigma Aldrich and were stored in a glovebox and used as received. The methylmagnesium iodide was titrated with iodine prior to use [72]. All other chemicals were purchased commercially and used as received, unless otherwise noted.

### 3.2. Experimental

#### 3.2.1. General Kumada Cross-Coupling Reaction Procedures

##### Method A: Kumada Cross-Coupling Reaction

In a glovebox, a flame-dried 7 mL vial equipped with a stir bar was charged with sulfonamide substrate (1.0 equiv), nickel precatalyst (15 mol %) and PhMe (0.10–0.20 M in substrate). The Grignard reagent (2.0 equiv) was then added dropwise via a syringe. After 24 h, the reaction was removed from the glovebox, quenched with methanol, filtered through a plug of silica gel eluting with 100% Et_2_O and concentrated in vacuo. Phenyltrimethylsilane (PhTMS; 8.6 μL, 0.050 mmol) was added and the yield was determined by ^1^H NMR based on comparison to PhTMS as internal standard before purification by column chromatography.

For reactions in which 1.0 equiv of MgI_2_ is added, the vial is wrapped in aluminum foil for the duration of the reaction due to the light sensitivity of MgI_2_.

(1) **Preparation of Grignard Reagent** Under a N_2_ atmosphere, a three-necked flask equipped with a stir bar, reflux condenser, and Schlenk filtration apparatus was charged with magnesium turnings (1.1 g, 45 mmol). The flask and magnesium turnings were then flame-dried under vacuum and the flask was back-filled with N_2_. Anhydrous Et_2_O (7.0 mL) and a crystal of iodine (ca. 2.0 mg) were added to the flask. Freshly distilled iodomethane (1.9 mL, 31 mmol) or 4-iodoanisole as a solution in Et_2_O (4.7 g, 20. mmol, 6.7 M in Et_2_O) was slowly added over 30 min to maintain a gentle reflux. The mixture was stirred for 2 h at room temperature then filtered through the fritted Schlenk filter into a Schlenk flask under N_2_ atmosphere. The magnesium turnings were washed with Et_2_O (2 × 1.0 mL) then the Schlenk flask was sealed, removed, and placed under an N_2_ atmosphere. The resulting methylmagnesium iodide was typically between 2.4 and 3.0 M as titrated by Knochel’s method [72] and could be stored, sealed under N_2_ atmosphere or in a glovebox, for up to 4 weeks.
(2) **Preparation of (*R*-BINAP)NiCl_2_** This method was adapted from a procedure reported by Jamison [57]. To a flame-dried 50 mL round bottom flask equipped with a stir bar was added NiCl_2_·6H_2_O (0.24 g, 1.0 mmol, 1.0 equiv). The flask was placed under vacuum and flame-dried until nearly all of the nickel compound had turned from emerald green to yellow-orange. Some of the green hexahydrate is necessary for the reaction to proceed. The flask was allowed to cool to room temperature then (*R*-BINAP) (0.62 g, 1.0 mmol, 1.0 equiv) was added. The flask was then equipped with a reflux condenser and was evacuated and backfilled with N_2_. Then the solids were dissolved in MeCN (20 mL, 0.05 M) and the reaction mixture was allowed to reflux for 24 h. Upon completion, the reaction was cooled to room temperature and the black crystalline precipitate was filtered under vacuum to yield a fine black powder (0.53 g, 0.71 mmol, 71% yield).

#### 3.2.2. Characterization Data for Kumada Cross-Coupled Products

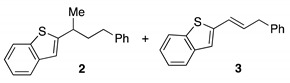



***2-(4-Phenylbutan-2-yl)benzo[b]thiophene* (2)** was prepared according to Method A. The following amounts of reagents were used: sulfonamide **1** (87 mg, 0.20 mmol, 1.0 equiv), (*R*-BINAP)NiCl_2_ (23 mg, 30. μmol, 15 mol %), PhMe (1.0 mL, 0.20 M), and methylmagnesium iodide (0.16 mL, 0.40 mmol, 2.0 equiv, 2.5 M in Et_2_O). Before purification, a ^1^H NMR yield of 54% was obtained containing 40% styrene **3** based on comparison to PhTMS as an internal standard. The residue was purified by flash chromatography (0–5% EtOAc/hexanes) to yield a mixture of the title compound and styrene **3**. To separate the major product and the styrene, an Upjohn dihydroxylation was performed [60,61]. The following amounts of reagents were used: substrate (30 mg, 0.12 mmol, 1.0 equiv), OsO_4_ (7.6 μL, 1.2 μmol, 1.0 mol %, 4% solution in H_2_O), *N*-methylmorpholine *N*-oxide (NMO) (16 mg, 0.13 mmol, 1.1 equiv), acetone (0.25 mL) and H_2_O (0.05 mL). The residue was purified by flash column chromatography to afford the title compound as a colorless oil. (13 mg, 48 μmol, 24% yield over two steps) with a small amount of styrene **3** (1.1 mg, 4.5 μmol, 2.2% yield). TLC R_f_ = 0.8 (5% EtOAc/hexanes); ^1^H NMR (500 MHz, CDCl_3_) δ 7.78 (d, *J* = 7.9 Hz, 1H), 7.68 (d, *J =* 7.9 Hz, 1H), 7.45–7.22 (m, 4H), 7.22–7.12 (m, 3H), 7.04 (s, 1H), 3.12 (sextet, *J* = 7.0 Hz, 1H), 2.73–2.50 (m, 2H), 2.12–1.92 (m, 2H), 1.41 (d, *J* = 7.0 Hz, 3H); ^13^C NMR (125.7 MHz, CDCl_3_) δ 152.5, 142.1, 140.0, 139.0, 128.5 (2C), 128.4 (2C), 125.8, 124.1, 123.5, 122.9, 122.3, 119.5, 40.5, 35.8, 33.6, 23.1; IR (neat) 2927, 1456, 904, 726 cm^−1^; HRMS (TOF MS ES+) *m/z*: [M + Na]^+^ calcd. for C_18_H_18_SNa 289.1027, found 289.1024.

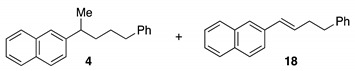



***2-(5-Phenylpentan-2-yl)naphthalene* (4)** was prepared according to Method A. The following amounts of reagents were used: sulfonamide **19** (22 mg, 50. μmol, 1.0 equiv), (*R*-BINAP)NiCl_2_ (5.6 mg, 7.5. μmol, 15 mol %), PhMe (0.25 mL, 0.20 M), and methylmagnesium iodide (40. μL, 0.10 mmol, 2.0 equiv, 2.8 M in Et_2_O). Before purification, a ^1^H NMR yield of 84% was obtained containing 13% styrene **18** based on comparison to PhTMS as an internal standard. The residue was purified by flash chromatography (100% hexanes) to yield the title compound as yellow oil (10. mg, 36 μmol, 74% yield) containing styrene **18** (1.3 mg, 5.0 μmol, 10%) and CH_2_Cl_2_ (0.8 mg, 9.4 μmol, 19%). TLC R_f_ = 0.8 (5% EtOAc/hexanes); ^1^H NMR (500 MHz, CDCl_3_) δ 7.81–7.74 (m, 3H), 7.60–7.55 (s, 1H), 7.42 (dddd, *J* = 16.3, 8.2, 6.8, 1.4 Hz, 2H), 7.33 (dd, *J* = 8.4, 1.8 Hz, 1H), 7.23 (d, *J* = 7.5 Hz, 2H), 7.17–7.09 (m, 3H), 2.88 (sextet, *J* = 7.0 Hz, 1H), 2.64–2.53 (m, 2H), 1.79–1.55 (m, 4H), 1.31 (d, *J* = 6.9 Hz, 3H); ^13^C NMR (126 MHz, CDCl_3_) δ 145.2, 142.7, 133.8, 132.3, 128.5 (2C), 128.4 (2C), 128.0, 127.7, 127.7, 125.9, 125.9, 125.8, 125.3, 125.2, 40.2, 38.0, 36.1, 29.7, 22.5; HRMS (TOF MS Cl+) *m/z*: [M]^+^ calcd. for C_21_H_22_ 274.1721, found 274.1710.

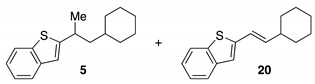



***2-(1-Cyclohexylpropan-2-yl)benzo[b]thiophene* (5)** was prepared according to Method A. The following amounts of reagents were used: sulfonamide **21** (43 mg, 0.10 mmol, 1.0 equiv), (*R*-BINAP)NiCl_2_ (11 mg, 15 μmol, 15 mol %), PhMe (0.50 mL, 0.20 M), and methylmagnesium iodide (70. μL, 0.20 mmol, 2.0 equiv, 2.8 M in Et_2_O). The residue was purified by flash column chromatography (0–15% Et_2_O/pentanes) to afford the title compound as a clear, colorless oil (12 mg, 46 μmol, 46% yield) containing styrene **20** (7.3 mg, 30. μmol, 30%) and minimal amounts of solvent. TLC R_f_ = 0.7 (100% pentanes); ^1^H NMR: (600 MHz, CDCl_3_) δ 7.83 (d, *J* = 7.2 Hz, 1H), 7.72 (d, *J* = 7.8 Hz, 1H), 7.30 (td, *J* = 7.6, 1.2 Hz, 1H), 7.26–7.22 (m, 1H), 7.00 (s, 1H), 3.27 (sextet, *J* = 12.6 Hz, 1H), 1.84–1.76 (m, 1H), 1.70–1.57 (m, 5H), 1.53–1.44 (m, 1H), 1.34 (d, *J* = 6.9 Hz, 3H), 1.32–1.23 (m, 2H), 1.19–1.11 (m, 2H), 0.96–0.85 (m, 2H).^13^C NMR (150.9 MHz, CDCl_3_) δ 153.6, 140.2, 138.9, 124.1, 123.4, 122.9, 122.4, 119.0, 66.0, 46.7, 35.2, 33.7, 33.3, 26.8, 26.4, 23.8, 15.4; HRMS (TOF MS CI+) *m/z* [M]+ calcd. for C_17_H_22_S 258.1442, found 258.1453.

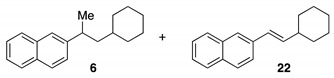



***2-(1-Cyclohexylpropan-2-yl)naphthalene* (6)** was prepared according to Method A. The following amounts of reagents were used: sulfonamide **23** (44 mg, 0.10 mmol, 1.0 equiv), (*R*-BINAP)NiCl_2_ (11 mg, 15 μmol, 15 mol %), PhMe (0.50 mL, 0.20 M), and methylmagnesium iodide (70. μL, 0.20 mmol, 2.0 equiv, 2.9 M in Et_2_O). Before purification, a ^1^H NMR yield of 69% was obtained with 22% styrene **22** based on comparison to PhTMS as an internal standard. The residue was purified by flash column chromatography (0–20% EtOAc/hexanes) to afford the title compound as a colorless oil (14 mg, 54 μmol, 54% yield) with a small amount of styrene **22** (3.7 mg, 15 μmol, 15% yield). TLC R_f_ = 0.7 (100% hexanes); ^1^H NMR (500 MHz, CDCl_3_) δ 7.85–7.73 (m, 3H), 7.60 (s, 1H), 7.47–7.38 (m, 2H), 7.35 (dd, *J* = 8.4, 1.8 Hz, 1H), 2.99 (sextet, *J* = 6.8 Hz, 1H), 1.81 (d, *J* = 13.0 Hz, 1H), 1.63 (tdd, *J* = 14.2, 8.1, 5.0 Hz, 5H), 1.50–1.41 (m, 1H), 1.28 (d, *J* = 6.9 Hz, 3H), 1.19–1.06 (m, 4H), 0.95–0.83 (m, 2H); ^13^C NMR (125.8 MHz, CDCl3) δ 145.8, 133.8, 132.3, 128.0, 127.72, 127.68, 126.0, 125.9, 125.2, 125.1, 46.3, 37.0, 35.3, 33.9, 33.5, 26.9, 26.4, 26.4, 23.1; HRMS (TOF MS CI+) *m/z*: [M]+ calcd. for C_19_H_24_ 252.1878, found 252.1868.

#### 3.2.3. Characterization Data for Ring Opening Kumada Cross-Coupled Products

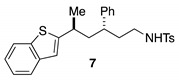



***N-(5-(benzo[b]thiophen-2-yl)-3-phenylhexyl)-4-methylbenzenesulfonamide* (7)** was prepared according to Method A. The following amounts of reagents were used: piperidine **24** (38 mg, 80 μmol, 1.0 equiv), (*R*-BINAP)NiCl_2_ (9.0 mg, 12 μmol, 15 mol %), methylmagnesium iodide (60. μL, 0.16 mmol, 2.0 equiv, 2.6 M in Et_2_O), and PhMe (0.5 mL). Before purification, a ^1^H NMR yield of 64% was obtained. The residue was purified by column chromatography (0–20% EtOAc/hexanes) to afford the title compound as pale yellow oil (23 mg, 49 μmol, 62% yield). The ratio of diastereomers was determined by integration of the resonances attributed to amine hydrogen in the ^1^H NMR spectrum. The relative configuration of **7** was assigned based on analogy to a compound that has been previously reported [12,47,48]. TLC R_f_ = 0.8 (20% EtOAc/hexanes); ^1^H NMR (400 MHz, CDCl_3_) δ 7.74 (d, *J* = 7.9 Hz, 1H), 7.67–7.56 (m, 3H), 7.37–7.13 (m, 9H), 7.07–7.00 (m, 2H), 6.93 (s, 1H), 2.85 (q, *J* = 6.7 Hz, 1H), 2.83–2.69 (m, 2H), 2.70–2.59 (m, 1H), 2.40 (s, 3H), 2.07–1.95 (m, 1H), 1.93–1.77 (m, 2H), 1.76–1.64 (m, 1H), 1.30 (d, *J* = 6.8 Hz, 3H); ^13^C NMR (151 MHz, CDCl_3_) δ 152.7, 143.5, 143.4, 140.0, 138.9, 136.9, 129.9, 129.8 (2C), 128.9 (2C), 127.7, 127.6 (2C), 127.2 (2C), 126.8, 124.2, 123.6, 123.0, 122.3, 119.1, 45.8, 41.5, 40.9, 36.6, 33.4, 22.0, 21.7; HRMS (TOF MS ES+) *m/z* [M + Na] calcd. for C_27_H_29_NO_2_S_2_Na 486.1537, found 486.1524.

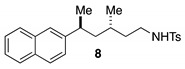



***4-Methyl-N-(3-methyl-5-(naphthalen-2-yl)hexyl)benzenesulfonamide* (8)** was prepared according to Method A. The following amounts of reagents were used: piperidine **25** (10. mg, 30. μmol, 1.0 equiv), (*R*-BINAP)NiCl_2_ (3.0 mg, 40. μmol, 15 mol %), methylmagnesium iodide (10. μL, 60. μmol, 2.0 equiv, 2.9 M in Et_2_O), and PhMe (0.30 mL). Before purification, a ^1^H NMR yield of 48% and 10:1 dr was obtained based on comparison to PhTMS as an internal standard. The residue was purified by flash column chromatography (0–15% EtOAc/hexanes) to afford the title compound as a colorless oil (5.4 mg, 14 μmol, 50% yield, 6:1 dr) with a small amount of styrene (0.6 mg, 0.2 μmol, 6%). The ratio of diastereomers was determined by integration of the resonances attributed to amine hydrogen in the ^1^H NMR spectrum. The relative configuration of the major **8** was assigned based on analogy to ring opened compound **7**. For clarity, the ^1^H NMR and ^13^C NMR data of the major and minor diastereomers have been tabulated individually.

TLC R_f_ = 0.7 (30% EtOAc/hexanes, stained with CAM); HRMS (TOF MS E+) *m/z* [M+Na] calcd. for C_24_H_29_NO_2_SNa, 418.1817; found, 418.1830.

**Major Diastereomer:**^1^H NMR (500 MHz, CDCl_3_) δ 7.81–7.76 (m, 3H), 7.69 (d, *J* = 8.3 Hz, 2H), 7.6 (s, 1H), 7.47–7.40 (m, 2H), 7.31–7.26 (m, 3H), 4.29 (t, *J* = 5.9 Hz, 1H), 3.03–2.82 (m, 3H), 2.41 (s, 3H), 1.49 (t, *J* = 6.2 Hz, 1H), 1.43–1.46 (m, 1H), 1.30–1.25 (m, 3H), 1.25 (d, *J* = 6.9 Hz, 3H), 0.79 (d, *J* = 6.5 Hz, 3H); ^13^C NMR (125.8, CDCl_3_) δ 145.1, 148.4, 133.7, 132.2, 129.7 (2C), 128.1, 127.6, 127.6, 127.1 (2C), 126.0, 125.6, 125.2, 125.0, 45.6, 41.1, 37.2, 36.5, 28.1, 29.1, 22.1, 21.5, 19.6.

**Minor Diastereomer:**^1^H NMR (500 MHz, CDCl_3_) δ 7.81–7.76 (m, 3H), 7.69 (d, *J* = 8.3, 2H), 7.63 (s, 1H), 7.47–7.40 (m, 2H), 7.31–7.26 (m, 3H), 4.39 (t, *J* = 5.5 Hz, 1H), 3.03–2.82 (m, 3H), 2.41 (s, 3H), 1.49 (t, *J* = 6.2 Hz, 1H), 1.43–1.46 (m, 1H), 1.30–1.25 (m, 3H), 1.25 (d, *J* = 6.9 Hz, 3H), 1.08 (d, *J* = 6.7, 3H); ^13^C NMR (125.8, CDCl_3_) δ 145.1, 148.4, 133.7, 132.2, 129.7 (2C), 128.1, 127.6, 127.6, 127.1 (2C), 126.0, 125.7, 125.2, 125.0, 45.6, 41.5, 37.2, 36.5, 29.7, 29.1, 22.1, 21.5, 19.6.

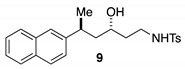



***N-(3-hydroxy-5-(naphthalen-2-yl)hexyl)-4-methylbenzenesulfonamide* (9)** was prepared according to Method A. The following amounts of reagents were used: piperidine **26** (93 mg, 0.20 mmol, 1.0 equiv), (*R*-BINAP)NiCl_2_ (23 mg, 30. μmol, 15 mol%), methylmagnesium iodide (0.14 mL, 0.40 mmol, 2.0 equiv, 2.9 M in Et_2_O), and PhMe (2.0 mL). The residue was purified by flash column chromatography (0–15% EtOAc/hexanes) to afford the title compound as a colorless oil (42 mg, 0.11 mmol, 53% yield, 5:1 dr). The ratio of diastereomers was determined by integration of the resonances attributed to amine hydrogen in the ^1^H NMR spectrum. The relative configuration of the major **9** was assigned based on analogy to ring opened compound **7**. For clarity, the ^1^H NMR and ^13^C NMR data of the major and minor diastereomers have been tabulated individually.

TLC R_f_ = 0.5 (30% EtOAc/hexanes, stained with CAM); HRMS (TOF MS E+) *m/z* [M + Na] calcd. for C_23_H_27_NO_3_SNa, 420.1609; found, 420.1604.

**Major Diastereomer:**^1^H NMR (500 MHz, CDCl_3_) δ 7.79–7.75 (m, 3H), 7.69 (d, *J* = 8.15 Hz, 2H), 7.59 (s, 1H), 7.43 (appar quint, *J* = 7.44 Hz, 2H), 7.31 (d, *J* = 8.82 Hz, 1H), 7.23 (d, *J* = 8.17 Hz, 2H), 5.27 (t, *J* = 5.55 Hz, 1H), 3.74–3.70 (m, 1H), 3.12–3.06 (m, 1H), 3.01–2.93 (m, 2H), 2.37 (s, 3H), 1.89 (br s, 1H), 1.86–1.80 (m, 1H), 1.72–1.62 (m, 2H), 1.53–1.46 (m, 1H), 1.28 (d, *J* = 7.23 Hz, 3H); ^13^C NMR (125.8, CDCl_3_) δ 144.4, 143.3, 136.9, 133.7, 132.3, 129.7 (2C), 128.4, 127.7, 127.6, 127.1 (2C), 126.1, 125.5, 124.4, 125.0, 69.2, 45.9, 40.8, 37.0, 36.0, 22.2, 21.5.

**Minor Diastereomer:**^1^H NMR (500 MHz, CDCl_3_) δ 7.79–7.75 (m, 3H), 7.65 (d, *J* = 8.14 Hz, 2H), 7.59 (s, 1H), 7.43 (appar quint, *J* = 7.44 Hz, 2H), 7.29 (d, *J* = 8.91 Hz, 1H), 7.19 (d, *J* = 8.05 Hz, 2H), 5.19 (t, *J* = 5.57 Hz, 1H), 3.43–3.39 (m, 1H), 3.12–3.06 (m, 1H), 2.91–2.82 (m, 2H), 2.35 (s, 3H), 1.86–1.80 (m, 1H), 1.76 (br s, 1H), 1.72–1.62 (m, 2H), 1.53–1.46 (m, 1H), 1.29 (d, *J* = 6.40 Hz, 3H); ^13^C NMR (125.8, CDCl_3_) δ 143.7, 143.3, 136.8, 133.7, 132.3, 129.7 (2C), 128.4, 127.7, 127.6, 127.1 (2C), 126.1, 125.5, 124.4, 125.0, 68.7, 45.6, 40.8, 36.45, 36.42 23.2, 21.5.

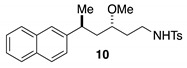



***N-(3-methoxy-5-(naphthalen-2-yl)hexyl)-4-methylbenzenesulfonamide* (10)** was prepared according to Method A. The following amounts of reagents were used: piperidine **12** (58 mg, 0.15 mmol, 1.0 equiv), (*R*-BINAP)NiCl_2_ (15 mg, 20. μmol, 15 mol %), methylmagnesium iodide (0.11 mL, 0.30 mmol, 2.0 equiv, 2.8 M in Et_2_O), and PhMe (1.5 mL). The residue was purified by flash column chromatography (0–25% EtOAc/hexanes) to afford the title compound as a colorless oil (32 mg, 80. μmol, 52% yield, 5:1 dr). The ratio of diastereomers was determined by integration of the resonances attributed to amine hydrogen in the ^1^H NMR spectrum. The relative configuration of the major **10** was assigned based on analogy to ring opened compound **7**. For clarity, the ^1^H NMR and ^13^C NMR data of the major and minor diastereomers have been tabulated individually.

TLC R_f_ = 0.4 (30% EtOAc/hexanes, stained with CAM); HRMS (TOF MS E+) *m/z* [M + Na] calcd. for C_24_H_29_NO_3_SNa, 424.1766; found, 434.1775.

**Major Diastereomer:**^1^H NMR (500 MHz, CDCl_3_) δ 7.81–7.76 (m, 3H), 7.68 (d, *J* = 8.37 Hz, 2H), 7.55 (s, 1H), 7.47–7.40 (m, 2H), 7.29 (dd, *J* = 8.48, 1.70 Hz, 1H), 7.26–7.24 (m, 1H), 7.21 (d, *J* = 7.97 Hz, 1H), 5.11 (t, *J* = 5.53 Hz, 1H), 3.15 (s, 3H), 3.08–2.91 (m, 3H), 2.86 (appar sextet, *J* = 7.37 Hz, 1H), 2.34 (s, 3H), 1.96 (ddd, *J* = 14.3, 8.50, 5.78 Hz, 1H), 1.82–1.76 (m, 1H), 1.58–1.50 (m, 2H), 1.28 (d, *J* = 6.98 Hz, 3H); ^13^C NMR (125.8, CDCl_3_) δ 144.1, 143.2, 136.8, 133.6, 132.3, 129.7 (2C), 128.3, 127.7, 127.6, 127.1 (2C), 126.1, 125.4, 125.24, 125.17 78.4, 56.3, 40.9, 40.5, 36.3, 32.0, 22.9, 21.5.

**Minor Diastereomer:**^1^H NMR (500 MHz, CDCl_3_) δ 7.81–7.76 (m, 3H), 7.70 (d, *J* = 8.60 Hz, 2H), 7.55 (s, 1H), 7.47–7.40 (m, 2H), 7.29 (dd, *J* = 8.48, 1.70 Hz, 1H), 7.26–7.24 (m, 1H), 7.21 (d, *J* = 7.97 Hz, 1H), 5.03 (t, *J* = 5.52 Hz, 1H), 3.20 (s, 3H), 3.08–2.91 (m, 3H), 2.86 (appar sextet, *J* = 7.37 Hz, 1H), 2.38 (s, 3H), 1.96 (ddd, *J* = 14.3, 8.50, 5.78 Hz, 1H), 1.71–1.66 (m, 1H), 1.47–1.40 (m, 2H), 1.27 (d, *J* = 7.32 Hz, 3H) ^13^C NMR (125.8, CDCl_3_) δ 144.2, 143.2, 136.9, 133.6, 132.3, 129.7 (2C), 128.3, 127.7, 127.6, 127.1 (2C), 126.1, 125.52, 125.46 125.4, 78.4, 56.3, 40.9, 40.5, 36.3, 32.0, 22.9, 21.5.

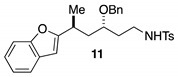



***N-(5-(benzofuran-2-yl)-3-(benzyloxy)hexyl)-4-methylbenzenesulfonamide* (11)** was prepared according to Method A. The following amounts of reagents were used: piperidine **27** (78 mg, 0.17 mmol, 1.0 equiv), (*R*-BINAP)NiCl_2_ (22 mg, 30. μmol, 15 mol %), methylmagnesium iodide (0.23 mL, 0.68 mmol, 4.0 equiv, 2.9 M in Et_2_O), and PhMe (1.5 mL). The residue was purified by flash column chromatography (0–15% EtOAc/hexanes) to afford a mixture of the title compound and styrene. To separate the major product and the styrene, a dihydroxylation was performed. The following amounts of reagents were used: AD-mix-β (52 mg, 1.4 g/mmol), *t*-BuOH (1.0 mL), and H_2_O (1.0 mL). The residue was purified by flash column chromatography to afford the title compound as a colorless oil. (7.0 mg, 15 μmol, 8.6% yield over two steps, 5:1 dr). The ratio of diastereomers was determined by integration of the resonances attributed to amine hydrogen in the ^1^H NMR spectrum. The relative configuration of the major **11** was assigned based on analogy to ring opened compound **7**. When the reaction was performed with 2.0 equivalents of methylmagnesium iodide, a ^1^H NMR yield of 41% was obtained based on comparison to PhTMS as an internal standard before purification. For clarity, the ^1^H NMR and ^13^C NMR data of the major and minor diastereomers have been tabulated individually.

TLC R_f_ = 0.5 (30% EtOAc/hexanes, stained with CAM); HRMS (TOF MS E+) *m/z* [M + Na] calcd. for C_28_H_31_NO_4_SNa, 500.1872; found, 500.1861.

**Major Diastereomer:**^1^H NMR (500 MHz, CDCl_3_) δ 7.64 (d, *J* = 7.9 Hz, 2H), 7.48 (d, *J* = 7.4 Hz, 1H), 7.42 (d, *J* = 7.9 Hz, 1H), 7.27–7.16 (m, 8H), 6.33 (s, 1H), 4.79 (t, *J* = 6.0 Hz, 1H), 4.45 (d, *J* = 11.5 Hz, 1H), 4.31 (d, *J* = 11.4 Hz, 1H), 3.49–3.42 (m, 1H), 3.09–2.95 (m, 3H), 2.38 (s, 3H), 2.12 (appar quint, *J* = 6.9 Hz, 1H), 1.87–1.81 (m, 1H), 1.65–1.54 (m, 3H), 1.29 (d, *J* = 6.9 Hz, 3H) ^13^C NMR (125.8, CDCl_3_) δ 162.6, 154.5, 143.3, 137.9, 136.9, 129.7 (2C), 128.5 (2C), 128.1 (2C), 127.9, 127.1 (2C), 123.4, 122.6, 120.5, 110.9, 101.1, 75.0, 70.7, 40.2, 39.1, 32.9, 30.3, 29.7, 21.5, 19.7.

**Minor Diastereomer:**^1^H NMR (500 MHz, CDCl_3_) δ 7.80 (d, *J* = 7.9 Hz, 2H), 7.75 (d, *J* = 7.7 Hz, 1H), 7.58 (d, *J* = 8.4 Hz, 1H), 7.27–7.16 (m, 8H), 6.25 (s, 1H), 4.79 (t, *J* = 6.0 Hz, 1H), 4.40 (s, 2H), 3.53–3.48 (m, 1H), 3.09–2.95 (m, 3H), 2.73 (appar quint, *J* = 6.5 Hz, 1H), 2.40 (s, 3H), 1.80–1.77 (m, 1H), 1.65–1.60 (m, 3H), 0.88 (d, *J* = 7.0 Hz, 3H); ^13^C NMR (125.8, CDCl_3_) δ 162.6, 154.5, 143.3, 137.9, 136.9, 129.4 (2C), 128.7 (2C), 128.3 (2C), 127.9, 127.1 (2C), 123.4, 122.6, 120.5, 110.9, 101.4, 75.6, 71.4, 39.9, 39.1, 32.8, 30.5, 29.7, 21.5, 20.2.

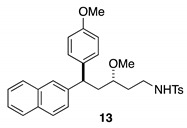



***N-3-methoxy-5-(4-methoxyphenyl)-5-(naphthalen-2-yl)pentyl)-4-methylbenzenesulfonamide* (13)** was prepared according to Method A. The following amounts of reagents were used: piperidine **12** (37 mg, 90 μmol, 1.0 equiv), Ni(dppe)Cl_2_ (7.0 mg, 10. μmol, 15 mol %), (4-methoxyphenyl)magnesium iodide (0.11 mL, 0.18 mmol, 2.0 equiv, 1.7 M in Et_2_O), and PhMe (0.90 mL). Before purification, a ^1^H NMR yield of 56% was obtained based on comparison to PhTMS as an internal standard. The residue was purified by flash column chromatography (0–20% EtOAc/hexanes) to afford the title compound as a yellow oil (26 mg, 50. μmol, 58% yield, 3:1 dr). The ratio of diastereomers was determined by integration of the resonances attributed to methyl hydrogens of the tosyl group in the ^1^H NMR spectrum. The relative configuration of the major **13** was assigned based on analogy to ring opened compound **7**. For clarity, the ^1^H NMR and ^13^C NMR data of the major and minor diastereomers have been tabulated individually.

TLC R_f_ = 0.3 (30% EtOAc/hexanes, stained with CAM); HRMS (TOF MS E+) *m/z* [M + H] calcd. for C_30_H_34_NO_4_S, 504.2209; found, 504.2206.

**Major Diastereomer:**^1^H NMR (500 MHz, CDCl_3_) δ 7.76 (d, *J* = 7.3 Hz, 2H), 7.70–7.69 (m, 3H), 7.61 (s, 1H), 7.43 (dt, *J* = 20.4, 7.3 Hz, 2H), 7.29–7.19 (m, 3H), 7.13 (d, *J* = 8.5 Hz, 2H), 6.81 (d, *J* = 8.6 Hz, 2H), 5.09–5.-5 (m, 1H), 4.12 (t, *J* = 7.8 Hz, 1H), 3.76 (s, 3H), 3.20 (s, 3H), 3.13–3.09 (m, 1H), 3.05–2.97 (m, 2H), 2.30 (s, 3H), 2.25 (appar sext, *J* = 7.3 Hz, 1H), 2.02–1.97 (m, 1H), 1.85–1.80 (m, 1H), 1.58–1.52 (m, 1H); ^13^C NMR (125.8, CDCl_3_) δ 158.2, 143.4, 142.4, 136.8, 135.2, 133.6, 129.7 (2C), 129.0 (2C), 128.8, 128.3, 127.8, 127.7, 127.2 (2C), 126.5, 126.2, 125.6, 125.5, 114.1 (2C), 78.0, 56.7, 55.3, 46.5, 40.3, 39.1, 31.8, 21.5.

**Minor Diastereomer:**^1^H NMR (500 MHz, CDCl_3_) δ 7.76 (d, *J* = 7.3 Hz, 2H), 7.73–7.70 (m, 3H), 7.63 (s, 1H), 7.43 (dt, *J* = 20.4, 7.3 Hz, 2H), 7.29–7.19 (m, 3H), 7.14 (d, *J* = 7.7 Hz, 2H), 6.81 (d, *J* = 8.8 Hz, 2H), 5.09–5.-5 (m, 1H), 4.12 (t, *J* = 7.8 Hz, 1H), 3.76 (s, 3H), 3.19 (s, 3H), 3.13–3.09 (m, 1H), 3.05–2.97 (m, 2H), 2.35 (s, 3H), 2.25 (appar sext, *J* = 7.3 Hz, 1H), 2.06–2.03 (m, 1H), 1.76–1.78 (m, 1H), 1.58–1.52 (m, 1H); ^13^C NMR (125.8, CDCl_3_) δ 158.2, 143.4, 142.0, 136.9, 136.5, 133.6, 129.7 (2C), 129.0 (2C), 128.8, 128.4, 127.8, 127.7, 127.2 (2C), 126.5, 126.2, 125.9, 125.6, 114.1 (2C), 78.0, 56.7, 55.3, 46.5, 40.3, 39.1, 31.9, 21.6.

#### 3.2.4. General Procedures for Synthesis of Starting Materials

##### Method B: Condensation Reaction

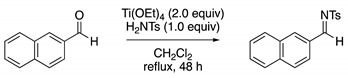



This method was adapted from a procedure reported by Ruano et al. [73]. A flame-dried two-neck flask equipped with a stir bar, condenser, septum and N_2_ inlet was charged with aldehyde (1.0 equiv), and *p*-toluenesulfonamide (1.0 equiv) and CH_2_Cl_2_ (330 mL). Then Ti(OEt)_4_ (2.0 equiv) was added dropwise. The deep orange solution was brought to reflux (~45 °C) and allowed to stir for 48 h. The solution was cooled to room temperature and was quenched with H_2_O. The mixture was vacuum filtered and the filtrate was concentrated in vacuo.

##### Method C: Methylation of Sulfonamide with Methyl Iodide

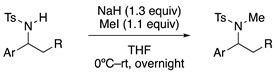



This method was adapted from a procedure reported by Jarvo [12,47,48]. To a suspension of NaH (1.3 equiv) in THF (0.10 M) was added a solution of sulfonamide (1.0 equiv) in THF (0.15 M) at 0 °C. The mixture was warmed to rt and allow to stir for 1 h before the addition of iodomethane (1.1 equiv). The reaction was allowed to stir overnight at rt. The excess NaH was quenched with sat. NH_4_Cl and the solution was extracted with EtOAc (×3). The combined organic layers were washed with brine, dried over Na_2_SO_4_, concentrated in vacuo, and purified by flash column chromatography.

##### Method D: Fe-Catalyzed Formal [4+2] Cycloaddition

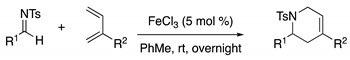



This method was adapted from a procedure reported by Matsubara [59]. To a flame-dried round-bottom flask equipped with a stir bar was added imine (1.0 equiv), FeCl_3_ (5.0 mol%), and PhMe (0.1 M). Once the solution was homogenous, diene (2.0 equiv) was added. The reaction mixture was allowed to stir at rt overnight. After completion, the reaction mixture was filtered through a short pad of silica, washed with excess ethyl acetate, and concentrated in vacuo.

##### Method E: Pd/C Reduction of Alkenes

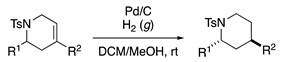



A flame-dried round-bottom flask with stir bar was charged with palladium on carbon (1.0 mg/3.5 mmol of substrate), flushed with N_2_, and capped with septum. Slowly, DCM was added, until Pd/C was fully submerged. Then MeOH (0.2 M in substrate), and alkene (1.0 equiv) were added. Vacuum was pulled on the flask until the solvent began to bubble, at which point the flask was backfilled with N_2_ (×3). An H_2_ balloon was added and the reaction mixture was allowed to stir vigorously until complete by ^1^H NMR. The balloon was then removed, and the flask was purged with N_2_ for 30 min. The septum was removed, and the reaction mixture was filtered through Celite using MeOH (100 mL). The collected solvent was then concentrated in vacuo.

##### Method F: TFA Mediated Aza-Prins Cyclization

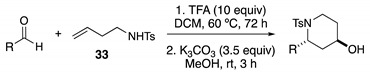



This method was adapted from a procedure reported by Sabitha [60,61]. To a flame-dried pressure tube equipped with a stir bar was added aldehyde (1.0 equiv), homoallylic sulfonamide **33** (1.1 equiv), and CH_2_Cl_2_ (0.10 M). Then trifluoroacetic acid (10.0 equiv) was added slowly via syringe. The solution was warmed to 60 °C and allowed to stir for 72 h. The solution was then cooled to rt and quenched with saturated aq. NaHCO_3_. Then the pH was adjusted to >7 by the addition of Et_3_N. The solution was transferred to a separatory funnel, and the aqueous layer was extracted with CH_2_Cl_2_ (×3). The combined organic layers were washed with brine, dried over Na_2_SO_4_, filtered and concentrated in vacuo. The residue was then redissolved in MeOH, and K_2_CO_3_ (3.5 equiv) was added to the flask and the slurry was allowed to stir at rt for 3 h. The solvent was removed under reduced pressure, then H_2_O was added and the residue was transferred to a separatory funnel. The aqueous layer was extracted with EtOAc (×3). The combined organic layers were washed with brine, dried over Na_2_SO_4_, filtered and concentrated in vacuo.

##### Method G: Alkylation of Secondary Alcohol

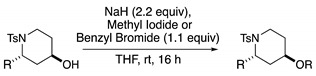



This method was adapted from a procedure reported by Yang [74]. In a glovebox, to a flame-dried round bottom flask equipped with a stir bar was added NaH (2.2 equiv). The flask was removed from the glovebox and NaH was dissolved in THF (0.2 M). Alcohol (1.0 equiv) was added dropwise as a solution in THF (0.3 M) and the reaction mixture was allowed to stir at rt for 1 h. Methyl iodide or benzyl bromide was then added dropwise to the stirring slurry and the reaction mixture was allowed to stir at rt overnight. The reaction was then quenched with saturated aq. NH_4_Cl and extracted with EtOAc (×3). The combined organic layers were washed with brine, dried over Na_2_SO_4_ and concentrated in vacuo.

#### 3.2.5. Synthesis and Characterization Data of Sulfonamide Starting Materials

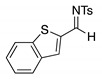



***N-(benzo[b]thiophen-2-ylmethylene)-4-methylbenzenesulfonamide* (28)** was prepared according to Method B. The following amounts of reagents were used: benzo[*b*]thiophene-2-carbaldehyde (3.2 g, 20. mmol, 1.0 equiv), *p*-toluenesulfonamide (3.1 g, 20. mmol, 1.0 equiv), Ti(OEt)_4_ (8.4 mL, 40. mmol, 2.0 equiv), and CH_2_Cl_2_ (330 mL). The residue was purified by flash column chromatography (5–25% EtOAc/hexanes) to yield the title compound as a pale yellow solid (5.0 g, 16 mmol, 80%). m.p. 148–150 °C; TLC R_f_ = 0.5 (25% EtOAc/hexanes); ^1^H NMR (500 MHz, CDCl_3_) δ 9.23 (s, 1H), 8.02 (s, 1H), 7.90 (d, *J* = 8.3, 3H), 7.86 (d, *J* = 8.2, 1H), 7.50 (td, *J* = 8.3, 1.2, 1H), 7.42 (td, *J* = 8.4, 1.2, 1H), 7.35 (d, *J* = 8.4, 2H), 2.44 (s, 3H); ^13^C NMR (126 MHz, CDCl_3_) δ 163.3, 144.8, 143.6, 138.9, 138.3, 137.3, 135.3, 130.0 (2C), 128.7, 128.2 (2C), 126.1, 125.5, 123.2, 21.8; IR (neat) 3259, 2921, 1566, 1305, 1292, 1152, 1087, 752 cm^−1^; HRMS (TOF MS ES+) *m*/*z* calcd. for C_16_H_13_NO_2_S_2_ [M + Na]^+^ 338.0285, found 338.0283.

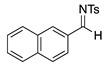



***4-Methyl-N-(naphthalen-2-ylmethylene)benzenesulfonamide* (29)** was prepared according to Method B. The following amounts of reagents were used: 2-naphthaldehyde (0.31 g, 2.0 mmol, 1.0 equiv), *p*-toluenesulfonamide (0.34 g, 2.0 mmol, 1.0 equiv), Ti(OEt)_4_ (0.59 mL, 4.0 mmol, 2.0 equiv), and CH_2_Cl_2_ (33 mL). The residue was purified by flash column chromatography (0–25% EtOAc/hexanes) to yield the title compound as a pale yellow solid (0.31 g, 1.0 mmol, 50% yield). Analytical data are consistent with literature values. [75] ^1^H NMR (400 MHz, CDCl_3_) δ 9.18 (s, 1H), 8.34 (s, 1H), 8.04 (dd, *J* = 8.6, 1.7 Hz, 1H), 7.98–7.86 (m, 5H), 7.67–7.55 (m, 2H), 7.36 (d, *J* = 8.0 Hz, 2H), 2.44 (s, 3H).

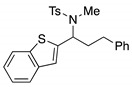



***N-(1-(benzo[b]thiophen-2-yl)-3-phenylpropyl)-N,4-dimethylbenzenesulfonamide* (1)** was prepared according to Method C. The following amounts of reagents were used: *N*-(1-(benzo[*b*]thiophen-2-yl)-3-phenylpropyl)-4-methylbenzenesulfonamide (680 mg, 1.6 mmol, 1.0 equiv), NaH (50. mg, 2.1 mmol, 1.3 equiv), methyl iodide (0.11 mL, 1.8 mmol, 1.1 equiv) and THF (30 mL). The residue was purified by flash column chromatography (5–25% EtOAc/hexanes) to yield the title compound as a yellow oil (0.52 g, 1.2 mmol, 68% yield) TLC R_f_ = 0.8 (25% EtOAc/hexanes); ^1^H NMR (500 MHz, CDCl_3_) δ 7.73 (d, *J* = 7.9 Hz, 1H), 7.70–7.62 (m, 3H), 7.40–7.27 (m, 4H), 7.27–7.19 (m, 3H), 7.16 (d, *J* = 7.7 Hz, 2H), 7.06 (s, 1H), 5.42 (t, *J* = 7.5 Hz, 1H), 2.77 (s, 3H), 2.69 (qdd, *J* = 14.1, 10.4, 5.9 Hz, 2H), 2.40 (s, 3H), 2.30 (dddd, *J* = 13.7, 10.3, 7.2, 5.4 Hz, 1H), 2.08 (dddd, *J* = 13.9, 10.5, 7.8, 6.3 Hz, 1H); ^13^C NMR (125.7 MHz, CDCl_3_) δ 143.31, 143.30, 141.0, 139.6, 139.2, 137.0, 129.6 (2C), 128.6 (2C), 128.5 (2C), 127.3 (2C), 126.3, 124.5, 124.4, 123.6, 122.9, 122.3, 56.5, 34.6, 33.0, 29.0, 21.6; HRMS (TOF MS ES+) *m/z:* [M + Na]^+^ calcd. for C_25_H_25_NO_2_S_2_Na 458.1224, found 458.1235.

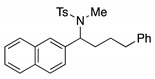



***N,4-dimethyl-N-(1-(naphthalen-2-yl)-4-phenylbutyl)benzenesulfonamide* (19)** was prepared according to Method C. The following amounts of reagents were used: 4-methyl-*N*-(1-(naphthalen-2-yl)-4-phenylbutyl)benzenesulfonamide (0.50 g, 1.2 mmol, 1.0 equiv), NaH (36 mg, 1.5 mmol, 1.3 equiv), methyl iodide (80. μL, 1.3 mmol, 1.1 equiv) and THF (23 mL). The residue was purified by flash column chromatography (0–25% EtOAc/hexanes) to yield the title compound as a pale yellow solid (0.42 g, 0.95 mmol, 82% yield). TLC R_f_ = 0.3 (25% EtOAc/hexanes); ^1^H NMR (500 MHz, CDCl_3_) δ 7.85 (td, *J* = 8.1, 7.1, 4.1 Hz, 2H), 7.80 (d, *J* = 8.5 Hz, 1H), 7.75 (dt, *J* = 6.1, 3.7 Hz, 1H), 7.72 (d, *J* = 8.1 Hz, 2H), 7.58–7.50 (m, 3H), 7.46–7.39 (m, 1H), 7.36–7.31 (m, 2H), 7.26 (t, *J* = 8.3 Hz, 2H), 7.20 (d, *J* = 7.3 Hz, 2H), 5.34 (t, *J* = 7.7 Hz, 1H), 2.72 (td, *J* = 7.5, 2.3 Hz, 2H), 2.68 (s, 3H), 2.44 (s, 3H), 2.16–2.06 (m, 1H), 1.88 (ddd, *J* = 15.6, 14.0, 7.6 Hz, 1H), 1.70 (quint, *J* = 9.0 Hz, 2H); ^13^C NMR (126 MHz, CDCl_3_) δ 143.1, 141.9, 135.9, 133.1, 132.9, 129.9, 129.6 (2C), 128.53 (2C), 128.46 (2C), 128.3, 128.1, 127.7, 127.6, 127.3 (2C), 126.7, 126.4, 126.3, 126.0, 60.0, 35.5, 30.0, 28.9, 28.3, 21.6; HRMS (TOF MS ES+) *m/z*: [M + Na] calcd. for C_28_H_29_NO_2_S 466.1817, found 466.1816.

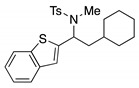



***N-(1-benzo[b]thiophen-2-yl)-2-cyclohexylethyl)-N,4-dimethylbenzenesulfonamide* (21)** was prepared according to Method C. The following amounts of reagents were used: *N*-(1-(benzo[*b*]thiophen-2-yl)-2-cyclohexylethyl)-4-methylbenzenesulfonamide (170 mg, 0.41 mmol, 1.0 equiv), NaH (31 mg, 0.53 mmol, 1.3 equiv), methyl iodide (30. μL, 0.45 mmol, 1.1 equiv), and THF (8.2 mL). The residue was purified by flash column chromatography (20% EtOAc/hexanes) to afford the title compound as a clear, colorless oil (170 mg, 0.40 mmol, 98% yield). TLC R_f_ = 0.40 (10% EtOAc/hexanes); ^1^H NMR (500 MHz, CDCl3) δ 7.73 (d, *J* = 7.8 Hz, 1H), 7.70 (d, *J* = 8.3 Hz, 2H), 7.66 (d, *J* = 7.2 Hz, 1H), 7.30 (dtd, *J* = 16.4, 7.2, 1.3 Hz, 2H), 7.24 (d, *J* = 8.0 Hz, 2H), 7.05 (s, 1H), 5.50 (t, *J* = 7.6, 1H), 2.71 (s, 3H), 2.39 (s, 3H), 1.92–1.78 (m, 2H), 1.76–1.59 (m, 5H), 1.33–1.23 (m, 1H), 1.20–1.10 (m, 3H), 1.04–0.83 (m, 2H); ^13^C NMR (125.8 MHz, CDCl3) δ 144.1, 143.0, 139.4, 139.1, 137.0, 129.4 (2C), 127.2 (2C), 124.2, 123.3, 122.3, 122.5, 122.1, 53.9, 40.3, 34.0, 33.4, 33.0, 28.7, 26.3, 26.0, 25.9, 21.4; HRMS (TOF MS ES+) *m/z* [M + Na]^+^ calcd. for C_24_H_29_NO_2_S_2_Na 450.1537, found 450.1530.

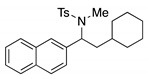



***N-(2-cyclohexyl-1(napthalen-2-yl)ethyl)-N,4-dimethylbenzenesulfonamide* (23)** was prepared according to Method C. The following amounts of reagents were used: *N*-(2-cyclohexyl-1-(naphthalen-2-yl)ethyl)-4-methylbenzenesulfonamide (190 mg, 0.46 mmol, 1.0 equiv), NaH (17 mg, 0.70 mmol, 1.5 equiv), methyl iodide (40. μL, 0.60 mmol, 1.1 equiv), and THF (11 mL). The residue was purified by flash column chromatography (0–10% EtOAc/hexanes) to afford the title compound as a yellow oil (180 mg, 0.43 mmol, 86% yield). TLC R_f_ = 0.4 (10% EtOAc/hexanes); 1H NMR (500 MHz, CDCl3) δ 7.87 (d, *J* = 9.2 Hz, 1H), 7.82 (dd, *J* = 11.3, 7.5 Hz, 2H), 7.75 (d, *J* = 8.2 Hz, 2H), 7.65 (br s, 1H), 7.56–7.51 (m, 2H), 7.49 (dd, *J* = 8.5, 1.5 Hz, 1H), 7.30 (d, *J* = 8.0 Hz, 2H), 5.45 (br t, *J* = 7.7 Hz, 1H), 2.72 (s, 3H), 2.46 (s, 3H), 2.02–1.88 (m, 2H), 1.81 (d, *J* = 12.5 Hz, 1H), 1.78–1.72 (m, 2H), 1.72–1.60 (m, 2H), 1.26– 1.12 (m, 4H), 1.09–0.87 (m, 2H); 13C NMR (125.8 MHz, CDCl3) δ 143.1, 137.6, 136.3, 133.1, 132.9, 129.6 (2C), 128.2, 128.1, 127.6, 127.3 (2C), 126.8, 126.6, 126.2, 57.5 (2C), 38.2, 34.2, 33.5, 33.4, 28.9, 26.6, 26.22, 26.21, 21.6; HRMS (TOF MS ES+) *m/z* [M + Na]+ calcd. for C26H31NO2SNa 444.1973, found 444.1968.

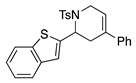



***2-(Benzo[b]thiophen-2-yl)-4-phenyl-1-tosyl-1,2,3,6-tetrahydropyridine* (30)** was prepared according to Method D. The following amounts of reagents were used: imine **28** (240 mg, 0.75 mmol, 1.0 equiv), buta-1,3-dien-2-ylbenzene (190 mg, 1.5 mmol, 2.0 equiv) [76], FeCl_3_ (6.0 mg, 40. μmol, 5.0 mol %), and PhMe (10. mL). The residue was purified by column chromatography (0–10% EtOAc/hexanes) to afford the title compound as a yellow oil (154 mg, 0.34 mmol, 46% yield). TLC R_f_ = 0.5 (20% EtOAc/hexanes); ^1^H NMR (500 MHz, CDCl_3_) δ 7.71 (d, *J* = 8.1 Hz, 2H), 7.68 (d, *J* = 7.8 Hz, 1H), 7.62 (d, *J* = 7.8 Hz, 1H), 7.36–7.22 (m, 8H), 7.18 (d, *J* = 8.0 Hz, 2H), 7.10 (s, 1H), 5.95 (s, 1H), 5.80 (d, *J* = 6.1 Hz, 1H), 4.35 (dt, *J* = 18.6, 3.5 Hz, 1H), 3.84 (dq, *J* = 18.7, 2.8 Hz, 1H), 2.96 (ddt, *J* = 16.3, 6.4, 3.2 Hz, 1H), 2.87 (d, *J* = 17.2 Hz, 1H), 2.33 (s, 3H); ^13^C NMR (126 MHz, CDCl_3_) δ 144.9, 144.6, 143.6, 140.0, 139.9, 129.9 (2C), 129.2, 128.8 (2C), 128.4, 127.4 (2C), 126.8 (2C), 124.5, 124.3, 123.5, 122.3, 122.3, 53.9, 42.2, 37.1, 36.7, 31.7, 21.7.

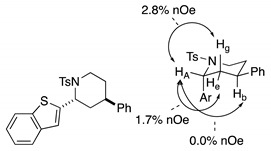



***2-(Benzo[b]thiophen-2-yl)-4-phenyl-1-tosylpiperidine* (24)** was prepared according to Method E. The following amounts of reagents were used: substrate **30** (100 mg, 0.22 mmol, 1.0 equiv), Pd/C (20 mg), DCM (2.0 mL) and MeOH (5.0 mL). The residue was purified by column chromatography (0–10% EtOAc/hexanes) to afford the title compound as a pale yellow oil (24 mg, 53 μmol, 25% yield, >20:1 dr trans:cis). The dr was determined based on the integration of the resonances attributed to the benzylic hydrogens in the ^1^H NMR spectrum. The relative configuration was assigned based on nOe analysis. TLC R_f_ = 0.5 (20% EtOAc/hexanes); ^1^H NMR (500 MHz, CDCl_3_) δ 7.81 (d, *J* = 8.4 Hz, 2H), 7.77 (d, *J* = 7.9 Hz, 1H), 7.68 (d, *J* = 7.7 Hz, 1H), 7.40–7.26 (m, 6H), 7.23–7.18 (m, 1H), 7.13 (d, *J* = 1.5 Hz, 1H), 7.06 (d, *J* = 7.4 Hz, 2H), 5.70 (d, *J* = 5.2 Hz, 1H), 4.03 (d, *J* = 14.0 Hz, 1H), 3.34 (ddd, *J* = 14.1, 12.7, 3.0 Hz, 1H), 2.93 (tt, *J* = 12.6, 3.6 Hz, 1H), 2.43 (s, 3H), 2.33 (d, *J* = 13.0 Hz, 1H), 2.01 (td, *J* = 13.5, 5.5 Hz, 1H), 1.68 (d, *J* = 12.5 Hz, 1H), 1.61 (td, *J* = 12.7, 4.4 Hz, 1H); ^13^C NMR (151 MHz, CDCl_3_) δ 144.8, 144.5, 139.9, 139.8, 129.8 (2C), 129.1, 128.7 (2C), 128.3, 127.3 (2C), 126.7 (2C), 124.4, 124.2, 123.4, 122.2, 122.2, 76.8, 53.8, 42.1, 36.9, 36.6, 31.6, 21.6; HRMS (TOF MS ES+) *m/z* [M+Na] calcd. for C_26_H_25_NO_2_S_2_Na 470.1224, found 470.1228.

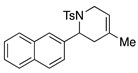



***4-Methyl-2-(naphthalen-2-yl)-1-tosyl-1,2,3,6-tetrahydropyridine* (31)** was prepared according to Method D. The following amounts of reagents were used: imine **28** (0.31 g, 1.0 mmol, 1.0 equiv), isoprene (1.5 mL, 15 mmol, 15 equiv), FeCl_3_ (16 mg, 0.10 mmol, 10. mol %), and PhMe (10. mL, 0.10 M). The residue was purified by flash column chromatography (0–5% EtOAc/hexanes) to afford the title compound as a yellow oil (150 mg, 0.40 mmol, 40% yield). TLC R_f_ = 0.5 (20% EtOAc/hexanes); ^1^H NMR (500 MHz, CDCl_3_) δ 7.78–7.71 (m, 3H), 7.69 (d, *J* = 8.3 Hz, 2H), 7.56 (s, 1H), 7.48–7.40 (m, 3H), 7.20 (d, *J* = 8.1 Hz, 2H), 5.43 (d, *J* = 3.5 Hz, 1H), 5.29 (s, 1H), 4.11 (d, *J* = 18.0 Hz, 1H), 3.35 (d, *J* = 18.1 Hz, 1H), 2.40–2.30 (m, 2H), 2.35 (s, 3H), 1.68 (s, 3H).

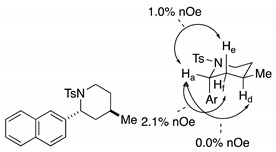



***4-Methyl-2-(naphthalen-2-yl)-1-tosylpiperidine* (25)** was prepared according to Method E. The following amounts of reagents were used: substrate **31** (53 mg, 0.14 mmol, 1.0 equiv), Pd/C (27 mg), DCM (1.0 mL) and MeOH (1.0 mL). The residue was purified by flash column chromatography (0–10% EtOAc/hexanes) to afford the title compound as a pale yellow oil (9.6 mg, 25 μmol, 18% yield, 6:1 dr cis:trans). The dr was determined based on the integration of the resonances attributed to the benzylic hydrogens in the ^1^H NMR spectrum. The relative configuration was assigned based on nOe analysis. For clarity, the ^1^H NMR and ^13^C NMR data of the major and minor diastereomers have been tabulated individually.

TLC R_f_ = 0.5 (10% EtOAc/hexanes); HRMS (TOF MS ES+) *m/z* [M + H] calcd. for C_23_H_26_NO_2_S_2_ 380.1684, found 380.1689.

**Major Diastereomer:**^1^H NMR (500 MHz, CDCl_3_) δ 7.80–7.76 (m, 4H), 7.73–7.71 (m, 1H), 7.63 (s, 1H), 7.46–7.44 (m, 3H), 7.28 (d, *J* = 8.0 Hz, 2H), 5.48 (d, *J* = 4.5 Hz, 1H), 3.96 (d, *J* = 14.4 Hz, 1H), 3.06 (ddd, *J* = 14.0, 13.2, 3.1 Hz, 1H), 2.69 (d, *J* = 25.9 Hz, 1H), 2.42 (s, 3H), 2.30 (d, *J* = 13.3 Hz, 1H), 1.43–1.36 (m, 2H), 0.98 (ddd, *J* = 24.5, 12.4, 4.5 Hz, 1H), 0.82 (d, *J* = 6.5 Hz, 3H); ^13^C NMR (151 MHz, CDCl_3_) δ 143.1, 138.8, 136.7, 133.3, 132.3, 129.7 (2C), 128.4, 128.0, 127.5, 127.1 (2C), 126.1, 125.9, 125.8, 125.1, 55.6, 42.0, 36.0, 33.0, 25.3, 22.2, 21.5.

**Minor Diastereomer:**^1^H NMR (500 MHz, CDCl_3_) δ 7.80–7.76 (m, 4H), 7.73–7.71 (m, 1H), 7.63 (s, 1H), 7.46–7.44 (m, 3H), 7.28 (d, *J* = 8.0 Hz, 2H), 5.25 (d, *J* = 4.9 Hz, 1H), 3.88 (d, *J* = 10.6 Hz, 1H), 3.04–2.98 (m, 1H), 2.42 (s, 3H), 2.38 (d, *J* = 3.86 Hz, 1H), 2.14 (d, *J* = 13.7 Hz, 1H), 1.43–1.36 (m, 2H), 0.79 (d, *J* = 6.5 Hz, 3H), 0.75 (d, *J* = 6.4 Hz, 1H); ^13^C NMR (151 MHz, CDCl_3_) δ 143.1, 138.8, 136.7, 133.3, 132.3, 129.6 (2C), 129.3, 128.0, 127.6, 127.1 (2C), 126.1, 125.9, 125.8, 124.0, 55.2, 41.8, 36.0, 33.1, 25.2, 23.3, 21.5.

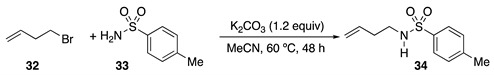



***N-(but-3-en-1-yl)-4-methylbenzenesulfonamide* (34)** was prepared according to a procedure reported by Jiang [77]. To a flame-dried flask equipped with a stir bar was added 4-bromo-1-butene **32** (4.1 mL, 40. mmol, 1.0 equiv), *p*-toluenesulfonamide **33** (6.8 g, 40. mmol, 1.0 equiv), K_2_CO_3_ (6.6 g, 48 mmol, 1.2 equiv), and MeCN (160 mL). The mixture was heated to 60 °C and allowed to stir for 3 d. The reaction mixture was quenched with saturated NH_4_Cl (100 mL) and extracted with EtOAc (3 × 50 mL). The combined organic layers were washed with H_2_O (50 mL) and brine (50 mL), dried over Na_2_SO_4_, and concentrated in vacuo. The residue was purified by column chromatography (5–25% EtOAc/hexanes) to afford the title compound as a clear, colorless oil (5.4 g, 24 mmol, 60%). Analytical data are consistent with literature values [77]. ^1^H NMR: (400 MHz, CDCl_3_) δ 7.76 (d, *J* = 8.2, 2H), 7.30 (d, *J* = 8.1, 2H), 5.63 (ddt, *J* = 17.1, 10.4, 6.8, 1H), 5.11 (br s, 1H), 5.02–4.93 (m, 2H), 2.99 (q, *J* = 6.7, 2H), 2.41 (s, 3H), 2.20 (q, *J* = 6.9, 2 H).

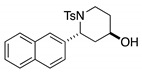



***2-(Naphthalen-2-yl)-1-tosylpiperidin-4-ol* (35)** was prepared according to Method F. The following amounts of reagents were used: 2-napthaldehyde (0.94 g, 6.0 mmol, 1.0 equiv), homoallylic sulfonamide **35** (1.1 mL, 6.0 mmol, 1.0 equiv), TFA (4.6 mL, 60. mmol, 10 equiv), and CH_2_Cl_2_ (60 mL, 0.10 M). The residue was purified by flash column chromatography (0–30% EtOAc/hexanes) to afford the title compound as an orange solid (0.72 g, 1.8 mmol, 31% yield, 5:1 dr trans:cis). The dr was determined based on the integration of the resonances attributed to the benzylic hydrogens in the ^1^H NMR spectrum. The relative configuration of the major **34** was assigned based on analogy to compound **24**. For clarity, the ^1^H NMR data of the major and minor diastereomers have been tabulated individually.

TLC R_f_ = 0.1 (30% EtOAc/hexanes, stained with CAM); HRMS (TOF MS ES+) *m/z* [M + H] calcd. for C_22_H_24_NO_3_S 382.1477, found 382.1483.

**Major Diastereomer:**^1^H NMR (500 MHz, CDCl_3_) δ 7.79–7.77 (m, 4H), 7.68 (s, 1H), 7.49–7.41 (m, 4H), 7.29 (d, *J* = 8.1 Hz, 2H), 5.54 (d, *J* = 4.5 Hz, 1H), 3.99 (d, *J* = 15.0 Hz, 1H), 3.74 (tt, *J* = 10.9, 7.9 Hz, 1H), 3.03 (td, *J* = 15.3, 2.7 Hz, 1H), 2.63 (dt, *J* = 13.3, 2.0 Hz 1H), 2.43 (s, 3H), 1.70 (br s, 1H), 1.58 (ddd *J* = 13.6, 11.3, 5.5 Hz, 1H), 1.26–1.18 (m, 2H). ^13^C NMR (151 MHz, CDCl_3_) δ 143.5, 138.3, 135.9, 133.3, 132.5, 130.0 (2C), 128.7, 128.0, 127.5, 127.0 (2C), 126.3, 126.2, 125.5, 124.7, 64.7, 55.8, 40.7, 36.2, 33.8, 21.6.

**Minor Diastereomer:**^1^H NMR (500 MHz, CDCl_3_) δ 7.74–7.70 (m, 4H), 7.63 (s, 1H), 7.60 (d, *J* = 8.3 Hz, 2H), 7.49–7.41 (m, 2H), 7.16 (d, *J* = 8.4 Hz, 2H), 5.10 (t, *J* = 5.1 Hz, 1H), 3.99 (d, *J* = 15.0 Hz, 1H), 3.67 (tt, *J* = 13.4, 4.6 Hz, 1H), 3.03 (td, *J* = 15.3, 2.7 Hz, 1H), 2.63 (dt, *J* = 13.3, 2.0 Hz 1H), 2.36 (s, 3H), 1.81–1.73 (m, 1H), 1.67 (br s, 1H), 1.26–1.18 (m, 2H). ^13^C NMR (151 MHz, CDCl_3_) δ 143.3, 137.7, 135.9, 133.2, 132.5, 129.6 (2C), 128.7, 128.2, 127.5, 127.2 (2C), 126.3, 126.0, 125.3, 124.8, 65.1, 55.5, 39.0, 37.0, 31.9, 21.5.

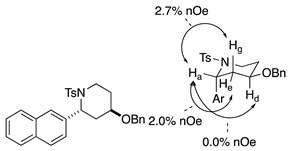



***4-(Benzyloxy)-2-(naphthalen-2-yl)-1-tosylpiperidine* (26)** was prepared according to Method G. The following amounts of reagents were used: alcohol **35** (0.25 g, 0.66 mmol, 1.0 equiv), NaH (63 mg, 2.6 mmol. 4.0 equiv), benzyl bromide (90. μL, 0.73 mmol, 1.1 equiv), and THF (2.3 mL, 0.2 M). The residue was purified by column chromatography (0–10% EtOAc/hexanes) to afford the title compound as a white solid (140 mg, 0.30 mmol, 56% yield, 5:1 dr trans:cis). The dr was determined based on the integration of the resonances attributed to the benzylic hydrogens in the ^1^H NMR spectrum. The relative configuration was assigned based on nOe analysis. For clarity, the ^1^H NMR and ^13^C NMR data of the major and minor diastereomers have been tabulated individually.

TLC R_f_ = 0.8 (20% EtOAc/hexanes, stained with CAM); HRMS (TOF MS ES+) *m/z* [M + Na] calcd. for C_29_H_29_NO_3_SNa 494.1766, found 494.1758.

**Major Diastereomer:**^1^H NMR (500 MHz, CDCl_3_) δ 7.80 (d, *J* = 8.1 Hz, 4H), 7.70 (d, 1H), 7.61 (s, 1H), 7.48–7.45 (m, 3H), 7.32–7.26 (m, 7H), 5.55 (d, *J* = 3.8 Hz, 1H), 4.50 (d, *J* = 11.9 Hz, 1H), 4.43 (d, *J* = 11.9 Hz, 1H), 4.02 (d, *J* = 14.7 Hz, 1H), 3.52 (tt, *J* = 10.8 Hz, 1H), 3.04 (td, *J* = 14.5, 2.5 Hz, 1H), 2.68 (d, *J* = 13.6 Hz, 1H), 2.43 (s, 3H), 1.80 (d, *J* = 11.5 Hz, 1H), 1.62 (ddd, *J* = 17.7, 11.9, 6.1 Hz, 1H), 1.31–1.34 (m, 1H); ^13^C NMR (151 MHz, CDCl_3_) δ 143.4, 138.3, 136.0, 133.2, 132.5, 130.0 (2C), 129.5, 128.6, 128.5 (2C), 128.0, 127.8, 127.7 (2C), 127.5, 127.0 (2C), 126.2, 126.1, 125.5, 124.8, 71.3, 70.2, 55.8, 40.8, 33.4, 30.8, 21.6.

**Minor Diastereomer:**^1^H NMR (500 MHz, CDCl_3_) δ 7.80 (d, *J* = 8.1 Hz, 4H), 7.70 (d, 1H), 7.59 (s, 1H), 7.48–7.45 (m, 3H), 7.32–7.26 (m, 2H), 7.15 (d, *J* = 8.2 Hz, 1H), 7.07 (t, *J* = 7.6 Hz, 1H), 6.96 (t, *J* = 7.3 Hz, 2H), 6.69 (d, *J* = 7.6 Hz, 1H), 5.17 (t, *J* = 5.1 Hz, 1H), 4.24, 4.20 (ABq, *J*_AB_ = 12.3 Hz, 2H), 3.80–3.68 (m, 3H), 2.53 (dt, *J* = 14.4, 9.4 Hz, 1H), 2.35 (s, 3H), 2.06 (ddd, *J* = 14.3, 5.3, 2.9 Hz, 1H), 1.81–1.80 (m, 2H), 1.31–1.34 (m, 1H); ^13^C NMR (151 MHz, CDCl_3_) δ 143.4, 138.4, 136.0, 133.2, 132.5, 130.0 (2C), 129.5, 128.6, 128.5 (2C), 128.0, 127.8, 127.7 (2C), 127.5, 127.19 (2C), 127.16, 125.9, 125.6, 125.2, 71.2, 69.8, 55.8, 39.3, 34.2, 30.8, 21.6.

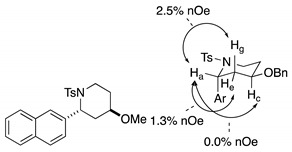



***4-Methoxy-2-(naphthalen-2-yl)-1-tosylpiperidine* (12)** was prepared according to Method G. The following amounts of reagents were used: alcohol **35** (110 mg, 0.30 mmol, 1.0 equiv), NaH (16 mg, 0.67 mmol, 2.2 equiv), methyl iodide (20. μL, 0.33 mmol, 1.1 equiv), and THF (1.5 mL, 0.20 M). The residue was purified by column chromatography (0–20% EtOAc/hexanes) to afford the title compound as a pale yellow solid (61 mg, 0.15 mmol, 52% yield, 5:1 dr trans:cis). The dr was determined based on the integration of the resonances attributed to the benzylic hydrogens in the ^1^H NMR spectrum. The relative configuration was assigned based on nOe analysis. For clarity, the ^1^H NMR and ^13^C NMR data of the major and minor diastereomers have been tabulated individually.

TLC R_f_ = 0.6 (30% EtOAc/hexanes, stained with CAM); HRMS (TOF MS ES+) *m/z* [M + Na] calcd. for C_23_H_25_NO_3_SNa 396.1633, found 396.1636.

**Major Diastereomer:**^1^H NMR (500 MHz, CDCl_3_) δ 7.83–7.76 (m, 5H), 7.72 (s, 1H), 7.54–7.51 (m, 1H), 7.48–7.46 (m, 2H), 7.29 (d, *J* = 7.9 Hz, 2H), 5.56 (s, 1H), 4.04 (d, *J* = 14.1 Hz, 1H), 3.29 (tt, *J* = 7.4, 3.0 Hz, 1H), 3.25 (s, 3H), 3.09 (t, *J* = 13.1 Hz, 1H), 2.68 (d, *J* = 13.2 Hz, 1H), 2.42 (s, 3H), 1.80 (d, *J* = 11.5 Hz, 1H), 1.53 (td, *J* = 12.2 Hz, 1H), 1.16 (qd, *J* = 11.6, 5.8 Hz, 1H); ^13^C NMR (151 MHz, CDCl_3_) δ 143.4, 138.4, 136.1, 133.4, 132.5, 130.0 (2C), 126.7, 128.1, 127.6, 127.0 (2C), 126.3, 126.1, 125.5, 124.7, 73.2, 55.8, 55.5, 40.8, 33.2, 30.2, 21.6.

**Minor Diastereomer:**^1^H NMR (500 MHz, CDCl_3_) δ 7.83–7.76 (m, 5H), 7.72 (s, 1H), 7.54–7.51 (m, 1H), 7.48–7.46 (m, 2H), 7.29 (d, *J* = 7.9 Hz, 2H), 4.99 (t, *J* = 5.38 Hz, 1H), 3.77–3.71 (m, 1H), 3.60 (dt, *J* = 13.6, 4.6 Hz, 1H), 3.42 (br s, 1H), 3.03 (s, 3H), 2.37–2.29 (m, 1H), 2.33 (s, 3H), 2.02 (d, *J* = 15.2 Hz, 1H), 1.86–1.82 (m, 1H), 1.72–1.68 (m, 1H); ^13^C NMR (151 MHz, CDCl_3_) δ 143.0, 138.3, 137.4, 133.1, 132.5, 129.4 (2C), 126.7, 128.0, 127.5, 127.2 (2C), 125.8, 125.7, 125.5, 125.2, 73.5, 56.4, 55.5, 40.0, 34.1, 29.6, 21.5.

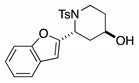



***2-(Naphthalen-2-yl)-1-tosylpiperidin-4-ol* (36)** was prepared according to Method F. The following amounts of reagents were used: 2-benzofurancarboxaldehyde (0.60 mL, 5.0 mmol, 1.0 equiv), homoallylic sulfonamide **34** (0.91 mL, 5.0 mmol, 1.0 equiv), TFA (3.8 mL, 50. mmol, 10 equiv), CH_2_Cl_2_ (50 mL, 0.10 M). The residue was purified by flash column chromatography (0–50% EtOAc/hexanes) to afford the title compound as an orange solid (0.42 g, 1.1 mmol, 22% yield, 5:1 dr trans:cis). The dr was determined based on the integration of the resonances attributed to the benzylic hydrogens in the ^1^H NMR spectrum. The relative configuration was assigned based on analogy to compound **26**. For clarity, the ^1^H NMR data of the major and minor diastereomers have been tabulated individually.

TLC R_f_ = 0.1 (30% EtOAc/hexanes, stained with CAM).

**Major Diastereomer:**^1^H NMR (500 MHz, CDCl_3_) δ 7.65 (d, *J* = 8.29 Hz, 2H), 7.46 (dd, *J* = 6.75, 2.12 Hz, 1H), 7.21–7.18 (m, 3H), 7.15 (d, *J* = 8.61 Hz, 2H), 6.49 (t, *J* = 2.0 Hz, 1H), 5.51 (d, *J* = 5.48 Hz, 1H), 3.97–3.90 (m, 2H), 3.23 (td, *J* = 13.5, 2.7 Hz, 1H), 2.51–2.45 (m, 1H), 2.33 (s, 3H), 1.94–1.88 (m, 1H), 1.75 (ddd, *J* = 13.0, 11.6, 5.9 Hz, 1H), 1.53 (d, *J* = 5.0 Hz, 1H), 1.44 (ddd, *J* = 24.1, 12.8, 4.5 Hz, 1H).

**Minor Diastereomer:**^1^H NMR (500 MHz, CDCl_3_) δ 7.65 (d, *J* = 8.29 Hz, 2H), 7.46 (dd, *J* = 6.75, 2.12 Hz, 1H), 7.21–7.18 (m, 3H), 7.15 (d, *J* = 8.61 Hz, 2H), 6.51 (t, *J* = 1.1 Hz, 1H), 5.33 (d, *J* = 5.6 Hz, 1H), 4.14–4.10 (m, 2H), 3.64 (td, *J* = 10.9, 4.0 Hz, 1H), 2.51–2.45 (m, 1H), 2.32 (s, 3H), 2.12 (ddd, *J* = 14.4, 6.7, 3.3 Hz, 1H), 1.94–1.88 (m, 1H), 1.53 (d, *J* = 5.0 Hz, 1H), 1.44 (ddd, *J* = 24.1, 12.8, 4.5 Hz, 1H).

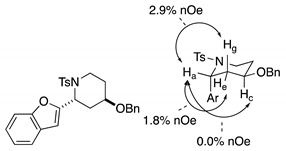



***2-(benzofuran-2-yl)-4-(benzyloxy)-1-tosylpiperidine* (27)** was prepared according to method G. The following amounts of reagents were used: alcohol **36** (0.15 g, 0.40 mmol, 1.0 equiv), NaH (46 mg, 1.9 mmol, 4.7 equiv), benzyl bromide (52 μL, 0.44 mmol, 1.1 equiv), and THF (3.0 mL, 0.2 M). The residue was purified by column chromatography (0–10% EtOAc/hexanes) to afford the title compound as a yellow solid (87 mg, 0.19 mmol, 47% yield, 5:1 dr trans:cis). The dr was determined based on the integration of the resonances attributed to the benzylic hydrogens in the ^1^H NMR spectrum. The relative configuration was assigned based on nOe analysis. For clarity, the ^1^H NMR and ^13^C NMR data of the major and minor diastereomers have been tabulated individually.

TLC R_f_ = 0.8 (30% EtOAc/hexanes, stained with CAM); HRMS (TOF MS ES+) *m/z* [M + Na] calcd. for C_27_H_27_NO_4_SNa 484.1559, found 484.1542.

**Major Diastereomer:**^1^H NMR (500 MHz, CDCl_3_) δ 7.65 (d, *J* = 8.3 Hz, 2H), 7.45, (d, *J* = 8.3 Hz, 1H), 7.30–7.17 (m, 8H), 7.15 (d, *J* = 8.2 Hz, 2H), 6.44 (s, 1H), 5.52 (d, *J* = 5.3 Hz, 1H), 4.49 (s, 2H), 3.94 (d, *J* = 13.7 Hz, 1H), 3.66 (tt, *J* = 11.2, 4.0 Hz, 1H), 3.20 (td, *J* = 13.4, 2.6 Hz, 1H), 2.57 (dt, *J* = 13.2, 1.8 Hz, 1H), 2.32 (s, 3H), 1.97 (d, *J* = 12.3 Hz, 1H), 1.78 (ddd, *J* = 13.0, 11.7, 5.8 Hz, 1H), 1.45 (qd, *J* = 12.8, 4.8 Hz, 1H); ^13^C NMR (151 MHz, CDCl_3_) δ 155.3, 154.7, 143.3, 138.2, 137.2, 129.5 (2C), 128.5 (2C), 128.1, 128.0, 127.8, 127.1, 126.9, 124.1, 122.9, 120.9, 111.1, 104.8, 71.8, 70.2, 51.7, 41.4, 34.2, 31.3, 21.5.

**Minor Diastereomer:**^1^H NMR (500 MHz, CDCl_3_) δ 7.68 (d, *J* = 8.3 Hz, 2H), 7.35, (d, *J* = 4.4 Hz, 1H), 7.30–7.17 (m, 5H), 7.07 (t, *J* = 7.5 Hz, 1H), 6.99 (t, *J* = 7.6, 2H), 6.79 (d, *J* = 7.6 Hz, 2H), 6.42 (s, 1H), 5.36 (d, *J* = 6.4 Hz, 1H), 4.28 (s, 2H), 3.76–3.73 (m, 2H), 3.66 (tt, *J* = 11.2, 4.0 Hz, 1H), 2.70 (d, *J* = 14.1 Hz, 1H), 2.32 (s, 3H), 1.97 (d, *J* = 12.3 Hz, 1H), 1.83 (d, *J* = 13.8 Hz, 1H), 1.72–1.68 (m, 1H); ^13^C NMR (151 MHz, CDCl_3_) δ 155.3, 154.7, 143.3, 138.2, 137.2, 129.5 (2C), 128.5 (2C), 128.1, 128.0, 127.8, 127.1, 126.9, 123.6, 122.6, 120.7, 110.9, 103.2, 71.8, 70.0, 49.6, 37.6, 31.0, 29.3, 15.3.

## 4. Conclusions

In conclusion, we have developed a Kumada XC reaction of benzylic sulfonamides with Grignard reagents including methylmagnesium iodide and arylmagnesium iodide. This reaction utilizes readily available starting materials that are not activated prior to the XC reaction. We have demonstrated that increasing the steric bulk adjacent to the reactive center destabilizes the conformation necessary for β-hydride elimination to occur. A stereospecific ring opening Kumada XC reaction has been established to synthesize highly substituted acyclic fragments. This work provides a basis for the XC reaction of simple benzylic sulfonamides.

## Figures and Tables

**Table 1:**
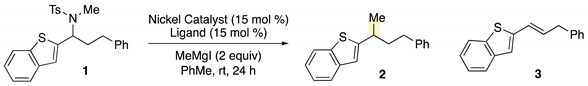
Optimization of Kumada XC Reaction of Benzylic Sulfonamides.

Entry	Nickel Catalyst	Ligand	Yield 2 (%) ^1^	Yield 3 (%) ^1^	RSM 1 (%) ^1^
1 ^2^	Ni(cod)_2_	*rac*-BINAP	25	10	19
2	Ni(cod)_2_	*rac*-BINAP	34	30	7
3	Ni(cod)_2_	DPEPhos	42	20	0
4	Ni(cod)_2_	XantPhos	0	<5	37
5	Ni(cod)_2_	dppe	0	<5	65
6	Ni(cod)_2_	SiMes·BF_4_	12	0	86
7	Ni(cod)_2_	BPhen	0	0	61
**8**	**(*R*-BINAP)NiCl_2_**	**–**	**54**	**40**	**0**

^1^ Yield of **2** and **3** and Recovered Starting Material (RSM) determined by ^1^H NMR based on comparison to PhTMS as internal standard. ^2^ 5 mol % Ni(cod)_2_.

## Data Availability

Data sharing not applicable.

## Supplementary Materials

The following are available online, ^1^H, ^13^C, COSY and NOE NMR data are available online.

## Sample Availability

Samples of the compounds are not available from the authors.
