# Supplementary material for: Nickel-Catalyzed Kumada Cross-Coupling Reactions of Benzylic Sulfonamides
Source: Molecules. 2021 Sep 30;26(19):5947. doi: 10.3390/molecules26195947 (PMC8512530; doi:10.3390/molecules26195947)
Supplement: Supplementary file 1 [file molecules-26-05947-s001.zip › molecules-1385874-supplementary.pdf]

1H spectrum

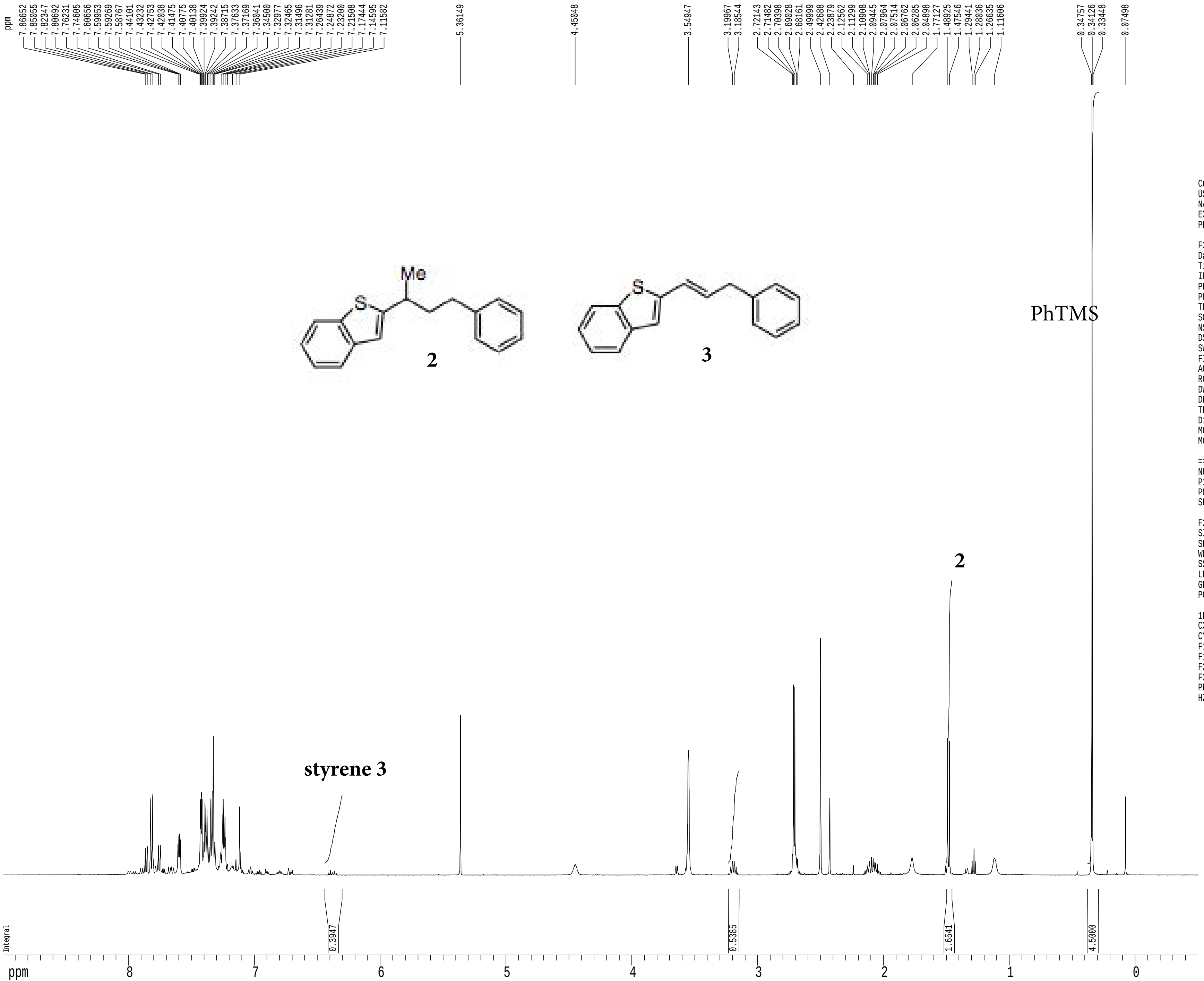

Current Data Parameters
USER khewitt1
NAME KAH-I-186
EXPNO 2
PROCNO 1

F2 - Acquisition Parameters
Date_ 20181105
Time 14.50
INSTRUM cryo500
PROBHD 5 mm CPTCI 1H-
PULPROG zg30
TD 48074
SOLVENT CDCl3T
NS 8
DS 2
SWH 8012.820 Hz
FIDRES 0.166677 Hz
AQ 2.9998677 sec
RG 7.1
DW 62.400 usec
DE 6.00 usec
TE 298.0 K
D1 0.10000000 sec
MCREST 0.00000000 sec
MCWRK 0.01500000 sec

======== CHANNEL f1 ========
NUC1 1H
P1 7.50 usec
PL1 1.60 dB
SFO1 500.2235015 MHz

F2 - Processing parameters
SI 65536
SF 500.2200000 MHz
WDW no
SSB 0
LB 0.00 Hz
GB 0
PC 1.00

1D NMR plot parameters
CX 22.80 cm
CY 15.00 cm
F1P 9.000 ppm
F1 4501.98 Hz
F2P -0.500 ppm
F2 -250.11 Hz
PPMCM 0.41667 ppm/cm
HZCM 208.42500 Hz/cm

1H spectrum

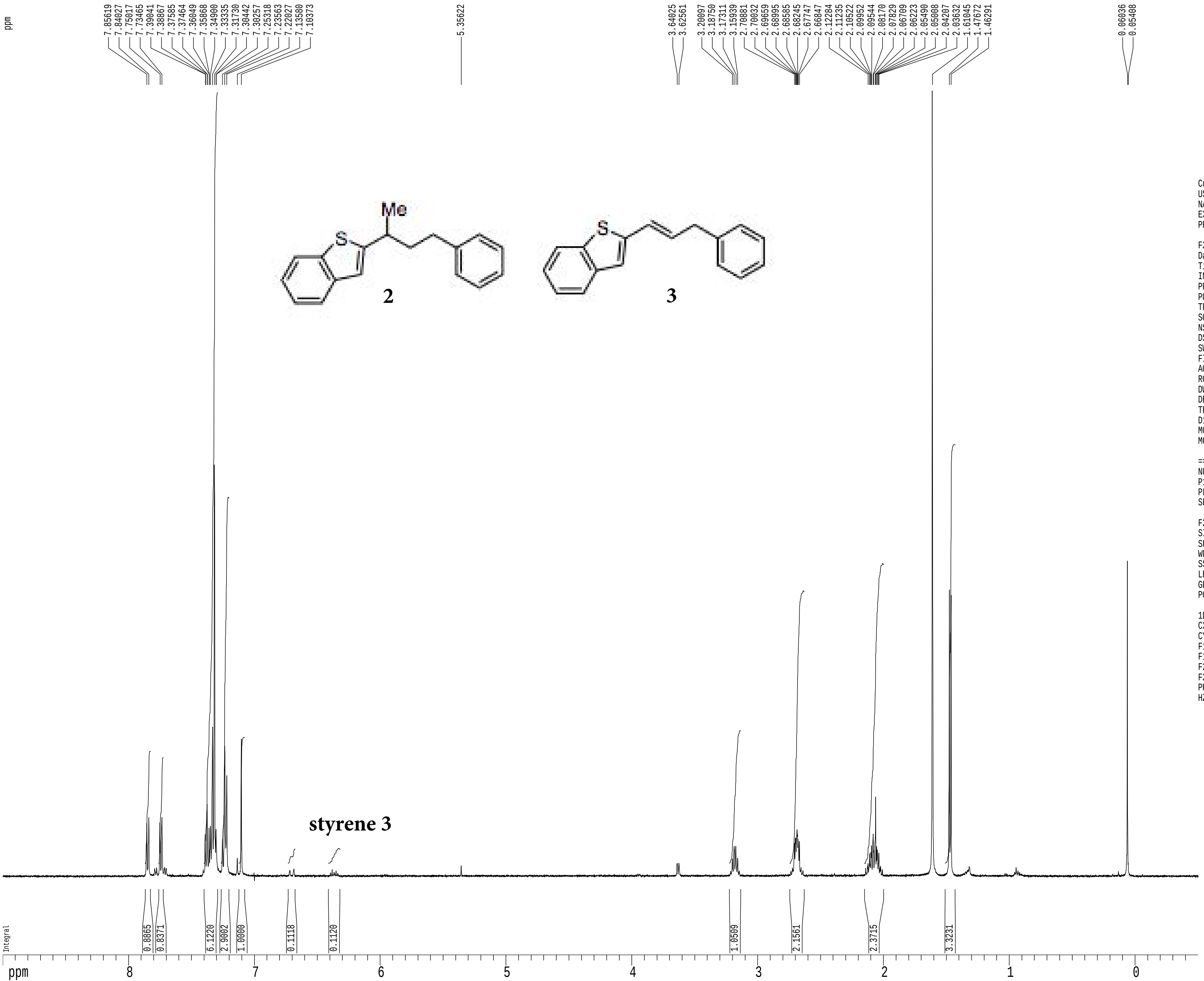

Current Data Parameters
USER khewitt1
NAME KAH-I-200-Z
EXPNO 4
PROCNO 1

F2 - Acquisition Parameters
Date_ 20210909
Time 16.37
INSTRUM gn500
PROBHD 5 mm broadband
PULPROG zg30
TD 48074
SOLVENT CDCl3T
NS 8
DS 2
SWH 8012.820 Hz
FIDRES 0.166677 Hz
AQ 2.9998677 sec
RG 1824.6
DW 62.400 usec
DE 6.00 usec
TE 298.0 K
D1 0.10000000 sec
MCREST 0.00000000 sec
MCWRK 0.01500000 sec

======== CHANNEL f1 ========
NUC1 1H
P1 12.00 usec
PL1 -6.00 dB
SFO1 498.6534906 MHz

F2 - Processing parameters
SI 65536
SF 498.6500000 MHz
WDW no
SSB 0
LB 0.00 Hz
GB 0
PC 1.00

1D NMR plot parameters
CX 22.80 cm
CY 15.00 cm
F1P 9.000 ppm
F1 4487.85 Hz
F2P -0.500 ppm
F2 -249.32 Hz
PPMCM 0.41667 ppm/cm
HZCM 207.77084 Hz/cm

Sample Calculation

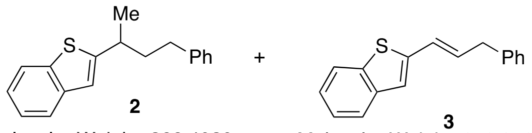

2 Molecular Weight: 266.4020   **3** Molecular Weight: 250.3590

Based on the NMR above: the ratio of compounds **3**/**2**:
integration of 2 = 1.05
integration of 3 = 0.11

$$r_{3/2} = \frac{0.11}{1.05} = 0.0948$$

14 mg total was isolated from column chromatography
theoretical yield = 0.20 mmol

$$\text{mmol of } \mathbf{2} = \frac{14}{266.40 + (250.36 \cdot 0.0948)} = 0.0483$$

$$\text{Yield of } \mathbf{2} = \frac{0.0483}{0.20} = 24\%$$

$$\text{Yield of } \mathbf{3} = \frac{0.0483 \cdot 0.0948}{0.20} = 2.2\%$$

Z-restored spin-echo 13C spectrum with 1H decoupling

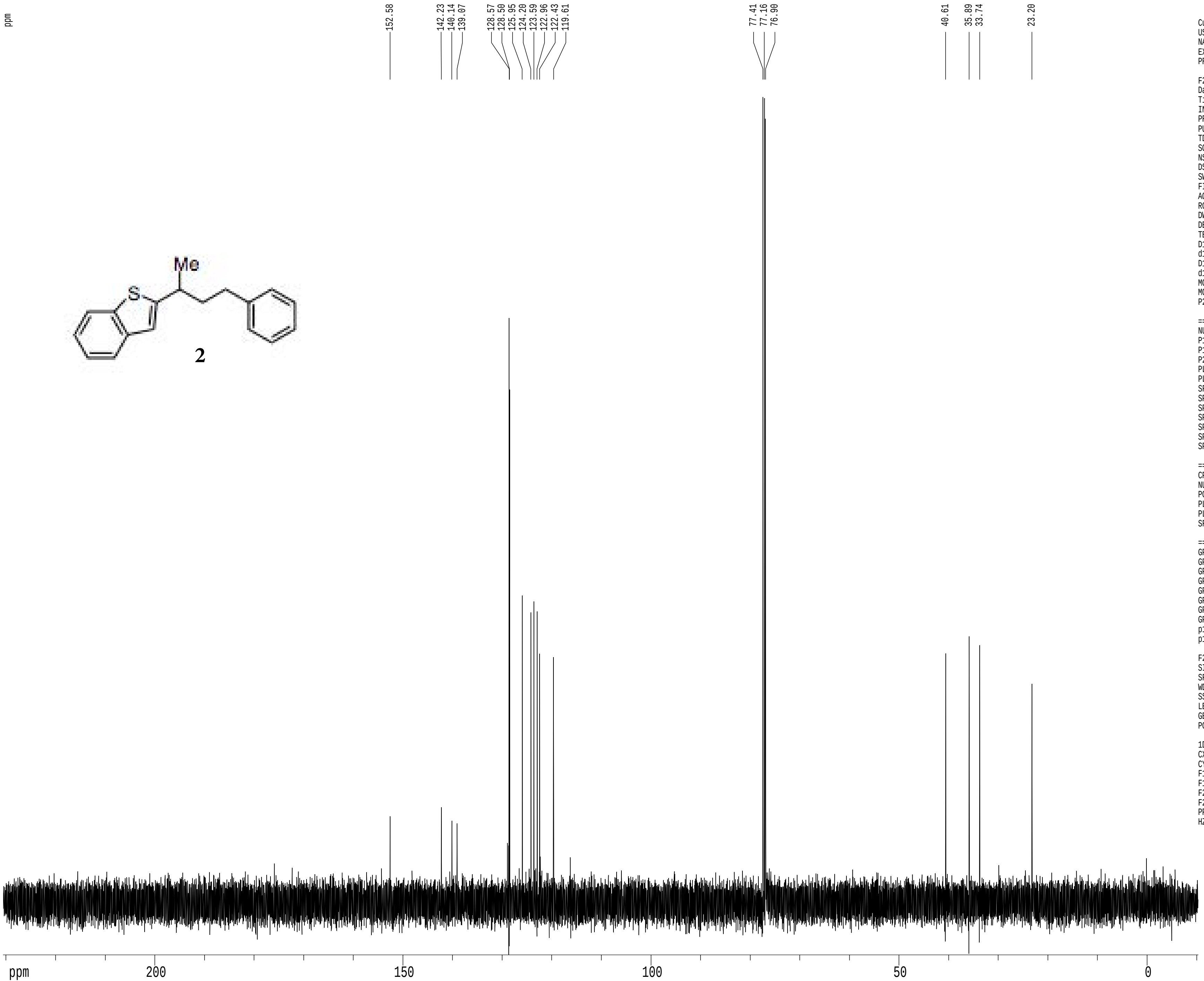

Current Data Parameters
USER khewitt1
NAME KAH-I-200-Z
EXPNO 2
PROCNO 1

F2 - Acquisition Parameters
Date_ 20181118
Time 15.00
INSTRUM cryo500
PROBHD 5 mm CPTCI 1H-
PULPROG SpinEchopg30gp2.prd
TD 65536
SOLVENT CDCl3
NS 120
DS 16
SWH 30303.031 Hz
FIDRES 0.462388 Hz
AQ 1.0813940 sec
RG 7298.2
DW 16.500 usec
DE 6.00 usec
TE 298.0 K
D1 0.25000000 sec
d11 0.03000000 sec
D16 0.00020000 sec
d17 0.00019600 sec
MCREST 0.00000000 sec
MCWRK 0.01500000 sec
P2 33.10 usec

======== CHANNEL f1 ========
NUC1 13C
P1 16.55 usec
P12 2000.00 usec
P20 500.00 usec
PL0 120.00 dB
PL1 -1.00 dB
SFO1 125.7942548 MHz
SP2 2.70 dB
SP4 2.70 dB
SPNAM2 Crp60comp.4
SPNAM4 Crp60,0.5,20.1
SPOFF2 0.00 Hz
SPOFF4 0.00 Hz

======== CHANNEL f2 ========
CPDPRG2 waltz16
NUC2 1H
PCPD2 100.00 usec
PL2 1.60 dB
PL12 23.54 dB
SFO2 500.2225011 MHz

====== GRADIENT CHANNEL =====
GPNAM1 SINE.100
GPNAM2 SINE.100
GPX1 0.00 %
GPX2 0.00 %
GPY1 0.00 %
GPY2 0.00 %
GPZ1 30.00 %
GPZ2 50.00 %
p15 500.00 usec
p16 1000.00 usec

F2 - Processing parameters
SI 65536
SF 125.7804071 MHz
WDW no
SSB 0
LB 0.00 Hz
GB 0
PC 2.00

1D NMR plot parameters
CX 22.80 cm
CY 15.65 cm
F1P 230.637 ppm
F1 29009.68 Hz
F2P -10.287 ppm
F2 -1293.96 Hz
PPMCM 10.56688 ppm/cm
HZCM 1329.10693 Hz/cm

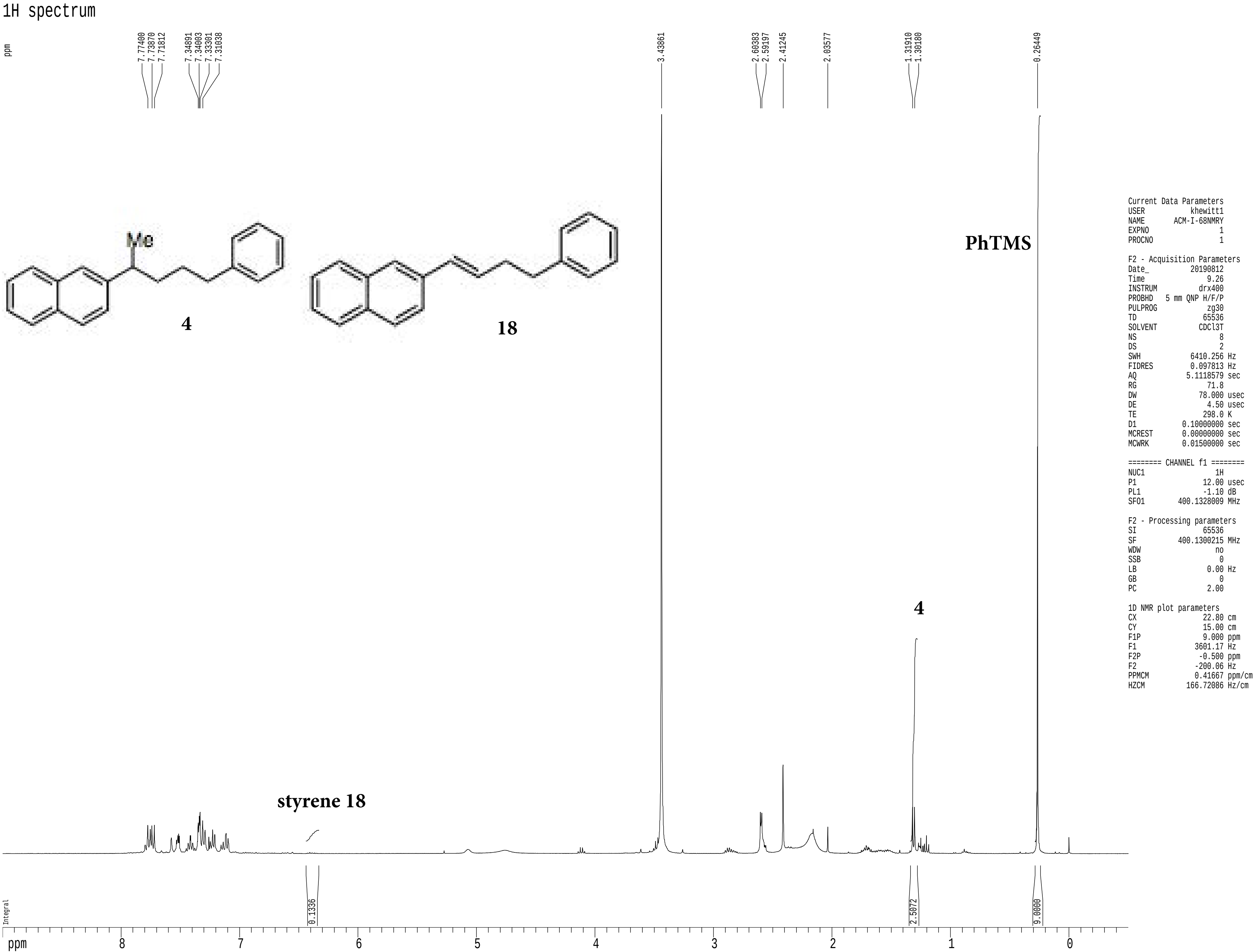
1H spectrum
ppm
7.77400
7.73870
7.71812
7.34891
7.34003
7.33301
7.31038
3.43861
2.60383
2.59197
2.41245
2.03577
1.31910
1.30180
0.26449
Me
4
18
PhTMS
4
styrene 18
Integral
0.1336
2.5072
9.0000
ppm 8 7 6 5 4 3 2 1 0
Current Data Parameters
USER khewitt1
NAME ACM-I-68NMRY
EXPNO 1
PROCNO 1
F2 - Acquisition Parameters
Date_ 20190812
Time 9.26
INSTRUM drx400
PROBHD 5 mm QNP H/F/P
PULPROG zg30
TD 65536
SOLVENT CDCl3T
NS 8
DS 2
SWH 6410.256 Hz
FIDRES 0.097813 Hz
AQ 5.1118579 sec
RG 71.8
DW 78.000 usec
DE 4.50 usec
TE 298.0 K
D1 0.10000000 sec
MCREST 0.00000000 sec
MCWRK 0.01500000 sec
======== CHANNEL f1 ========
NUC1 1H
P1 12.00 usec
PL1 -1.10 dB
SFO1 400.1328009 MHz
F2 - Processing parameters
SI 65536
SF 400.1300215 MHz
WDW no
SSB 0
LB 0.00 Hz
GB 0
PC 2.00
1D NMR plot parameters
CX 22.80 cm
CY 15.00 cm
F1P 9.000 ppm
F1 3601.17 Hz
F2P -0.500 ppm
F2 -200.06 Hz
PPMCM 0.41667 ppm/cm
HZCM 166.72086 Hz/cm

1H spectrum

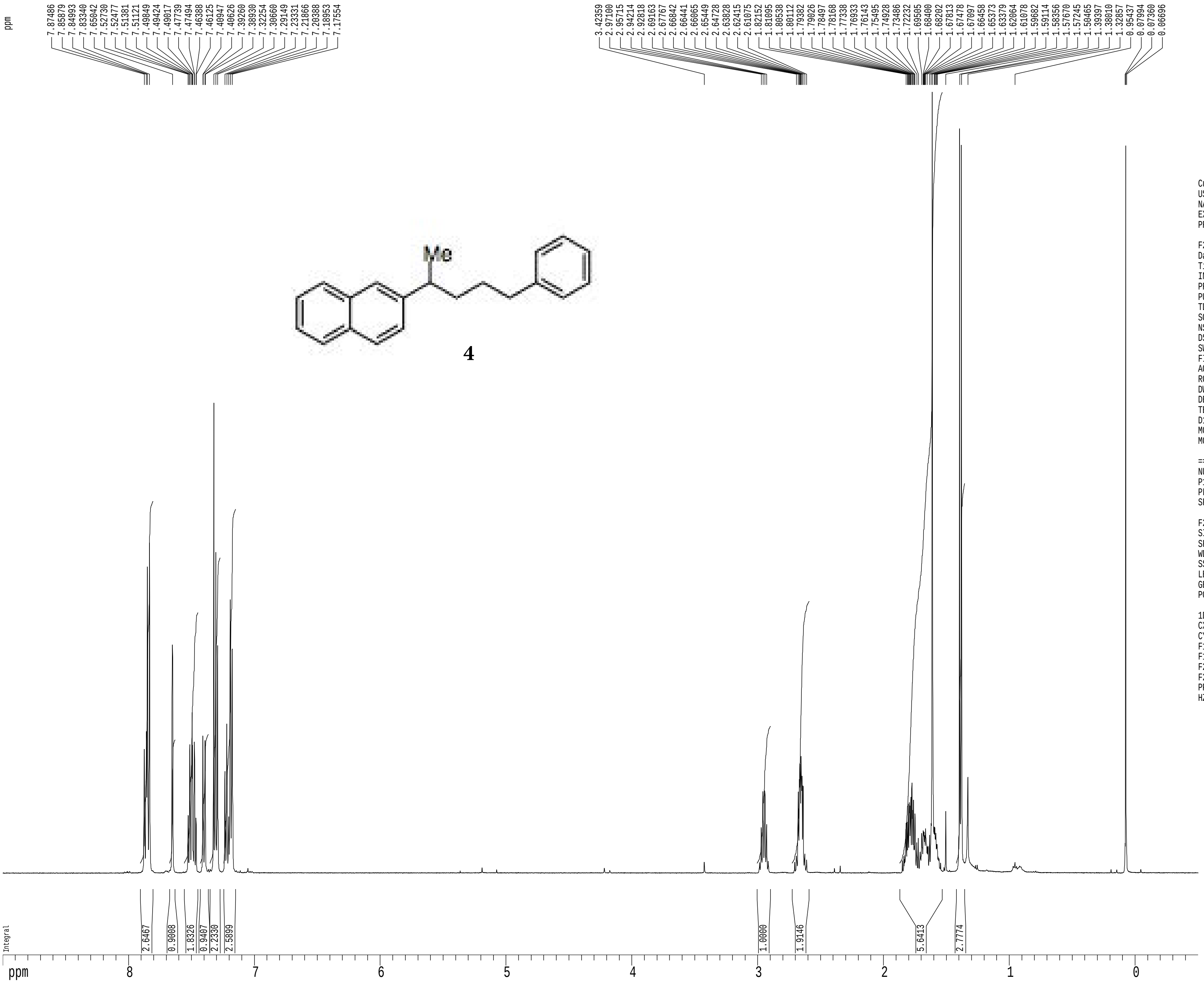

Current Data Parameters
USER khewitt1
NAME AM-I-123-Z
EXPNO 1
PROCNO 1

F2 - Acquisition Parameters
Date_ 20210809
Time 18.21
INSTRUM cryo500
PROBHD 5 mm CPTCI 1H-
PULPROG zg30
TD 48074
SOLVENT CDCl3T
NS 8
DS 2
SWH 8012.820 Hz
FIDRES 0.166677 Hz
AQ 2.9998677 sec
RG 8
DW 62.400 usec
DE 6.00 usec
TE 298.0 K
D1 0.10000000 sec
MCREST 0.00000000 sec
MCWRK 0.01500000 sec

======== CHANNEL f1 ========
NUC1 1H
P1 9.75 usec
PL1 1.60 dB
SFO1 500.2235015 MHz

F2 - Processing parameters
SI 65536
SF 500.2200000 MHz
WDW no
SSB 0
LB 0.00 Hz
GB 0
PC 1.00

1D NMR plot parameters
CX 22.80 cm
CY 15.00 cm
F1P 9.000 ppm
F1 4501.98 Hz
F2P -0.500 ppm
F2 -250.11 Hz
PPMCM 0.41667 ppm/cm
HZCM 208.42500 Hz/cm

Z-restored spin-echo 13C spectrum with 1H decoupling

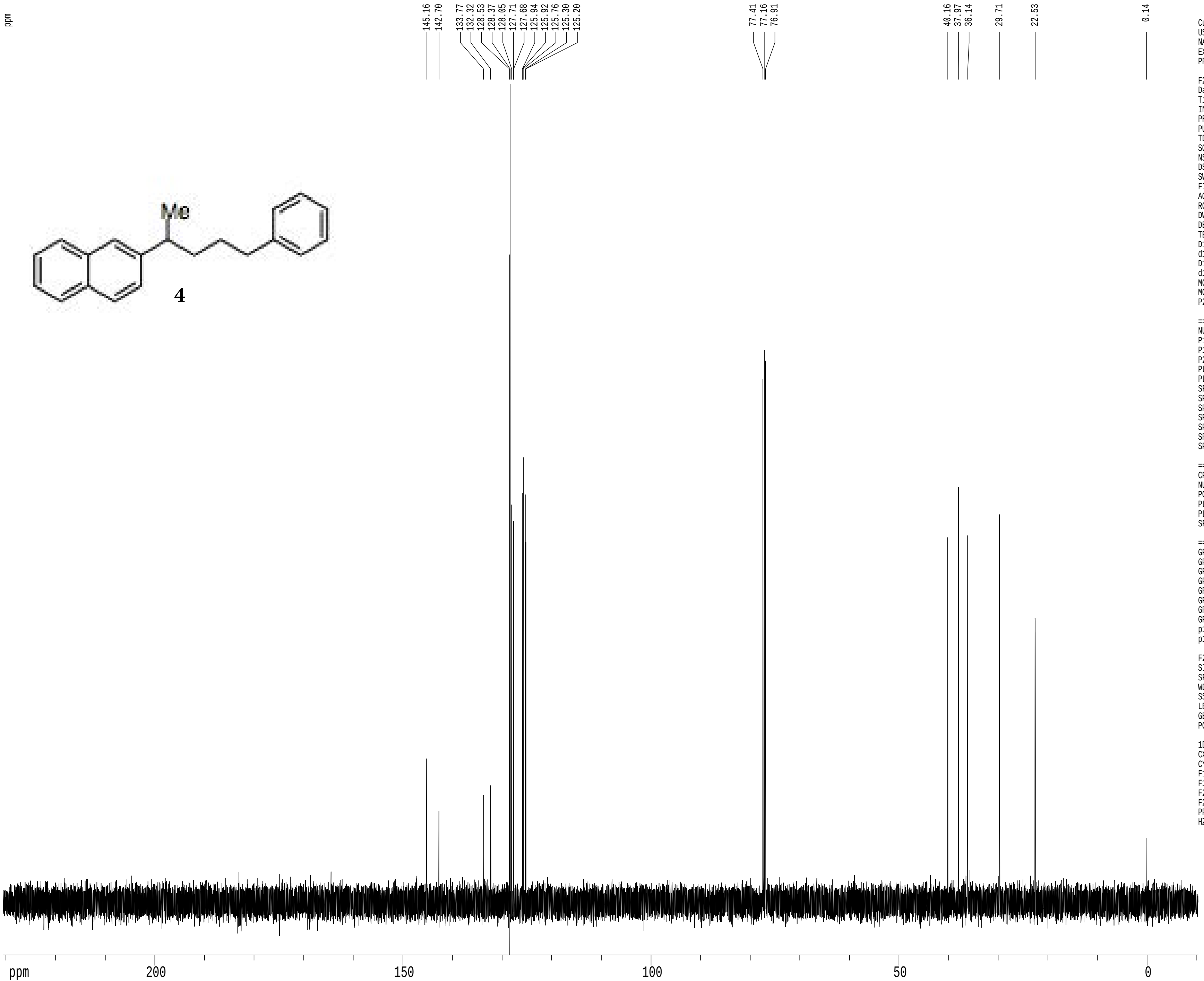

4


```
Current Data Parameters
USER          khewitt1
NAME          AM-I-123-Z
EXPNO                2
PROCNO               1

F2 - Acquisition Parameters
Date_         20210809
Time             18.23
INSTRUM        cryo500
PROBHD   5 mm CPTCI 1H-
PULPROG  SpinEchopg30gp2.prd
TD               65536
SOLVENT          CDCl3
NS                 152
DS                  16
SWH          30303.031 Hz
FIDRES        0.462388 Hz
AQ           1.0813940 sec
RG             14596.5
DW              16.500 usec
DE                6.00 usec
TE               298.0 K
D1          0.25000000 sec
d11         0.03000000 sec
D16         0.00020000 sec
d17         0.00019600 sec
MCREST      0.00000000 sec
MCWRK       0.01500000 sec
P2               37.70 usec

======== CHANNEL f1 ========
NUC1               13C
P1               18.85 usec
P12            2000.00 usec
P20             500.00 usec
PL0             120.00 dB
PL1              -1.00 dB
SFO1       125.7942548 MHz
SP2               1.55 dB
SP4               1.55 dB
SPNAM2     Crp60comp.4
SPNAM4   Crp60,0.5,20.1
SPOFF2            0.00 Hz
SPOFF4            0.00 Hz

======== CHANNEL f2 ========
CPDPRG2         waltz16
NUC2                 1H
PCPD2           100.00 usec
PL2               1.60 dB
PL12             22.00 dB
SFO2       500.2225011 MHz

====== GRADIENT CHANNEL =====
GPNAM1         SINE.100
GPNAM2         SINE.100
GPX1               0.00 %
GPX2               0.00 %
GPY1               0.00 %
GPY2               0.00 %
GPZ1              30.00 %
GPZ2              50.00 %
p15              500.00 usec
p16             1000.00 usec

F2 - Processing parameters
SI                65536
SF          125.7804071 MHz
WDW                  no
SSB                   0
LB                 0.00 Hz
GB                    0
PC                 2.00

1D NMR plot parameters
CX                22.80 cm
CY                15.65 cm
F1P             230.637 ppm
F1             29009.68 Hz
F2P             -10.287 ppm
F2             -1293.96 Hz
PPMCM          10.56688 ppm/cm
HZCM         1329.10693 Hz/cm
```

h

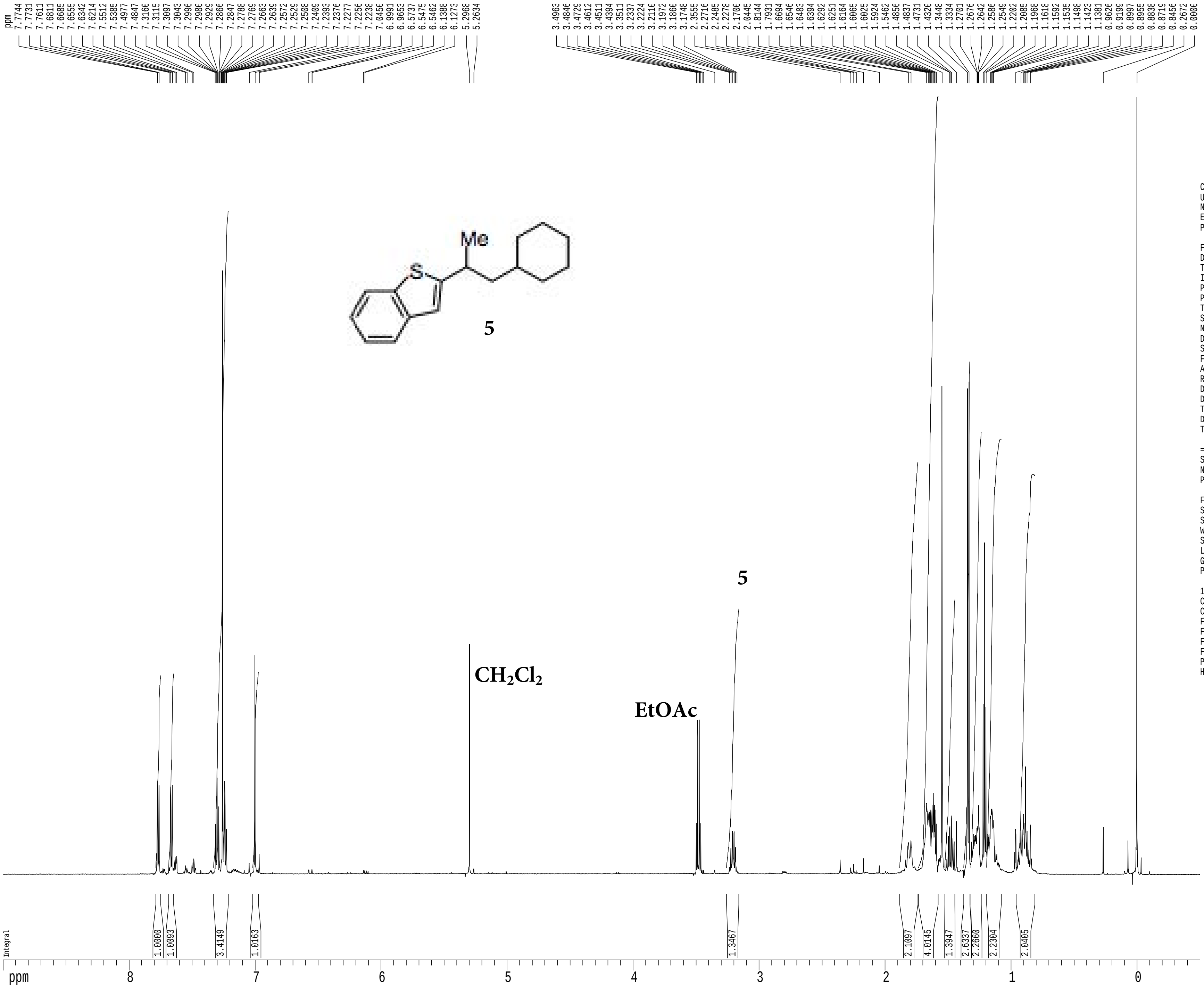

Current Data Parameters
USER khewitt1
NAME ACM-I-123-benzoCH
EXPNO 31
PROCNO 1

F2 - Acquisition Parameters
Date_ 20191115
Time 18.27
INSTRUM av600
PROBHD 5 mm CPBBO BB-
PULPROG zg30
TD 98074
SOLVENT CDCl3T
NS 8
DS 2
SWH 9615.385 Hz
FIDRES 0.098042 Hz
AQ 5.0998979 sec
RG 10
DW 52.000 usec
DE 13.70 usec
TE 298.0 K
D1 0.10000000 sec
TD0 1

======== CHANNEL f1 ========
SFO1 600.1342009 MHz
NUC1 1H
P1 12.00 usec

F2 - Processing parameters
SI 65536
SF 600.1300376 MHz
WDW EM
SSB 0
LB 0.30 Hz
GB 0
PC 1.00

1D NMR plot parameters
CX 22.80 cm
CY 15.00 cm
F1P 9.000 ppm
F1 5401.17 Hz
F2P -0.500 ppm
F2 -300.06 Hz
PPMCM 0.41667 ppm/cm
HZCM 250.05420 Hz/cm

13C spectrum with 1H decoupling

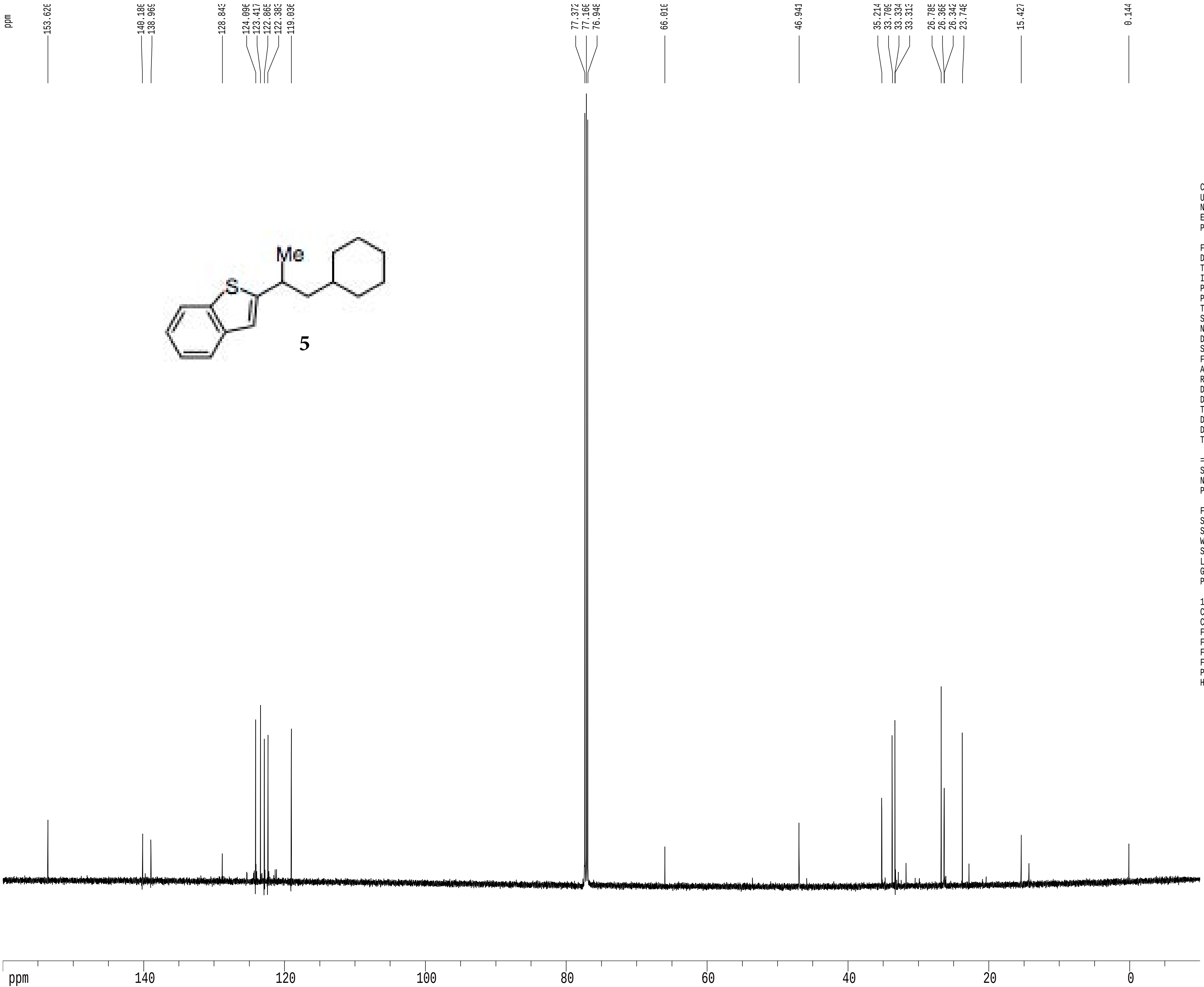

5

Current Data Parameters
USER khewitt1
NAME ACM-I-123-benzoCH
EXPNO 2
PROCNO 1

F2 - Acquisition Parameters
Date_ 20191115
Time 18.30
INSTRUM av600
PROBHD 5 mm CPBBO BB-
PULPROG zgdc30
TD 65536
SOLVENT CDCl3T
NS 447
DS 4
SWH 36231.883 Hz
FIDRES 0.552855 Hz
AQ 0.9044468 sec
RG 2050
DW 13.800 usec
DE 19.63 usec
TE 298.0 K
D1 0.40000001 sec
D11 0.03000000 sec
TD0 1

======== CHANNEL f1 ========
SFO1 150.9194080 MHz
NUC1 13C
P1 10.10 usec

F2 - Processing parameters
SI 65536
SF 150.9027936 MHz
WDW no
SSB 0
LB 0.00 Hz
GB 0
PC 1.00

1D NMR plot parameters
CX 22.80 cm
CY 15.00 cm
F1P 160.000 ppm
F1 24144.45 Hz
F2P -10.000 ppm
F2 -1509.03 Hz
PPMCM 7.45614 ppm/cm
HZCM 1125.15234 Hz/cm

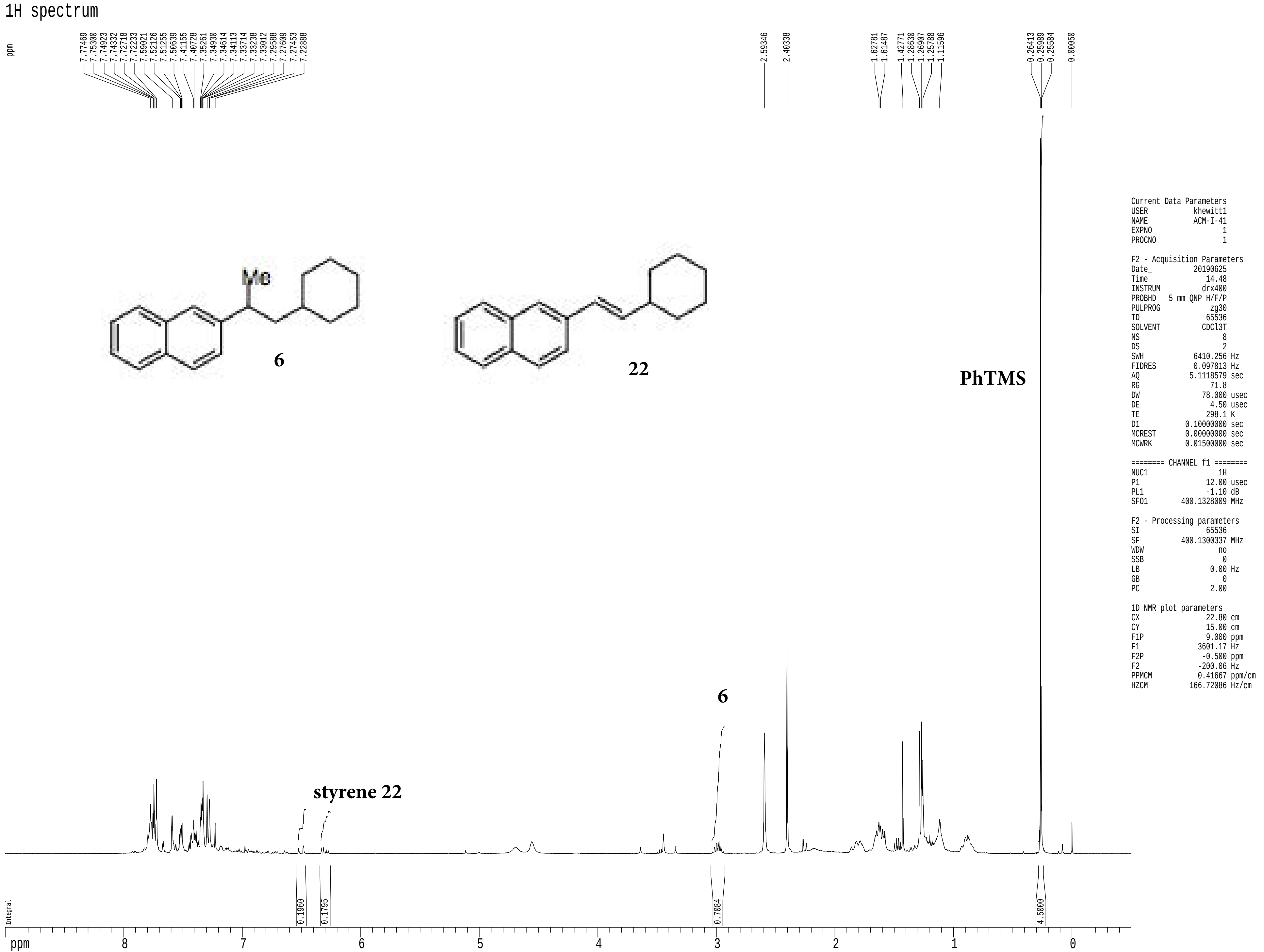
1H spectrum
ppm
7.77469
7.75300
7.74923
7.74332
7.72718
7.72233
7.59021
7.52126
7.51255
7.50639
7.41155
7.40728
7.35261
7.34930
7.34614
7.34113
7.33714
7.33238
7.33012
7.29588
7.27609
7.27453
7.22888
2.59346
2.40338
1.62781
1.61487
1.42771
1.28630
1.26907
1.25788
1.11596
0.26413
0.25989
0.25584
0.00050
Me
6
22
PhTMS
6
styrene 22
Integral
0.1960
0.1795
0.7084
4.5000
ppm
8
7
6
5
4
3
2
1
0
Current Data Parameters
USER khewitt1
NAME ACM-I-41
EXPNO 1
PROCNO 1
F2 - Acquisition Parameters
Date_ 20190625
Time 14.48
INSTRUM drx400
PROBHD 5 mm QNP H/F/P
PULPROG zg30
TD 65536
SOLVENT CDCl3T
NS 8
DS 2
SWH 6410.256 Hz
FIDRES 0.097813 Hz
AQ 5.1118579 sec
RG 71.8
DW 78.000 usec
DE 4.50 usec
TE 298.1 K
D1 0.10000000 sec
MCREST 0.00000000 sec
MCWRK 0.01500000 sec
======== CHANNEL f1 ========
NUC1 1H
P1 12.00 usec
PL1 -1.10 dB
SFO1 400.1328009 MHz
F2 - Processing parameters
SI 65536
SF 400.1300337 MHz
WDW no
SSB 0
LB 0.00 Hz
GB 0
PC 2.00
1D NMR plot parameters
CX 22.80 cm
CY 15.00 cm
F1P 9.000 ppm
F1 3601.17 Hz
F2P -0.500 ppm
F2 -200.06 Hz
PPMCM 0.41667 ppm/cm
HZCM 166.72086 Hz/cm

1H spectrum

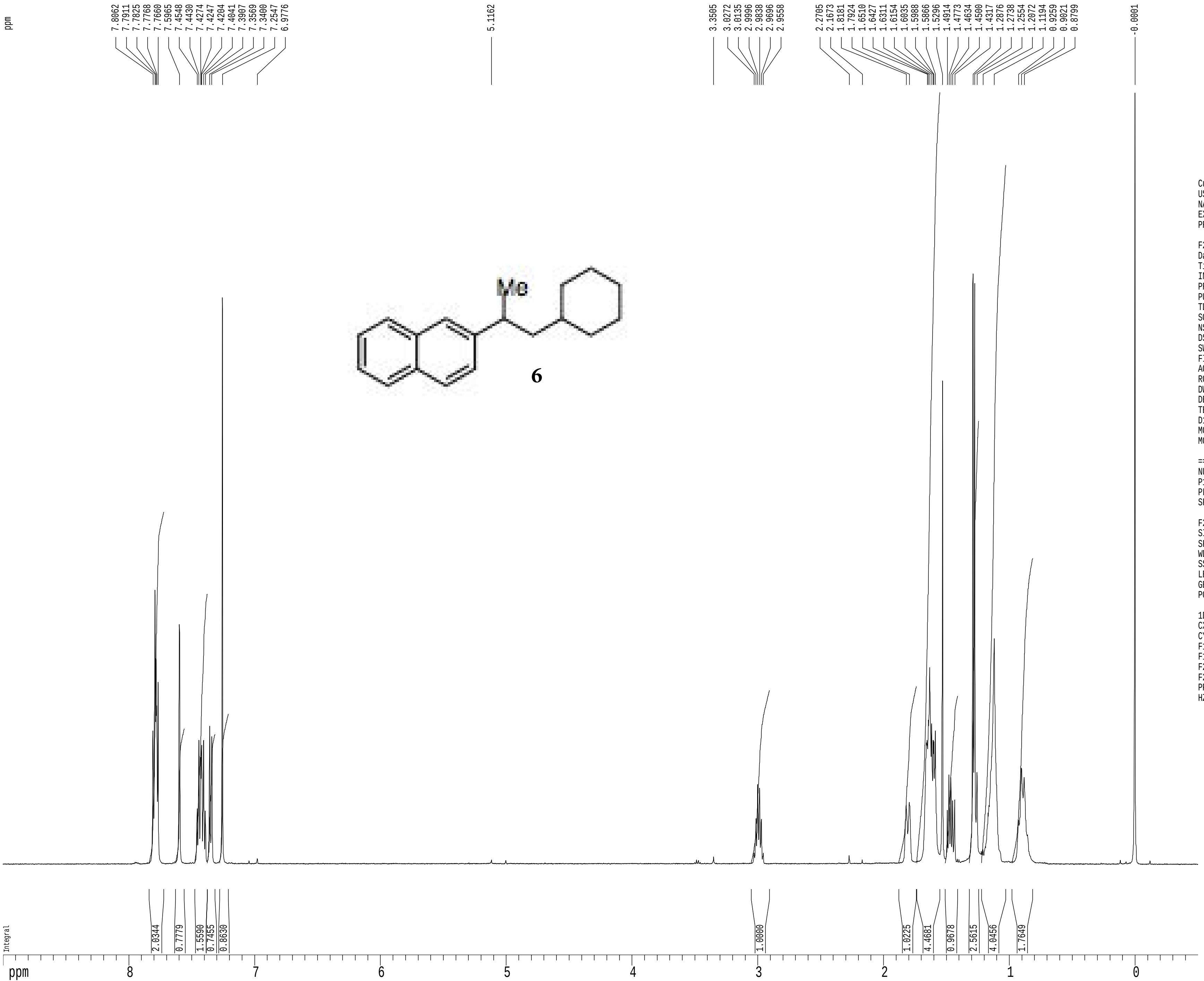


```
Current Data Parameters
USER           khewitt1
NAME        ACM-I-126ch
EXPNO                 1
PROCNO                1

F2 - Acquisition Parameters
Date_          20191115
Time              18.14
INSTRUM         cryo500
PROBHD   5 mm CPTCI 1H-
PULPROG            zg30
TD                81728
SOLVENT           CDCl3T
NS                   10
DS                    2
SWH          8012.820 Hz
FIDRES       0.098043 Hz
AQ          5.0998774 sec
RG                  4.5
DW            62.400 usec
DE              6.00 usec
TE              298.2 K
D1         0.10000000 sec
MCREST     0.00000000 sec
MCWRK      0.01500000 sec

======== CHANNEL f1 ========
NUC1                 1H
P1              7.50 usec
PL1              1.60 dB
SFO1      500.2235015 MHz

F2 - Processing parameters
SI                65536
SF        500.2200341 MHz
WDW                  EM
SSB                   0
LB              0.30 Hz
GB                    0
PC                 1.00

1D NMR plot parameters
CX              22.80 cm
CY              15.00 cm
F1P             9.000 ppm
F1            4501.98 Hz
F2P            -0.500 ppm
F2            -250.11 Hz
PPMCM         0.41667 ppm/cm
HZCM        208.42502 Hz/cm
```

Z-restored spin-echo 13C spectrum with 1H decoupling

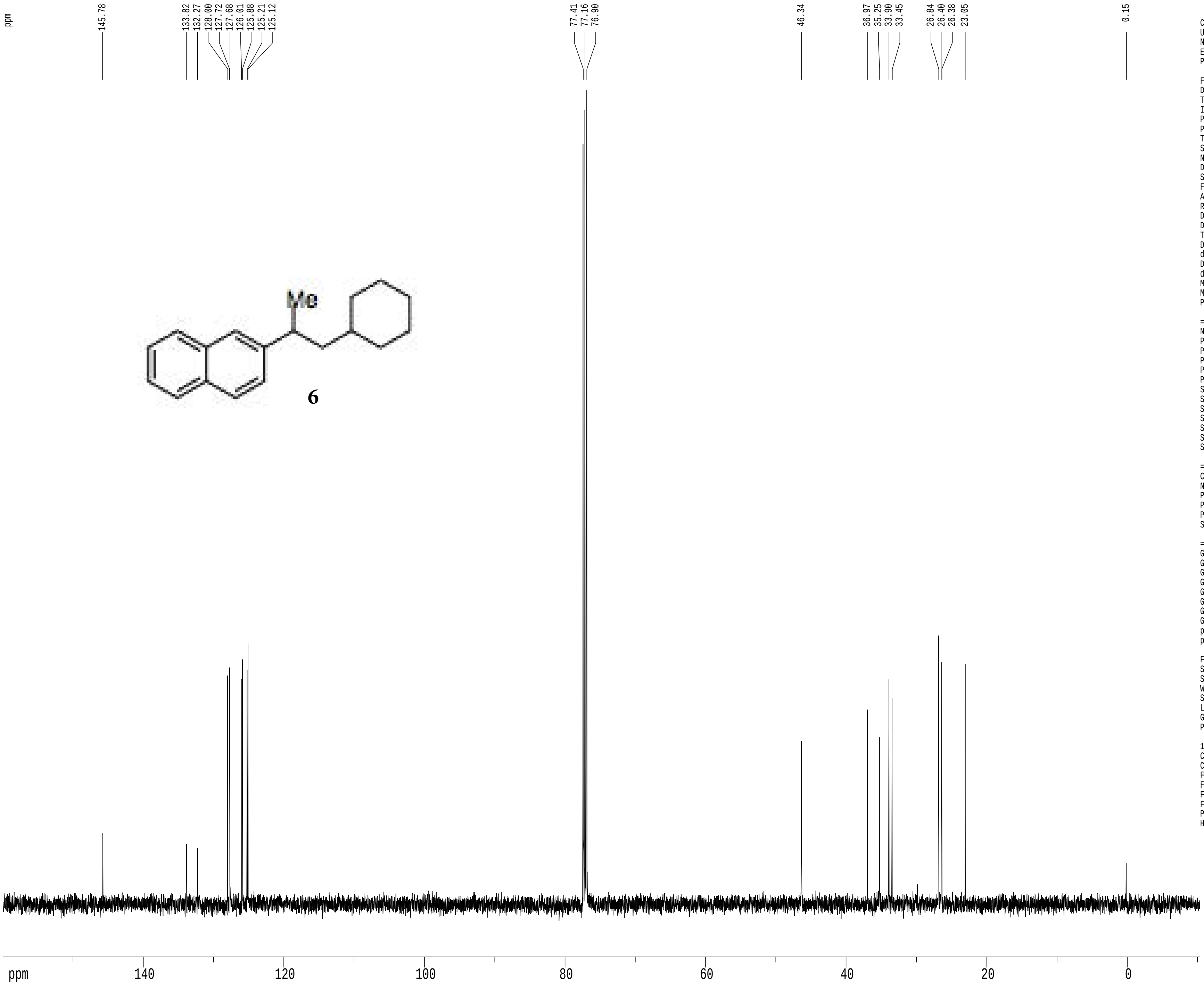

Current Data Parameters
USER khewitt1
NAME ACM-I-126ch
EXPNO 2
PROCNO 1

F2 - Acquisition Parameters
Date_ 20191115
Time 18.29
INSTRUM cryo500
PROBHD 5 mm CPTCI 1H-
PULPROG SpinEchopg30gp2.prd
TD 65536
SOLVENT CDCl3
NS 616
DS 16
SWH 30303.031 Hz
FIDRES 0.462388 Hz
AQ 1.0813940 sec
RG 7298.2
DW 16.500 usec
DE 6.00 usec
TE 298.2 K
D1 0.25000000 sec
d11 0.03000000 sec
D16 0.00020000 sec
d17 0.00019600 sec
MCREST 0.00000000 sec
MCWRK 0.01500000 sec
P2 33.10 usec

======== CHANNEL f1 ========
NUC1 13C
P1 16.55 usec
P12 2000.00 usec
P20 500.00 usec
PL0 120.00 dB
PL1 -1.00 dB
SFO1 125.7942548 MHz
SP2 2.70 dB
SP4 2.70 dB
SPNAM2 Crp60comp.4
SPNAM4 Crp60,0.5,20.1
SPOFF2 0.00 Hz
SPOFF4 0.00 Hz

======== CHANNEL f2 ========
CPDPRG2 waltz16
NUC2 1H
PCPD2 100.00 usec
PL2 1.60 dB
PL12 23.54 dB
SFO2 500.2225011 MHz

====== GRADIENT CHANNEL =====
GPNAM1 SINE.100
GPNAM2 SINE.100
GPX1 0.00 %
GPX2 0.00 %
GPY1 0.00 %
GPY2 0.00 %
GPZ1 30.00 %
GPZ2 50.00 %
p15 500.00 usec
p16 1000.00 usec

F2 - Processing parameters
SI 65536
SF 125.7804062 MHz
WDW EM
SSB 0
LB 1.00 Hz
GB 0
PC 2.00

1D NMR plot parameters
CX 22.80 cm
CY 15.65 cm
F1P 160.000 ppm
F1 20124.87 Hz
F2P -10.358 ppm
F2 -1302.84 Hz
PPMCM 7.47185 ppm/cm
HZCM 939.81177 Hz/cm

1H spectrum

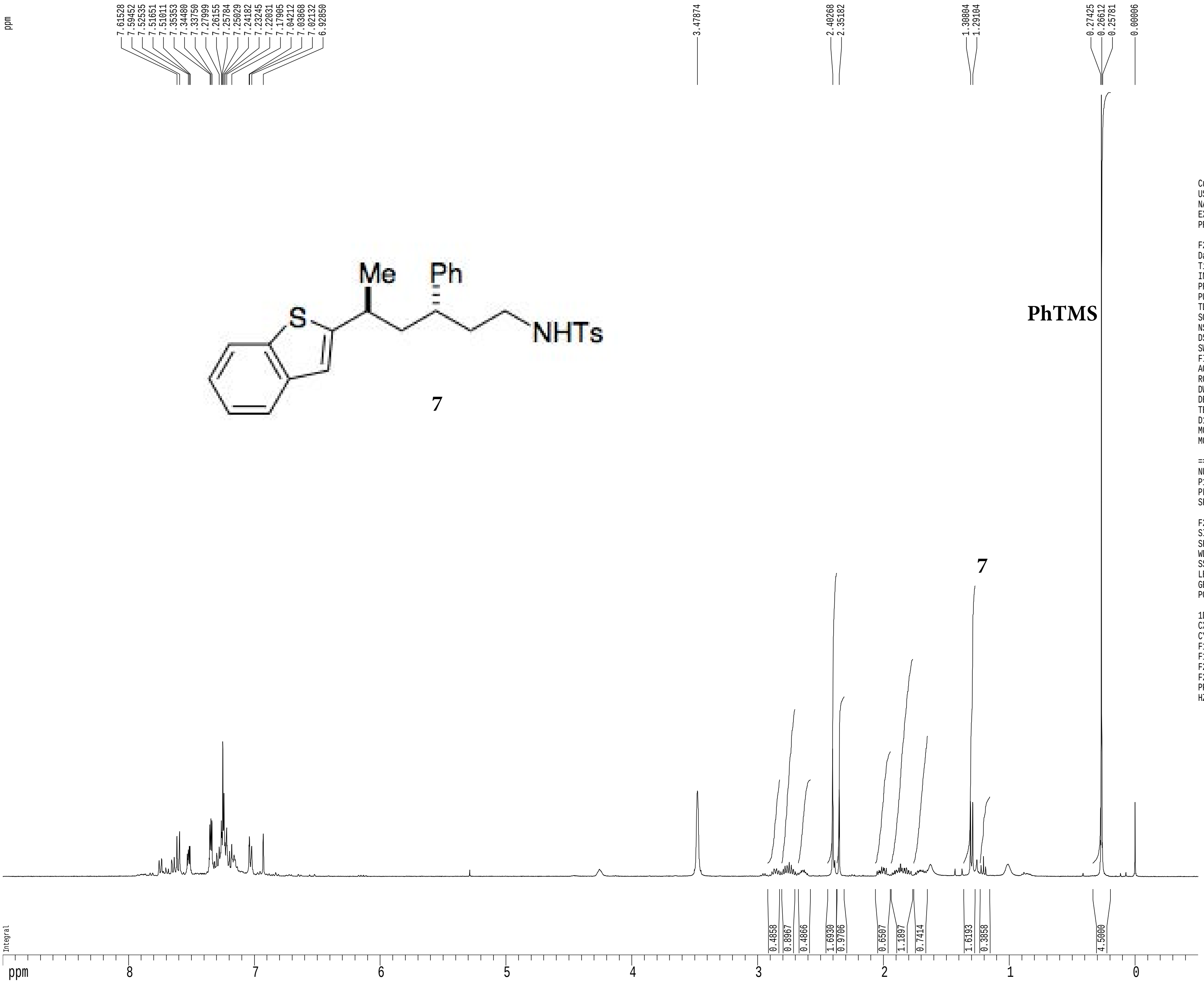


```
Current Data Parameters
USER          khewitt1
NAME          KAH-I-222
EXPNO                1
PROCNO               1

F2 - Acquisition Parameters
Date_          20181208
Time              14.51
INSTRUM          drx400
PROBHD   5 mm QNP H/F/P
PULPROG            zg30
TD                38460
SOLVENT           CDCl3
NS                    8
DS                    2
SWH            6410.256 Hz
FIDRES         0.166673 Hz
AQ            2.9999299 sec
RG                  181
DW               78.000 usec
DE                 4.50 usec
TE                298.0 K
D1           0.10000000 sec
MCREST       0.00000000 sec
MCWRK        0.01500000 sec

======== CHANNEL f1 ========
NUC1                 1H
P1                12.00 usec
PL1               -1.10 dB
SFO1        400.1328009 MHz

F2 - Processing parameters
SI                65536
SF          400.1300249 MHz
WDW                  no
SSB                   0
LB                 0.00 Hz
GB                    0
PC                 2.00

1D NMR plot parameters
CX                22.80 cm
CY                15.00 cm
F1P               9.000 ppm
F1              3601.17 Hz
F2P              -0.500 ppm
F2              -200.06 Hz
PPMCM           0.41667 ppm/cm
HZCM          166.72086 Hz/cm
```

1H spectrum

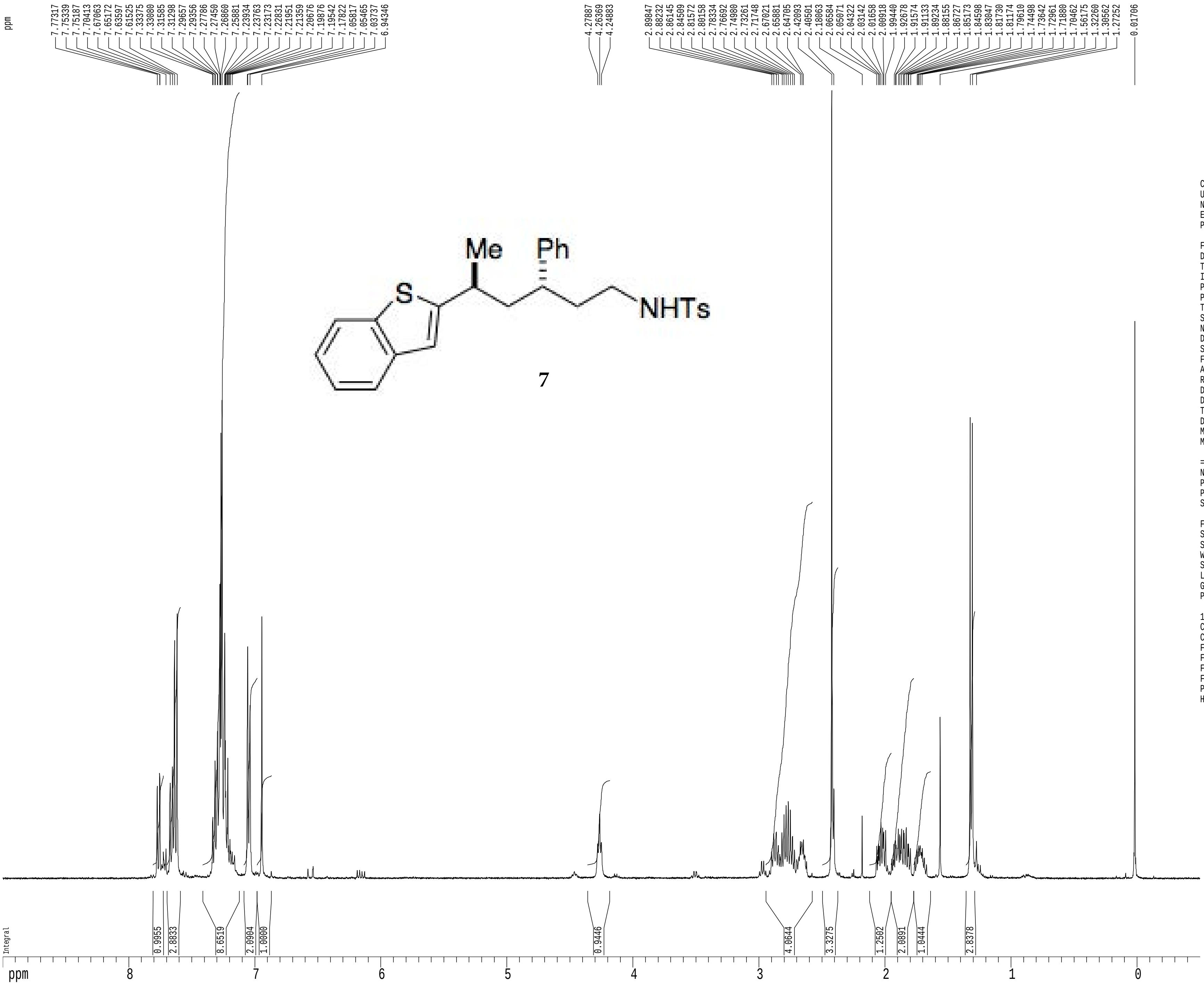

Current Data Parameters
USER khewitt1
NAME KAH-I-222-2
EXPNO 1
PROCNO 1

F2 - Acquisition Parameters
Date_ 20190329
Time 15.37
INSTRUM drx400
PROBHD 5 mm QNP H/F/P
PULPROG zg30
TD 38460
SOLVENT CDCl3T
NS 8
DS 2
SWH 6410.256 Hz
FIDRES 0.166673 Hz
AQ 2.9999299 sec
RG 287.4
DW 78.000 usec
DE 4.50 usec
TE 298.1 K
D1 0.10000000 sec
MCREST 0.00000000 sec
MCWRK 0.01500000 sec

======== CHANNEL f1 ========
NUC1 1H
P1 12.00 usec
PL1 -1.10 dB
SFO1 400.1328009 MHz

F2 - Processing parameters
SI 65536
SF 400.1300175 MHz
WDW no
SSB 0
LB 0.00 Hz
GB 0
PC 2.00

1D NMR plot parameters
CX 22.80 cm
CY 15.00 cm
F1P 9.000 ppm
F1 3601.17 Hz
F2P -0.500 ppm
F2 -200.06 Hz
PPMCM 0.41667 ppm/cm
HZCM 166.72084 Hz/cm

c13.c

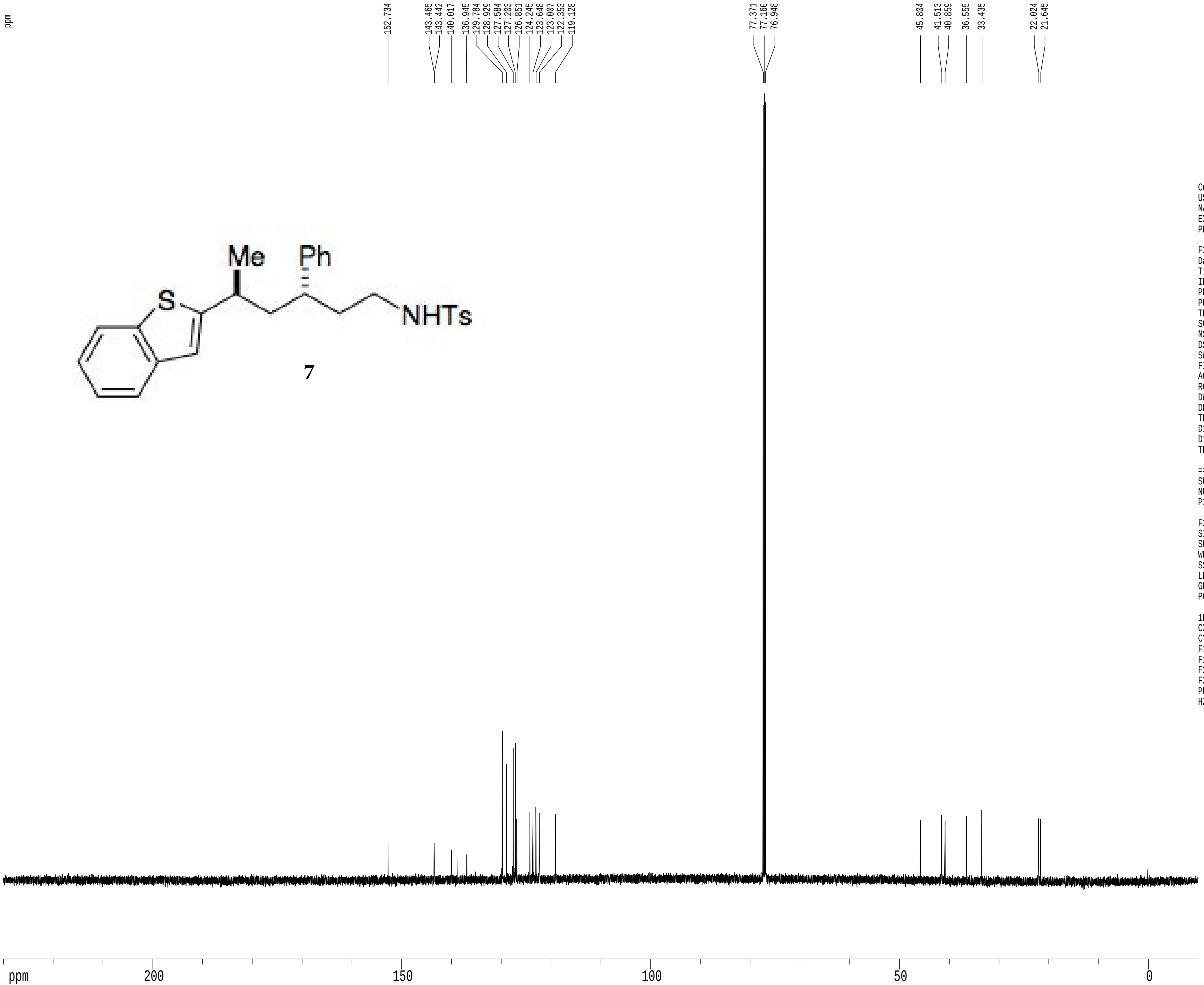

Current Data Parameters
USER khewitt1
NAME KAH-I-222-Z-C
EXPNO 1
PROCNO 1

F2 - Acquisition Parameters
Date_ 20210713
Time 14.14
INSTRUM av600
PROBHD 5 mm CPBBO BB-
PULPROG zgdc30
TD 65536
SOLVENT CDCl3T
NS 146
DS 4
SWH 36231.883 Hz
FIDRES 0.552855 Hz
AQ 0.9044468 sec
RG 2050
DW 13.800 usec
DE 19.63 usec
TE 298.0 K
D1 0.40000001 sec
D11 0.03000000 sec
TD0 1

======== CHANNEL f1 ========
SFO1 150.9194080 MHz
NUC1 13C
P1 10.10 usec

F2 - Processing parameters
SI 65536
SF 150.9027958 MHz
WDW no
SSB 0
LB 0.00 Hz
GB 0
PC 1.00

1D NMR plot parameters
CX 22.80 cm
CY 15.00 cm
F1P 230.136 ppm
F1 34728.11 Hz
F2P -9.965 ppm
F2 -1503.78 Hz
PPMCM 10.53074 ppm/cm
HZCM 1589.11780 Hz/cm

1H spectrum

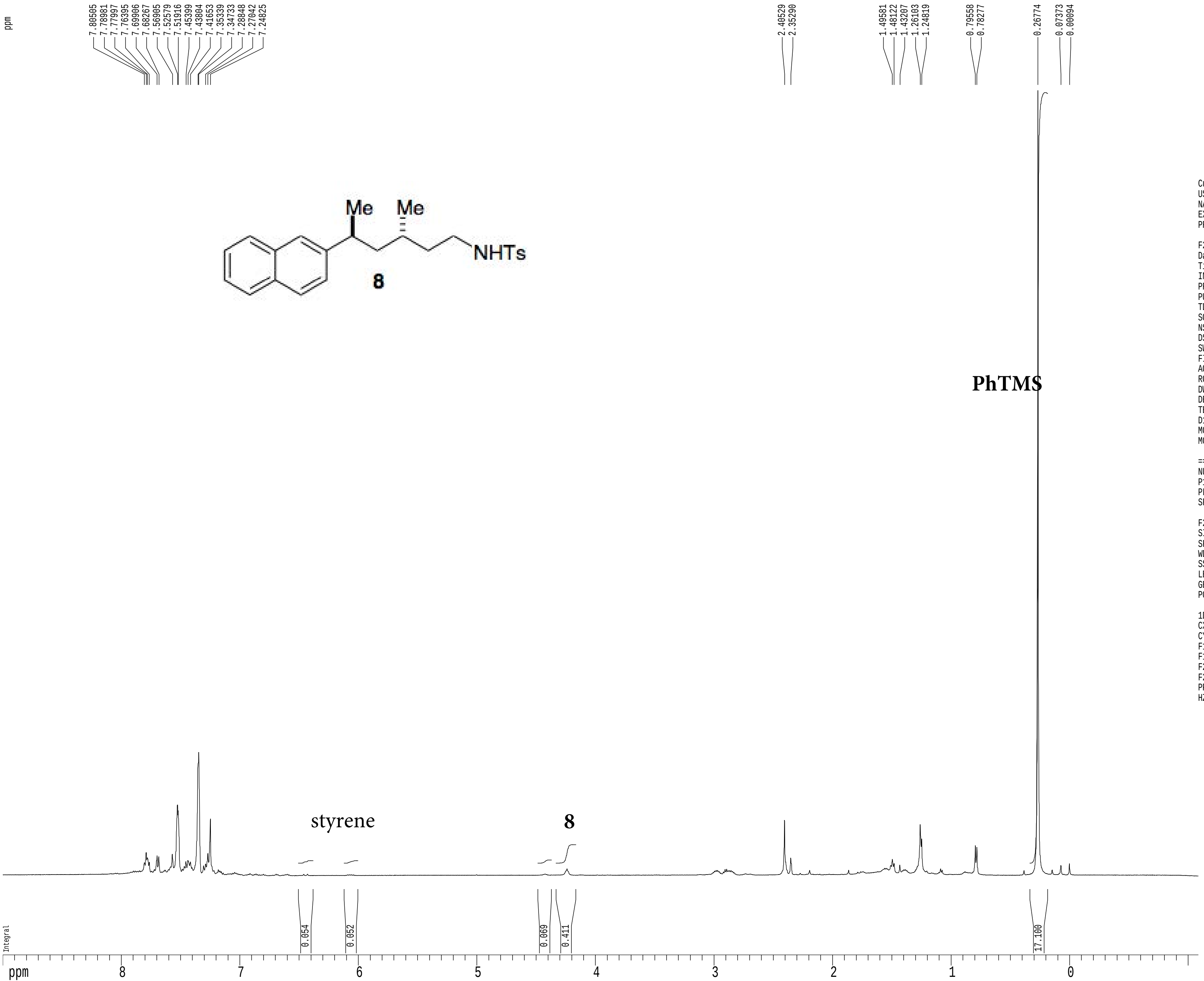


```
Current Data Parameters
USER          caherber
NAME     CAH-I-247-Crude
EXPNO                  1
PROCNO                 1

F2 - Acquisition Parameters
Date_           20210710
Time               11.36
INSTRUM          cryo500
PROBHD   5 mm CPTCI 1H-
PULPROG             zg30
TD                 81728
SOLVENT            CDCl3
NS                     8
DS                     2
SWH            8012.820 Hz
FIDRES         0.098043 Hz
AQ            5.0998774 sec
RG                   6.3
DW                62.400 usec
DE                  6.00 usec
TE                 298.0 K
D1           0.10000000 sec
MCREST       0.00000000 sec
MCWRK        0.01500000 sec

======== CHANNEL f1 ========
NUC1                  1H
P1                  9.75 usec
PL1                 1.60 dB
SFO1         500.2235015 MHz

F2 - Processing parameters
SI                 65536
SF           500.2200370 MHz
WDW                   no
SSB                    0
LB                  0.00 Hz
GB                     0
PC                  1.00

1D NMR plot parameters
CX                 22.80 cm
CY                 15.00 cm
F1P                9.000 ppm
F1               4501.98 Hz
F2P               -1.083 ppm
F2               -541.88 Hz
PPMCM            0.44225 ppm/cm
HZCM           221.22217 Hz/cm
```

1H spectrum

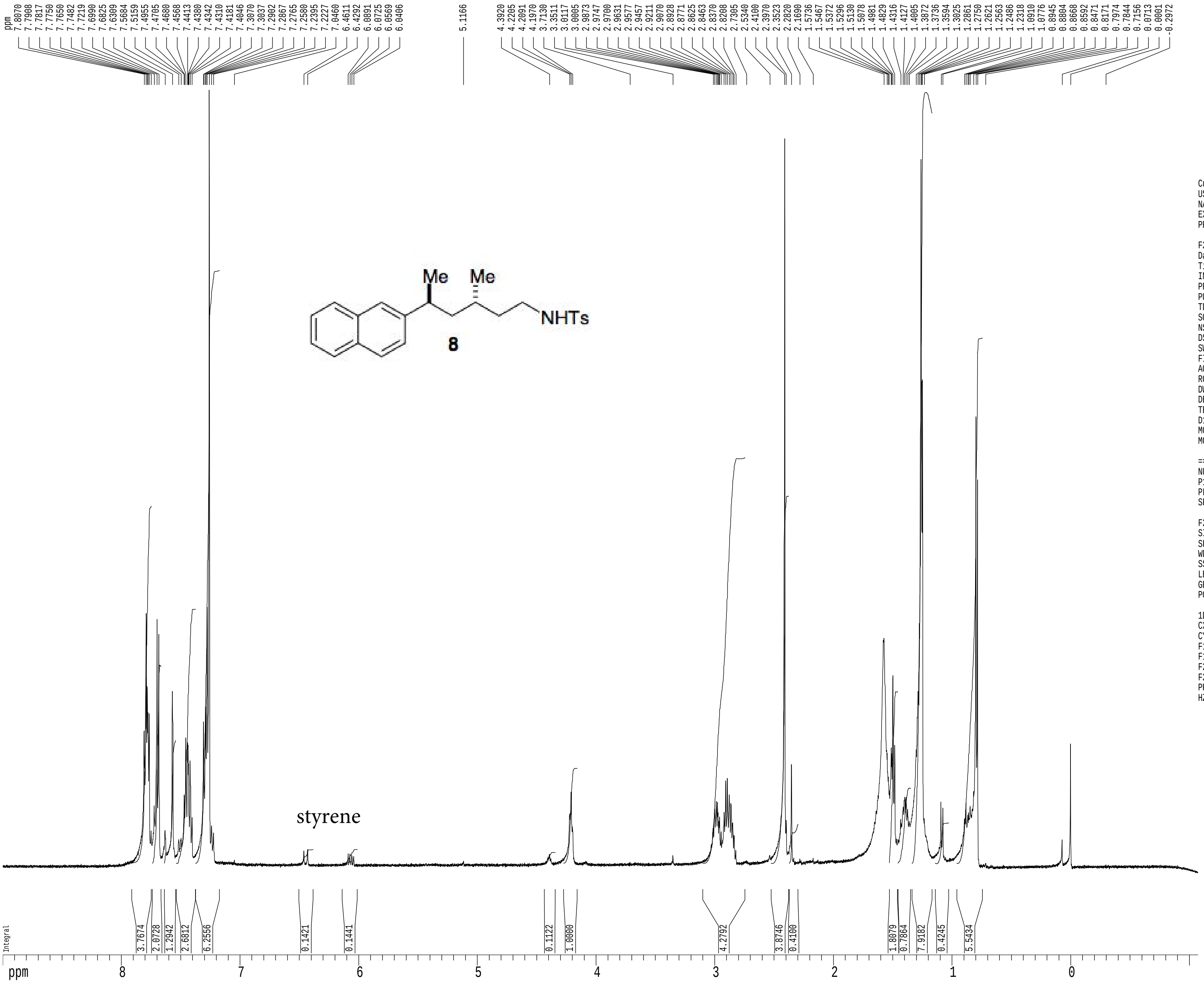


```
Current Data Parameters
USER          caherber
NAME     CAH-I-247-full
EXPNO                1
PROCNO               1

F2 - Acquisition Parameters
Date_         20210713
Time              9.25
INSTRUM        cryo500
PROBHD   5 mm CPTCI 1H-
PULPROG           zg30
TD               81728
SOLVENT          CDCl3
NS                   8
DS                   2
SWH           8012.820 Hz
FIDRES        0.098043 Hz
AQ           5.0998774 sec
RG                 6.3
DW              62.400 usec
DE                6.00 usec
TE               298.0 K
D1          0.10000000 sec
MCREST      0.00000000 sec
MCWRK       0.01500000 sec

======== CHANNEL f1 ========
NUC1                1H
P1                9.75 usec
PL1               1.60 dB
SFO1       500.2235015 MHz

F2 - Processing parameters
SI               65536
SF         500.2200315 MHz
WDW                 no
SSB                  0
LB                0.00 Hz
GB                   0
PC                1.00

1D NMR plot parameters
CX               22.80 cm
CY               15.00 cm
F1P              9.000 ppm
F1             4501.98 Hz
F2P             -1.072 ppm
F2             -536.38 Hz
PPMCM          0.44177 ppm/cm
HZCM         220.98083 Hz/cm
```

Z-restored spin-echo 13C spectrum with 1H decoupling

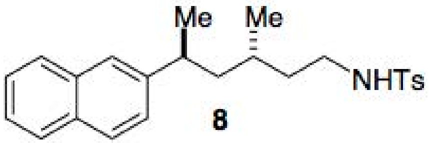

8

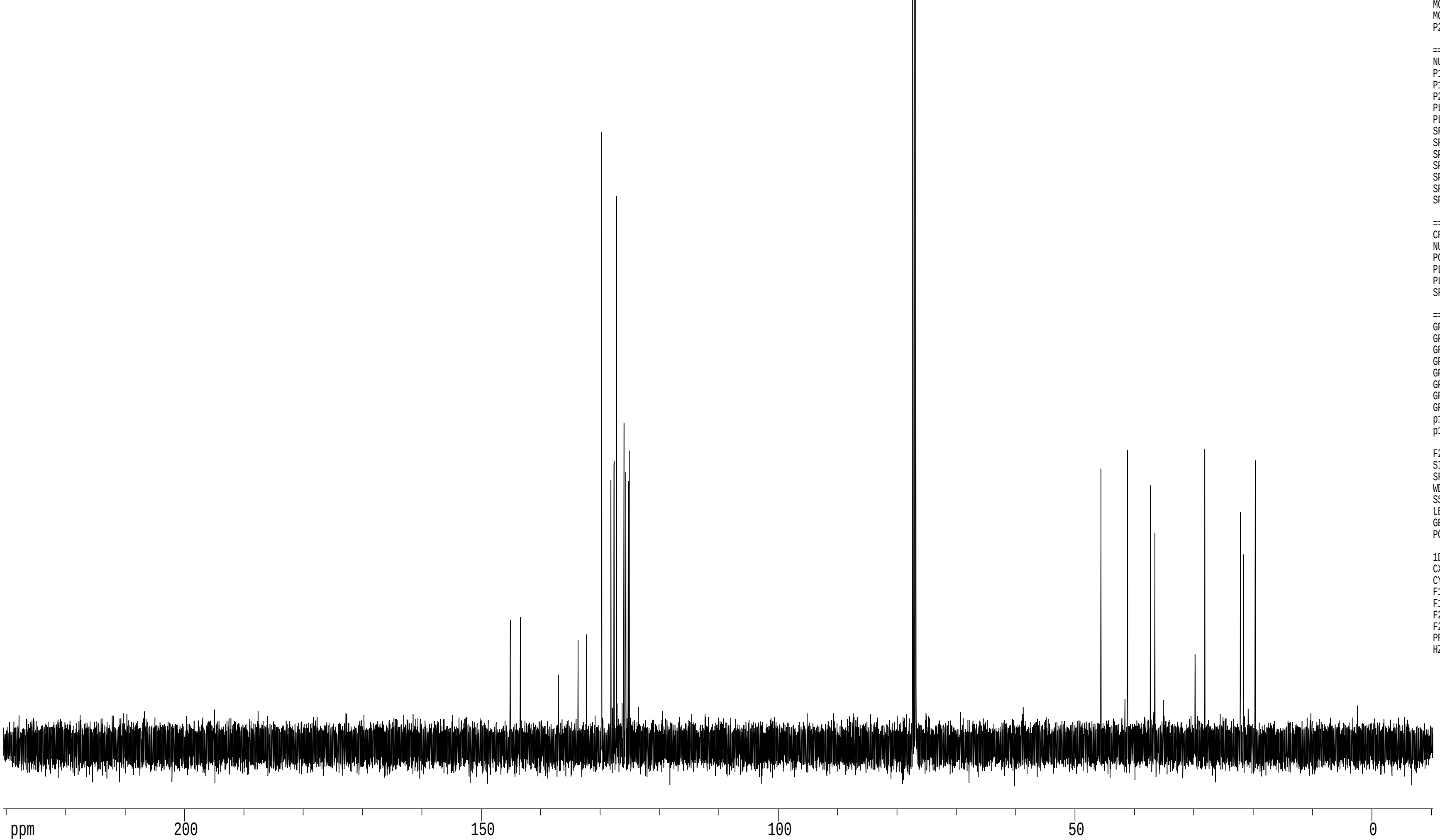


```
Current Data Parameters
USER          caherber
NAME     CAH-I-247-full
EXPNO                 2
PROCNO                1

F2 - Acquisition Parameters
Date_          20210713
Time               9.36
INSTRUM         cryo500
PROBHD   5 mm CPTCI 1H-
PULPROG  SpinEchopg30gp2.prd
TD                65536
SOLVENT           CDCl3
NS                  512
DS                   16
SWH           30303.031 Hz
FIDRES         0.462388 Hz
AQ            1.0813940 sec
RG               7298.2
DW               16.500 usec
DE                 6.00 usec
TE                298.0 K
D1           0.25000000 sec
d11          0.03000000 sec
D16          0.00020000 sec
d17          0.00019600 sec
MCREST       0.00000000 sec
MCWRK        0.01500000 sec
P2                37.70 usec

======== CHANNEL f1 ========
NUC1                13C
P1                18.85 usec
P12             2000.00 usec
P20              500.00 usec
PL0              120.00 dB
PL1               -1.00 dB
SFO1        125.7942548 MHz
SP2                1.55 dB
SP4                1.55 dB
SPNAM2      Crp60comp.4
SPNAM4   Crp60,0.5,20.1
SPOFF2             0.00 Hz
SPOFF4             0.00 Hz

======== CHANNEL f2 ========
CPDPRG2         waltz16
NUC2                 1H
PCPD2            100.00 usec
PL2                1.60 dB
PL12              22.00 dB
SFO2        500.2225011 MHz

====== GRADIENT CHANNEL =====
GPNAM1         SINE.100
GPNAM2         SINE.100
GPX1               0.00 %
GPX2               0.00 %
GPY1               0.00 %
GPY2               0.00 %
GPZ1              30.00 %
GPZ2              50.00 %
p15              500.00 usec
p16             1000.00 usec

F2 - Processing parameters
SI                65536
SF          125.7804190 MHz
WDW                  no
SSB                   0
LB                 0.00 Hz
GB                    0
PC                 2.00

1D NMR plot parameters
CX                22.80 cm
CY                15.65 cm
F1P             230.637 ppm
F1             29009.68 Hz
F2P             -10.287 ppm
F2             -1293.96 Hz
PPMCM          10.56688 ppm/cm
HZCM         1329.10706 Hz/cm
```

1H spectrum

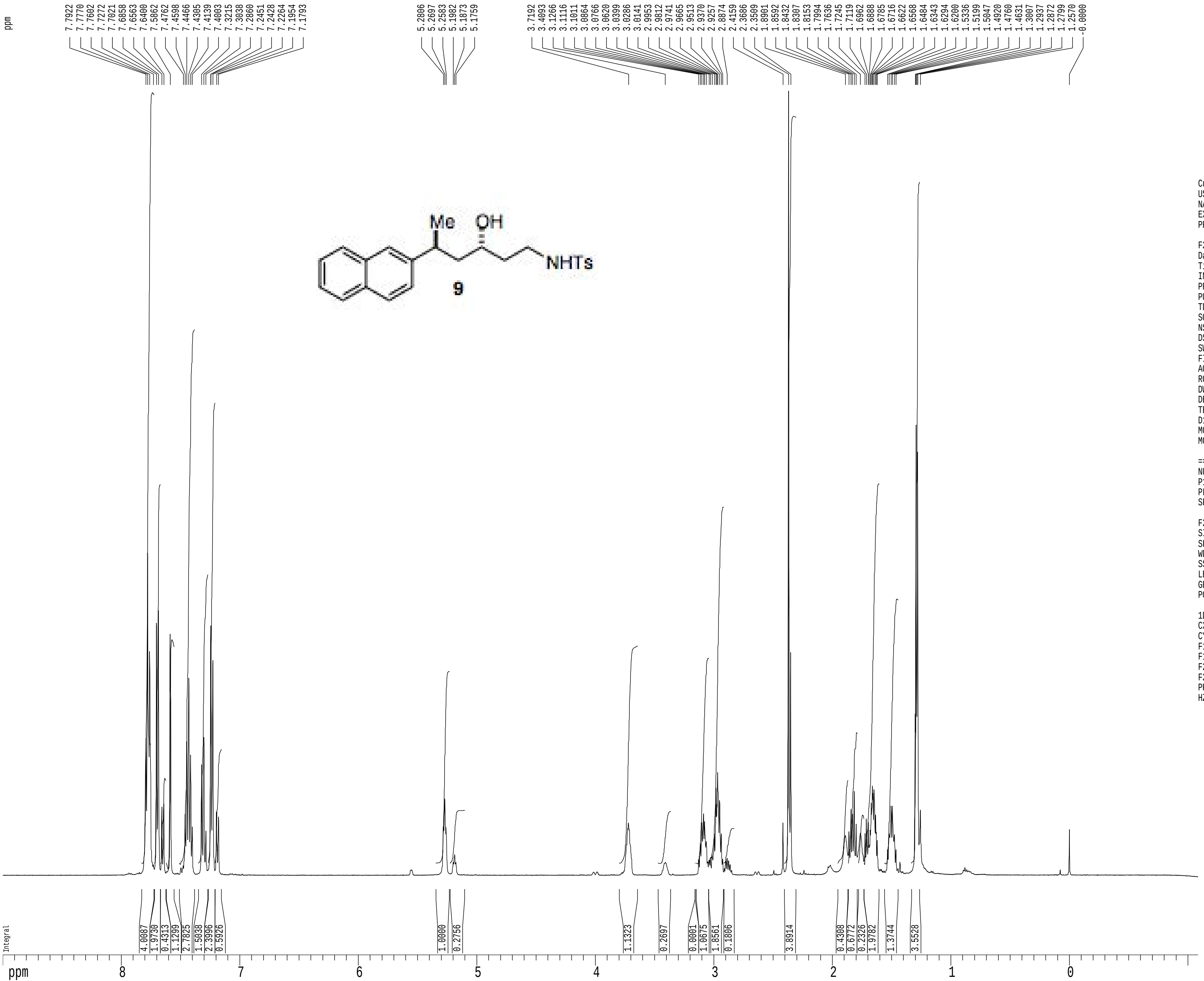

Current Data Parameters
USER caherber
NAME CAH-I-239-full
EXPNO 1
PROCNO 1

F2 - Acquisition Parameters
Date_ 20210623
Time 11.35
INSTRUM cryo500
PROBHD 5 mm CPTCI 1H-
PULPROG zg30
TD 81728
SOLVENT CDCl3
NS 8
DS 2
SWH 8012.820 Hz
FIDRES 0.098043 Hz
AQ 5.0998774 sec
RG 5
DW 62.400 usec
DE 6.00 usec
TE 298.0 K
D1 0.10000000 sec
MCREST 0.00000000 sec
MCWRK 0.01500000 sec

======== CHANNEL f1 ========
NUC1 1H
P1 9.75 usec
PL1 1.60 dB
SFO1 500.2235015 MHz

F2 - Processing parameters
SI 65536
SF 500.2200379 MHz
WDW no
SSB 0
LB 0.00 Hz
GB 0
PC 1.00

1D NMR plot parameters
CX 22.80 cm
CY 15.00 cm
F1P 9.000 ppm
F1 4501.98 Hz
F2P -1.085 ppm
F2 -542.74 Hz
PPMCM 0.44232 ppm/cm
HZCM 221.25967 Hz/cm

Z-restored spin-echo 13C spectrum with 1H decoupling

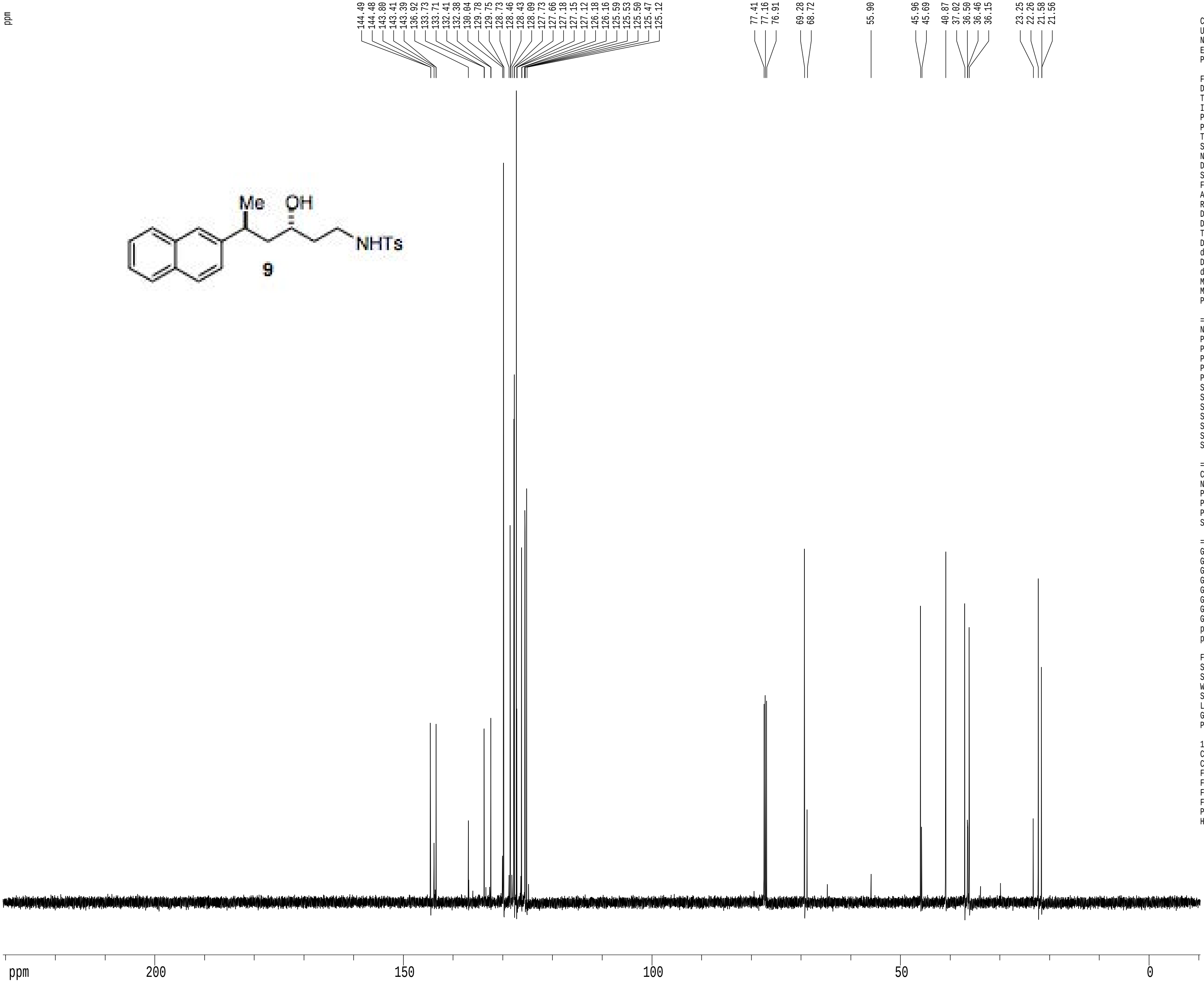


```
Current Data Parameters
USER            caherber
NAME      CAH-I-239-full
EXPNO                  2
PROCNO                 1

F2 - Acquisition Parameters
Date_           20210623
Time               11.43
INSTRUM          cryo500
PROBHD   5 mm CPTCI 1H-
PULPROG  SpinEchopg30gp2.prd
TD                 65536
SOLVENT            CDCl3
NS                   296
DS                    16
SWH            30303.031 Hz
FIDRES          0.462388 Hz
AQ             1.0813940 sec
RG               14596.5
DW                16.500 usec
DE                  6.00 usec
TE                 298.0 K
D1            0.25000000 sec
d11           0.03000000 sec
D16           0.00020000 sec
d17           0.00019600 sec
MCREST        0.00000000 sec
MCWRK         0.01500000 sec
P2                 37.70 usec

======== CHANNEL f1 ========
NUC1                 13C
P1                 18.85 usec
P12              2000.00 usec
P20               500.00 usec
PL0               120.00 dB
PL1                -1.00 dB
SFO1         125.7942548 MHz
SP2                 1.55 dB
SP4                 1.55 dB
SPNAM2       Crp60comp.4
SPNAM4    Crp60,0.5,20.1
SPOFF2              0.00 Hz
SPOFF4              0.00 Hz

======== CHANNEL f2 ========
CPDPRG2            waltz16
NUC2                    1H
PCPD2             100.00 usec
PL2                 1.60 dB
PL12               22.00 dB
SFO2         500.2225011 MHz

====== GRADIENT CHANNEL =====
GPNAM1           SINE.100
GPNAM2           SINE.100
GPX1                 0.00 %
GPX2                 0.00 %
GPY1                 0.00 %
GPY2                 0.00 %
GPZ1                30.00 %
GPZ2                50.00 %
p15               500.00 usec
p16              1000.00 usec

F2 - Processing parameters
SI                 65536
SF           125.7804131 MHz
WDW                   no
SSB                    0
LB                  0.00 Hz
GB                     0
PC                  2.00

1D NMR plot parameters
CX                 22.80 cm
CY                 15.65 cm
F1P              230.637 ppm
F1              29009.68 Hz
F2P              -10.287 ppm
F2              -1293.96 Hz
PPMCM           10.56688 ppm/cm
HZCM          1329.10693 Hz/cm
```

1H spectrum

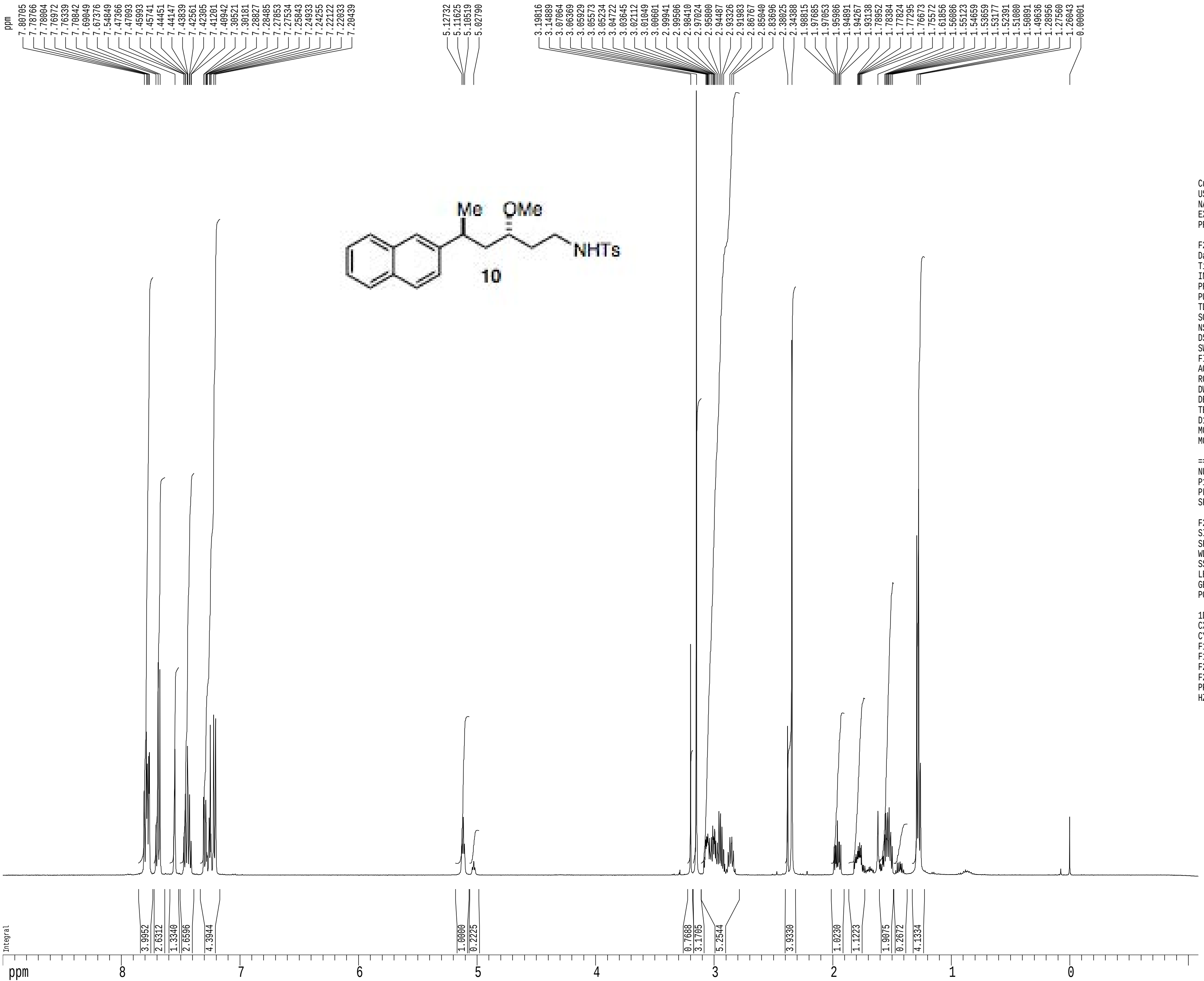


```
Current Data Parameters
USER           caherber
NAME          CAH-I-212
EXPNO                 2
PROCNO                1

F2 - Acquisition Parameters
Date_          20210508
Time              10.24
INSTRUM         cryo500
PROBHD   5 mm CPTCI 1H-
PULPROG            zg30
TD                81728
SOLVENT           CDCl3
NS                    8
DS                    2
SWH            8012.820 Hz
FIDRES         0.098043 Hz
AQ            5.0998774 sec
RG                    4
DW               62.400 usec
DE                 6.00 usec
TE                298.0 K
D1           0.10000000 sec
MCREST       0.00000000 sec
MCWRK        0.01500000 sec

======== CHANNEL f1 ========
NUC1                 1H
P1                 9.75 usec
PL1                1.60 dB
SFO1        500.2235015 MHz

F2 - Processing parameters
SI                65536
SF          500.2200364 MHz
WDW                  no
SSB                   0
LB                 0.00 Hz
GB                    0
PC                 1.00

1D NMR plot parameters
CX                22.80 cm
CY                15.00 cm
F1P               9.000 ppm
F1              4501.98 Hz
F2P              -1.082 ppm
F2              -541.27 Hz
PPMCM           0.44220 ppm/cm
HZCM          221.19518 Hz/cm
```

Z-restored spin-echo 13C spectrum with 1H decoupling

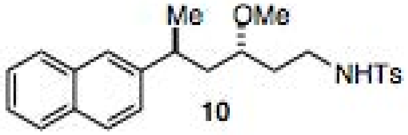

10

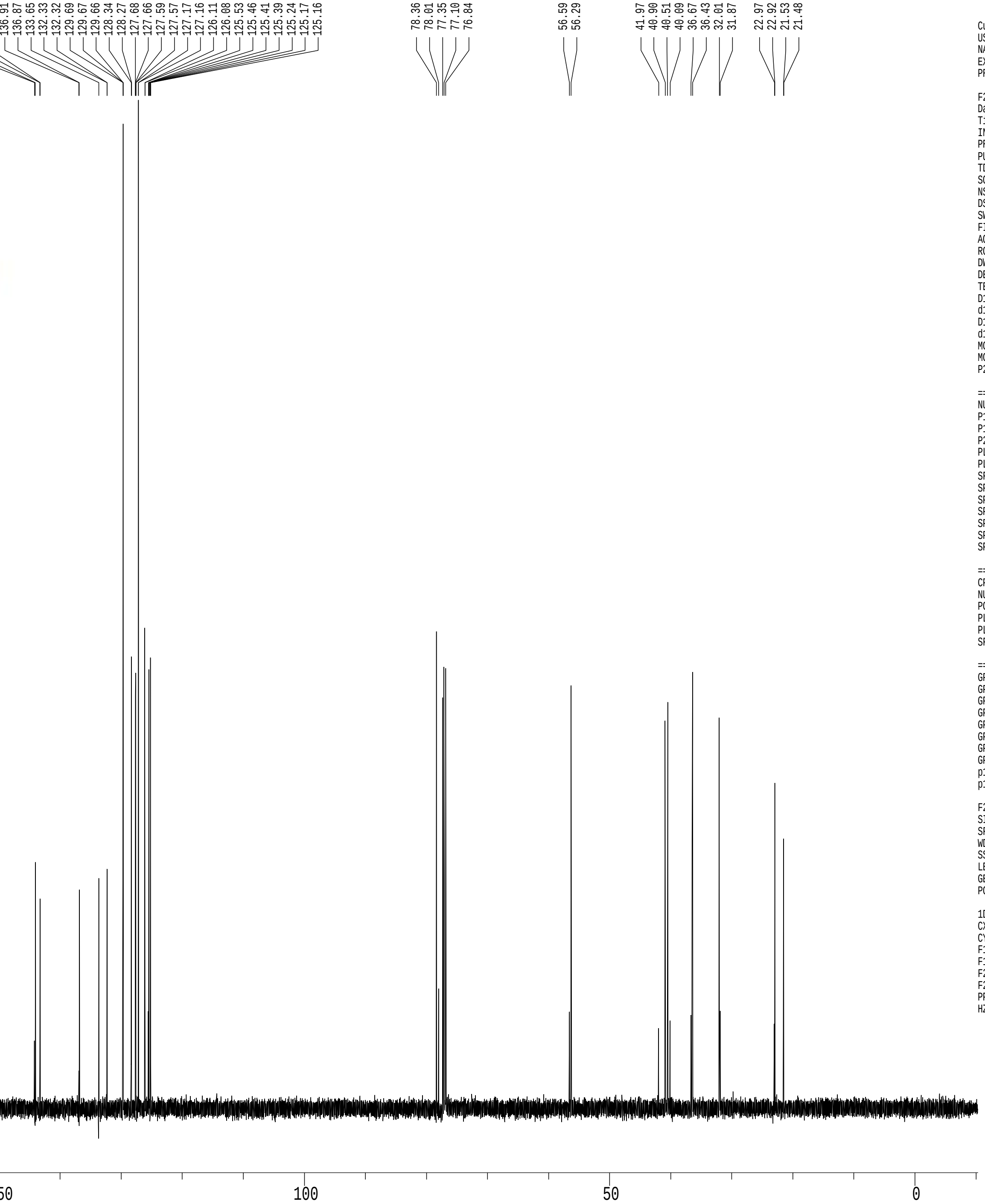

Current Data Parameters
USER caherber
NAME CAH-I-212
EXPNO 3
PROCNO 1

F2 - Acquisition Parameters
Date_ 20210508
Time 10.28
INSTRUM cryo500
PROBHD 5 mm CPTCI 1H-
PULPROG SpinEchopg30gp2.prd
TD 65536
SOLVENT CDCl3
NS 144
DS 16
SWH 30303.031 Hz
FIDRES 0.462388 Hz
AQ 1.0813940 sec
RG 2896.3
DW 16.500 usec
DE 6.00 usec
TE 298.0 K
D1 0.25000000 sec
d11 0.03000000 sec
D16 0.00020000 sec
d17 0.00019600 sec
MCREST 0.00000000 sec
MCWRK 0.01500000 sec
P2 37.70 usec

======== CHANNEL f1 ========
NUC1 13C
P1 18.85 usec
P12 2000.00 usec
P20 500.00 usec
PL0 120.00 dB
PL1 -1.00 dB
SFO1 125.7942548 MHz
SP2 1.55 dB
SP4 1.55 dB
SPNAM2 Crp60comp.4
SPNAM4 Crp60,0.5,20.1
SPOFF2 0.00 Hz
SPOFF4 0.00 Hz

======== CHANNEL f2 ========
CPDPRG2 waltz16
NUC2 1H
PCPD2 100.00 usec
PL2 1.60 dB
PL12 22.00 dB
SFO2 500.2225011 MHz

====== GRADIENT CHANNEL =====
GPNAM1 SINE.100
GPNAM2 SINE.100
GPX1 0.00 %
GPX2 0.00 %
GPY1 0.00 %
GPY2 0.00 %
GPZ1 30.00 %
GPZ2 50.00 %
p15 500.00 usec
p16 1000.00 usec

F2 - Processing parameters
SI 65536
SF 125.7804190 MHz
WDW no
SSB 0
LB 0.00 Hz
GB 0
PC 2.00

1D NMR plot parameters
CX 22.80 cm
CY 15.65 cm
F1P 230.637 ppm
F1 29009.68 Hz
F2P -10.287 ppm
F2 -1293.96 Hz
PPMCM 10.56688 ppm/cm
HZCM 1329.10706 Hz/cm

1H spectrum

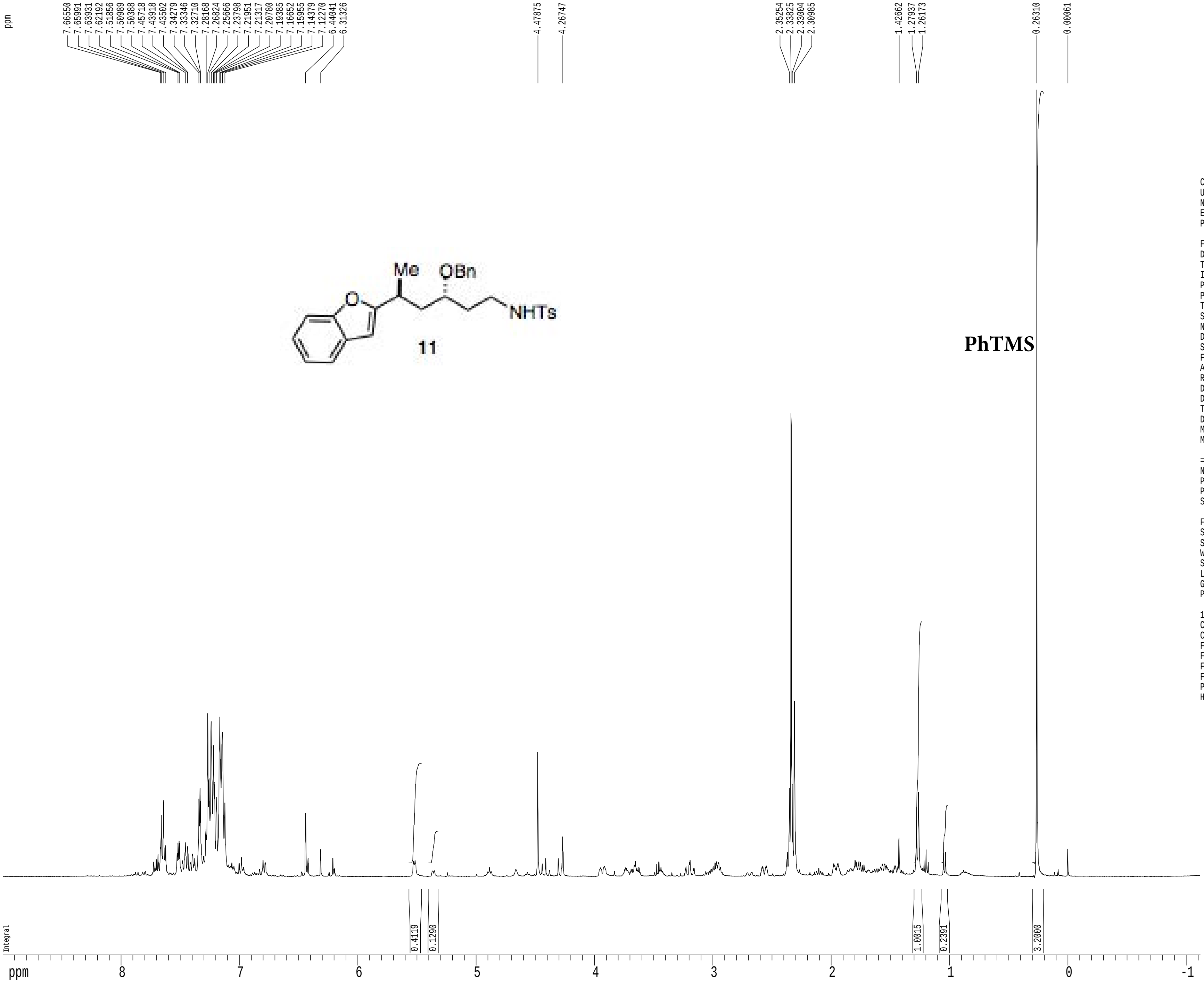

11

Current Data Parameters
USER caherber
NAME CAH-I-233-Crude
EXPNO 1
PROCNO 1

F2 - Acquisition Parameters
Date_ 20210604
Time 16.44
INSTRUM drx400
PROBHD 5 mm QNP H/F/P
PULPROG zg30
TD 65536
SOLVENT CDCl3
NS 8
DS 2
SWH 6410.256 Hz
FIDRES 0.097813 Hz
AQ 5.1118579 sec
RG 71.8
DW 78.000 usec
DE 4.50 usec
TE 298.0 K
D1 0.10000000 sec
MCREST 0.00000000 sec
MCWRK 0.01500000 sec

======== CHANNEL f1 ========
NUC1 1H
P1 12.00 usec
PL1 -1.60 dB
SFO1 400.1328009 MHz

F2 - Processing parameters
SI 65536
SF 400.1300427 MHz
WDW no
SSB 0
LB 0.00 Hz
GB 0
PC 2.00

1D NMR plot parameters
CX 22.80 cm
CY 15.00 cm
F1P 9.000 ppm
F1 3601.17 Hz
F2P -1.117 ppm
F2 -446.91 Hz
PPMCM 0.44372 ppm/cm
HZCM 177.54726 Hz/cm

1H spectrum

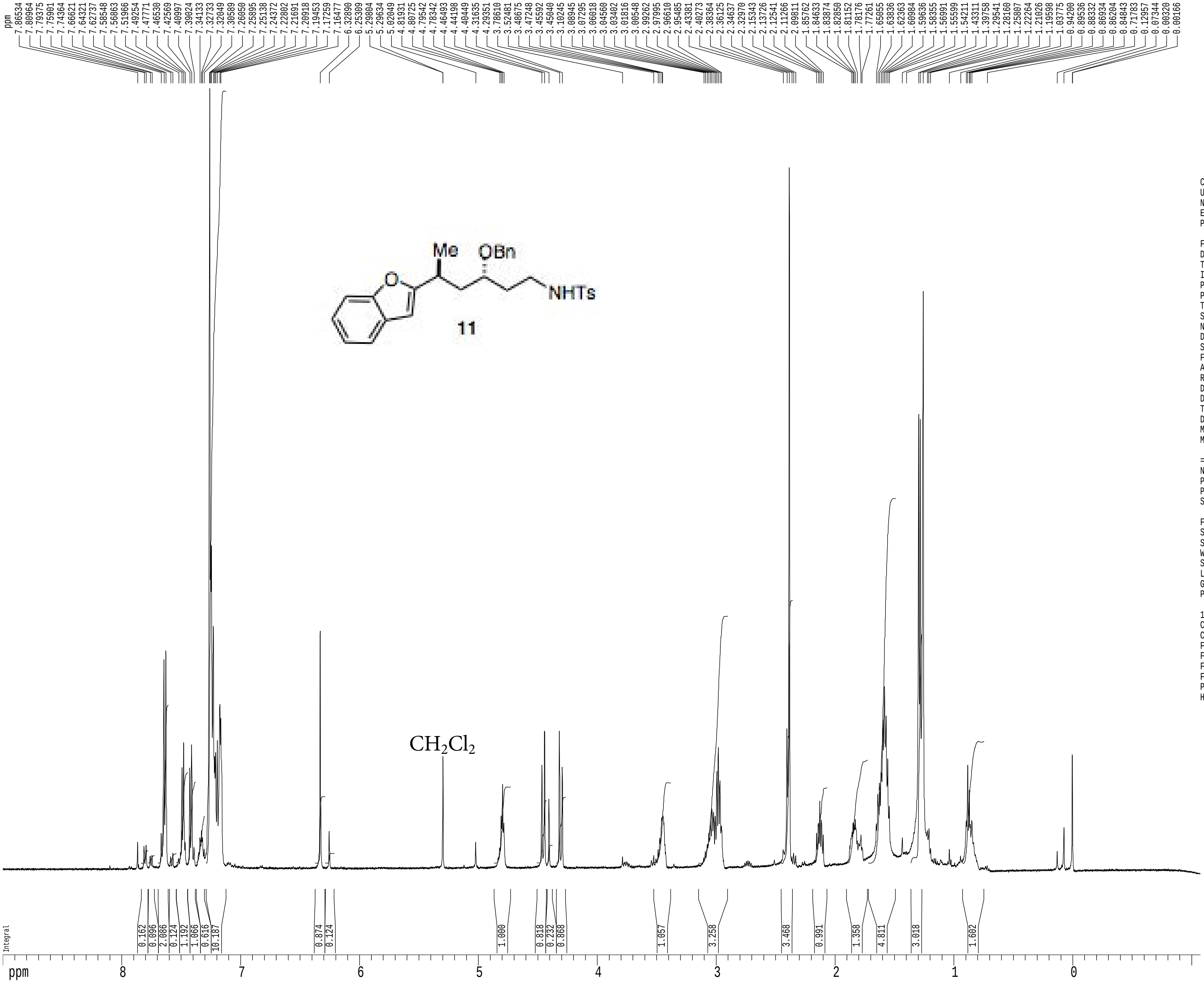


```
Current Data Parameters
USER              caherber
NAME         CAH-I-246-full
EXPNO                    1
PROCNO                   1

F2 - Acquisition Parameters
Date_             20210708
Time                 11.14
INSTRUM            cryo500
PROBHD   5 mm CPTCI 1H-
PULPROG               zg30
TD                   81728
SOLVENT              CDCl3
NS                       8
DS                       2
SWH               8012.820 Hz
FIDRES            0.098043 Hz
AQ               5.0998774 sec
RG                     6.3
DW                  62.400 usec
DE                    6.00 usec
TE                   298.0 K
D1              0.10000000 sec
MCREST          0.00000000 sec
MCWRK           0.01500000 sec

======== CHANNEL f1 ========
NUC1                    1H
P1                    9.75 usec
PL1                   1.60 dB
SFO1          500.2235015 MHz

F2 - Processing parameters
SI                   65536
SF            500.2200313 MHz
WDW                     no
SSB                      0
LB                    0.00 Hz
GB                       0
PC                    1.00

1D NMR plot parameters
CX                   22.80 cm
CY                   15.00 cm
F1P                  9.000 ppm
F1                 4501.98 Hz
F2P                 -1.072 ppm
F2                 -536.14 Hz
PPMCM              0.44175 ppm/cm
HZCM             220.97008 Hz/cm
```

Z-restored spin-echo 13C spectrum with 1H decoupling

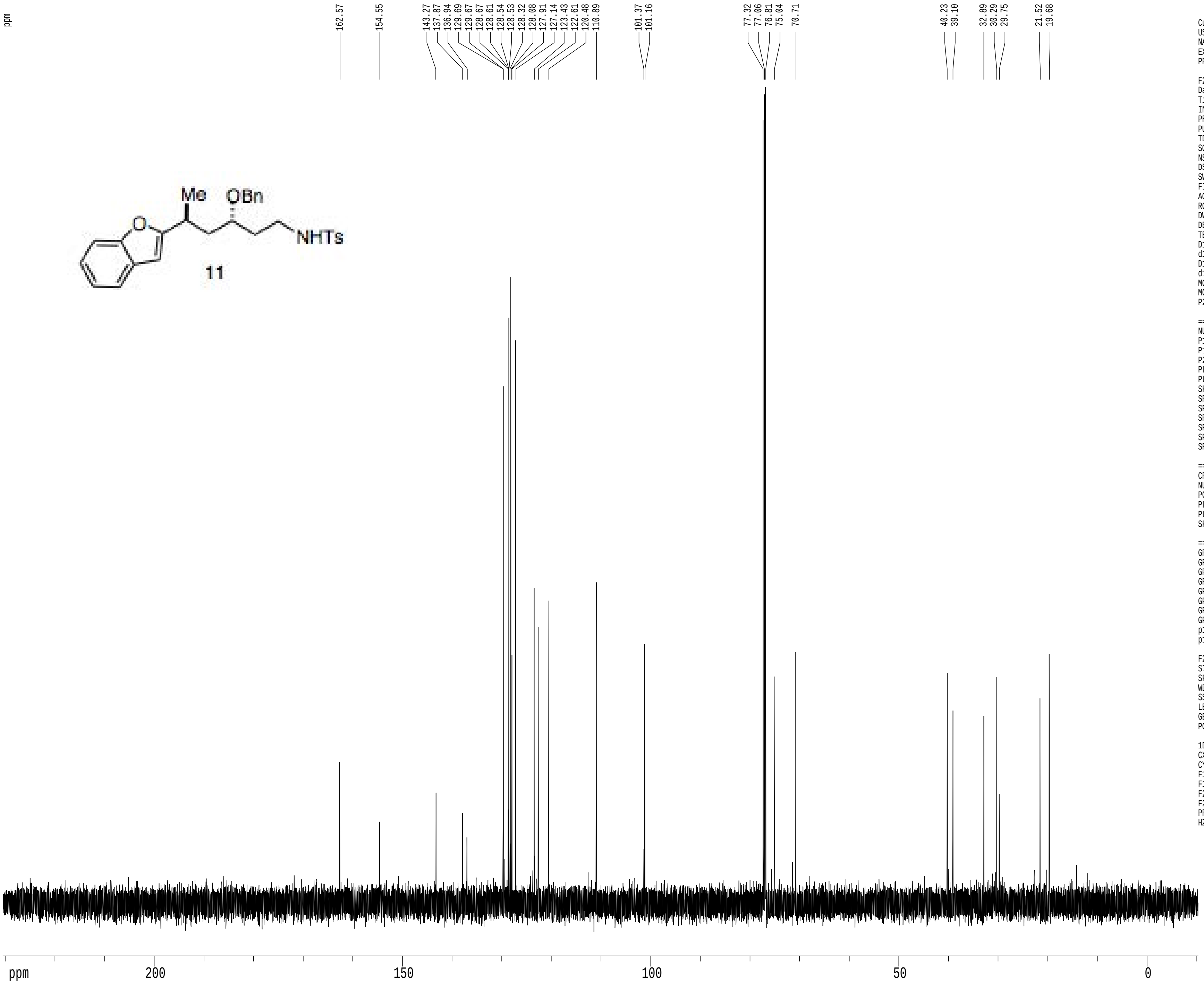

Current Data Parameters
USER caherber
NAME CAH-I-246-full
EXPNO 2
PROCNO 1

F2 - Acquisition Parameters
Date_ 20210708
Time 11.23
INSTRUM cryo500
PROBHD 5 mm CPTCI 1H-
PULPROG SpinEchopg30gp2.prd
TD 65536
SOLVENT CDCl3
NS 448
DS 16
SWH 30303.031 Hz
FIDRES 0.462388 Hz
AQ 1.0813940 sec
RG 14596.5
DW 16.500 usec
DE 6.00 usec
TE 298.0 K
D1 0.25000000 sec
d11 0.03000000 sec
D16 0.00020000 sec
d17 0.00019600 sec
MCREST 0.00000000 sec
MCWRK 0.01500000 sec
P2 37.70 usec

======== CHANNEL f1 ========
NUC1 13C
P1 18.85 usec
P12 2000.00 usec
P20 500.00 usec
PL0 120.00 dB
PL1 -1.00 dB
SFO1 125.7942548 MHz
SP2 1.55 dB
SP4 1.55 dB
SPNAM2 Crp60comp.4
SPNAM4 Crp60,0.5,20.1
SPOFF2 0.00 Hz
SPOFF4 0.00 Hz

======== CHANNEL f2 ========
CPDPRG2 waltz16
NUC2 1H
PCPD2 100.00 usec
PL2 1.60 dB
PL12 22.00 dB
SFO2 500.2225011 MHz

====== GRADIENT CHANNEL =====
GPNAM1 SINE.100
GPNAM2 SINE.100
GPX1 0.00 %
GPX2 0.00 %
GPY1 0.00 %
GPY2 0.00 %
GPZ1 30.00 %
GPZ2 50.00 %
p15 500.00 usec
p16 1000.00 usec

F2 - Processing parameters
SI 65536
SF 125.7804190 MHz
WDW no
SSB 0
LB 0.00 Hz
GB 0
PC 2.00

1D NMR plot parameters
CX 22.80 cm
CY 15.65 cm
F1P 230.637 ppm
F1 29009.68 Hz
F2P -10.287 ppm
F2 -1293.96 Hz
PPMCM 10.56688 ppm/cm
HZCM 1329.10706 Hz/cm

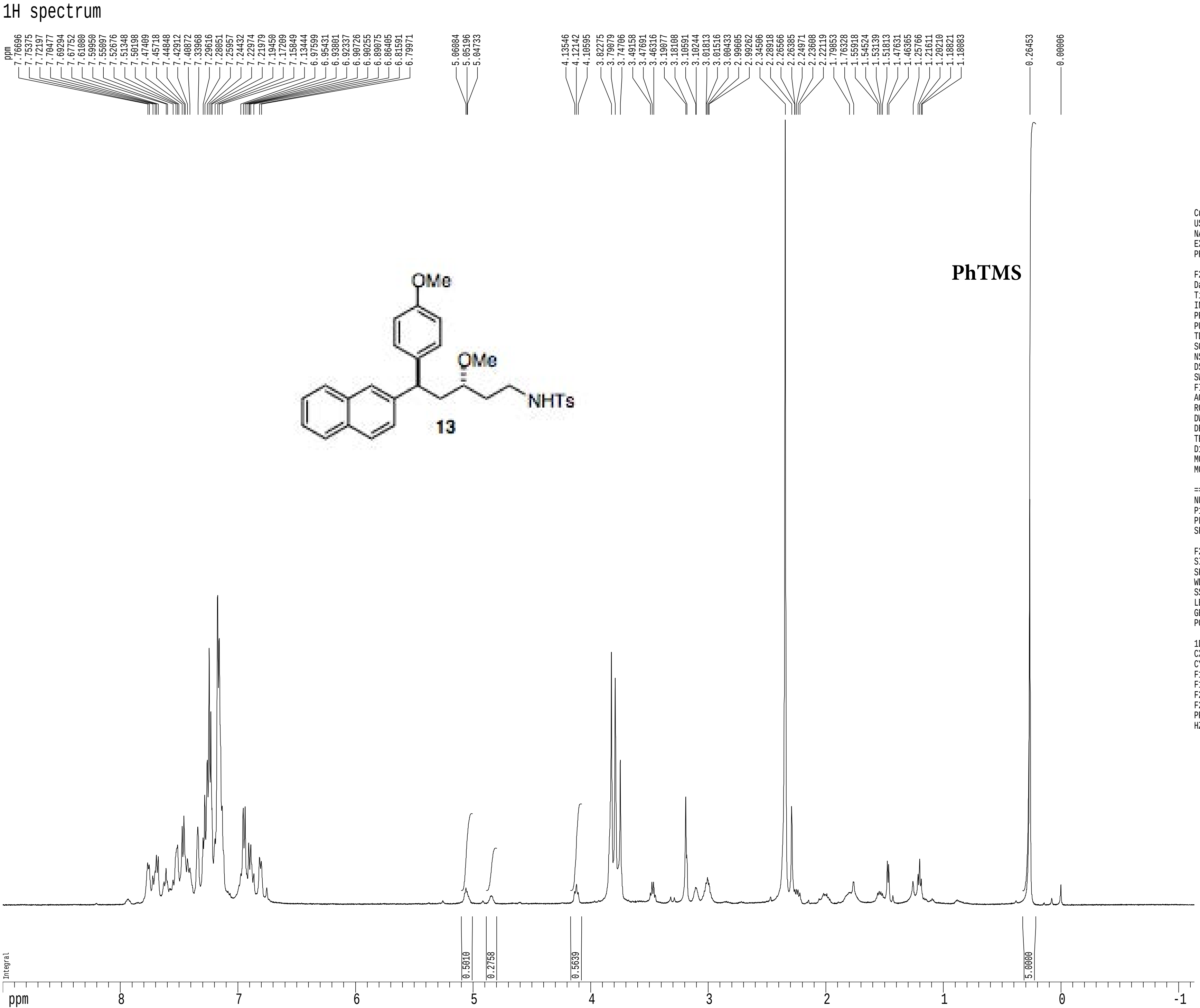


```
Current Data Parameters
USER            caherber
NAME     CAH-I-284-Crude
EXPNO                  1
PROCNO                 1

F2 - Acquisition Parameters
Date_           20210917
Time               10.29
INSTRUM            gn500
PROBHD   5 mm broadband
PULPROG             zg30
TD                 81728
SOLVENT            CDCl3
NS                     8
DS                     2
SWH             8012.820 Hz
FIDRES          0.098043 Hz
AQ             5.0998774 sec
RG                  90.5
DW                62.400 usec
DE                  6.00 usec
TE                 298.0 K
D1            0.10000000 sec
MCREST        0.00000000 sec
MCWRK         0.01500000 sec

======== CHANNEL f1 ========
NUC1                  1H
P1                 12.00 usec
PL1                -6.00 dB
SFO1         498.6534906 MHz

F2 - Processing parameters
SI                 65536
SF           498.6500485 MHz
WDW                   no
SSB                    0
LB                  0.00 Hz
GB                     0
PC                  1.00

1D NMR plot parameters
CX                 22.80 cm
CY                 15.00 cm
F1P                9.000 ppm
F1               4487.85 Hz
F2P               -1.132 ppm
F2               -564.38 Hz
PPMCM            0.44438 ppm/cm
HZCM           221.58908 Hz/cm
```

1H

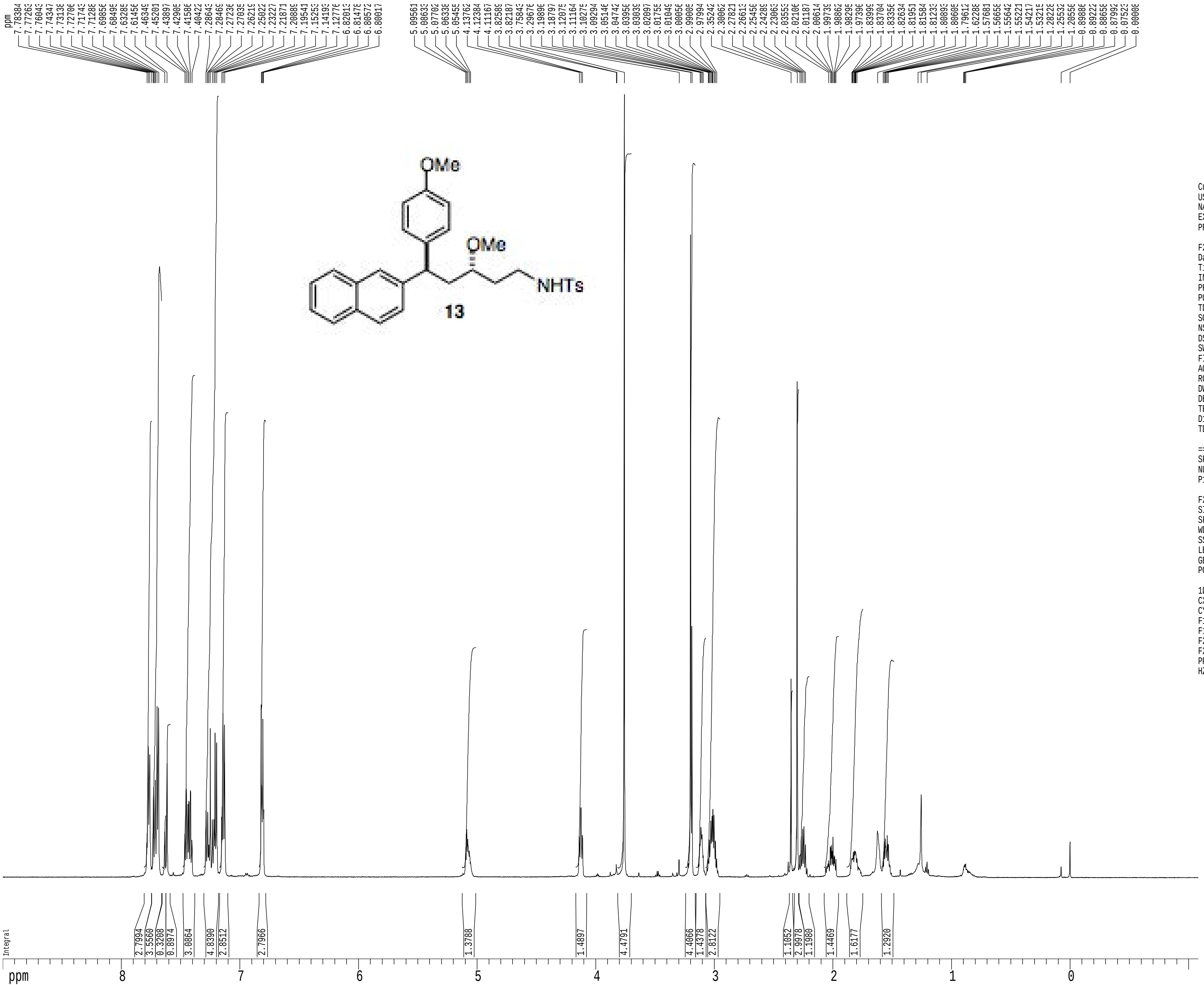

Current Data Parameters
USER caherber
NAME CAH-I-285-fll-600
EXPNO 4
PROCNO 1

F2 - Acquisition Parameters
Date_ 20210920
Time 10.59
INSTRUM av600
PROBHD 5 mm CPBBO BB-
PULPROG zg30
TD 98074
SOLVENT CDCl3
NS 8
DS 2
SWH 9615.385 Hz
FIDRES 0.098042 Hz
AQ 5.0998979 sec
RG 10
DW 52.000 usec
DE 14.23 usec
TE 298.0 K
D1 0.10000000 sec
TD0 1

======== CHANNEL f1 ========
SFO1 600.1342009 MHz
NUC1 1H
P1 9.50 usec

F2 - Processing parameters
SI 65536
SF 600.1300407 MHz
WDW no
SSB 0
LB 0.00 Hz
GB 0
PC 1.00

1D NMR plot parameters
CX 22.80 cm
CY 15.00 cm
F1P 9.000 ppm
F1 5401.17 Hz
F2P -1.079 ppm
F2 -647.47 Hz
PPMCM 0.44206 ppm/cm
HZCM 265.29141 Hz/cm

13C

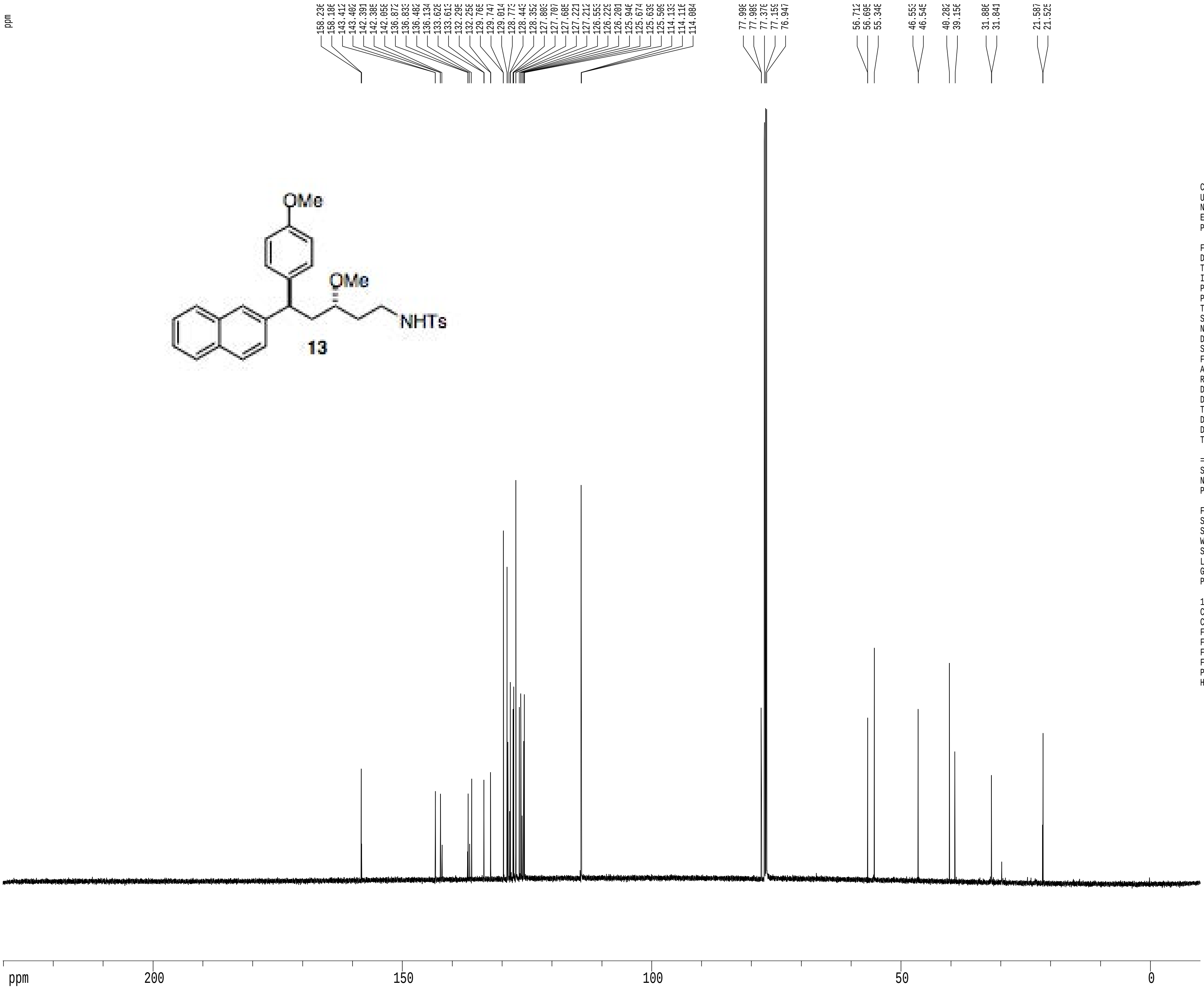

13

```
Current Data Parameters
USER            caherber
NAME      CAH-I-285-fll-600
EXPNO                  5
PROCNO                 1

F2 - Acquisition Parameters
Date_          20210920
Time              11.09
INSTRUM           av600
PROBHD   5 mm CPBBO BB-
PULPROG         zgdc30
TD                65536
SOLVENT           CDCl3
NS                  307
DS                    4
SWH           36231.883 Hz
FIDRES         0.552855 Hz
AQ            0.9044468 sec
RG                 2050
DW               13.800 usec
DE                19.63 usec
TE                298.1 K
D1           0.40000001 sec
D11          0.03000000 sec
TD0                   1

======== CHANNEL f1 ========
SFO1        150.9194080 MHz
NUC1                13C
P1                10.10 usec

F2 - Processing parameters
SI                65536
SF          150.9027986 MHz
WDW                  no
SSB                   0
LB                 0.00 Hz
GB                    0
PC                 1.00

1D NMR plot parameters
CX                22.80 cm
CY                15.00 cm
F1P             230.117 ppm
F1             34725.34 Hz
F2P              -9.984 ppm
F2             -1506.54 Hz
PPMCM          10.53074 ppm/cm
HZCM         1589.11780 Hz/cm
```

1H spectrum

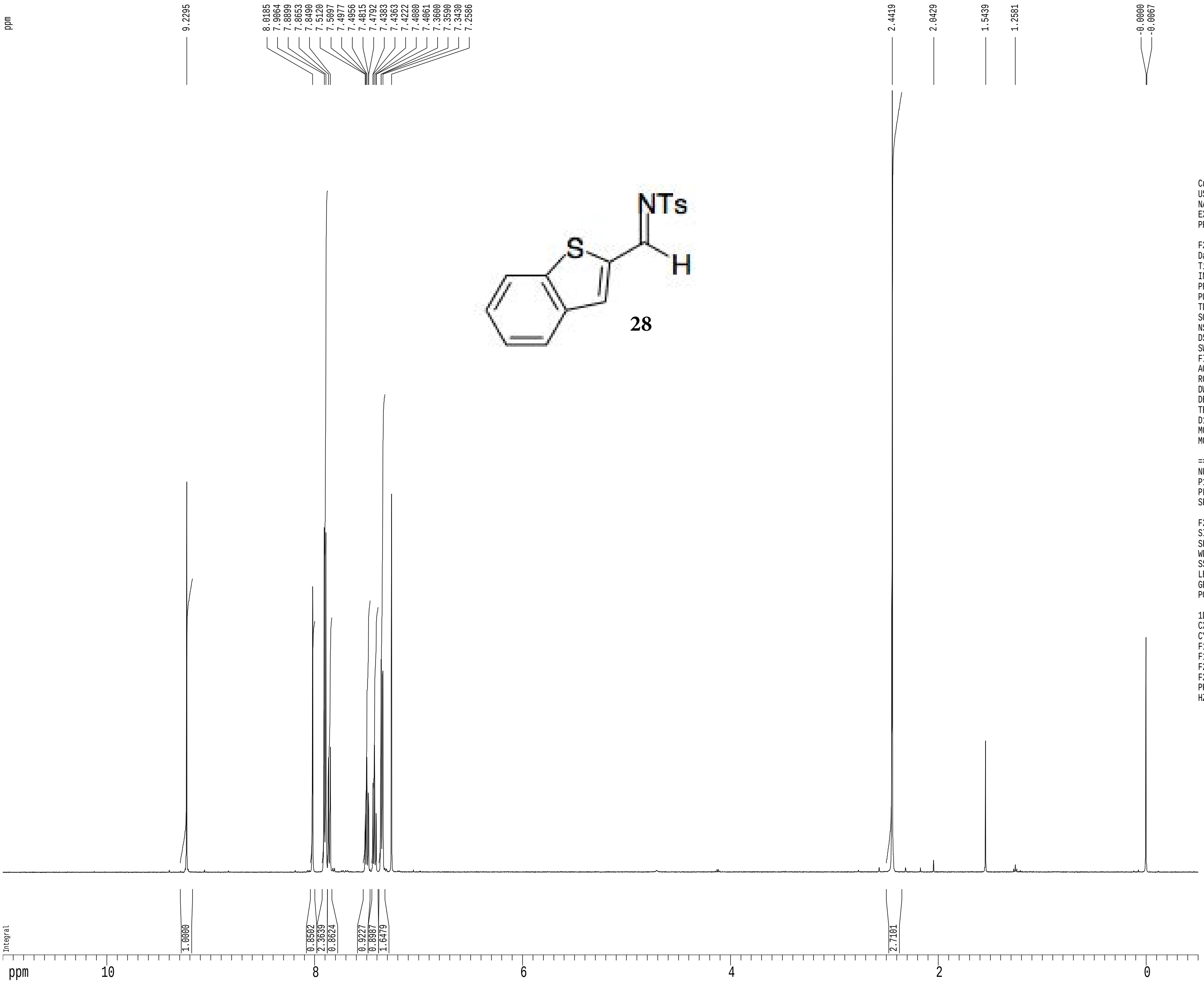


```
Current Data Parameters
USER            khewitt1
NAME        KAH-I-153-Z-2
EXPNO                  1
PROCNO                 1

F2 - Acquisition Parameters
Date_           20181110
Time               17.24
INSTRUM          cryo500
PROBHD   5 mm CPTCI 1H-
PULPROG             zg30
TD                 48074
SOLVENT           CDCl3T
NS                     8
DS                     2
SWH            8012.820 Hz
FIDRES         0.166677 Hz
AQ            2.9998677 sec
RG                  10.1
DW                62.400 usec
DE                  6.00 usec
TE                 298.0 K
D1            0.10000000 sec
MCREST        0.00000000 sec
MCWRK         0.01500000 sec

======== CHANNEL f1 ========
NUC1                  1H
P1                  7.50 usec
PL1                 1.60 dB
SFO1         500.2235015 MHz

F2 - Processing parameters
SI                 65536
SF           500.2200329 MHz
WDW                   no
SSB                    0
LB                  0.00 Hz
GB                     0
PC                  1.00

1D NMR plot parameters
CX                 22.80 cm
CY                 15.00 cm
F1P               11.000 ppm
F1               5502.42 Hz
F2P               -0.500 ppm
F2               -250.11 Hz
PPMCM            0.50439 ppm/cm
HZCM           252.30397 Hz/cm
```

Z-restored spin-echo 13C spectrum with 1H decoupling

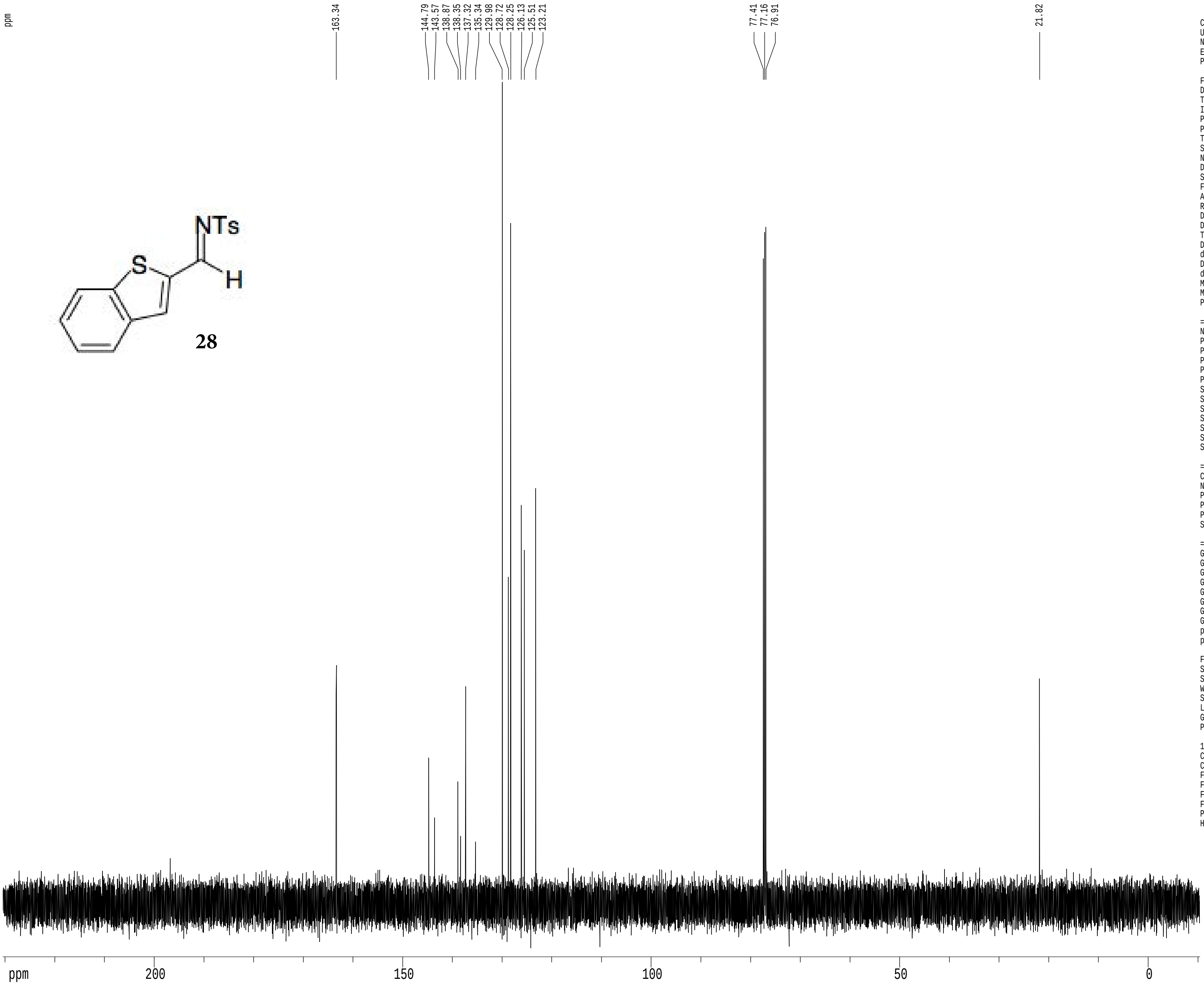


```
Current Data Parameters
USER            khewitt1
NAME       KAH-I-153-Z-2
EXPNO                  2
PROCNO                 1

F2 - Acquisition Parameters
Date_           20181110
Time               17.26
INSTRUM          cryo500
PROBHD   5 mm CPTCI 1H-
PULPROG  SpinEchopg30gp2.prd
TD                 65536
SOLVENT            CDCl3
NS                   154
DS                    16
SWH            30303.031 Hz
FIDRES          0.462388 Hz
AQ             1.0813940 sec
RG                 13004
DW                16.500 usec
DE                  6.00 usec
TE                 298.0 K
D1            0.25000000 sec
d11           0.03000000 sec
D16           0.00020000 sec
d17           0.00019600 sec
MCREST        0.00000000 sec
MCWRK         0.01500000 sec
P2                 33.10 usec

======== CHANNEL f1 ========
NUC1                 13C
P1                 16.55 usec
P12              2000.00 usec
P20               500.00 usec
PL0               120.00 dB
PL1                -1.00 dB
SFO1         125.7942548 MHz
SP2                 2.70 dB
SP4                 2.70 dB
SPNAM2       Crp60comp.4
SPNAM4    Crp60,0.5,20.1
SPOFF2              0.00 Hz
SPOFF4              0.00 Hz

======== CHANNEL f2 ========
CPDPRG2          waltz16
NUC2                  1H
PCPD2             100.00 usec
PL2                 1.60 dB
PL12               23.54 dB
SFO2         500.2225011 MHz

====== GRADIENT CHANNEL =====
GPNAM1          SINE.100
GPNAM2          SINE.100
GPX1                0.00 %
GPX2                0.00 %
GPY1                0.00 %
GPY2                0.00 %
GPZ1               30.00 %
GPZ2               50.00 %
p15               500.00 usec
p16              1000.00 usec

F2 - Processing parameters
SI                 65536
SF           125.7804071 MHz
WDW                   no
SSB                    0
LB                  0.00 Hz
GB                     0
PC                  1.50

1D NMR plot parameters
CX                 22.80 cm
CY                 15.65 cm
F1P              230.460 ppm
F1              28987.37 Hz
F2P              -10.460 ppm
F2              -1315.66 Hz
PPMCM           10.56667 ppm/cm
HZCM          1329.08020 Hz/cm
```

1H spectrum

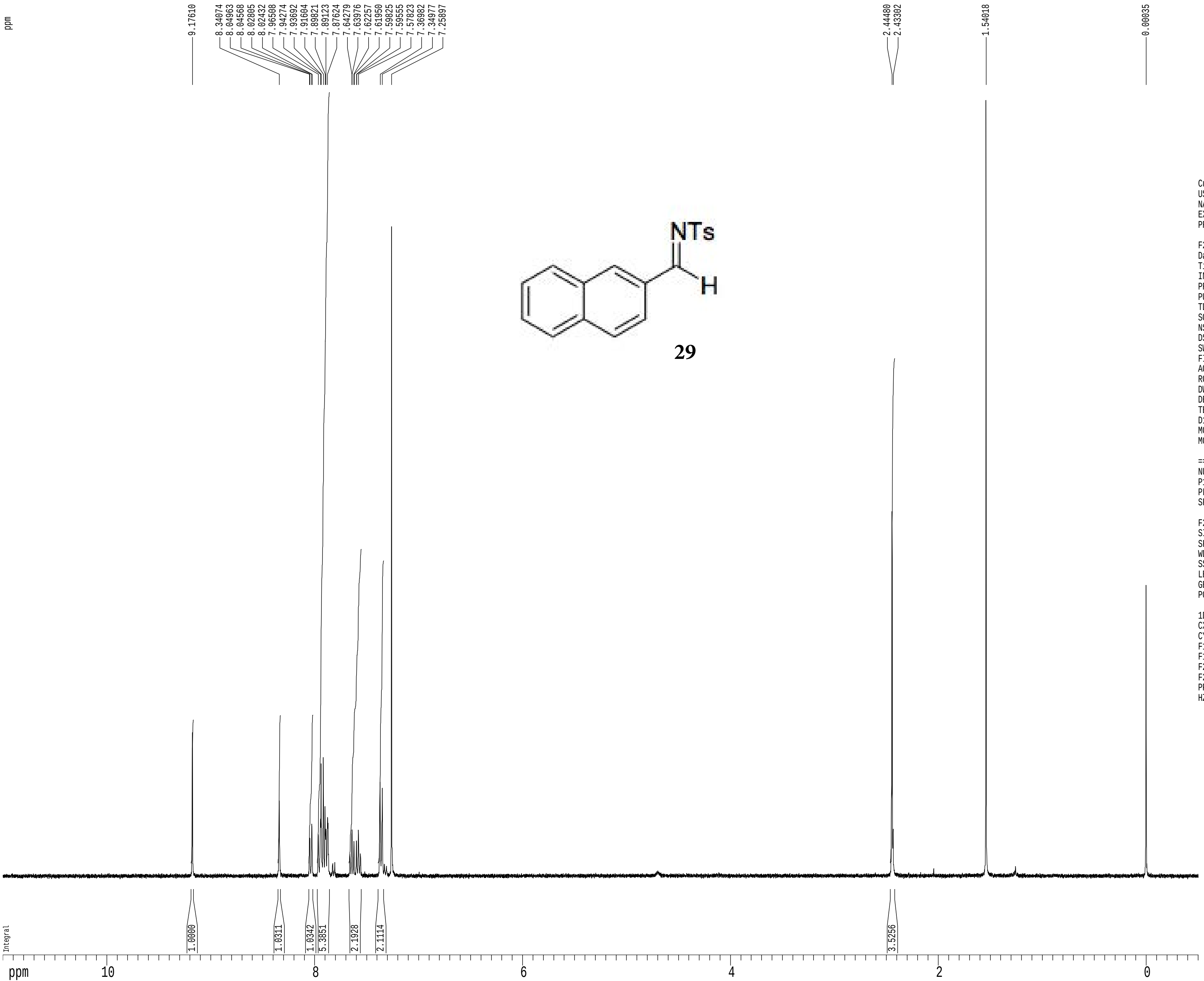

29

Current Data Parameters
USER khewitt1
NAME CAH-I-219
EXPNO 2
PROCNO 1

F2 - Acquisition Parameters
Date_ 20210506
Time 9.54
INSTRUM drx400
PROBHD 5 mm QNP H/F/P
PULPROG zg30
TD 65536
SOLVENT CDCl3
NS 8
DS 2
SWH 6410.256 Hz
FIDRES 0.097813 Hz
AQ 5.1118579 sec
RG 1149.4
DW 78.000 usec
DE 4.50 usec
TE 298.0 K
D1 0.10000000 sec
MCREST 0.00000000 sec
MCWRK 0.01500000 sec

======== CHANNEL f1 ========
NUC1 1H
P1 12.00 usec
PL1 -1.60 dB
SFO1 400.1328009 MHz

F2 - Processing parameters
SI 65536
SF 400.1300220 MHz
WDW no
SSB 0
LB 0.00 Hz
GB 0
PC 2.00

1D NMR plot parameters
CX 22.80 cm
CY 15.00 cm
F1P 11.000 ppm
F1 4401.43 Hz
F2P -0.500 ppm
F2 -200.07 Hz
PPMCM 0.50439 ppm/cm
HZCM 201.81998 Hz/cm

1H spectrum

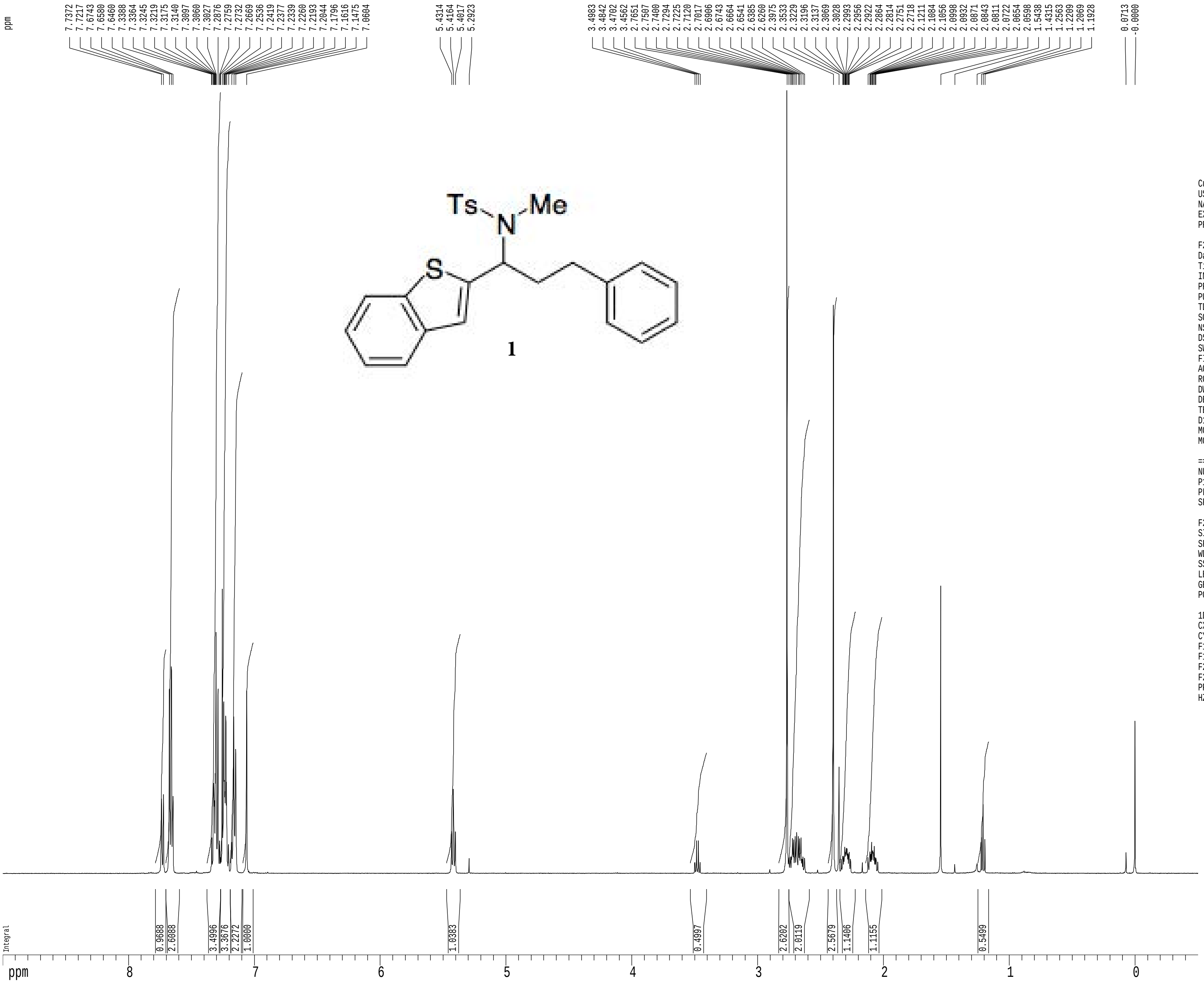


```
Current Data Parameters
USER          khewitt1
NAME        KAH-I-172-Z
EXPNO              1
PROCNO             1

F2 - Acquisition Parameters
Date_          20181107
Time              21.08
INSTRUM         cryo500
PROBHD   5 mm CPTCI 1H-
PULPROG            zg30
TD                48074
SOLVENT          CDCl3T
NS                    8
DS                    2
SWH            8012.820 Hz
FIDRES         0.166677 Hz
AQ            2.9998677 sec
RG                    9
DW               62.400 usec
DE                 6.00 usec
TE                298.0 K
D1           0.10000000 sec
MCREST       0.00000000 sec
MCWRK        0.01500000 sec

======== CHANNEL f1 ========
NUC1                 1H
P1                 7.50 usec
PL1                1.60 dB
SFO1        500.2235015 MHz

F2 - Processing parameters
SI                65536
SF          500.2200355 MHz
WDW                  no
SSB                   0
LB                 0.00 Hz
GB                    0
PC                 1.00

1D NMR plot parameters
CX                22.80 cm
CY                15.00 cm
F1P               9.000 ppm
F1              4501.98 Hz
F2P              -0.500 ppm
F2              -250.11 Hz
PPMCM           0.41667 ppm/cm
HZCM          208.42502 Hz/cm
```

Z-restored spin-echo 13C spectrum with 1H decoupling

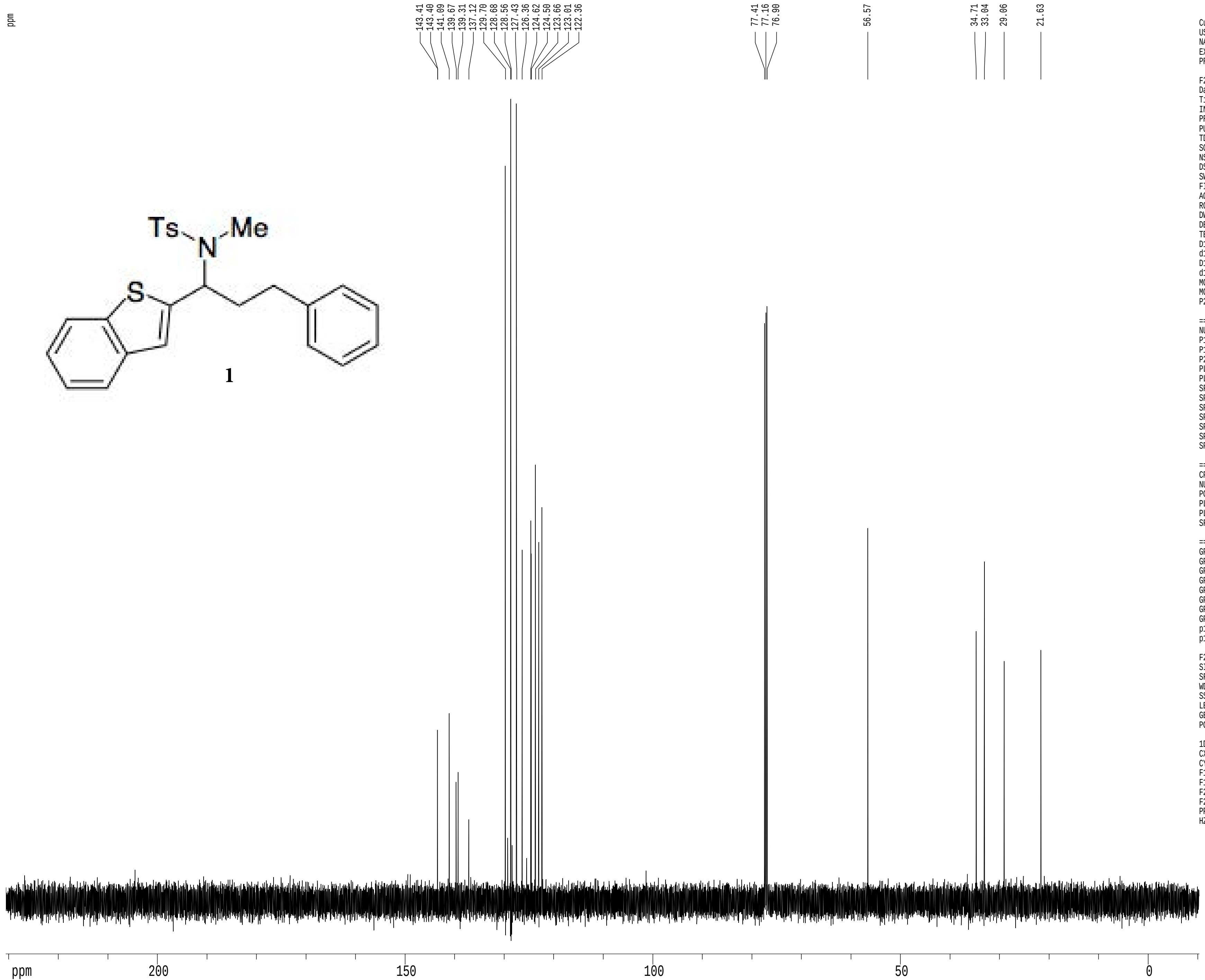

1

```
Current Data Parameters
USER          khewitt1
NAME        KAH-I-172-Z
EXPNO                2
PROCNO               1

F2 - Acquisition Parameters
Date_         20181107
Time             21.12
INSTRUM        cryo500
PROBHD   5 mm CPTCI 1H-
PULPROG  SpinEchopg30gp2.prd
TD               65536
SOLVENT          CDCl3
NS                 196
DS                  16
SWH          30303.031 Hz
FIDRES        0.462388 Hz
AQ           1.0813940 sec
RG              2896.3
DW              16.500 usec
DE                6.00 usec
TE               298.0 K
D1          0.25000000 sec
d11         0.03000000 sec
D16         0.00020000 sec
d17         0.00019600 sec
MCREST      0.00000000 sec
MCWRK       0.01500000 sec
P2               33.10 usec

======== CHANNEL f1 ========
NUC1               13C
P1               16.55 usec
P12            2000.00 usec
P20             500.00 usec
PL0             120.00 dB
PL1              -1.00 dB
SFO1       125.7942548 MHz
SP2               2.70 dB
SP4               2.70 dB
SPNAM2     Crp60comp.4
SPNAM4   Crp60,0.5,20.1
SPOFF2            0.00 Hz
SPOFF4            0.00 Hz

======== CHANNEL f2 ========
CPDPRG2         waltz16
NUC2                 1H
PCPD2           100.00 usec
PL2               1.60 dB
PL12             23.54 dB
SFO2       500.2225011 MHz

====== GRADIENT CHANNEL =====
GPNAM1         SINE.100
GPNAM2         SINE.100
GPX1              0.00 %
GPX2              0.00 %
GPY1              0.00 %
GPY2              0.00 %
GPZ1             30.00 %
GPZ2             50.00 %
p15             500.00 usec
p16            1000.00 usec

F2 - Processing parameters
SI                65536
SF          125.7804080 MHz
WDW                  no
SSB                   0
LB                 0.00 Hz
GB                    0
PC                 2.50

1D NMR plot parameters
CX                22.80 cm
CY                15.65 cm
F1P             230.637 ppm
F1             29009.68 Hz
F2P             -10.287 ppm
F2             -1293.96 Hz
PPMCM          10.56688 ppm/cm
HZCM         1329.10693 Hz/cm
```

1H spectrum

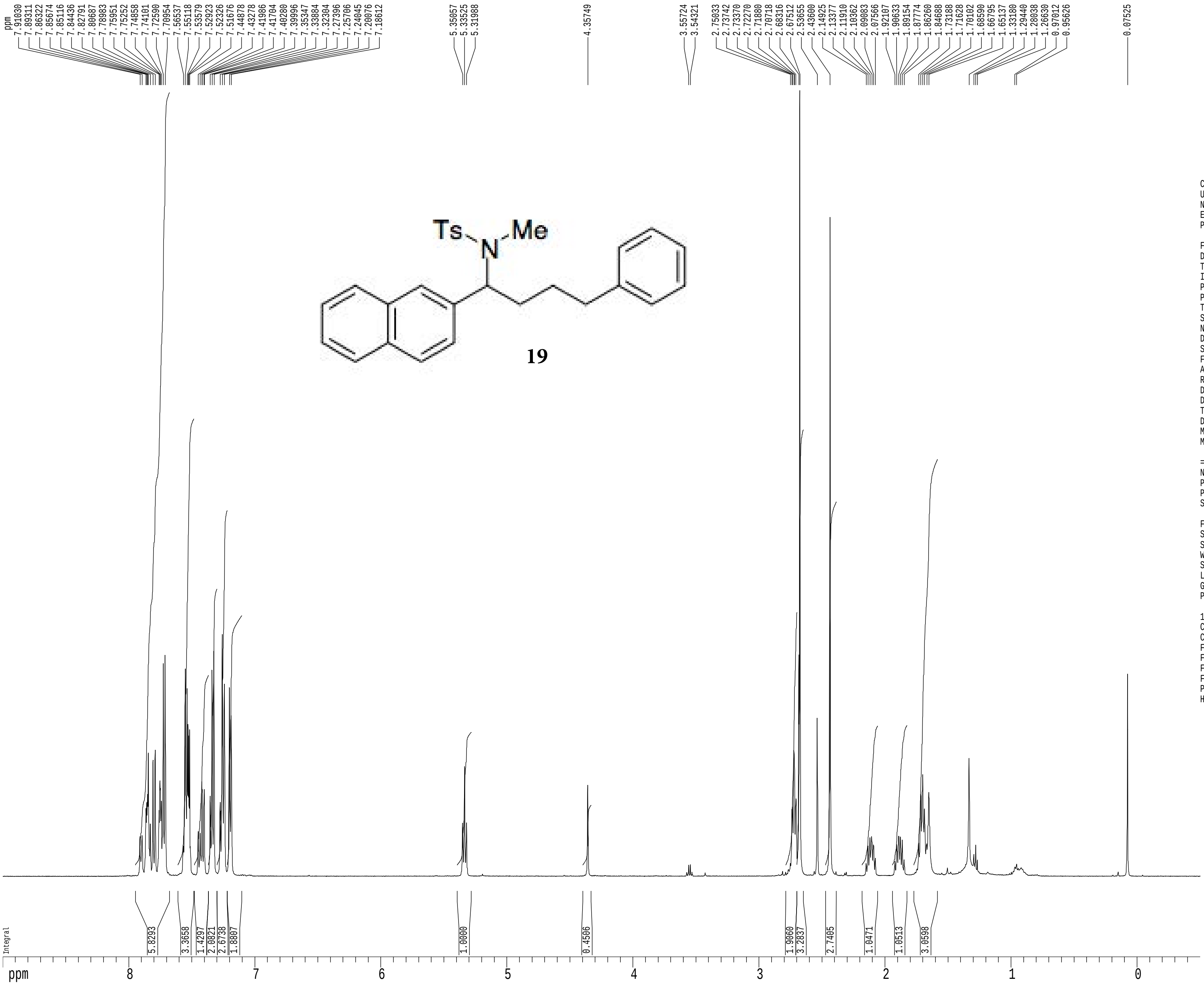

Current Data Parameters
USER khewitt1
NAME AM-I-046-Z
EXPNO 1
PROCNO 1

F2 - Acquisition Parameters
Date_ 20210809
Time 18.13
INSTRUM cryo500
PROBHD 5 mm CPTCI 1H-
PULPROG zg30
TD 48074
SOLVENT CDCl3T
NS 8
DS 2
SWH 8012.820 Hz
FIDRES 0.166677 Hz
AQ 2.9998677 sec
RG 5
DW 62.400 usec
DE 6.00 usec
TE 298.0 K
D1 0.10000000 sec
MCREST 0.00000000 sec
MCWRK 0.01500000 sec

======== CHANNEL f1 ========
NUC1 1H
P1 9.75 usec
PL1 1.60 dB
SFO1 500.2235015 MHz

F2 - Processing parameters
SI 65536
SF 500.2200000 MHz
WDW no
SSB 0
LB 0.00 Hz
GB 0
PC 1.00

1D NMR plot parameters
CX 22.80 cm
CY 15.00 cm
F1P 9.000 ppm
F1 4501.98 Hz
F2P -0.500 ppm
F2 -250.11 Hz
PPMCM 0.41667 ppm/cm
HZCM 208.42500 Hz/cm

Z-restored spin-echo 13C spectrum with 1H decoupling

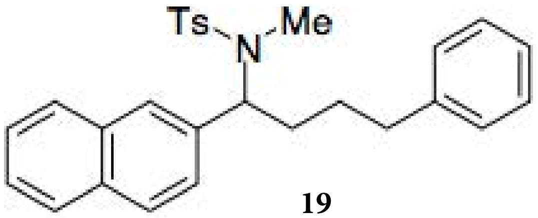

19

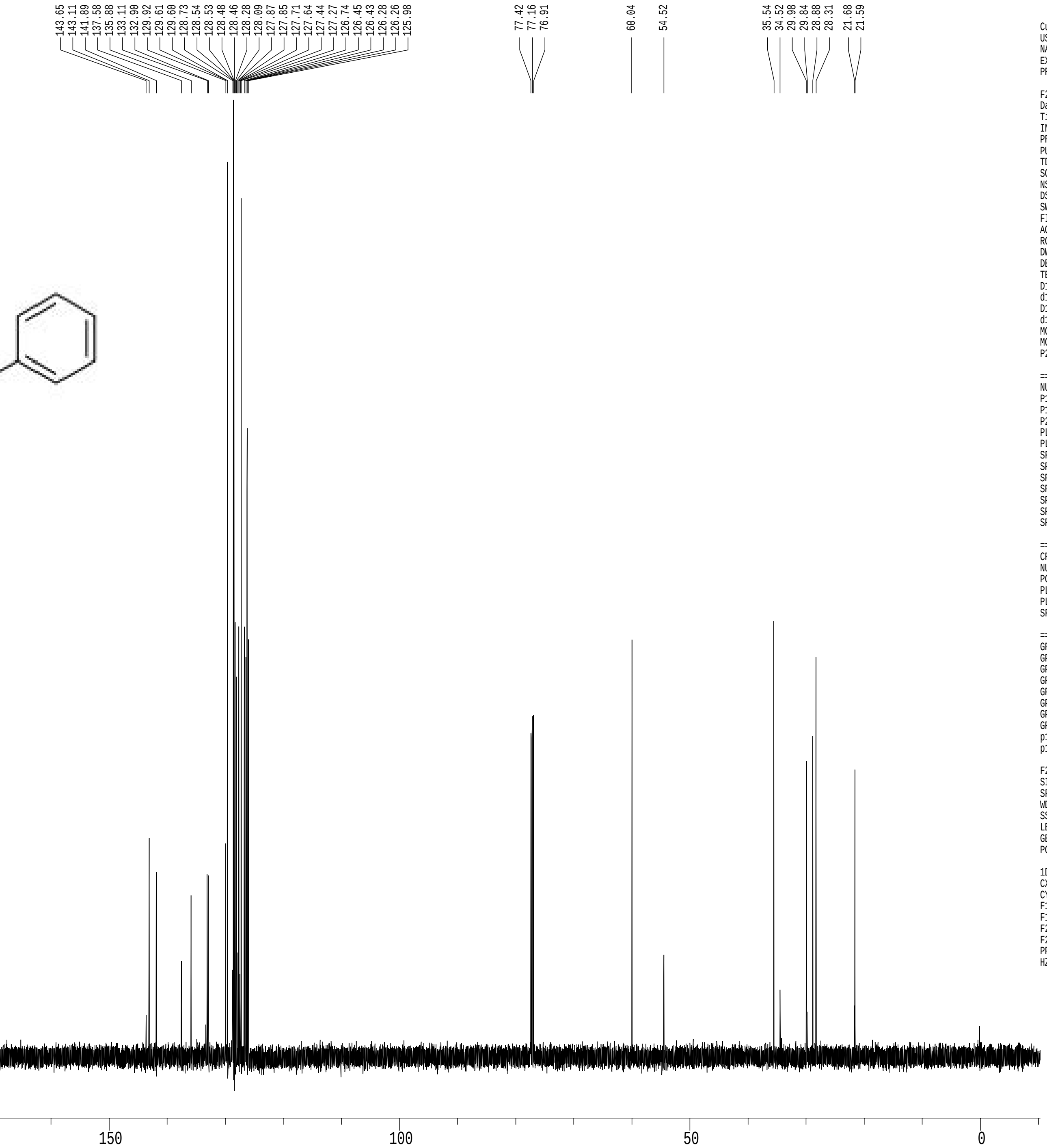


```
Current Data Parameters
USER          khewitt1
NAME          AM-I-046-Z
EXPNO                2
PROCNO               1

F2 - Acquisition Parameters
Date_          20210809
Time              18.16
INSTRUM         cryo500
PROBHD   5 mm CPTCI 1H-
PULPROG  SpinEchopg30gp2.prd
TD                65536
SOLVENT           CDCl3
NS                  152
DS                   16
SWH          30303.031 Hz
FIDRES        0.462388 Hz
AQ           1.0813940 sec
RG               7298.2
DW               16.500 usec
DE                 6.00 usec
TE                298.0 K
D1          0.25000000 sec
d11         0.03000000 sec
D16         0.00020000 sec
d17         0.00019600 sec
MCREST      0.00000000 sec
MCWRK       0.01500000 sec
P2                37.70 usec

======== CHANNEL f1 ========
NUC1                13C
P1                18.85 usec
P12             2000.00 usec
P20              500.00 usec
PL0              120.00 dB
PL1               -1.00 dB
SFO1        125.7942548 MHz
SP2                1.55 dB
SP4                1.55 dB
SPNAM2      Crp60comp.4
SPNAM4   Crp60,0.5,20.1
SPOFF2             0.00 Hz
SPOFF4             0.00 Hz

======== CHANNEL f2 ========
CPDPRG2         waltz16
NUC2                 1H
PCPD2            100.00 usec
PL2                1.60 dB
PL12              22.00 dB
SFO2        500.2225011 MHz

====== GRADIENT CHANNEL =====
GPNAM1         SINE.100
GPNAM2         SINE.100
GPX1               0.00 %
GPX2               0.00 %
GPY1               0.00 %
GPY2               0.00 %
GPZ1              30.00 %
GPZ2              50.00 %
p15              500.00 usec
p16             1000.00 usec

F2 - Processing parameters
SI                65536
SF          125.7804099 MHz
WDW                  no
SSB                   0
LB                 0.00 Hz
GB                    0
PC                 2.00

1D NMR plot parameters
CX                22.80 cm
CY                15.65 cm
F1P             230.637 ppm
F1             29009.68 Hz
F2P             -10.287 ppm
F2             -1293.96 Hz
PPMCM          10.56688 ppm/cm
HZCM         1329.10693 Hz/cm
```

1H spectrum

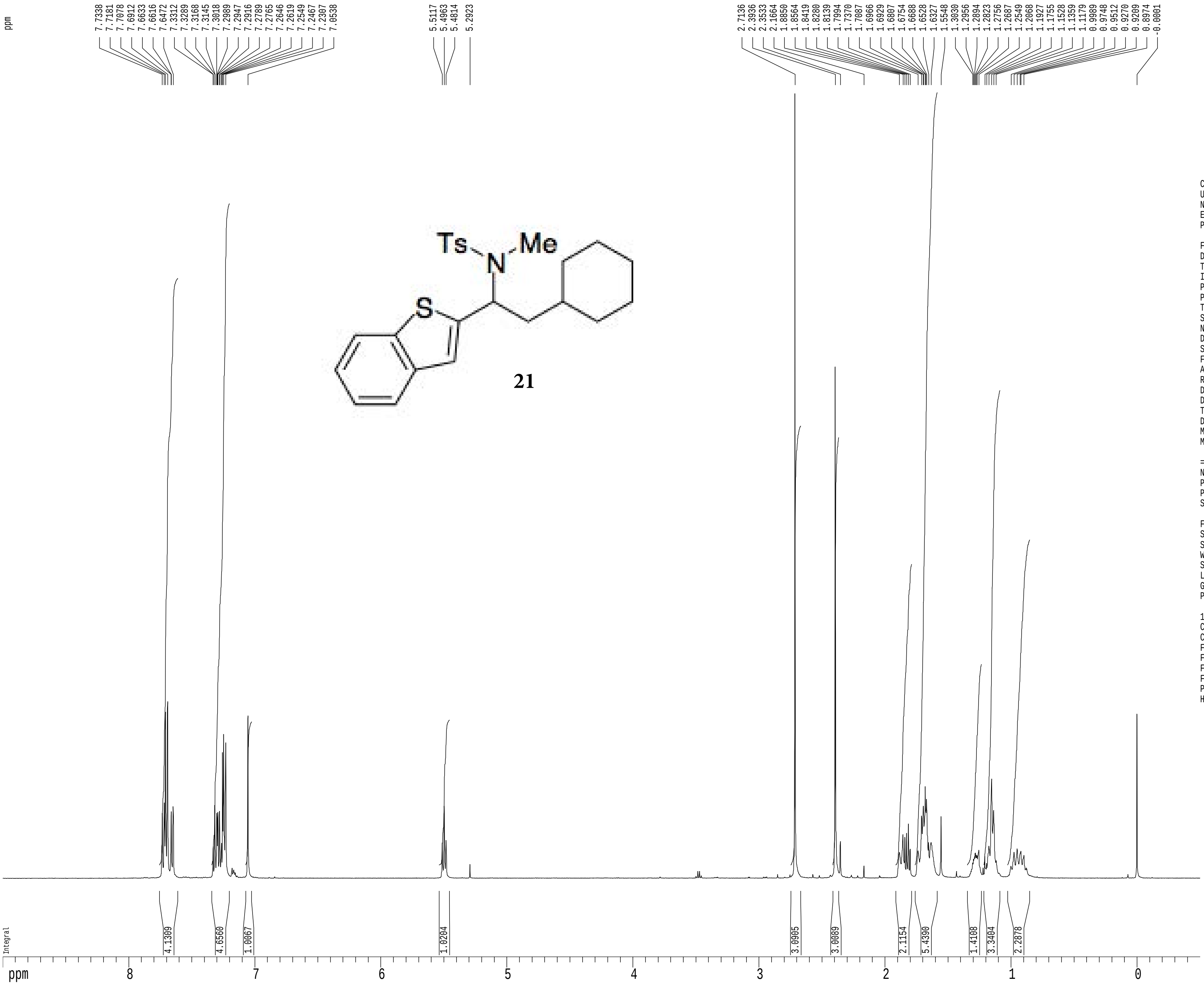

Current Data Parameters
USER khewitt1
NAME ACM-I-120-benzCH
EXPNO 1
PROCNO 1

F2 - Acquisition Parameters
Date_ 20191112
Time 9.06
INSTRUM gn500
PROBHD 5 mm broadband
PULPROG zg30
TD 81728
SOLVENT CDCl3T
NS 10
DS 2
SWH 8012.820 Hz
FIDRES 0.098043 Hz
AQ 5.0998774 sec
RG 181
DW 62.400 usec
DE 6.00 usec
TE 298.0 K
D1 0.10000000 sec
MCREST 0.00000000 sec
MCWRK 0.01500000 sec

======== CHANNEL f1 ========
NUC1 1H
P1 12.00 usec
PL1 -5.00 dB
SFO1 498.8534919 MHz

F2 - Processing parameters
SI 65536
SF 498.8500296 MHz
WDW EM
SSB 0
LB 0.30 Hz
GB 0
PC 1.00

1D NMR plot parameters
CX 22.80 cm
CY 15.00 cm
F1P 9.000 ppm
F1 4489.65 Hz
F2P -0.500 ppm
F2 -249.42 Hz
PPMCM 0.41667 ppm/cm
HZCM 207.85419 Hz/cm

13C spectrum with 1H decoupling

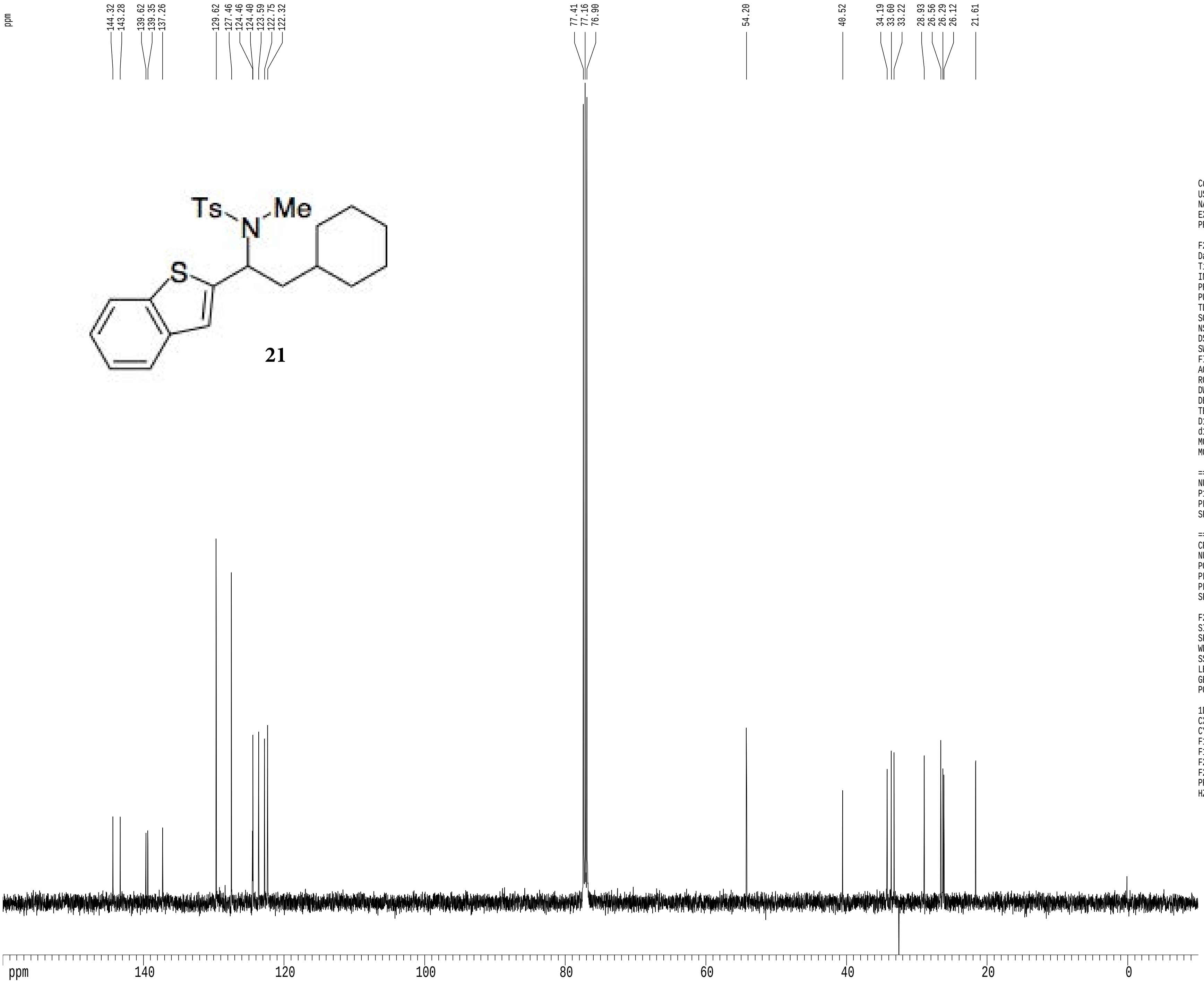

Current Data Parameters
USER khewitt1
NAME ACM-I-120-benzCH
EXPNO 2
PROCNO 1

F2 - Acquisition Parameters
Date_ 20191112
Time 9.07
INSTRUM gn500
PROBHD 5 mm broadband
PULPROG zgdc30
TD 65536
SOLVENT CDCl3
NS 544
DS 4
SWH 30303.031 Hz
FIDRES 0.462388 Hz
AQ 1.0813940 sec
RG 46341
DW 16.500 usec
DE 4.50 usec
TE 298.0 K
D1 0.25000000 sec
d11 0.03000000 sec
MCREST 0.00000000 sec
MCWRK 0.01500000 sec

======== CHANNEL f1 ========
NUC1 13C
P1 10.00 usec
PL1 -6.00 dB
SFO1 125.4497300 MHz

======== CHANNEL f2 ========
CPDPRG2 waltz16
NUC2 1H
PCPD2 80.00 usec
PL2 -5.00 dB
PL12 13.20 dB
SFO2 498.8524943 MHz

F2 - Processing parameters
SI 65536
SF 125.4359203 MHz
WDW EM
SSB 0
LB 1.00 Hz
GB 0
PC 2.00

1D NMR plot parameters
CX 22.80 cm
CY 15.65 cm
F1P 160.000 ppm
F1 20069.75 Hz
F2P -10.000 ppm
F2 -1254.36 Hz
PPMCM 7.45614 ppm/cm
HZCM 935.26788 Hz/cm

1H spectrum

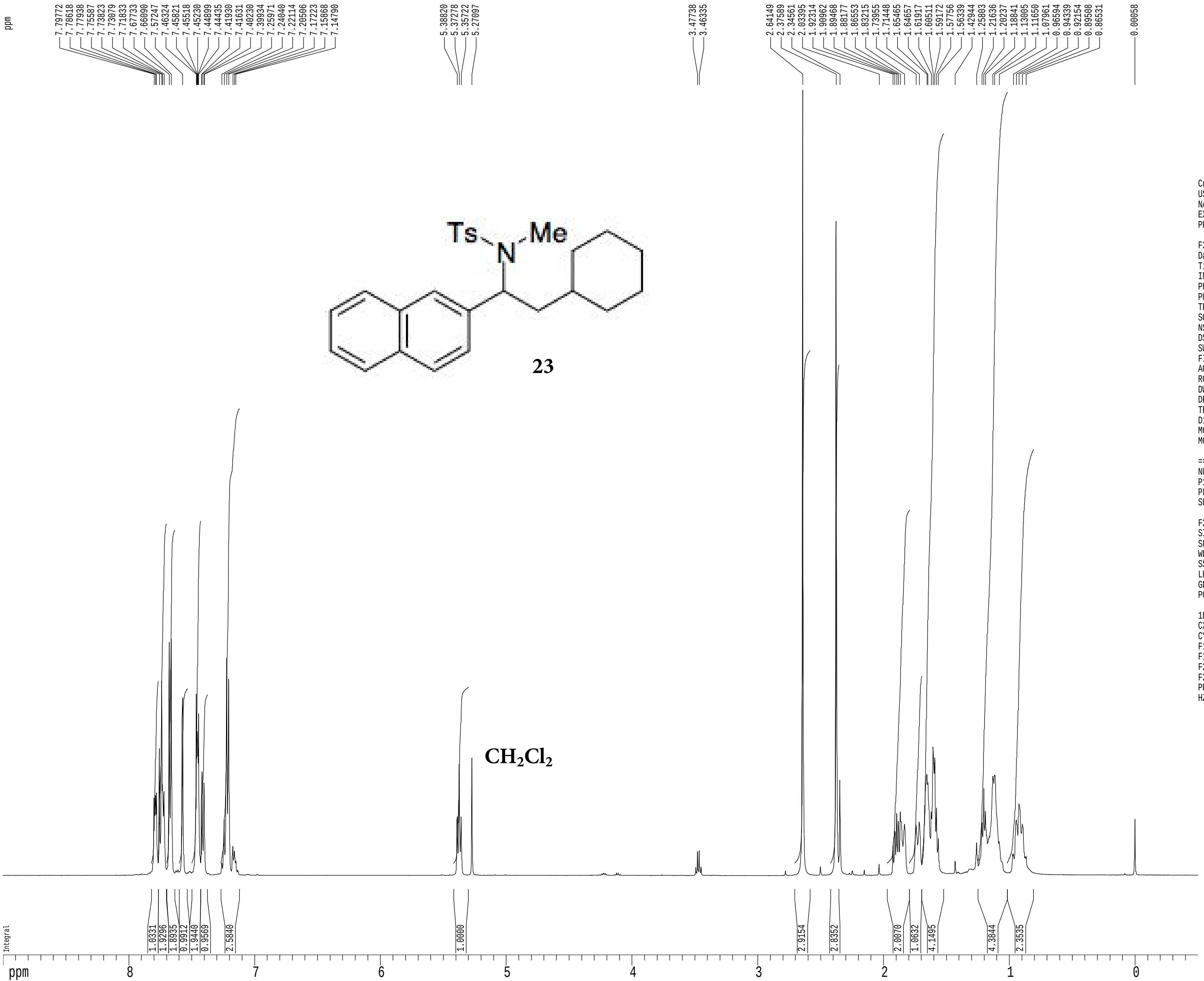

Current Data Parameters
USER khewitt1
NAME ACM-I-120nap-CH
EXPNO 1
PROCNO 1

F2 - Acquisition Parameters
Date_ 20191112
Time 9.24
INSTRUM cryo500
PROBHD 5 mm CPTCI 1H-
PULPROG zg30
TD 81728
SOLVENT CDCl3T
NS 10
DS 2
SWH 8012.820 Hz
FIDRES 0.098043 Hz
AQ 5.0998774 sec
RG 5
DW 62.400 usec
DE 6.00 usec
TE 298.2 K
D1 0.10000000 sec
MCREST 0.00000000 sec
MCWRK 0.01500000 sec

======== CHANNEL f1 ========
NUC1 1H
P1 7.50 usec
PL1 1.60 dB
SFO1 500.2235015 MHz

F2 - Processing parameters
SI 65536
SF 500.2200411 MHz
WDW EM
SSB 0
LB 0.30 Hz
GB 0
PC 1.00

1D NMR plot parameters
CX 22.80 cm
CY 15.00 cm
F1P 9.000 ppm
F1 4501.98 Hz
F2P -0.500 ppm
F2 -250.11 Hz
PPMCM 0.41667 ppm/cm
HZCM 208.42502 Hz/cm

Z-restored spin-echo 13C spectrum with 1H decoupling

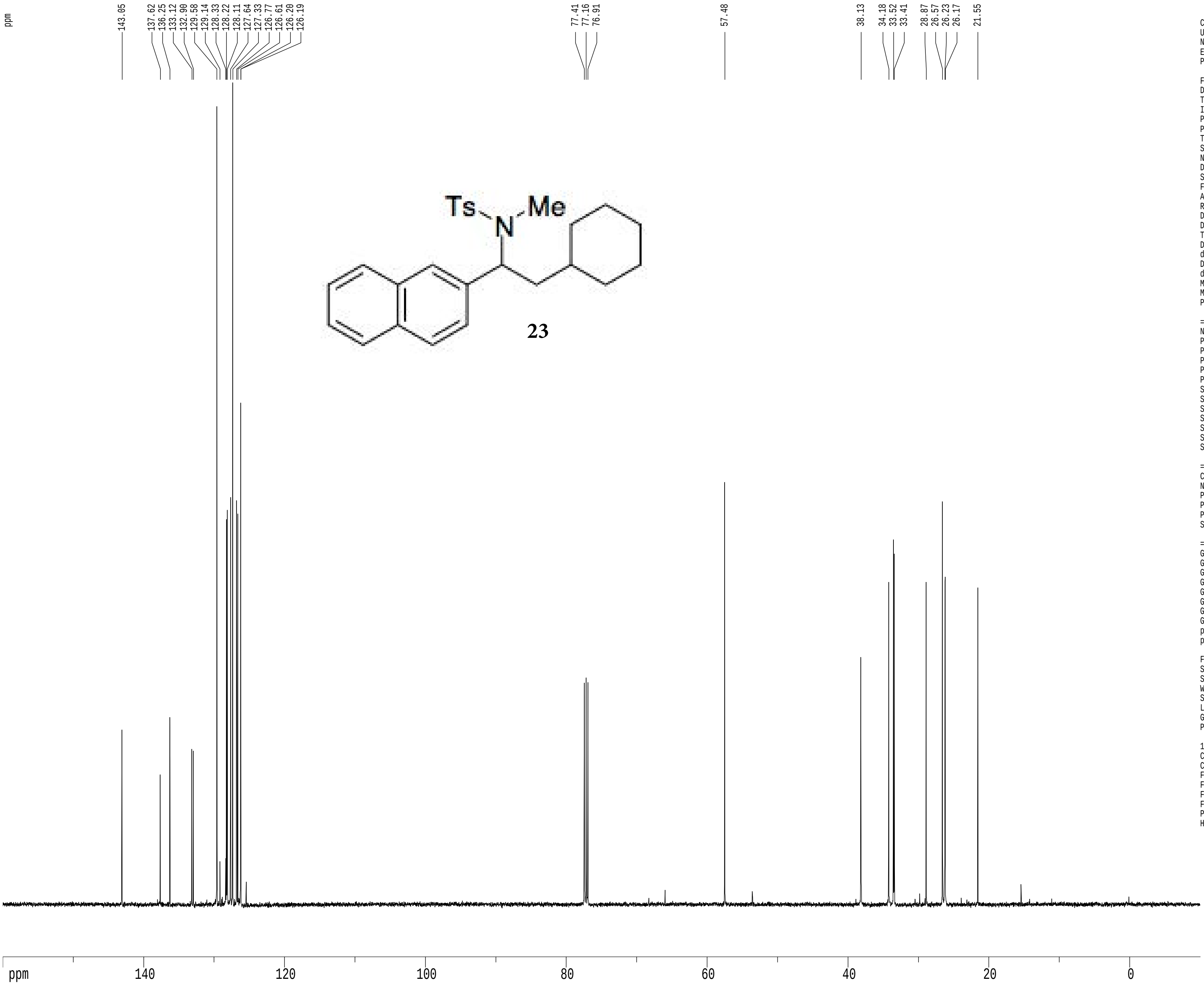

Current Data Parameters
USER khewitt1
NAME ACM-I-120nap-CH
EXPNO 2
PROCNO 1

F2 - Acquisition Parameters
Date_ 20191112
Time 9.37
INSTRUM cryo500
PROBHD 5 mm CPTCI 1H-
PULPROG SpinEchopg30gp2.prd
TD 65536
SOLVENT CDCl3
NS 504
DS 16
SWH 30303.031 Hz
FIDRES 0.462388 Hz
AQ 1.0813940 sec
RG 3251
DW 16.500 usec
DE 6.00 usec
TE 298.2 K
D1 0.25000000 sec
d11 0.03000000 sec
D16 0.00020000 sec
d17 0.00019600 sec
MCREST 0.00000000 sec
MCWRK 0.01500000 sec
P2 33.10 usec

======== CHANNEL f1 ========
NUC1 13C
P1 16.55 usec
P12 2000.00 usec
P20 500.00 usec
PL0 120.00 dB
PL1 -1.00 dB
SFO1 125.7942548 MHz
SP2 2.70 dB
SP4 2.70 dB
SPNAM2 Crp60comp.4
SPNAM4 Crp60,0.5,20.1
SPOFF2 0.00 Hz
SPOFF4 0.00 Hz

======== CHANNEL f2 ========
CPDPRG2 waltz16
NUC2 1H
PCPD2 100.00 usec
PL2 1.60 dB
PL12 23.54 dB
SFO2 500.2225011 MHz

====== GRADIENT CHANNEL =====
GPNAM1 SINE.100
GPNAM2 SINE.100
GPX1 0.00 %
GPX2 0.00 %
GPY1 0.00 %
GPY2 0.00 %
GPZ1 30.00 %
GPZ2 50.00 %
p15 500.00 usec
p16 1000.00 usec

F2 - Processing parameters
SI 65536
SF 125.7804136 MHz
WDW EM
SSB 0
LB 1.00 Hz
GB 0
PC 2.00

1D NMR plot parameters
CX 22.80 cm
CY 15.65 cm
F1P 160.000 ppm
F1 20124.87 Hz
F2P -10.000 ppm
F2 -1257.80 Hz
PPMCM 7.45614 ppm/cm
HZCM 937.83643 Hz/cm

1H spectrum

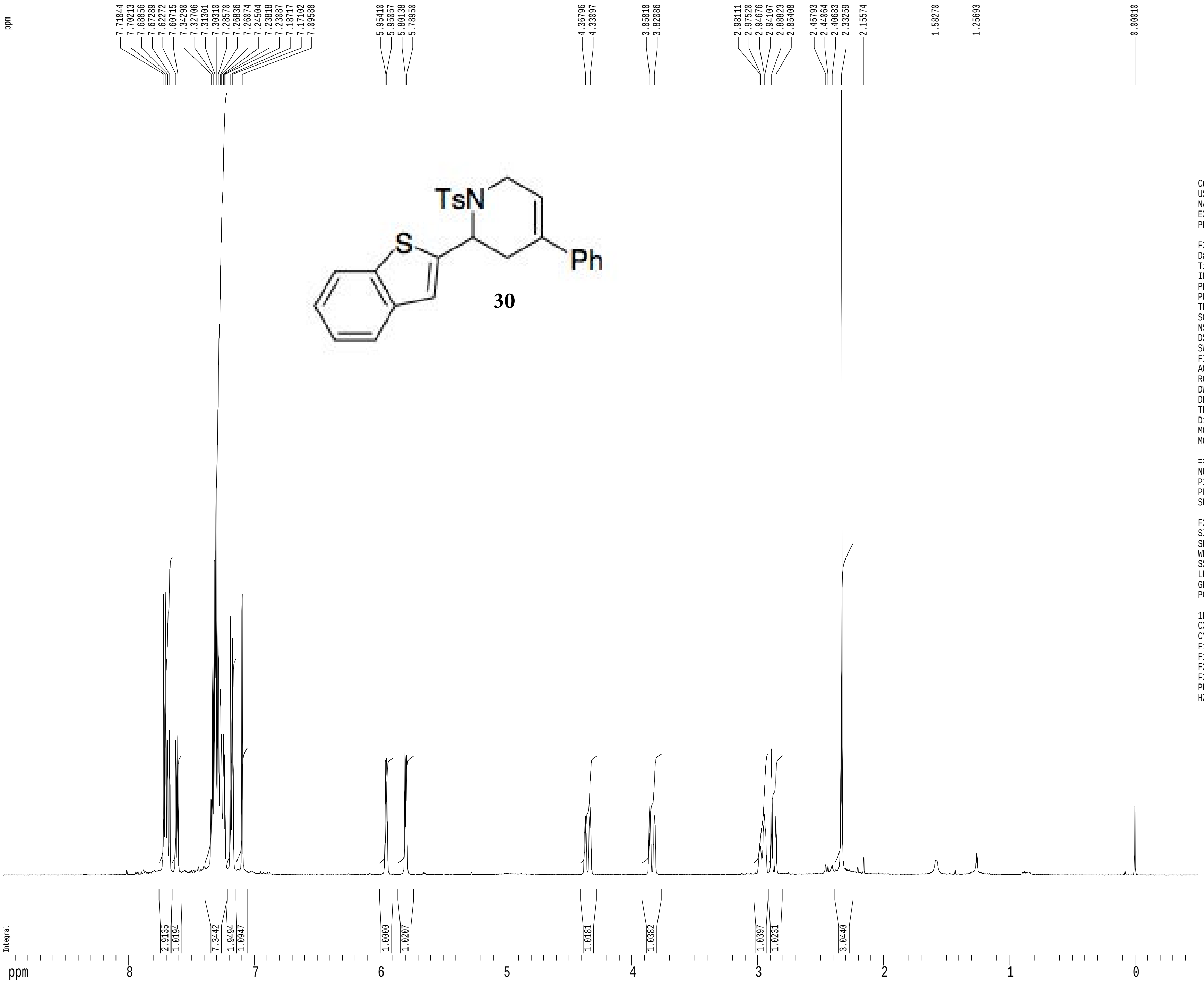

Current Data Parameters
USER khewitt1
NAME KAH-I-291-Z
EXPNO 3
PROCNO 1

F2 - Acquisition Parameters
Date_ 20190302
Time 14.24
INSTRUM cryo500
PROBHD 5 mm CPTCI 1H-
PULPROG zg30
TD 48074
SOLVENT CDCl3T
NS 8
DS 2
SWH 8012.820 Hz
FIDRES 0.166677 Hz
AQ 2.9998677 sec
RG 6.3
DW 62.400 usec
DE 6.00 usec
TE 298.0 K
D1 0.10000000 sec
MCREST 0.00000000 sec
MCWRK 0.01500000 sec

======== CHANNEL f1 ========
NUC1 1H
P1 7.50 usec
PL1 1.60 dB
SFO1 500.2235015 MHz

F2 - Processing parameters
SI 65536
SF 500.2200426 MHz
WDW no
SSB 0
LB 0.00 Hz
GB 0
PC 1.00

1D NMR plot parameters
CX 22.80 cm
CY 15.00 cm
F1P 9.000 ppm
F1 4501.98 Hz
F2P -0.500 ppm
F2 -250.11 Hz
PPMCM 0.41667 ppm/cm
HZCM 208.42502 Hz/cm

Z-restored spin-echo 13C spectrum with 1H decoupling

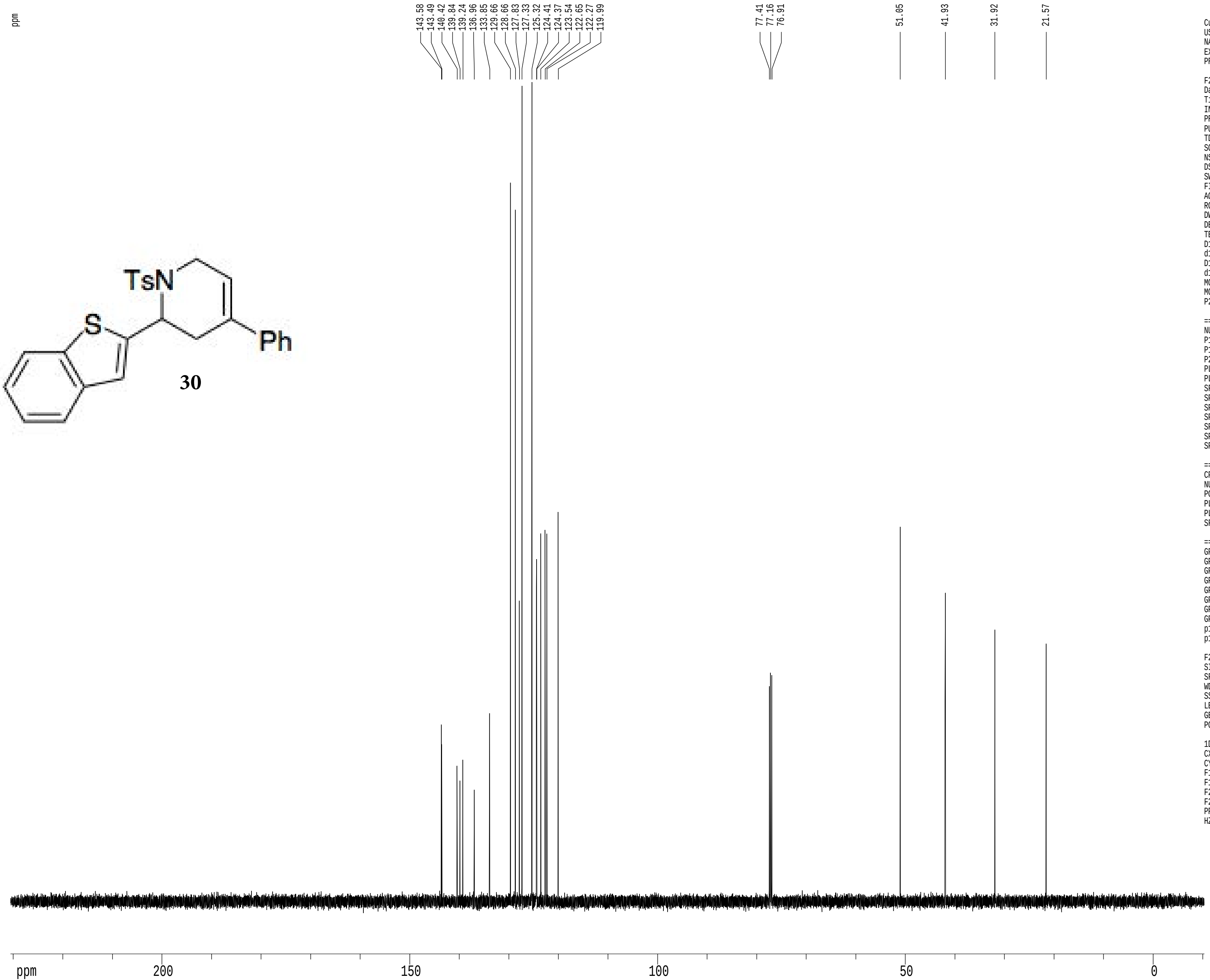

30

```
Current Data Parameters
USER            khewitt1
NAME          KAH-I-291-Z
EXPNO                  5
PROCNO                 1

F2 - Acquisition Parameters
Date_           20190302
Time               14.39
INSTRUM          cryo500
PROBHD   5 mm CPTCI 1H-
PULPROG  SpinEchopg30gp2.prd
TD                 65536
SOLVENT            CDCl3
NS                   216
DS                    16
SWH            30303.031 Hz
FIDRES          0.462388 Hz
AQ             1.0813940 sec
RG                 13004
DW                16.500 usec
DE                  6.00 usec
TE                 298.0 K
D1            0.25000000 sec
d11           0.03000000 sec
D16           0.00020000 sec
d17           0.00019600 sec
MCREST        0.00000000 sec
MCWRK         0.01500000 sec
P2                 33.10 usec

======== CHANNEL f1 ========
NUC1                 13C
P1                 16.55 usec
P12              2000.00 usec
P20               500.00 usec
PL0               120.00 dB
PL1                -1.00 dB
SFO1         125.7942548 MHz
SP2                 2.70 dB
SP4                 2.70 dB
SPNAM2       Crp60comp.4
SPNAM4    Crp60,0.5,20.1
SPOFF2              0.00 Hz
SPOFF4              0.00 Hz

======== CHANNEL f2 ========
CPDPRG2           waltz16
NUC2                   1H
PCPD2             100.00 usec
PL2                 1.60 dB
PL12               23.54 dB
SFO2         500.2225011 MHz

====== GRADIENT CHANNEL =====
GPNAM1          SINE.100
GPNAM2          SINE.100
GPX1                0.00 %
GPX2                0.00 %
GPY1                0.00 %
GPY2                0.00 %
GPZ1               30.00 %
GPZ2               50.00 %
p15               500.00 usec
p16              1000.00 usec

F2 - Processing parameters
SI                 65536
SF          125.7804136 MHz
WDW                   no
SSB                    0
LB                  0.00 Hz
GB                     0
PC                  2.00

1D NMR plot parameters
CX                 22.80 cm
CY                 15.65 cm
F1P              230.637 ppm
F1              29009.68 Hz
F2P              -10.287 ppm
F2              -1293.96 Hz
PPMCM           10.56688 ppm/cm
HZCM          1329.10693 Hz/cm
```

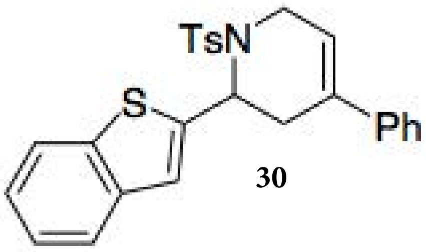

30

gcosy60

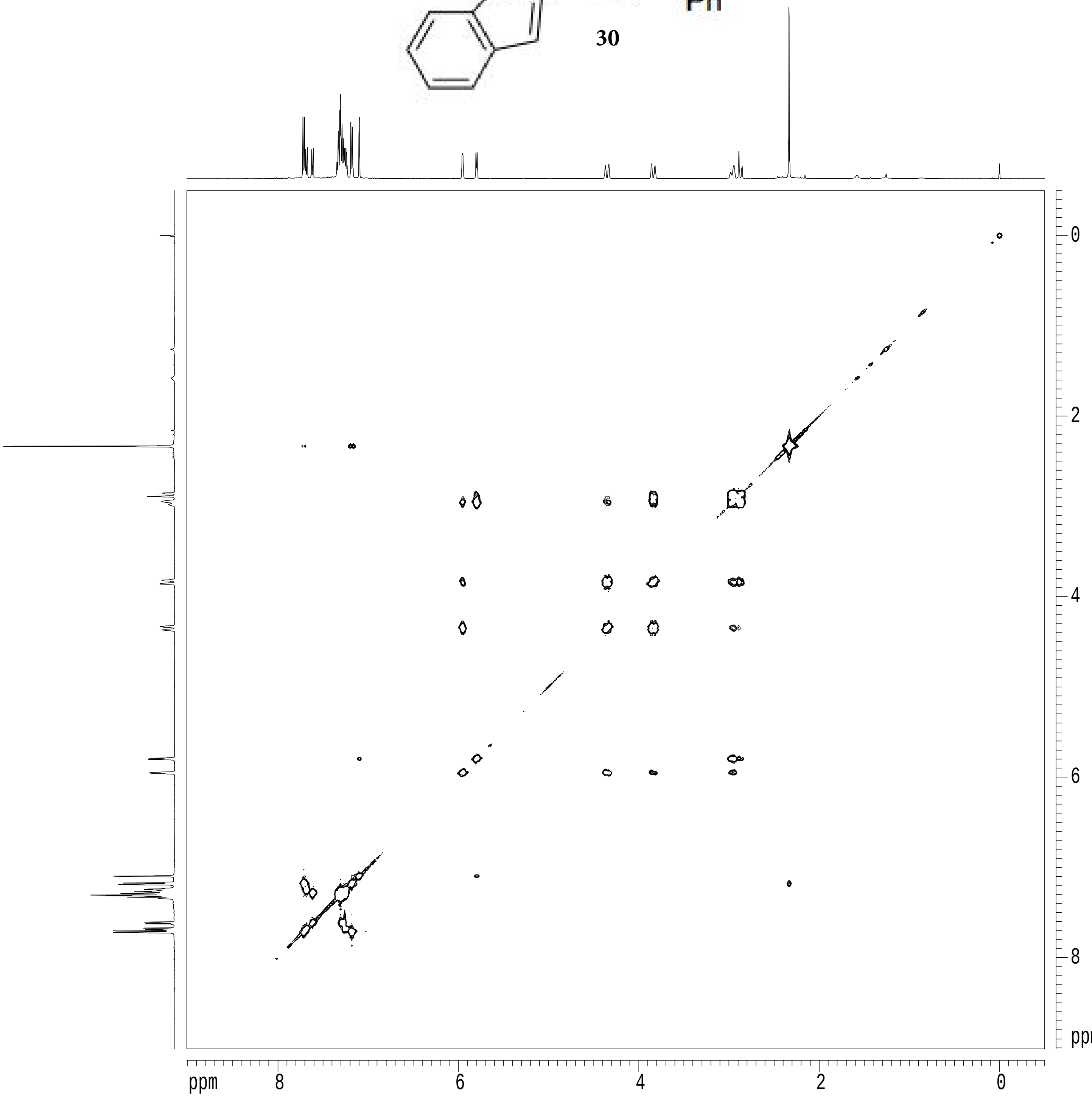


```
        Current Data Parameters
USER            khewitt1
NAME          KAH-I-291-Z
EXPNO                  4
PROCNO                 1

      F2 - Acquisition Parameters
Date_           20190302
Time               14.26
INSTRUM          cryo500
PROBHD   5 mm CPTCI 1H-
PULPROG    cosygp60.prd
TD                  2048
SOLVENT            CDCl3
NS                     1
DS                    16
SWH             8012.820 Hz
FIDRES          3.912510 Hz
AQ             0.1278452 sec
RG                   114
DW                62.400 usec
DE                  6.00 usec
TE                 298.0 K
d0            0.00000300 sec
D1            1.00000000 sec
d13           0.00000300 sec
D16           0.00020000 sec
IN0           0.00012480 sec

     ======== CHANNEL f1 ========
NUC1                  1H
P1                  7.50 usec
PL1                 1.60 dB
SFO1         500.2235015 MHz

     ====== GRADIENT CHANNEL =====
GPNAM1          sine.100
GPNAM2          sine.100
GPX1                0.00 %
GPX2                0.00 %
GPY1                0.00 %
GPY2                0.00 %
GPZ1               17.00 %
GPZ2               17.00 %
P16              1000.00 usec

      F1 - Acquisition parameters
ND0                    1
TD                   512
SFO1            500.2235 MHz
FIDRES         15.650040 Hz
SW                16.018 ppm
FnMODE                QF

      F2 - Processing parameters
SI                  1024
SF           500.2200426 MHz
WDW                 SINE
SSB                    0
LB                  0.00 Hz
GB                     0
PC                  1.00

      F1 - Processing parameters
SI                  1024
MC2                   QF
SF           500.2200426 MHz
WDW                 SINE
SSB                    0
LB                  0.00 Hz
GB                     0

        2D NMR plot parameters
CX2                15.00 cm
CX1                15.00 cm
F2PLO              9.011 ppm
F2LO             4507.45 Hz
F2PHI             -0.500 ppm
F2HI             -250.16 Hz
F1PLO              9.011 ppm
F1LO             4507.45 Hz
F1PHI             -0.500 ppm
F1HI             -250.16 Hz
F2PPMCM          0.63407 ppm/cm
F2HZCM         317.17413 Hz/cm
F1PPMCM          0.63407 ppm/cm
F1HZCM         317.17413 Hz/cm
```

1H spectrum

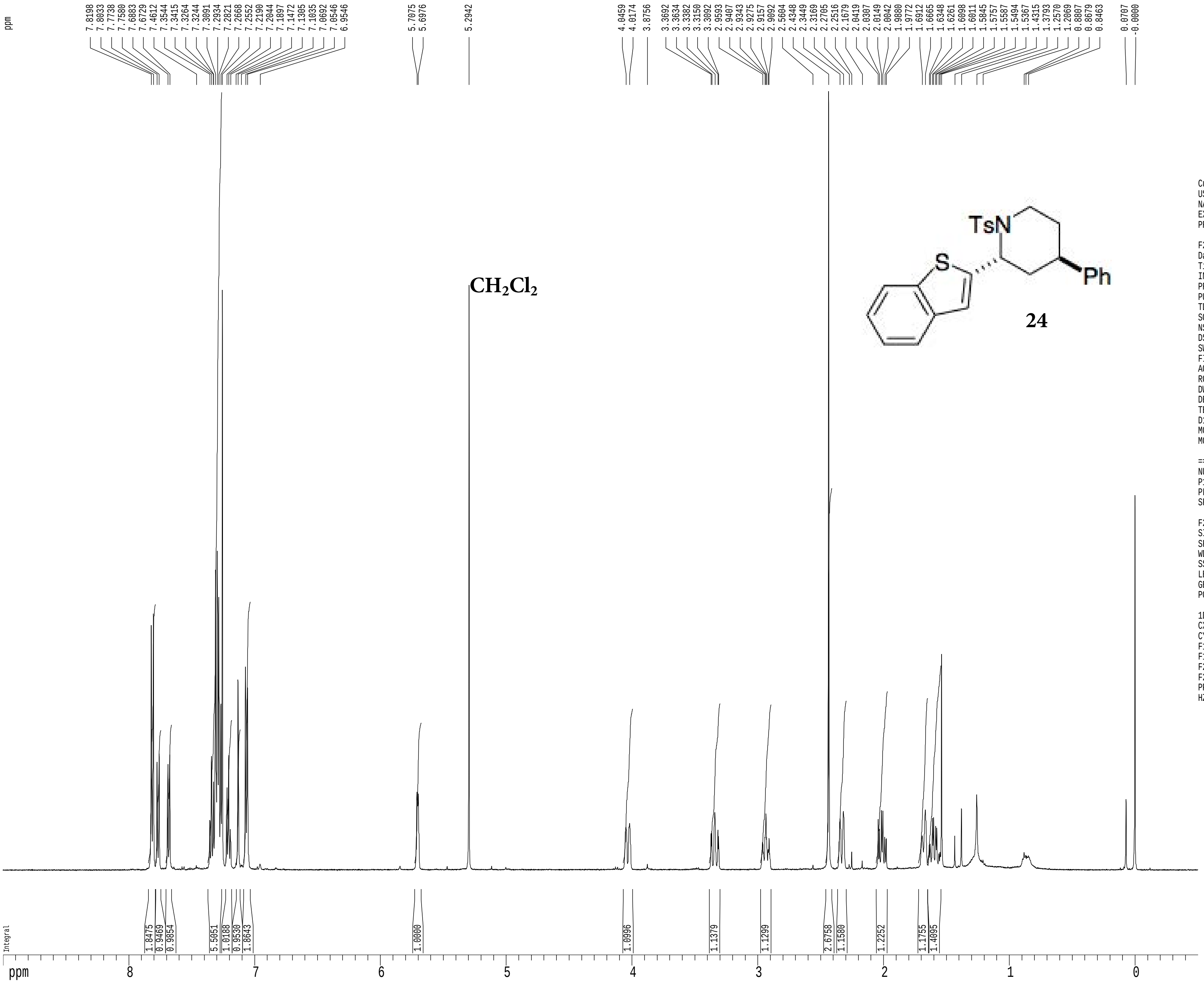

24

```
Current Data Parameters
USER            khewitt1
NAME         KAH-I-214-Z
EXPNO                  1
PROCNO                 1

F2 - Acquisition Parameters
Date_           20181204
Time               11.04
INSTRUM          cryo500
PROBHD   5 mm CPTCI 1H-
PULPROG             zg30
TD                 48074
SOLVENT           CDCl3T
NS                     8
DS                     2
SWH             8012.820 Hz
FIDRES          0.166677 Hz
AQ             2.9998677 sec
RG                   7.1
DW                62.400 usec
DE                  6.00 usec
TE                 298.0 K
D1            0.10000000 sec
MCREST        0.00000000 sec
MCWRK         0.01500000 sec

======== CHANNEL f1 ========
NUC1                  1H
P1                  7.50 usec
PL1                 1.60 dB
SFO1         500.2235015 MHz

F2 - Processing parameters
SI                 65536
SF           500.2200349 MHz
WDW                   no
SSB                    0
LB                  0.00 Hz
GB                     0
PC                  1.00

1D NMR plot parameters
CX                 22.80 cm
CY                 15.00 cm
F1P                9.000 ppm
F1               4501.98 Hz
F2P               -0.500 ppm
F2               -250.11 Hz
PPMCM            0.41667 ppm/cm
HZCM           208.42502 Hz/cm
```

c13.c

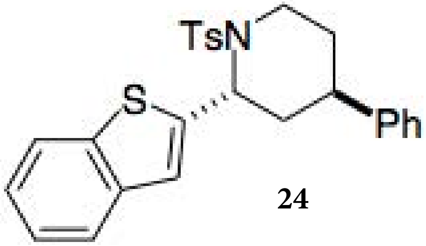

24

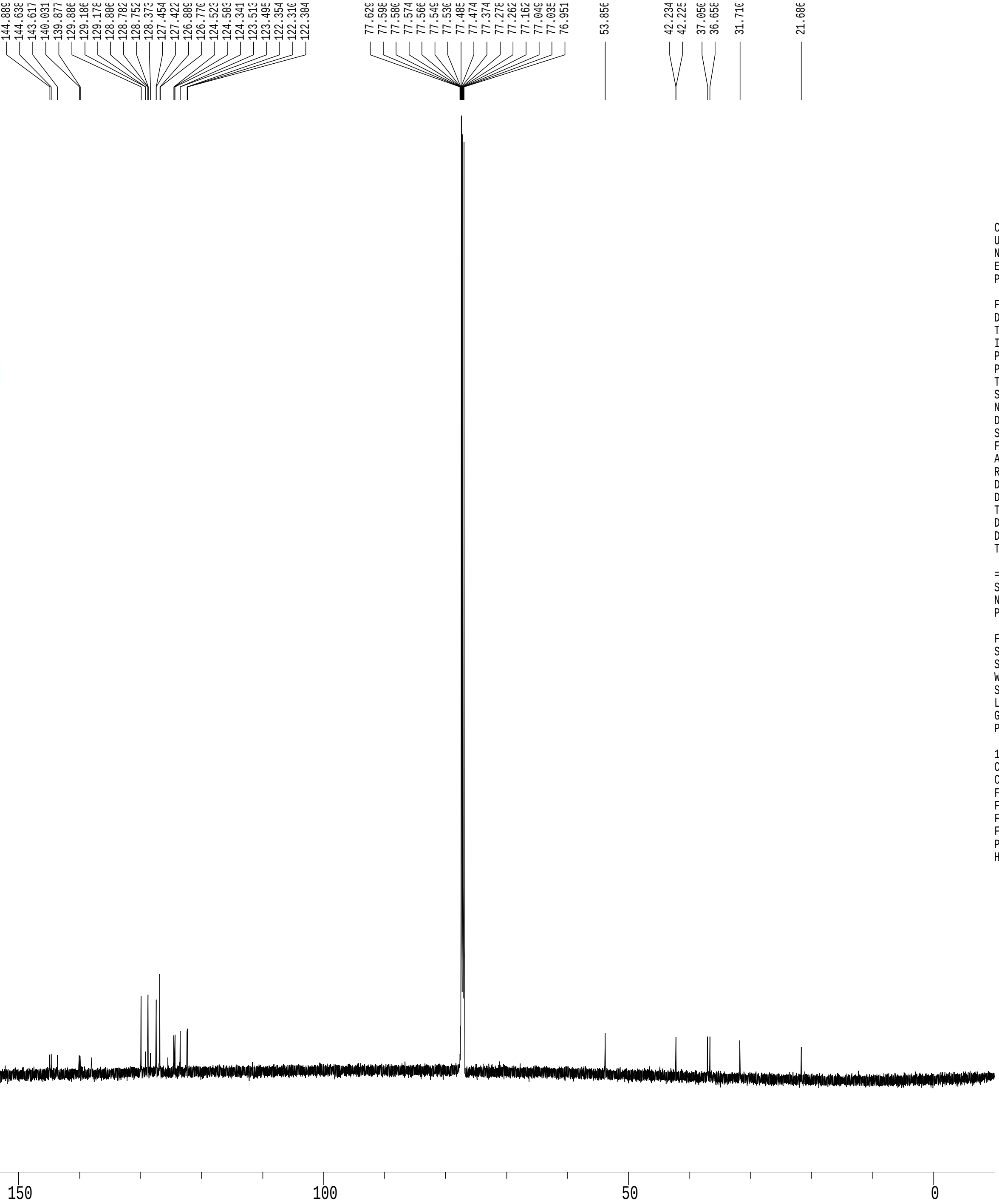

Current Data Parameters
USER khewitt1
NAME KAH-I-214-Z-C
EXPNO 2
PROCNO 1

F2 - Acquisition Parameters
Date_ 20210714
Time 11.13
INSTRUM av600
PROBHD 5 mm CPBBO BB-
PULPROG zgdc30
TD 65536
SOLVENT CDCl3T
NS 1024
DS 4
SWH 36231.883 Hz
FIDRES 0.552855 Hz
AQ 0.9044468 sec
RG 2050
DW 13.800 usec
DE 19.63 usec
TE 298.0 K
D1 0.40000001 sec
D11 0.03000000 sec
TD0 1

======== CHANNEL f1 ========
SFO1 150.9194080 MHz
NUC1 13C
P1 10.10 usec

F2 - Processing parameters
SI 65536
SF 150.9027936 MHz
WDW no
SSB 0
LB 0.00 Hz
GB 0
PC 0.10

1D NMR plot parameters
CX 22.80 cm
CY 15.00 cm
F1P 230.150 ppm
F1 34730.32 Hz
F2P -9.951 ppm
F2 -1501.56 Hz
PPMCM 10.53074 ppm/cm
HZCM 1589.11768 Hz/cm

gcosy60

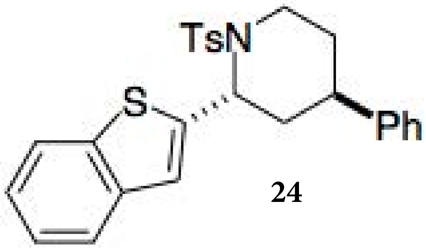

24

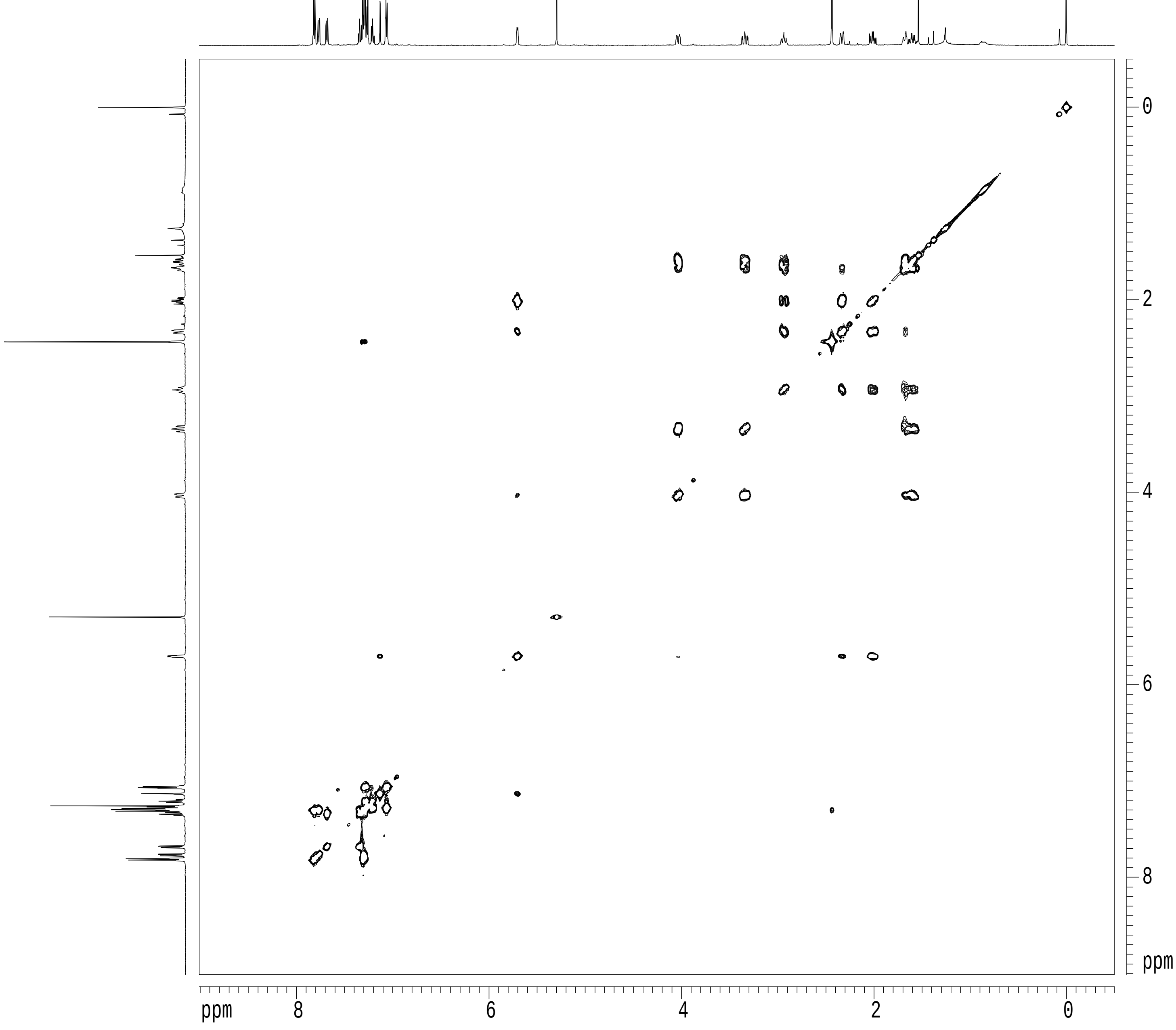

Current Data Parameters
USER khewitt1
NAME KAH-I-214-Z
EXPNO 2
PROCNO 1

F2 - Acquisition Parameters
Date_ 20181204
Time 11.06
INSTRUM cryo500
PROBHD 5 mm CPTCI 1H-
PULPROG cosygp60.prd
TD 2048
SOLVENT CDCl3
NS 1
DS 16
SWH 8012.820 Hz
FIDRES 3.912510 Hz
AQ 0.1278452 sec
RG 362
DW 62.400 usec
DE 6.00 usec
TE 298.0 K
d0 0.00000300 sec
D1 1.00000000 sec
d13 0.00000300 sec
D16 0.00020000 sec
IN0 0.00012480 sec

======== CHANNEL f1 ========
NUC1 1H
P1 7.50 usec
PL1 1.60 dB
SFO1 500.2235015 MHz

====== GRADIENT CHANNEL =====
GPNAM1 sine.100
GPNAM2 sine.100
GPX1 0.00 %
GPX2 0.00 %
GPY1 0.00 %
GPY2 0.00 %
GPZ1 17.00 %
GPZ2 17.00 %
P16 1000.00 usec

F1 - Acquisition parameters
ND0 1
TD 512
SFO1 500.2235 MHz
FIDRES 15.650040 Hz
SW 16.018 ppm
FnMODE QF

F2 - Processing parameters
SI 1024
SF 500.2200349 MHz
WDW SINE
SSB 0
LB 0.00 Hz
GB 0
PC 1.00

F1 - Processing parameters
SI 1024
MC2 QF
SF 500.2200349 MHz
WDW SINE
SSB 0
LB 0.00 Hz
GB 0

2D NMR plot parameters
CX2 15.00 cm
CX1 15.00 cm
F2PLO 9.011 ppm
F2LO 4507.33 Hz
F2PHI -0.500 ppm
F2HI -250.28 Hz
F1PLO 9.011 ppm
F1LO 4507.33 Hz
F1PHI -0.500 ppm
F1HI -250.28 Hz
F2PPMCM 0.63407 ppm/cm
F2HZCM 317.17413 Hz/cm
F1PPMCM 0.63407 ppm/cm
F1HZCM 317.17413 Hz/cm

gnoe

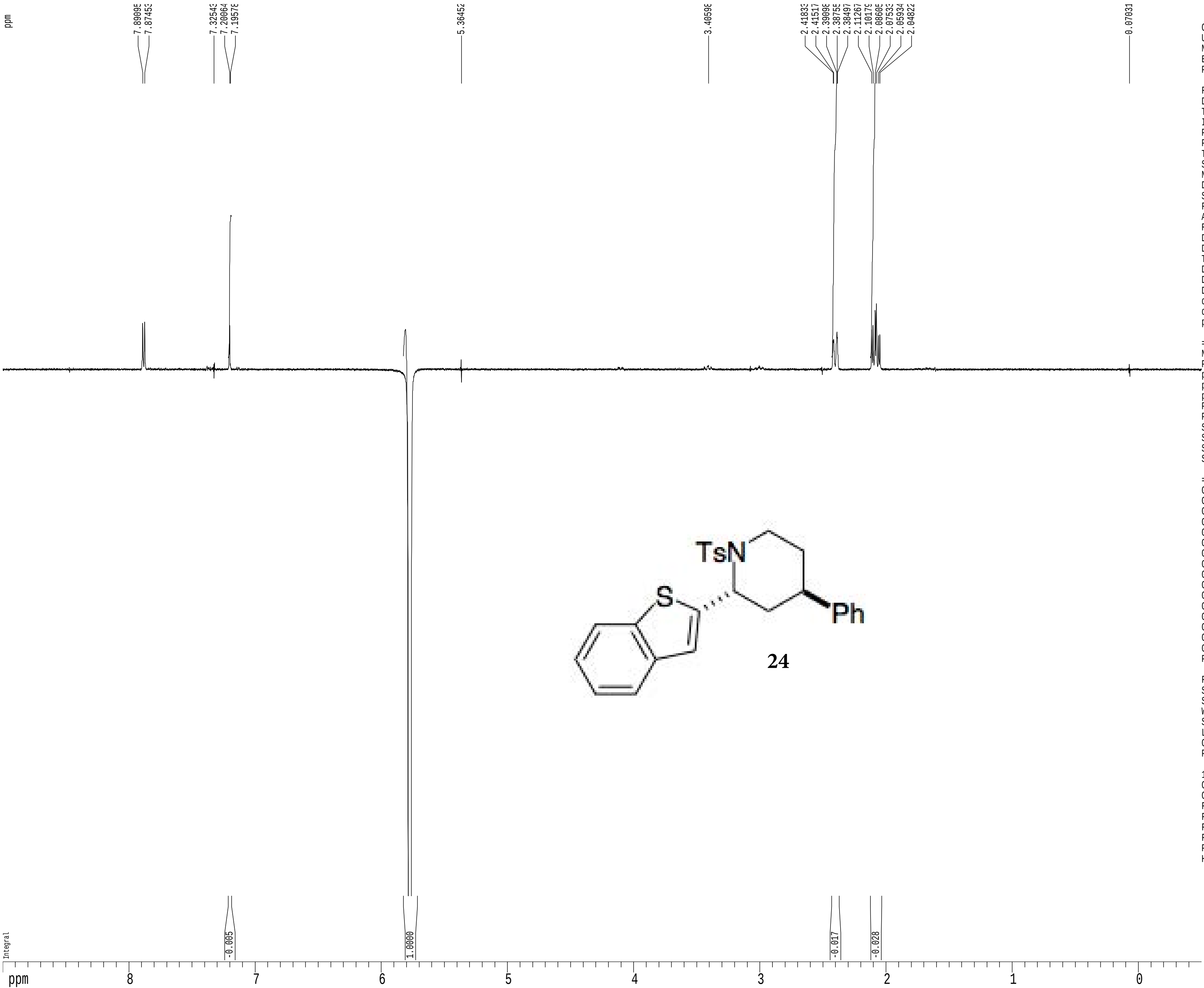

24

```
Current Data Parameters
USER            khewitt1
NAME          KAH-I-214-Z
EXPNO                  3
PROCNO                 1

F2 - Acquisition Parameters
Date_           20181204
Time               11.30
INSTRUM          cryo500
PROBHD   5 mm CPTCI 1H-
PULPROG   gnoe1cc22.prd
TD                 65536
SOLVENT            CDCl3
NS                   128
DS                     8
SWH             8012.820 Hz
FIDRES          0.122266 Hz
AQ             4.0894966 sec
RG                   114
DW                62.400 usec
DE                  6.00 usec
TE                 298.0 K
D1            1.00000000 sec
D8            0.50000000 sec
D16           0.00020000 sec
d21           0.33376500 sec
d22           0.16399699 sec
p2                 15.00 usec

======== CHANNEL f1 ========
NUC1                  1H
P1                  7.50 usec
p3                 22.50 usec
p4                 30.00 usec
p5                 20.00 usec
P29             40000.00 usec
PL1                 1.60 dB
SFO1         500.2228874 MHz
SP9                61.60 dB
SPNAM9         gauss1.512
SPOFF9              0.00 Hz

====== GRADIENT CHANNEL =====
GPNAM1          sine.100
GPNAM2          sine.100
GPNAM3          sine.100
GPNAM4          sine.100
GPX1                0.00 %
GPX2                0.00 %
GPX3                0.00 %
GPX4                0.00 %
GPY1                0.00 %
GPY2                0.00 %
GPY3                0.00 %
GPY4                0.00 %
GPZ1                7.00 %
GPZ2                3.00 %
GPZ3                2.30 %
GPZ4               -2.30 %
P16              1000.00 usec

F2 - Processing parameters
SI                 65536
SF           500.2200000 MHz
WDW                   no
SSB                    0
LB                  0.00 Hz
GB                     0
PC                  1.00

1D NMR plot parameters
CX                 22.80 cm
CY                 50.00 cm
F1P                9.000 ppm
F1               4501.98 Hz
F2P               -0.500 ppm
F2               -250.11 Hz
PPMCM            0.41667 ppm/cm
HZCM           208.42500 Hz/cm
```

1H spectrum

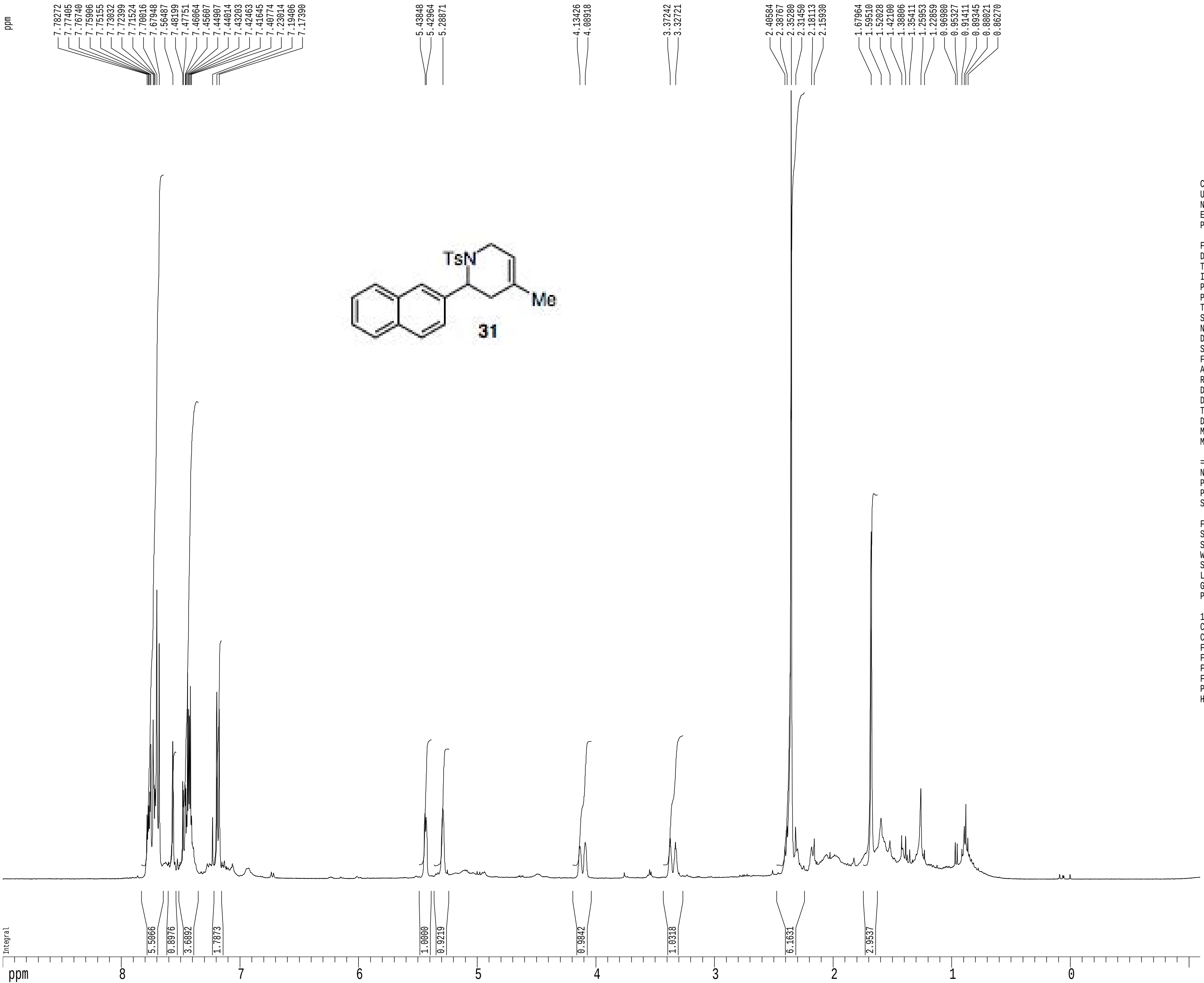


```
Current Data Parameters
USER          caherber
NAME         CAH-I-221
EXPNO                1
PROCNO               1

F2 - Acquisition Parameters
Date_         20210525
Time             11.17
INSTRUM         drx400
PROBHD   5 mm QNP H/F/P
PULPROG           zg30
TD               65536
SOLVENT          CDCl3
NS                   8
DS                   2
SWH           6410.256 Hz
FIDRES        0.097813 Hz
AQ           5.1118579 sec
RG                50.8
DW              78.000 usec
DE                4.50 usec
TE               298.0 K
D1          0.10000000 sec
MCREST      0.00000000 sec
MCWRK       0.01500000 sec

======== CHANNEL f1 ========
NUC1                1H
P1               12.00 usec
PL1              -1.60 dB
SFO1       400.1328009 MHz

F2 - Processing parameters
SI               65536
SF         400.1300333 MHz
WDW                 no
SSB                  0
LB                0.00 Hz
GB                   0
PC                2.00

1D NMR plot parameters
CX               22.80 cm
CY               15.00 cm
F1P              9.000 ppm
F1             3601.17 Hz
F2P             -1.093 ppm
F2             -437.51 Hz
PPMCM          0.44269 ppm/cm
HZCM         177.13533 Hz/cm
```

1H spectrum

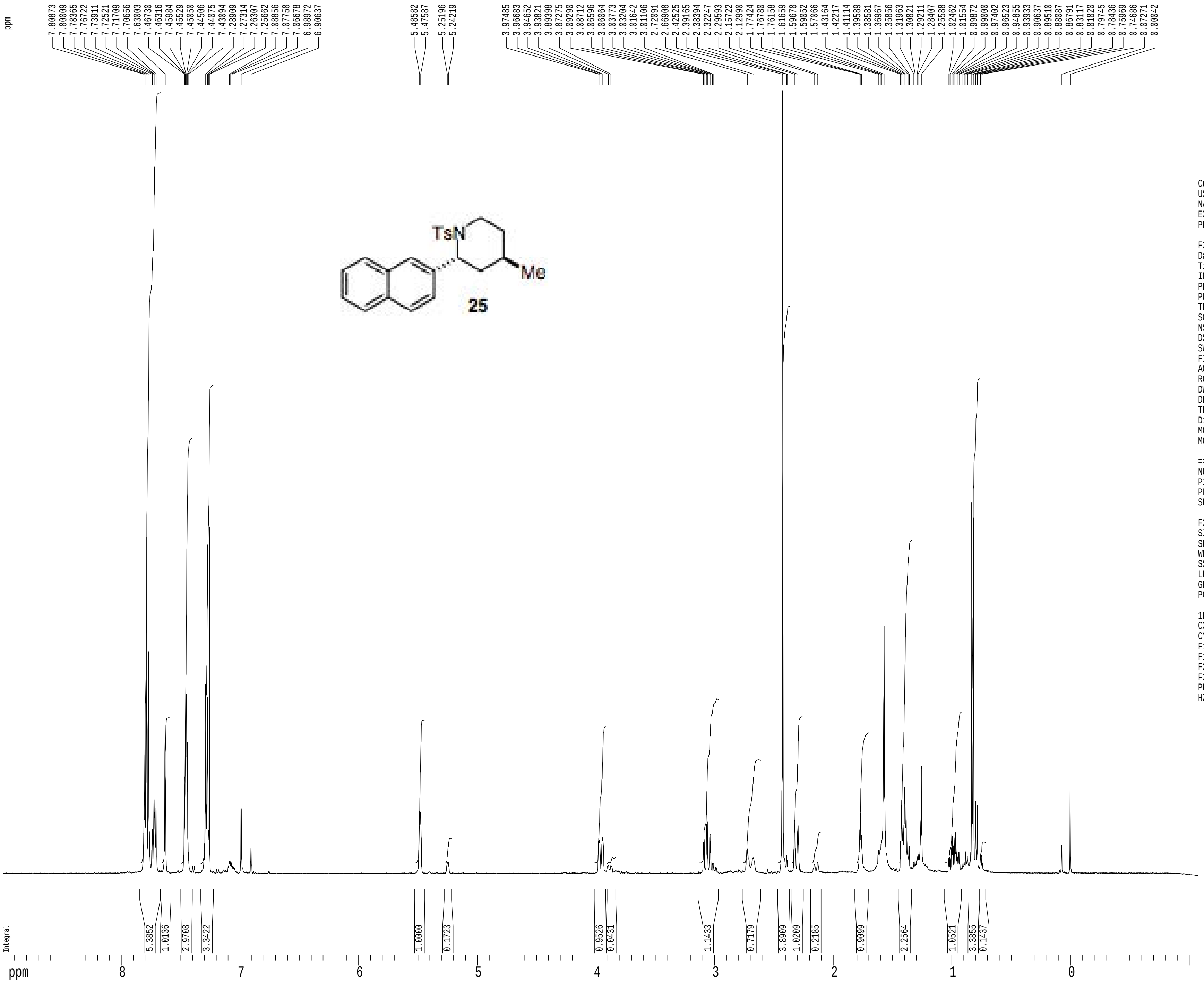

Current Data Parameters
USER caherber
NAME CAH-I-241-full
EXPNO 1
PROCNO 1

F2 - Acquisition Parameters
Date_ 20210707
Time 11.46
INSTRUM cryo500
PROBHD 5 mm CPTCI 1H-
PULPROG zg30
TD 81728
SOLVENT CDCl3
NS 8
DS 2
SWH 8012.820 Hz
FIDRES 0.098043 Hz
AQ 5.0998774 sec
RG 6.3
DW 62.400 usec
DE 6.00 usec
TE 298.0 K
D1 0.10000000 sec
MCREST 0.00000000 sec
MCWRK 0.01500000 sec

======== CHANNEL f1 ========
NUC1 1H
P1 9.75 usec
PL1 1.60 dB
SFO1 500.2235015 MHz

F2 - Processing parameters
SI 65536
SF 500.2200327 MHz
WDW no
SSB 0
LB 0.00 Hz
GB 0
PC 1.00

1D NMR plot parameters
CX 22.80 cm
CY 15.00 cm
F1P 9.000 ppm
F1 4501.98 Hz
F2P -1.075 ppm
F2 -537.60 Hz
PPMCM 0.44187 ppm/cm
HZCM 221.03436 Hz/cm

Z-restored spin-echo 13C spectrum with 1H decoupling

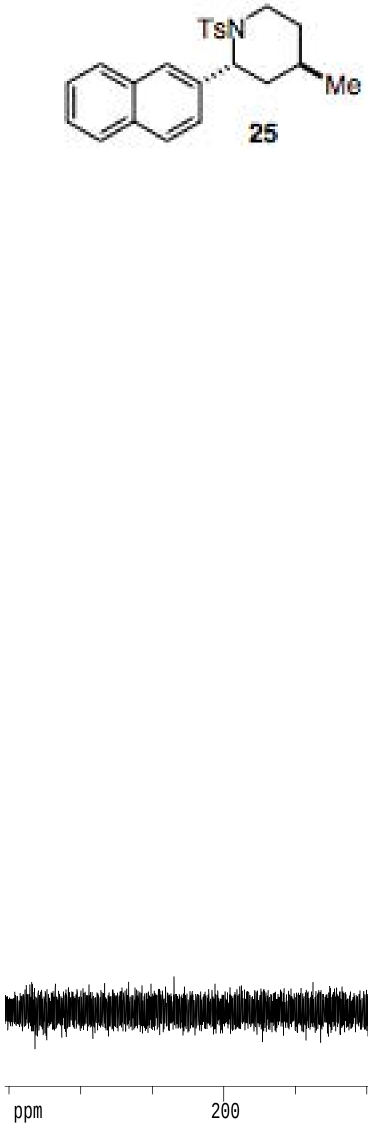

25

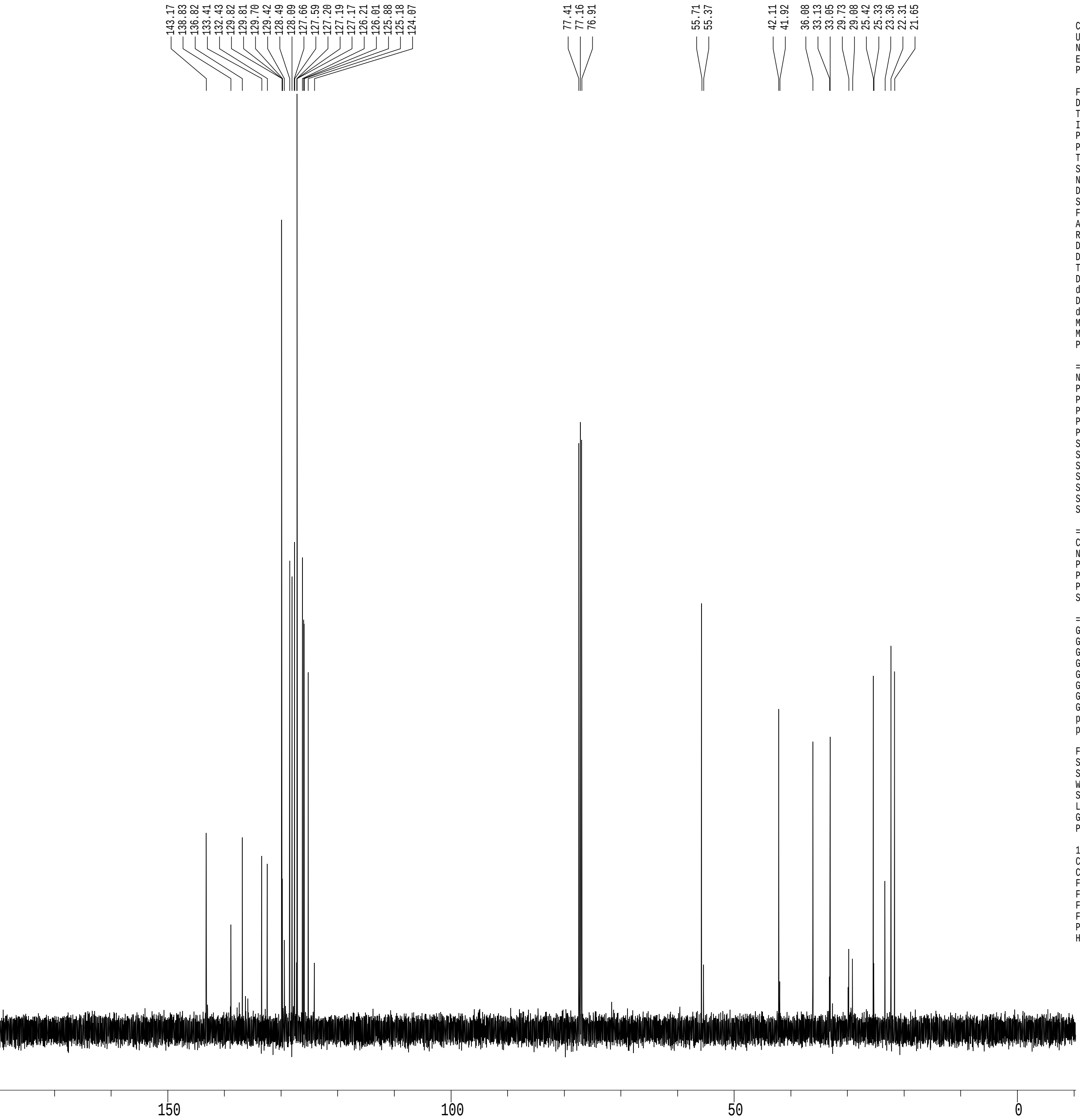

Current Data Parameters
USER caherber
NAME CAH-I-241-full
EXPNO 5
PROCNO 1

F2 - Acquisition Parameters
Date_ 20210707
Time 13.59
INSTRUM cryo500
PROBHD 5 mm CPTCI 1H-
PULPROG SpinEchopg30gp2.prd
TD 65536
SOLVENT CDCl3
NS 264
DS 16
SWH 30303.031 Hz
FIDRES 0.462388 Hz
AQ 1.0813940 sec
RG 7298.2
DW 16.500 usec
DE 6.00 usec
TE 298.0 K
D1 0.25000000 sec
d11 0.03000000 sec
D16 0.00020000 sec
d17 0.00019600 sec
MCREST 0.00000000 sec
MCWRK 0.01500000 sec
P2 37.70 usec

======== CHANNEL f1 ========
NUC1 13C
P1 18.85 usec
P12 2000.00 usec
P20 500.00 usec
PL0 120.00 dB
PL1 -1.00 dB
SFO1 125.7942548 MHz
SP2 1.55 dB
SP4 1.55 dB
SPNAM2 Crp60comp.4
SPNAM4 Crp60,0.5,20.1
SPOFF2 0.00 Hz
SPOFF4 0.00 Hz

======== CHANNEL f2 ========
CPDPRG2 waltz16
NUC2 1H
PCPD2 100.00 usec
PL2 1.60 dB
PL12 22.00 dB
SFO2 500.2225011 MHz

====== GRADIENT CHANNEL =====
GPNAM1 SINE.100
GPNAM2 SINE.100
GPX1 0.00 %
GPX2 0.00 %
GPY1 0.00 %
GPY2 0.00 %
GPZ1 30.00 %
GPZ2 50.00 %
p15 500.00 usec
p16 1000.00 usec

F2 - Processing parameters
SI 65536
SF 125.7804076 MHz
WDW no
SSB 0
LB 0.00 Hz
GB 0
PC 2.00

1D NMR plot parameters
CX 22.80 cm
CY 15.65 cm
F1P 230.637 ppm
F1 29009.68 Hz
F2P -10.287 ppm
F2 -1293.96 Hz
PPMCM 10.56688 ppm/cm
HZCM 1329.10693 Hz/cm

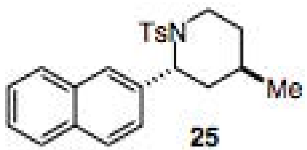

25

gcosy60

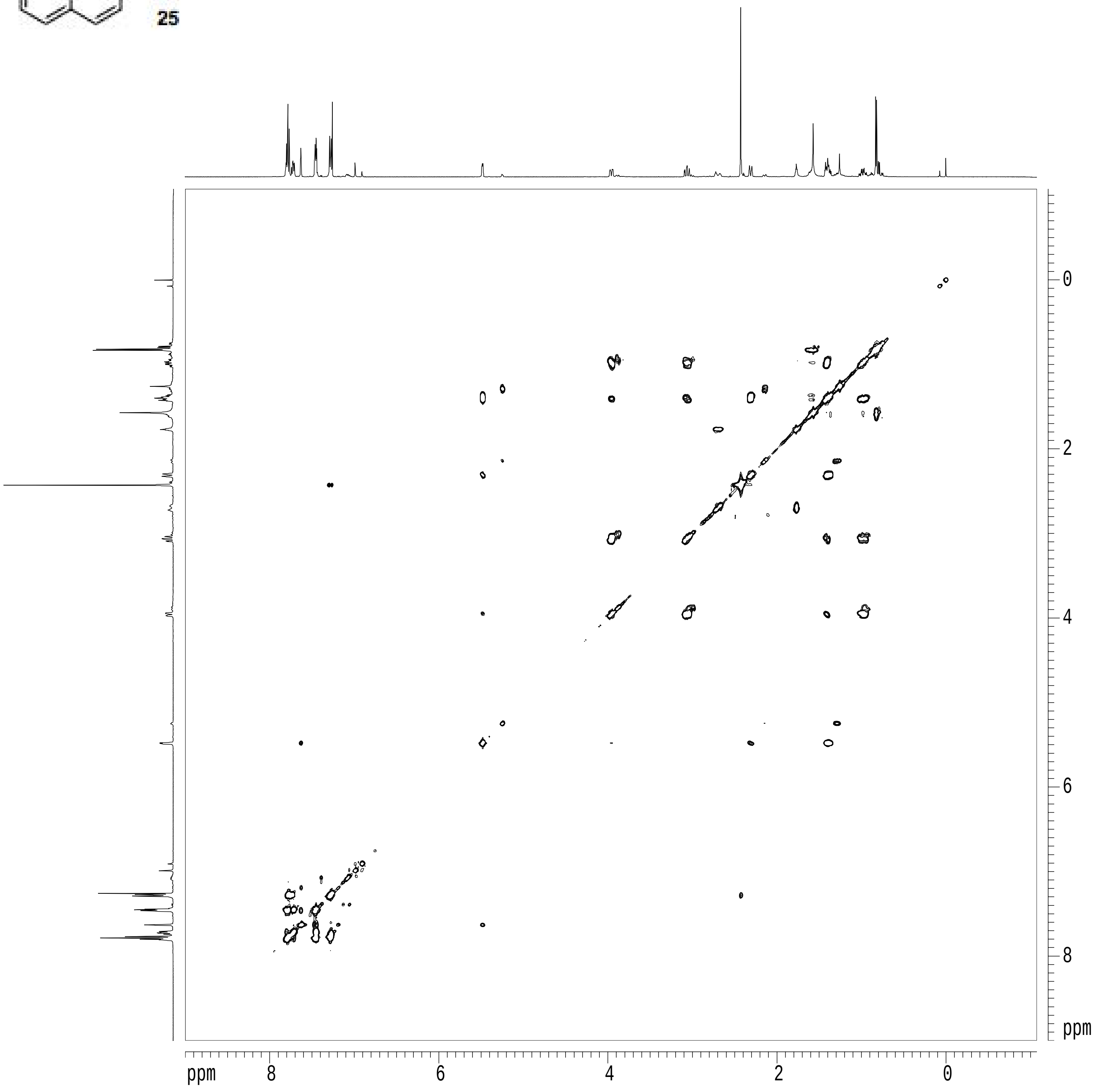


```
        Current Data Parameters
USER           caherber
NAME     CAH-I-241-full
EXPNO                 2
PROCNO                1

      F2 - Acquisition Parameters
Date_          20210707
Time              11.49
INSTRUM         cryo500
PROBHD   5 mm CPTCI 1H-
PULPROG    cosygp60.prd
TD                 2048
SOLVENT           CDCl3
NS                    1
DS                   16
SWH            8012.820 Hz
FIDRES         3.912510 Hz
AQ            0.1278452 sec
RG                143.7
DW               62.400 usec
DE                 6.00 usec
TE                298.0 K
d0           0.00000300 sec
D1           1.00000000 sec
d13          0.00000300 sec
D16          0.00020000 sec
IN0          0.00012480 sec

     ======== CHANNEL f1 ========
NUC1                 1H
P1                 9.75 usec
PL1                1.60 dB
SFO1        500.2235015 MHz

     ====== GRADIENT CHANNEL =====
GPNAM1       SMSQ10.100
GPNAM2       SMSQ10.100
GPX1               0.00 %
GPX2               0.00 %
GPY1               0.00 %
GPY2               0.00 %
GPZ1              17.00 %
GPZ2              17.00 %
P16             1000.00 usec

      F1 - Acquisition parameters
ND0                   1
TD                  512
SFO1           500.2235 MHz
FIDRES        15.650040 Hz
SW               16.018 ppm
FnMODE               QF

      F2 - Processing parameters
SI                 1024
SF          500.2200327 MHz
WDW                SINE
SSB                   0
LB                 0.00 Hz
GB                    0
PC                 1.00

      F1 - Processing parameters
SI                 1024
MC2                  QF
SF          500.2200327 MHz
WDW                SINE
SSB                   0
LB                 0.00 Hz
GB                    0

      2D NMR plot parameters
CX2               15.00 cm
CX1               15.00 cm
F2PLO             9.000 ppm
F2LO            4501.98 Hz
F2PHI            -1.075 ppm
F2HI            -537.60 Hz
F1PLO             9.000 ppm
F1LO            4501.98 Hz
F1PHI            -1.075 ppm
F1HI            -537.60 Hz
F2PPMCM         0.67165 ppm/cm
F2HZCM        335.97217 Hz/cm
F1PPMCM         0.67165 ppm/cm
F1HZCM        335.97217 Hz/cm
```

gnoe

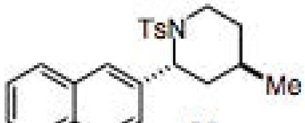

25

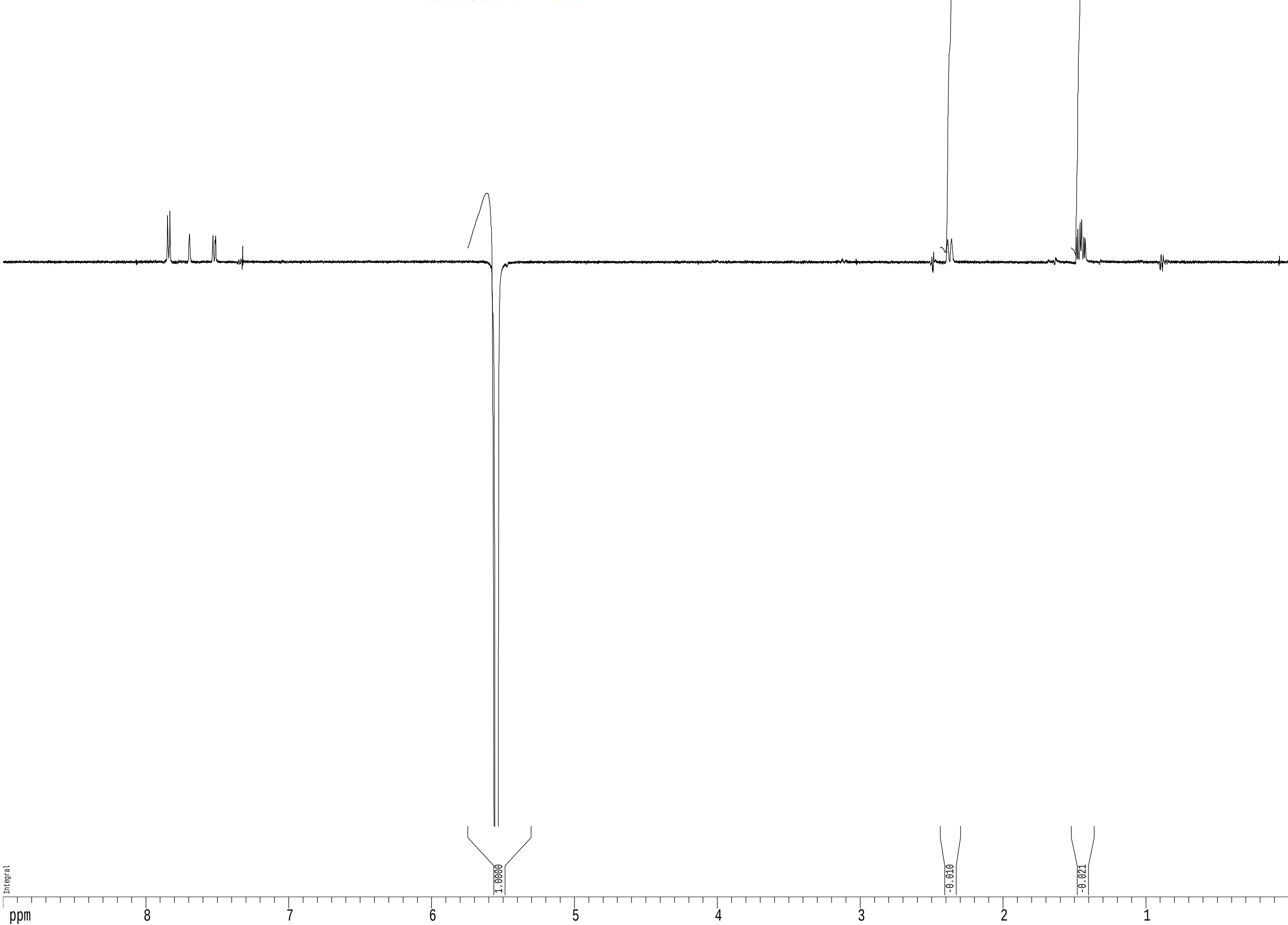

Current Data Parameters
USER caherber
NAME CAH-I-241-full
EXPNO 4
PROCNO 1

F2 - Acquisition Parameters
Date_ 20210707
Time 13.52
INSTRUM cryo500
PROBHD 5 mm CPTCI 1H-
PULPROG gnoe1cc22.prd
TD 65536
SOLVENT CDCl3
NS 128
DS 8
SWH 8012.820 Hz
FIDRES 0.122266 Hz
AQ 4.0894966 sec
RG 101.6
DW 62.400 usec
DE 6.00 usec
TE 298.0 K
D1 1.00000000 sec
D8 0.50000000 sec
D16 0.00020000 sec
d21 0.33375451 sec
d22 0.16399699 sec
p2 19.50 usec

======== CHANNEL f1 ========
NUC1 1H
P1 9.75 usec
p3 29.25 usec
p4 39.00 usec
p5 26.00 usec
P29 40000.00 usec
PL1 1.60 dB
SFO1 500.2227742 MHz
SP9 60.00 dB
SPNAM9 gauss1.512
SPOFF9 0.00 Hz

====== GRADIENT CHANNEL =====
GPNAM1 SMSQ10.100
GPNAM2 SMSQ10.100
GPNAM3 SMSQ10.100
GPNAM4 SMSQ10.100
GPX1 0.00 %
GPX2 0.00 %
GPX3 0.00 %
GPX4 0.00 %
GPY1 0.00 %
GPY2 0.00 %
GPY3 0.00 %
GPY4 0.00 %
GPZ1 7.00 %
GPZ2 3.00 %
GPZ3 2.30 %
GPZ4 -2.30 %
P16 1000.00 usec

F2 - Processing parameters
SI 65536
SF 500.2200000 MHz
WDW no
SSB 0
LB 0.00 Hz
GB 0
PC 1.00

1D NMR plot parameters
CX 22.80 cm
CY 50.00 cm
F1P 9.000 ppm
F1 4501.98 Hz
F2P 0.000 ppm
F2 0.00 Hz
PPMCM 0.39474 ppm/cm
HZCM 197.45528 Hz/cm

1H spectrum

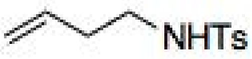

34

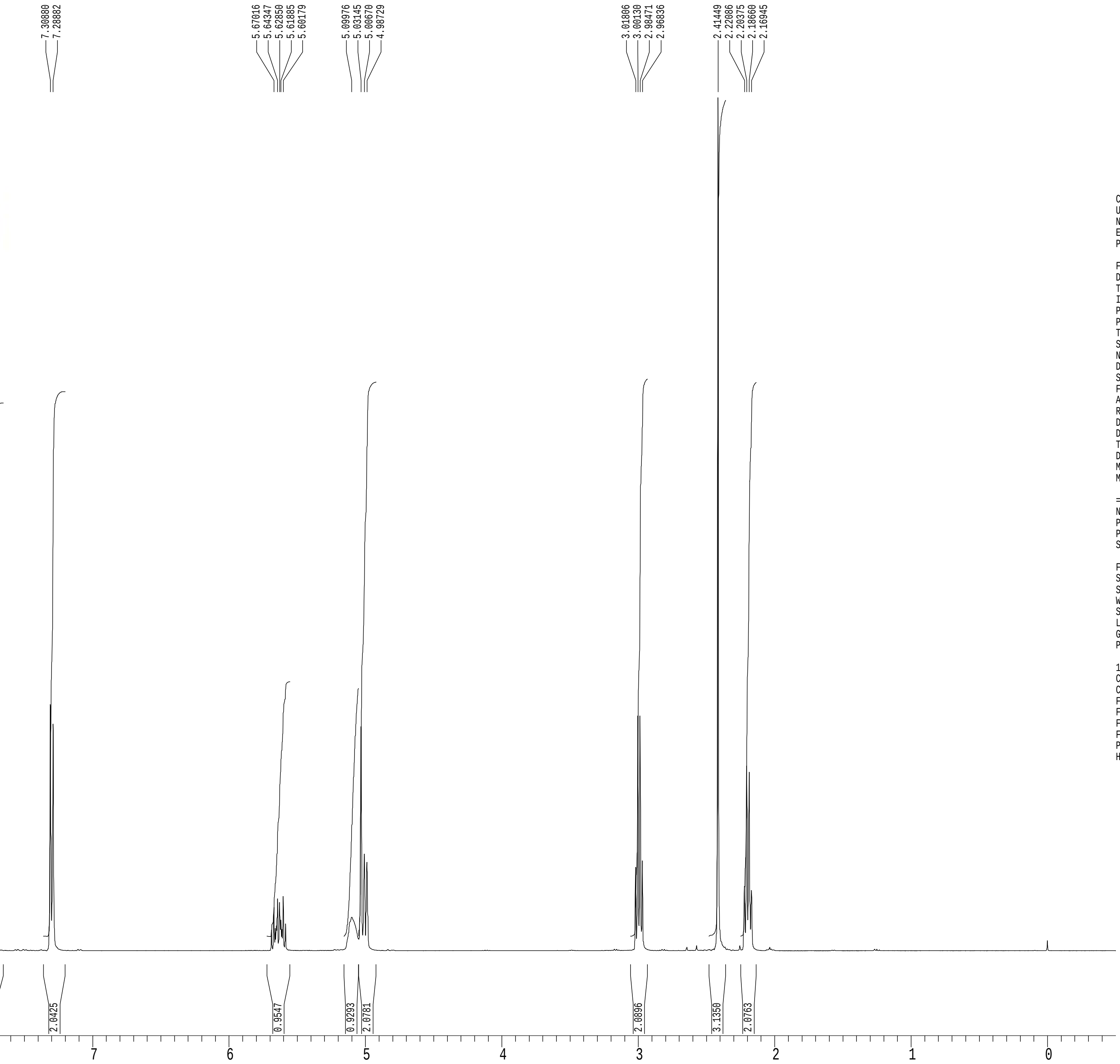


```
Current Data Parameters
USER          khewitt1
NAME        KAH-I-102Z
EXPNO                1
PROCNO               1

F2 - Acquisition Parameters
Date_         20180922
Time             12.03
INSTRUM         drx400
PROBHD   5 mm QNP H/F/P
PULPROG           zg30
TD               38460
SOLVENT         CDCl3T
NS                   8
DS                   2
SWH           6410.256 Hz
FIDRES        0.166673 Hz
AQ           2.9999299 sec
RG                  32
DW              78.000 usec
DE                4.50 usec
TE               297.9 K
D1          0.10000000 sec
MCREST      0.00000000 sec
MCWRK       0.01500000 sec

======== CHANNEL f1 ========
NUC1                1H
P1               12.00 usec
PL1              -1.10 dB
SFO1       400.1328009 MHz

F2 - Processing parameters
SI               65536
SF         400.1300086 MHz
WDW                 no
SSB                  0
LB                0.00 Hz
GB                   0
PC                2.00

1D NMR plot parameters
CX               22.80 cm
CY               15.00 cm
F1P              9.000 ppm
F1             3601.17 Hz
F2P             -0.500 ppm
F2             -200.06 Hz
PPMCM          0.41667 ppm/cm
HZCM         166.72084 Hz/cm
```

1H spectrum

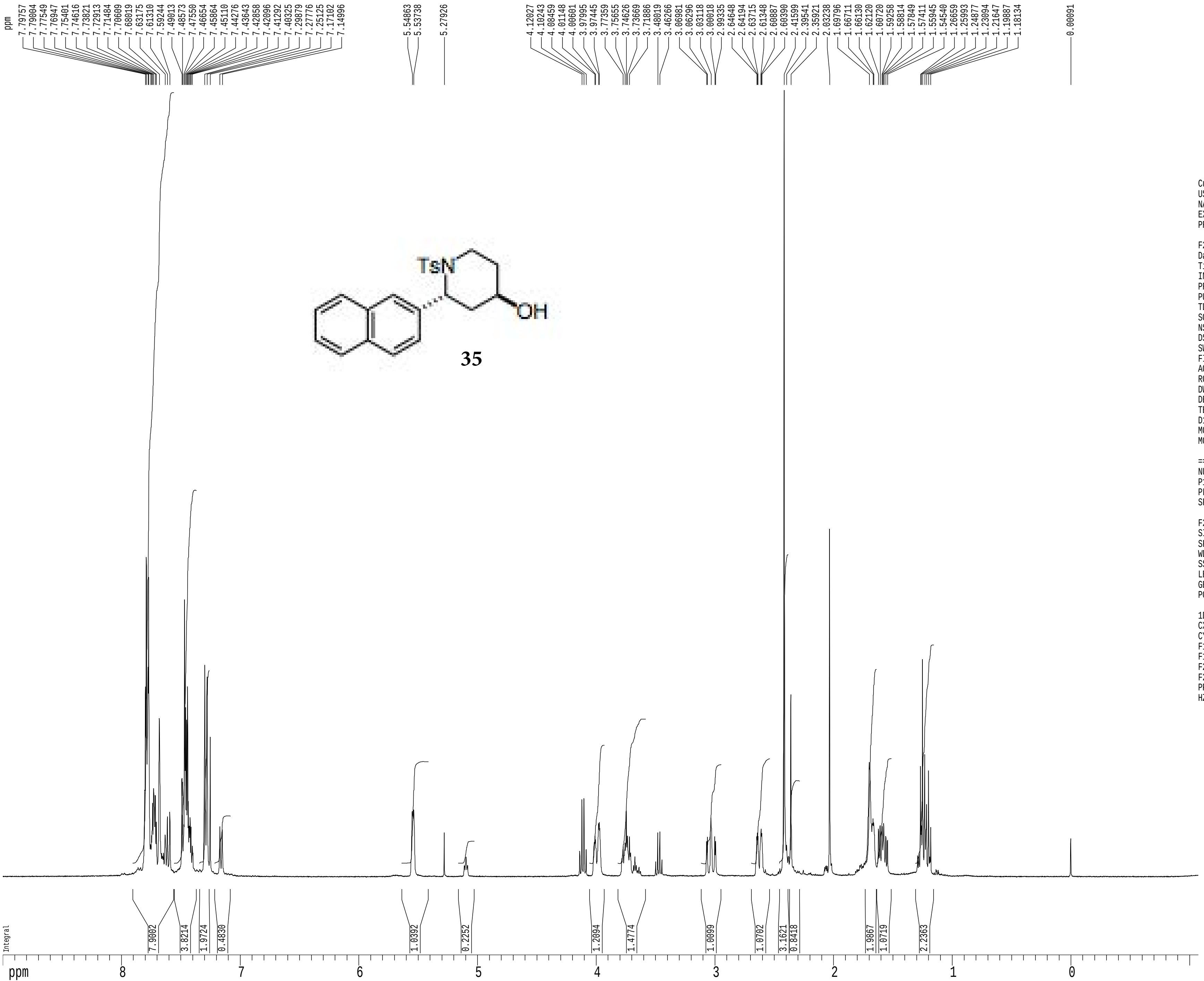


```
Current Data Parameters
USER          caherber
NAME          CAH-I-201
EXPNO                1
PROCNO               1

F2 - Acquisition Parameters
Date_          20210330
Time              18.04
INSTRUM          drx400
PROBHD   5 mm QNP H/F/P
PULPROG            zg30
TD                65536
SOLVENT           CDCl3
NS                    8
DS                    2
SWH            6410.256 Hz
FIDRES         0.097813 Hz
AQ            5.1118579 sec
RG                  128
DW               78.000 usec
DE                 4.50 usec
TE                298.0 K
D1           0.10000000 sec
MCREST       0.00000000 sec
MCWRK        0.01500000 sec

======== CHANNEL f1 ========
NUC1                 1H
P1                12.00 usec
PL1               -1.60 dB
SFO1        400.1328009 MHz

F2 - Processing parameters
SI                65536
SF          400.1300245 MHz
WDW                  no
SSB                   0
LB                 0.00 Hz
GB                    0
PC                 2.00

1D NMR plot parameters
CX                22.80 cm
CY                15.00 cm
F1P               9.000 ppm
F1              3601.17 Hz
F2P              -1.071 ppm
F2              -428.71 Hz
PPMCM           0.44173 ppm/cm
HZCM          176.74925 Hz/cm
```

Z-restored spin-echo 13C spectrum with 1H decoupling

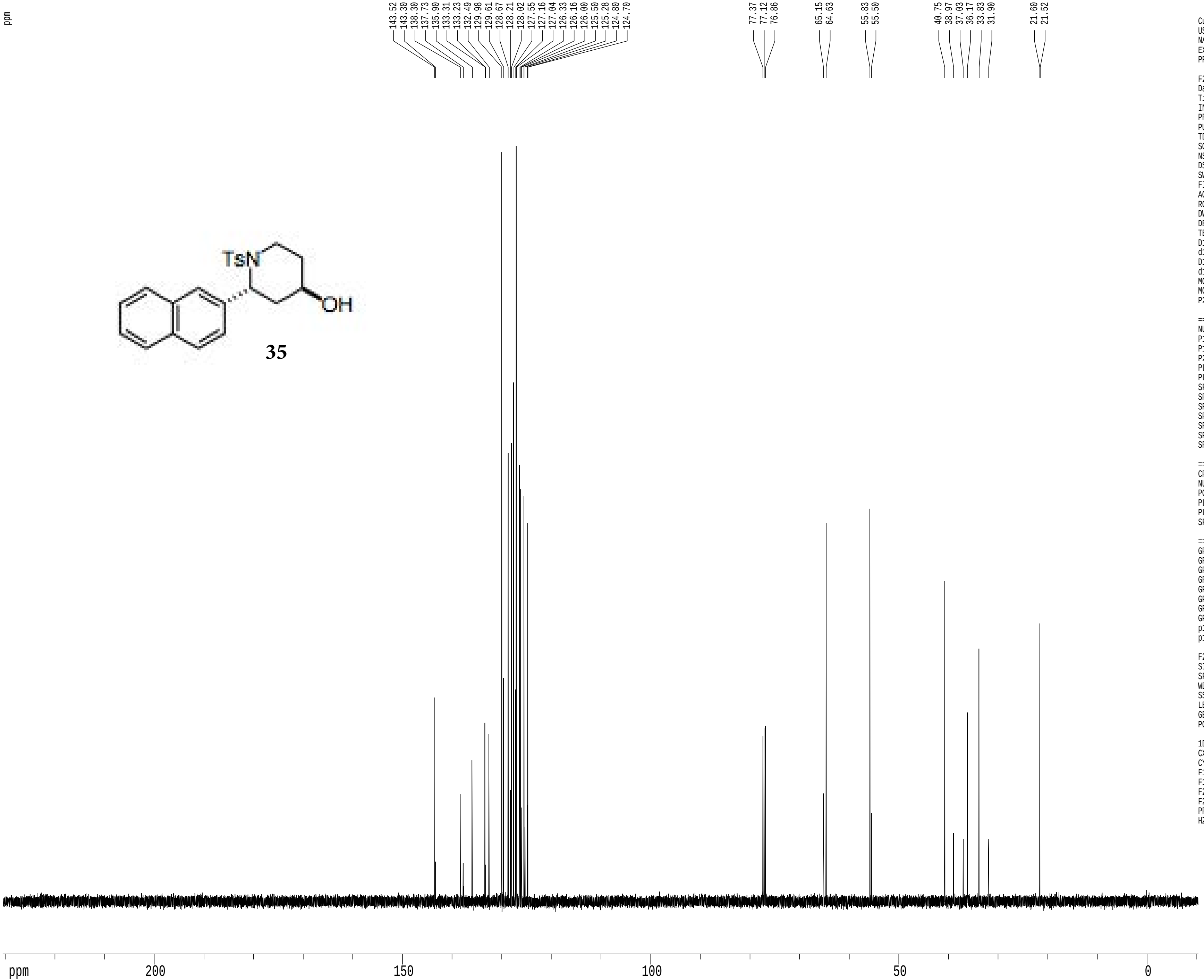

35

Current Data Parameters
USER caherber
NAME CAH-I-202-13C
EXPNO 1
PROCNO 1

F2 - Acquisition Parameters
Date_ 20210828
Time 14.09
INSTRUM cryo500
PROBHD 5 mm CPTCI 1H-
PULPROG SpinEchopg30gp2.prd
TD 65536
SOLVENT CDCl3
NS 200
DS 16
SWH 30303.031 Hz
FIDRES 0.462388 Hz
AQ 1.0813940 sec
RG 13004
DW 16.500 usec
DE 6.00 usec
TE 298.0 K
D1 0.25000000 sec
d11 0.03000000 sec
D16 0.00020000 sec
d17 0.00019600 sec
MCREST 0.00000000 sec
MCWRK 0.01500000 sec
P2 37.70 usec

======== CHANNEL f1 ========
NUC1 13C
P1 18.85 usec
P12 2000.00 usec
P20 500.00 usec
PL0 120.00 dB
PL1 -1.00 dB
SFO1 125.7942548 MHz
SP2 1.55 dB
SP4 1.55 dB
SPNAM2 Crp60comp.4
SPNAM4 Crp60,0.5,20.1
SPOFF2 0.00 Hz
SPOFF4 0.00 Hz

======== CHANNEL f2 ========
CPDPRG2 waltz16
NUC2 1H
PCPD2 100.00 usec
PL2 1.60 dB
PL12 22.00 dB
SFO2 500.2225011 MHz

====== GRADIENT CHANNEL =====
GPNAM1 SINE.100
GPNAM2 SINE.100
GPX1 0.00 %
GPX2 0.00 %
GPY1 0.00 %
GPY2 0.00 %
GPZ1 30.00 %
GPZ2 50.00 %
p15 500.00 usec
p16 1000.00 usec

F2 - Processing parameters
SI 65536
SF 125.7804190 MHz
WDW no
SSB 0
LB 0.00 Hz
GB 0
PC 2.00

1D NMR plot parameters
CX 22.80 cm
CY 15.65 cm
F1P 230.637 ppm
F1 29009.68 Hz
F2P -10.287 ppm
F2 -1293.96 Hz
PPMCM 10.56688 ppm/cm
HZCM 1329.10706 Hz/cm

1H spectrum

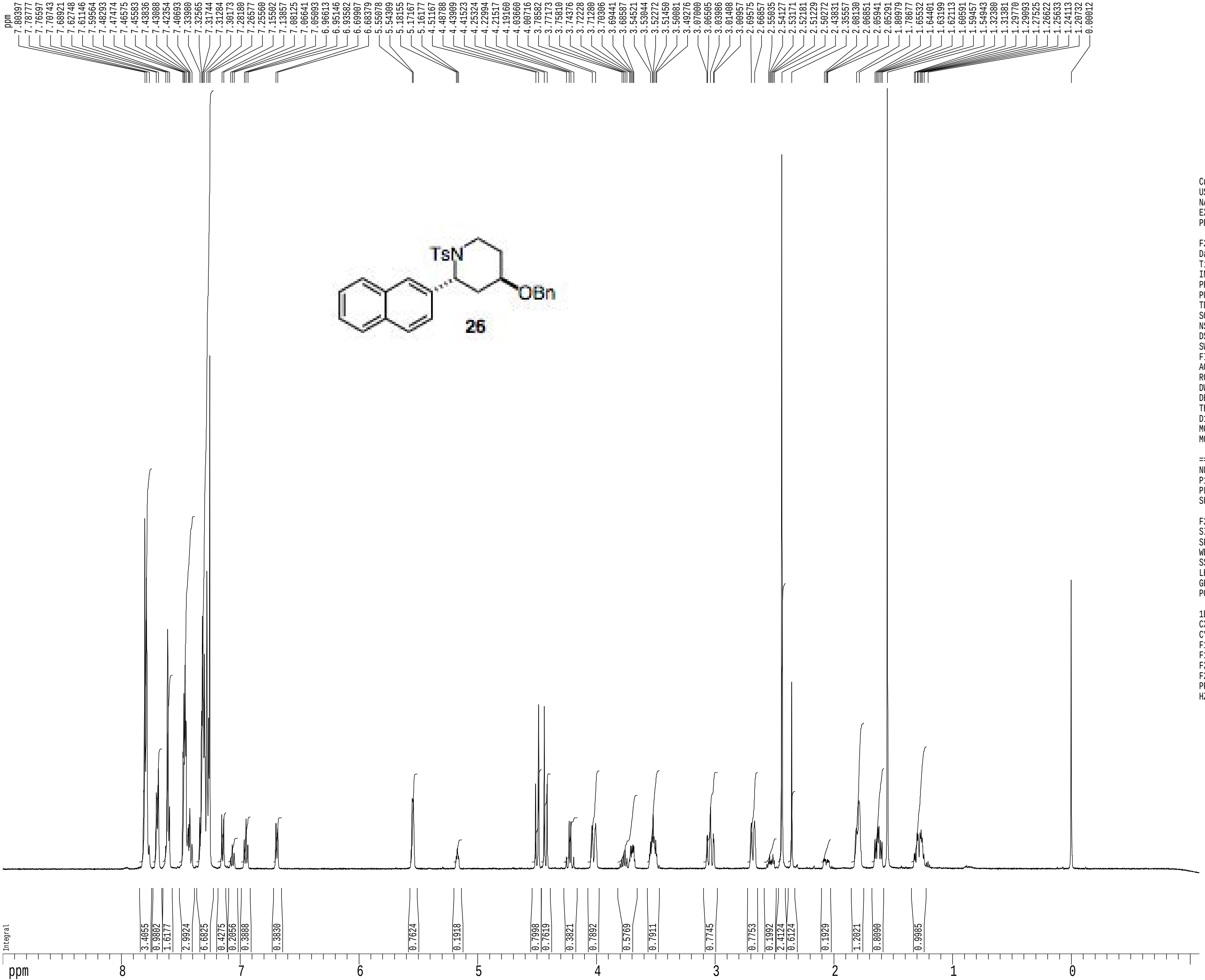

Current Data Parameters

USER	caherber
NAME	CAH-I-236-full
EXPNO	1
PROCNO	1

F2 - Acquisition Parameters

Date_	20210616
Time	7.56
INSTRUM	cryo500
PROBHD	5 mm CPTCI 1H-
PULPROG	zg30
TD	48074
SOLVENT	CDCl3
NS	8
DS	2
SWH	8012.820 Hz
FIDRES	0.166677 Hz
AQ	2.9998677 sec
RG	5.7
DW	62.400 usec
DE	6.00 usec
TE	298.0 K
D1	0.10000000 sec
MCREST	0.00000000 sec
MCWRK	0.01500000 sec

======== CHANNEL f1 ========

NUC1	1H
P1	9.75 usec
PL1	1.60 dB
SFO1	500.2235015 MHz

F2 - Processing parameters

SI	65536
SF	500.2200329 MHz
WDW	no
SSB	0
LB	0.00 Hz
GB	0
PC	1.00

1D NMR plot parameters

CX	22.80 cm
CY	15.00 cm
F1P	9.000 ppm
F1	4501.98 Hz
F2P	-1.075 ppm
F2	-537.73 Hz
PPMCM	0.44189 ppm/cm
HZCM	221.03986 Hz/cm

Z-restored spin-echo 13C spectrum with 1H decoupling

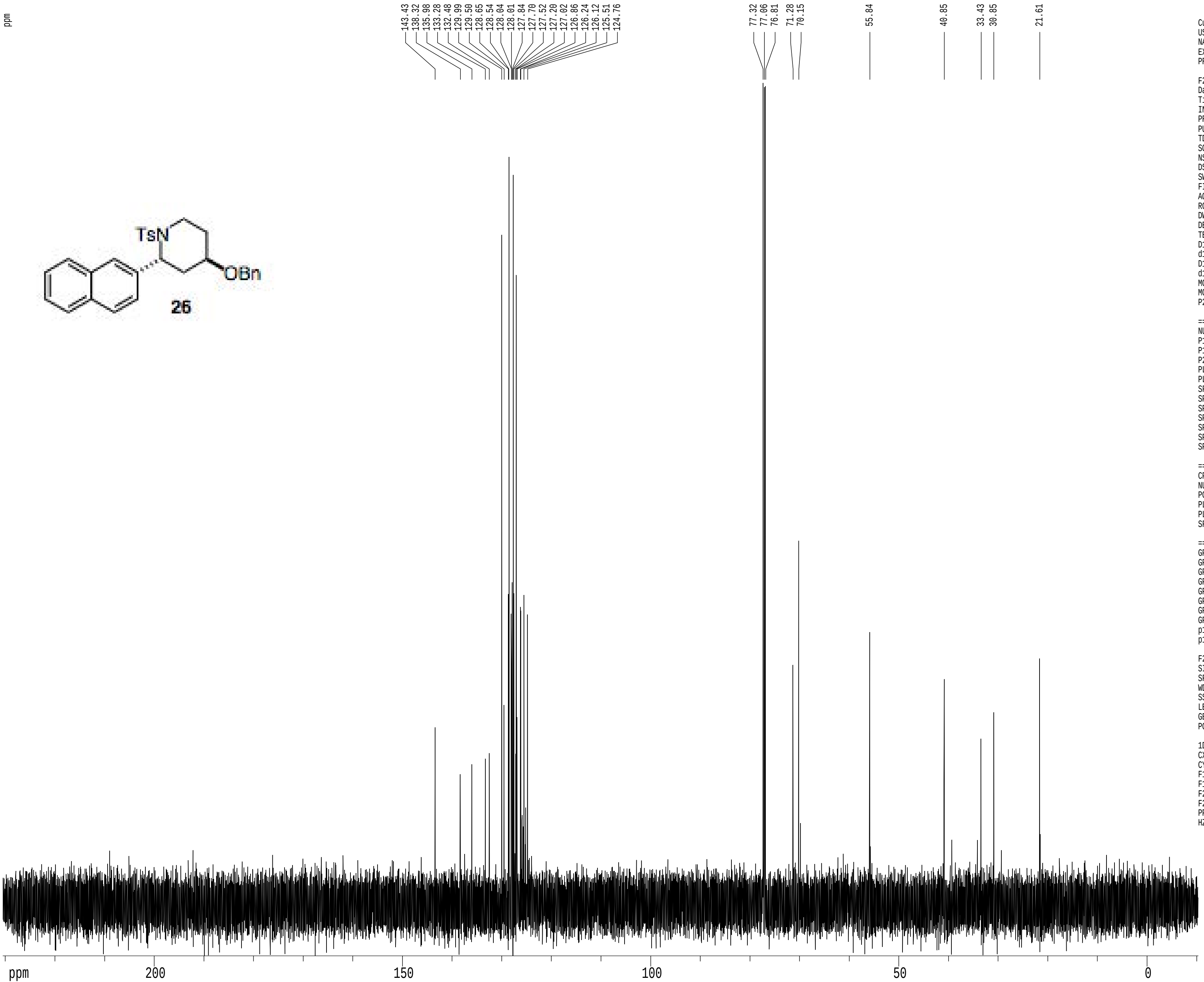

26

```
Current Data Parameters
USER          caherber
NAME      CAH-I-236-full
EXPNO                5
PROCNO               1

F2 - Acquisition Parameters
Date_         20210616
Time              8.47
INSTRUM        cryo500
PROBHD   5 mm CPTCI 1H-
PULPROG  SpinEchopg30gp2.prd
TD               65536
SOLVENT          CDCl3
NS                 128
DS                  16
SWH          30303.031 Hz
FIDRES        0.462388 Hz
AQ           1.0813940 sec
RG                3251
DW              16.500 usec
DE                6.00 usec
TE               298.0 K
D1          0.25000000 sec
d11         0.03000000 sec
D16         0.00020000 sec
d17         0.00019600 sec
MCREST      0.00000000 sec
MCWRK       0.01500000 sec
P2               37.70 usec

======== CHANNEL f1 ========
NUC1               13C
P1               18.85 usec
P12            2000.00 usec
P20             500.00 usec
PL0             120.00 dB
PL1              -1.00 dB
SFO1       125.7942548 MHz
SP2               1.55 dB
SP4               1.55 dB
SPNAM2     Crp60comp.4
SPNAM4   Crp60,0.5,20.1
SPOFF2            0.00 Hz
SPOFF4            0.00 Hz

======== CHANNEL f2 ========
CPDPRG2         waltz16
NUC2                 1H
PCPD2            100.00 usec
PL2                1.60 dB
PL12              22.00 dB
SFO2        500.2225011 MHz

====== GRADIENT CHANNEL =====
GPNAM1         SINE.100
GPNAM2         SINE.100
GPX1               0.00 %
GPX2               0.00 %
GPY1               0.00 %
GPY2               0.00 %
GPZ1              30.00 %
GPZ2              50.00 %
p15              500.00 usec
p16             1000.00 usec

F2 - Processing parameters
SI                65536
SF          125.7804190 MHz
WDW                  no
SSB                   0
LB                 0.00 Hz
GB                    0
PC                 2.00

1D NMR plot parameters
CX                22.80 cm
CY                15.65 cm
F1P             230.637 ppm
F1             29009.68 Hz
F2P             -10.287 ppm
F2             -1293.96 Hz
PPMCM          10.56688 ppm/cm
HZCM         1329.10706 Hz/cm
```

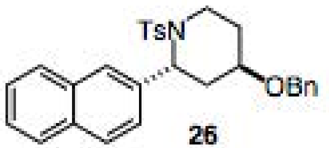

26

gcosy60

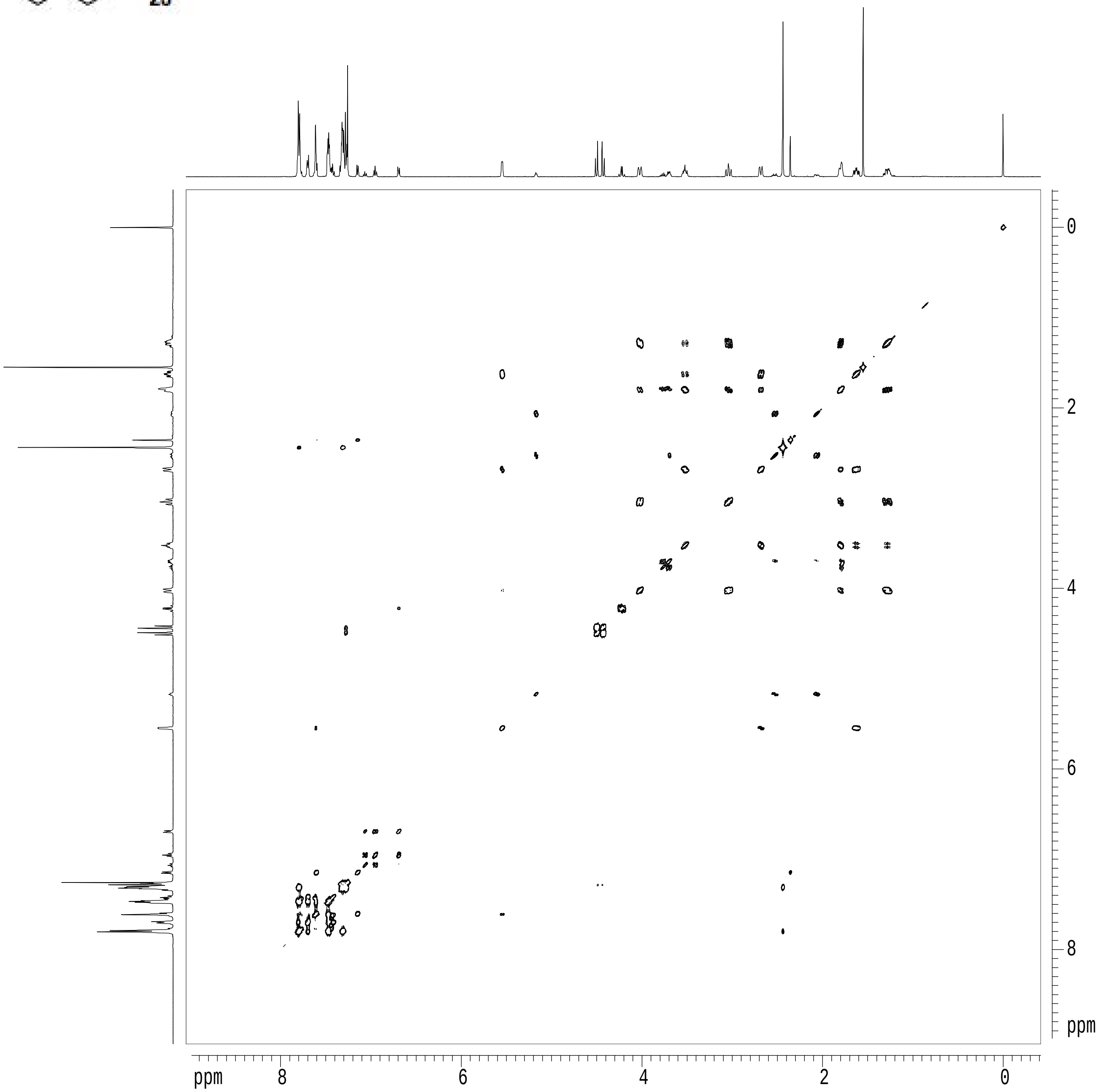

Current Data Parameters

USER	caherber
NAME	CAH-I-236-full
EXPNO	2
PROCNO	1

F2 - Acquisition Parameters

Date_	20210616
Time	8.03
INSTRUM	cryo500
PROBHD	5 mm CPTCI 1H-
PULPROG	cosygp60.prd
TD	2048
SOLVENT	CDCl3
NS	1
DS	16
SWH	4734.849 Hz
FIDRES	2.311938 Hz
AQ	0.2163188 sec
RG	322.5
DW	105.600 usec
DE	6.00 usec
TE	298.0 K
d0	0.00000300 sec
D1	1.00000000 sec
d13	0.00000300 sec
D16	0.00020000 sec
IN0	0.00021120 sec

======== CHANNEL f1 ========

NUC1	1H
P1	9.75 usec
PL1	1.60 dB
SFO1	500.2221588 MHz

====== GRADIENT CHANNEL =====

GPNAM1	SMSQ10.100
GPNAM2	SMSQ10.100
GPX1	0.00 %
GPX2	0.00 %
GPY1	0.00 %
GPY2	0.00 %
GPZ1	17.00 %
GPZ2	17.00 %
P16	1000.00 usec

F1 - Acquisition parameters

ND0	1
TD	512
SFO1	500.2222 MHz
FIDRES	9.247751 Hz
SW	9.465 ppm
FnMODE	QF

F2 - Processing parameters

SI	1024
SF	500.2200329 MHz
WDW	SINE
SSB	0
LB	0.00 Hz
GB	0
PC	1.00

F1 - Processing parameters

SI	1024
MC2	QF
SF	500.2200329 MHz
WDW	SINE
SSB	0
LB	0.00 Hz
GB	0

2D NMR plot parameters

CX2	15.00 cm
CX1	15.00 cm
F2PLO	9.048 ppm
F2LO	4526.21 Hz
F2PHI	-0.417 ppm
F2HI	-208.63 Hz
F1PLO	9.048 ppm
F1LO	4526.21 Hz
F1PHI	-0.417 ppm
F1HI	-208.63 Hz
F2PPMCM	0.63104 ppm/cm
F2HZCM	315.65659 Hz/cm
F1PPMCM	0.63104 ppm/cm
F1HZCM	315.65659 Hz/cm

gnoe

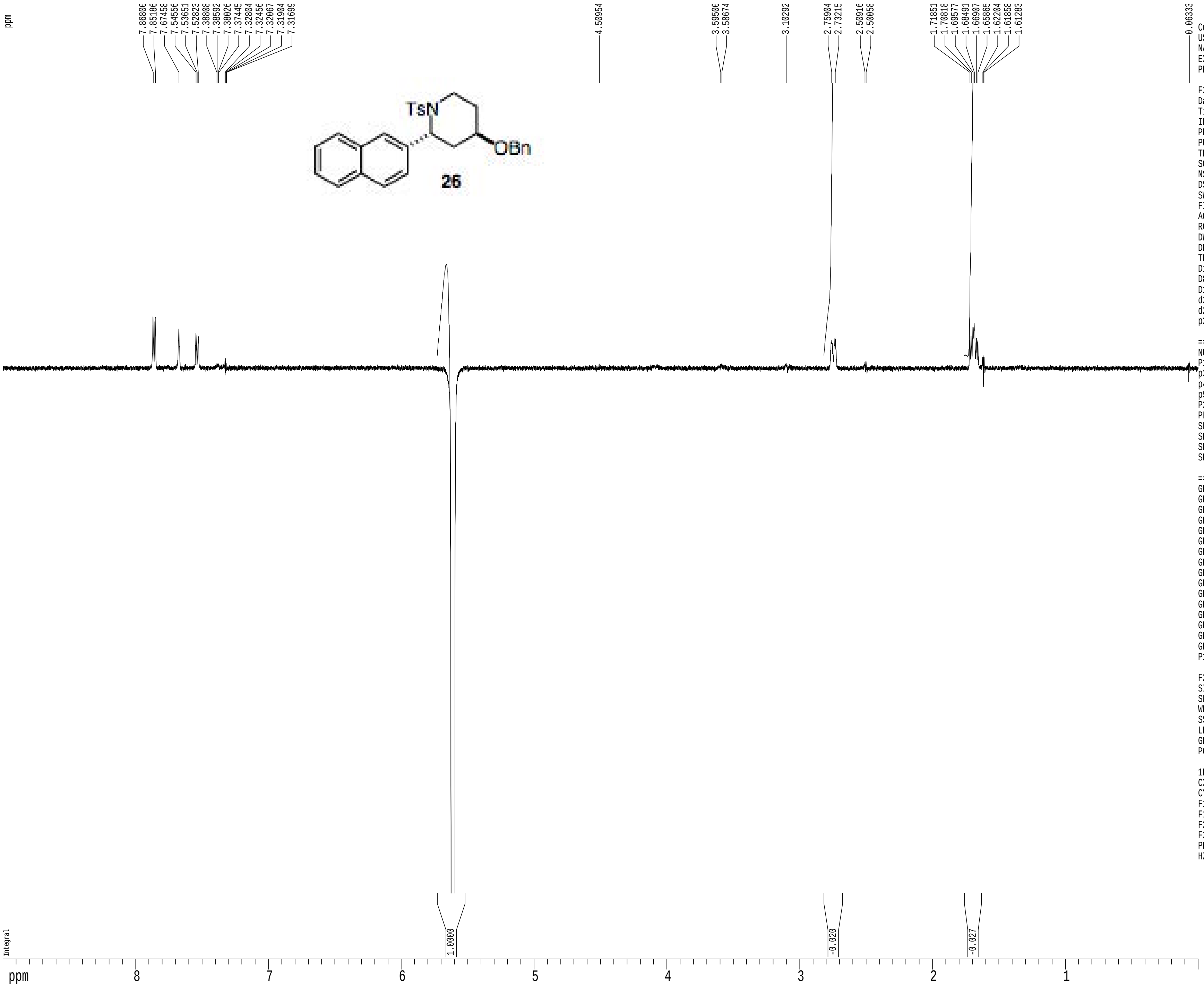

Current Data Parameters
USER caherber
NAME CAH-I-236-full
EXPNO 3
PROCNO 1

F2 - Acquisition Parameters
Date_ 20210616
Time 8.29
INSTRUM cryo500
PROBHD 5 mm CPTCI 1H-
PULPROG gnoe1cc22.prd
TD 65536
SOLVENT CDCl3
NS 128
DS 8
SWH 8012.820 Hz
FIDRES 0.122266 Hz
AQ 4.0894966 sec
RG 161.3
DW 62.400 usec
DE 6.00 usec
TE 298.0 K
D1 1.00000000 sec
D8 0.50000000 sec
D16 0.00020000 sec
d21 0.33375451 sec
d22 0.16399699 sec
p2 19.50 usec

======== CHANNEL f1 ========
NUC1 1H
P1 9.75 usec
p3 29.25 usec
p4 39.00 usec
p5 26.00 usec
P29 40000.00 usec
PL1 1.60 dB
SFO1 500.2228073 MHz
SP9 60.00 dB
SPNAM9 gauss1.512
SPOFF9 0.00 Hz

====== GRADIENT CHANNEL =====
GPNAM1 SMSQ10.100
GPNAM2 SMSQ10.100
GPNAM3 SMSQ10.100
GPNAM4 SMSQ10.100
GPX1 0.00 %
GPX2 0.00 %
GPX3 0.00 %
GPX4 0.00 %
GPY1 0.00 %
GPY2 0.00 %
GPY3 0.00 %
GPY4 0.00 %
GPZ1 7.00 %
GPZ2 3.00 %
GPZ3 2.30 %
GPZ4 -2.30 %
P16 1000.00 usec

F2 - Processing parameters
SI 65536
SF 500.2200000 MHz
WDW no
SSB 0
LB 0.00 Hz
GB 0
PC 1.00

1D NMR plot parameters
CX 22.80 cm
CY 50.00 cm
F1P 9.000 ppm
F1 4501.98 Hz
F2P 0.000 ppm
F2 0.00 Hz
PPMCM 0.39474 ppm/cm
HZCM 197.45528 Hz/cm

1H spectrum

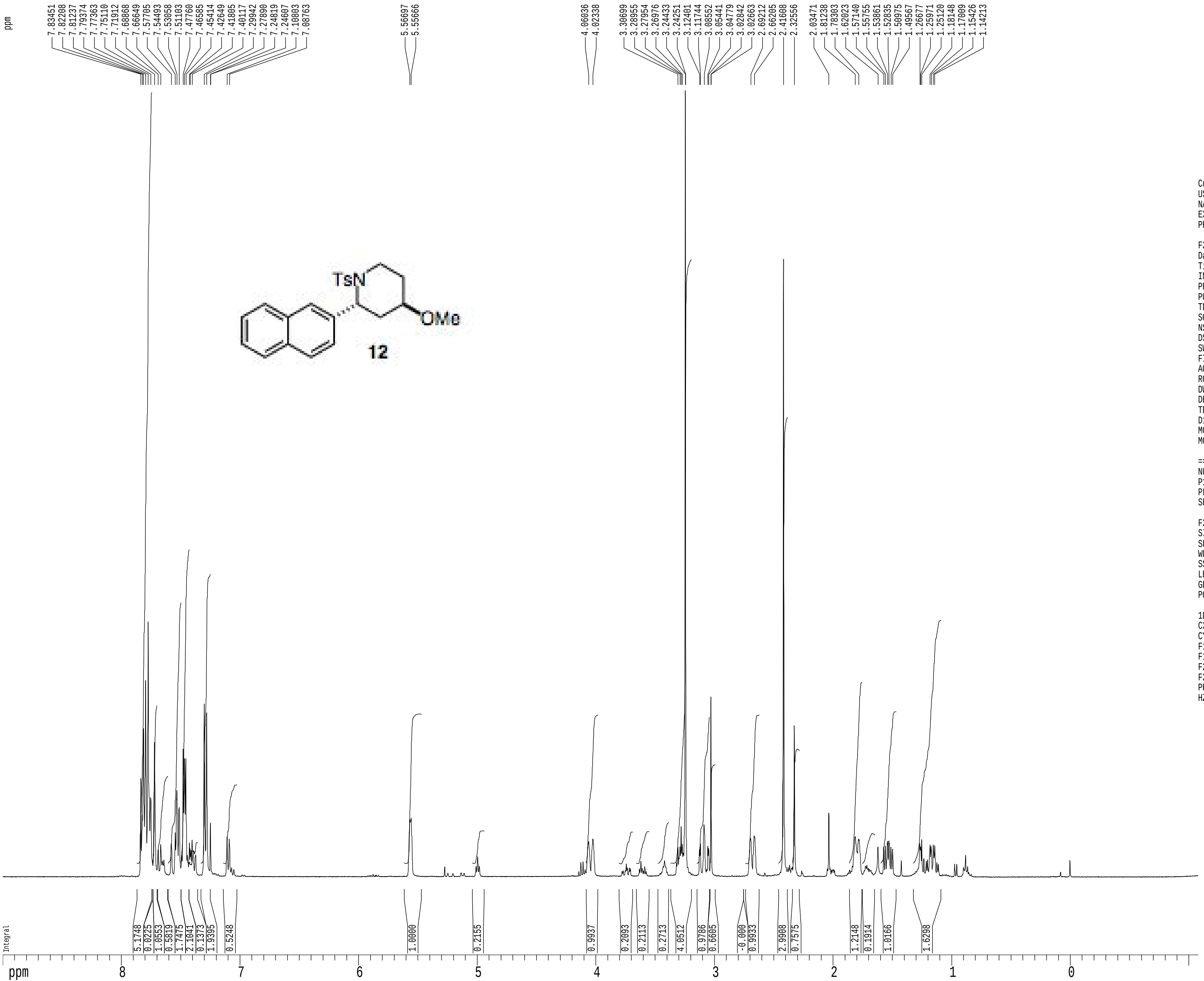

Current Data Parameters
USER caherber
NAME CAH-I-209
EXPNO 1
PROCNO 1

F2 - Acquisition Parameters
Date_ 20210415
Time 17.05
INSTRUM drx400
PROBHD 5 mm QNP H/F/P
PULPROG zg30
TD 65536
SOLVENT CDCl3
NS 8
DS 2
SWH 6410.256 Hz
FIDRES 0.097813 Hz
AQ 5.1118579 sec
RG 114
DW 78.000 usec
DE 4.50 usec
TE 298.0 K
D1 0.10000000 sec
MCREST 0.00000000 sec
MCWRK 0.01500000 sec

======== CHANNEL f1 ========
NUC1 1H
P1 12.00 usec
PL1 -1.60 dB
SFO1 400.1328009 MHz

F2 - Processing parameters
SI 65536
SF 400.1300267 MHz
WDW no
SSB 0
LB 0.00 Hz
GB 0
PC 2.00

1D NMR plot parameters
CX 22.80 cm
CY 15.00 cm
F1P 9.000 ppm
F1 3601.17 Hz
F2P -1.077 ppm
F2 -430.96 Hz
PPMCM 0.44198 ppm/cm
HZCM 176.84787 Hz/cm

Z-restored spin-echo 13C spectrum with 1H decoupling

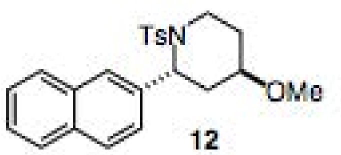

12

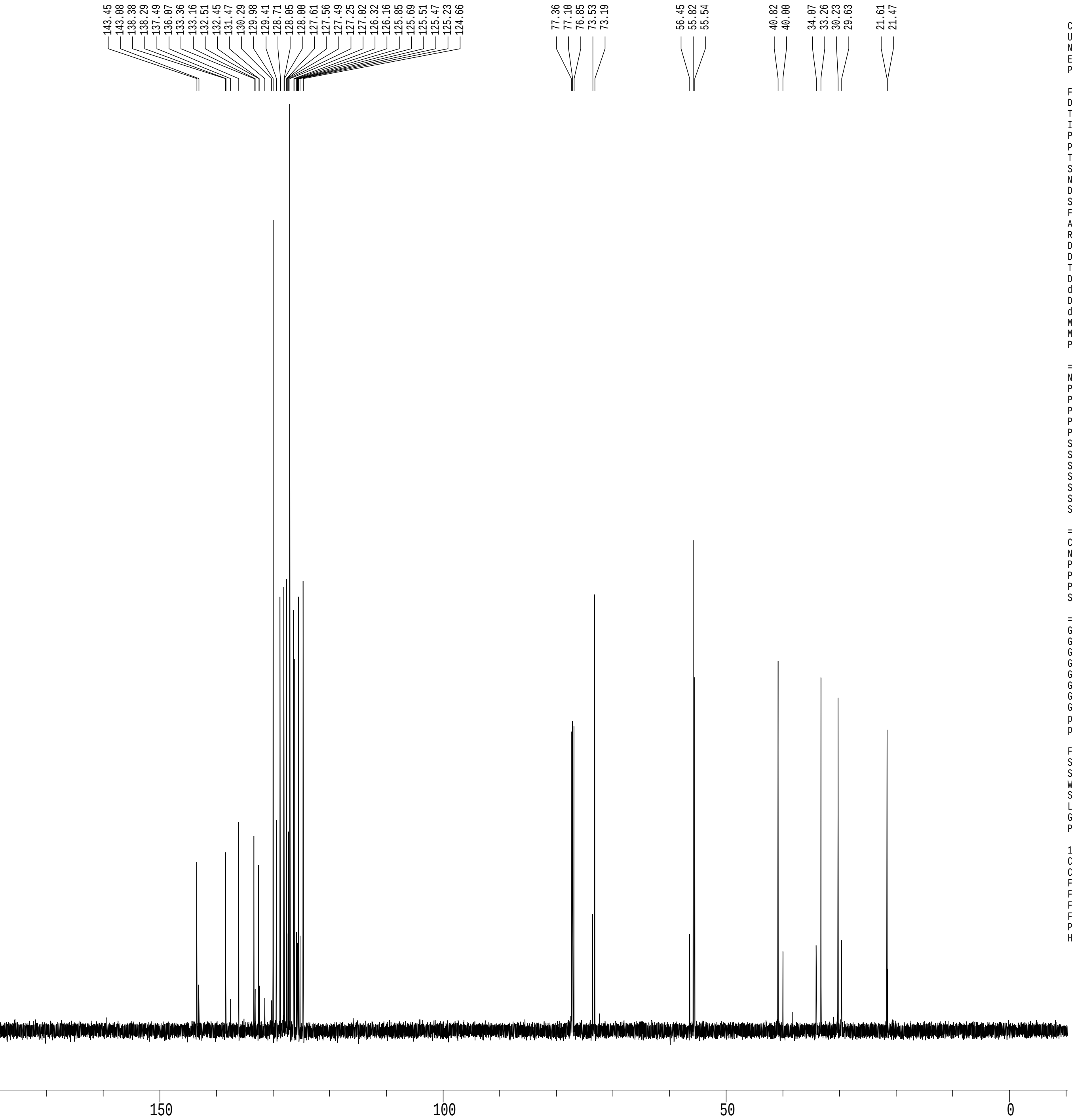

Current Data Parameters
USER caherber
NAME CAH-I-209-COSY
EXPNO 3
PROCNO 1

F2 - Acquisition Parameters
Date_ 20210417
Time 11.08
INSTRUM cryo500
PROBHD 5 mm CPTCI 1H-
PULPROG SpinEchopg30gp2.prd
TD 65536
SOLVENT CDCl3
NS 296
DS 16
SWH 30303.031 Hz
FIDRES 0.462388 Hz
AQ 1.0813940 sec
RG 2580.3
DW 16.500 usec
DE 6.00 usec
TE 298.0 K
D1 0.25000000 sec
d11 0.03000000 sec
D16 0.00020000 sec
d17 0.00019600 sec
MCREST 0.00000000 sec
MCWRK 0.01500000 sec
P2 37.70 usec

======== CHANNEL f1 ========
NUC1 13C
P1 18.85 usec
P12 2000.00 usec
P20 500.00 usec
PL0 120.00 dB
PL1 -1.00 dB
SFO1 125.7942548 MHz
SP2 1.55 dB
SP4 1.55 dB
SPNAM2 Crp60comp.4
SPNAM4 Crp60,0.5,20.1
SPOFF2 0.00 Hz
SPOFF4 0.00 Hz

======== CHANNEL f2 ========
CPDPRG2 waltz16
NUC2 1H
PCPD2 100.00 usec
PL2 1.60 dB
PL12 22.00 dB
SFO2 500.2225011 MHz

====== GRADIENT CHANNEL =====
GPNAM1 SINE.100
GPNAM2 SINE.100
GPX1 0.00 %
GPX2 0.00 %
GPY1 0.00 %
GPY2 0.00 %
GPZ1 30.00 %
GPZ2 50.00 %
p15 500.00 usec
p16 1000.00 usec

F2 - Processing parameters
SI 65536
SF 125.7804190 MHz
WDW no
SSB 0
LB 0.00 Hz
GB 0
PC 2.00

1D NMR plot parameters
CX 22.80 cm
CY 15.65 cm
F1P 230.637 ppm
F1 29009.68 Hz
F2P -10.287 ppm
F2 -1293.96 Hz
PPMCM 10.56688 ppm/cm
HZCM 1329.10706 Hz/cm

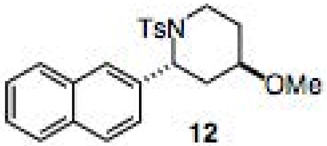

12

gcosy60

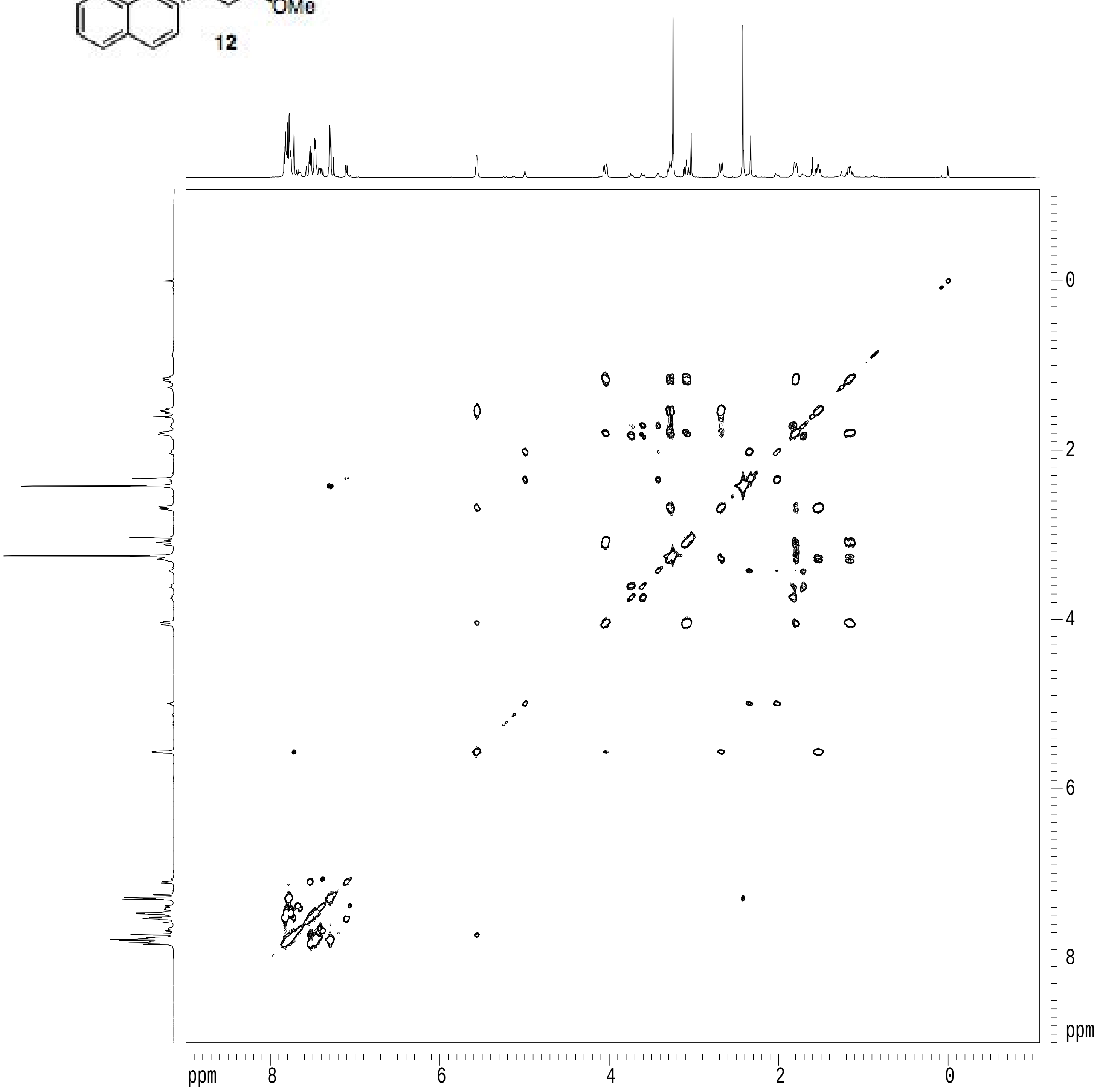

Current Data Parameters

USER	caherber
NAME	CAH-I-209-COSY
EXPNO	2
PROCNO	1

F2 - Acquisition Parameters

Date_	20210417
Time	10.51
INSTRUM	cryo500
PROBHD	5 mm CPTCI 1H-
PULPROG	cosygp60.prd
TD	2048
SOLVENT	CDCl3
NS	1
DS	16
SWH	8012.820 Hz
FIDRES	3.912510 Hz
AQ	0.1278452 sec
RG	35.9
DW	62.400 usec
DE	6.00 usec
TE	298.0 K
d0	0.00000300 sec
D1	1.00000000 sec
d13	0.00000300 sec
D16	0.00020000 sec
IN0	0.00012480 sec

======== CHANNEL f1 ========

NUC1	1H
P1	9.75 usec
PL1	1.60 dB
SFO1	500.2235015 MHz

====== GRADIENT CHANNEL =====

GPNAM1	SMSQ10.100
GPNAM2	SMSQ10.100
GPX1	0.00 %
GPX2	0.00 %
GPY1	0.00 %
GPY2	0.00 %
GPZ1	17.00 %
GPZ2	17.00 %
P16	1000.00 usec

F1 - Acquisition parameters

ND0	1
TD	512
SFO1	500.2235 MHz
FIDRES	15.650040 Hz
SW	16.018 ppm
FnMODE	QF

F2 - Processing parameters

SI	1024
SF	500.2200362 MHz
WDW	SINE
SSB	0
LB	0.00 Hz
GB	0
PC	1.00

F1 - Processing parameters

SI	1024
MC2	QF
SF	500.2200362 MHz
WDW	SINE
SSB	0
LB	0.00 Hz
GB	0

2D NMR plot parameters

CX2	15.00 cm
CX1	15.00 cm
F2PLO	9.000 ppm
F2LO	4501.98 Hz
F2PHI	-1.082 ppm
F2HI	-541.03 Hz
F1PLO	9.000 ppm
F1LO	4501.98 Hz
F1PHI	-1.082 ppm
F1HI	-541.03 Hz
F2PPMCM	0.67211 ppm/cm
F2HZCM	336.20062 Hz/cm
F1PPMCM	0.67211 ppm/cm
F1HZCM	336.20062 Hz/cm

gnoe

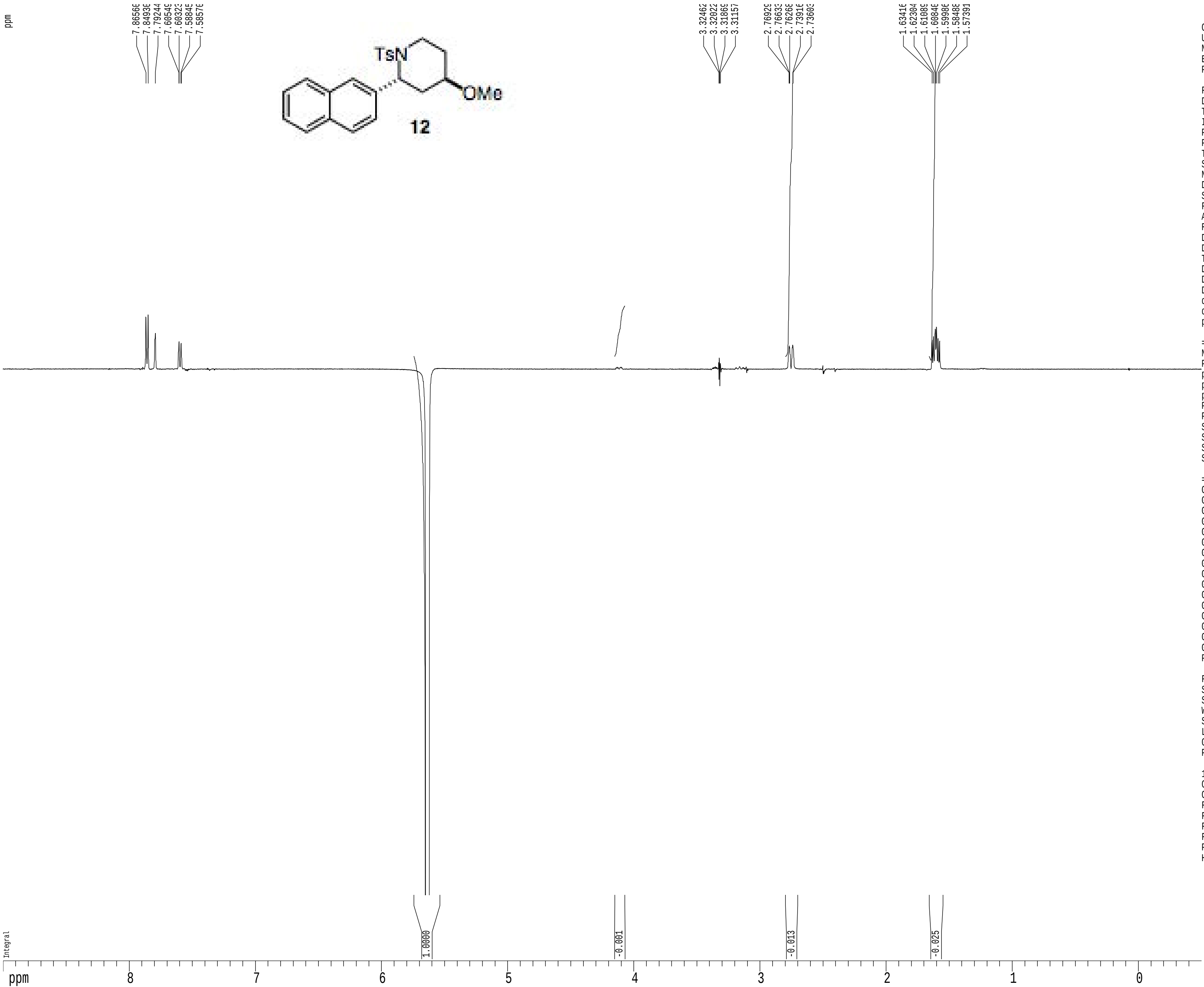

Current Data Parameters
USER caherber
NAME CAH-I-209-noe
EXPNO 2
PROCNO 1

F2 - Acquisition Parameters
Date_ 20210417
Time 14.16
INSTRUM cryo500
PROBHD 5 mm CPTCI 1H-
PULPROG gnoe1cc22.prd
TD 65536
SOLVENT CDCl3
NS 128
DS 8
SWH 8012.820 Hz
FIDRES 0.122266 Hz
AQ 4.0894966 sec
RG 80.6
DW 62.400 usec
DE 6.00 usec
TE 298.0 K
D1 1.00000000 sec
D8 0.50000000 sec
D16 0.00020000 sec
d21 0.33375451 sec
d22 0.16399699 sec
p2 19.50 usec

======== CHANNEL f1 ========
NUC1 1H
P1 9.75 usec
p3 29.25 usec
p4 39.00 usec
p5 26.00 usec
P29 40000.00 usec
PL1 1.60 dB
SFO1 500.2228198 MHz
SP9 60.00 dB
SPNAM9 gauss1.512
SPOFF9 0.00 Hz

====== GRADIENT CHANNEL =====
GPNAM1 SMSQ10.100
GPNAM2 SMSQ10.100
GPNAM3 SMSQ10.100
GPNAM4 SMSQ10.100
GPX1 0.00 %
GPX2 0.00 %
GPX3 0.00 %
GPX4 0.00 %
GPY1 0.00 %
GPY2 0.00 %
GPY3 0.00 %
GPY4 0.00 %
GPZ1 7.00 %
GPZ2 3.00 %
GPZ3 2.30 %
GPZ4 -2.30 %
P16 1000.00 usec

F2 - Processing parameters
SI 65536
SF 500.2200000 MHz
WDW no
SSB 0
LB 0.00 Hz
GB 0
PC 1.00

1D NMR plot parameters
CX 22.80 cm
CY 50.00 cm
F1P 9.000 ppm
F1 4501.98 Hz
F2P -0.500 ppm
F2 -250.11 Hz
PPMCM 0.41667 ppm/cm
HZCM 208.42500 Hz/cm

1H spectrum

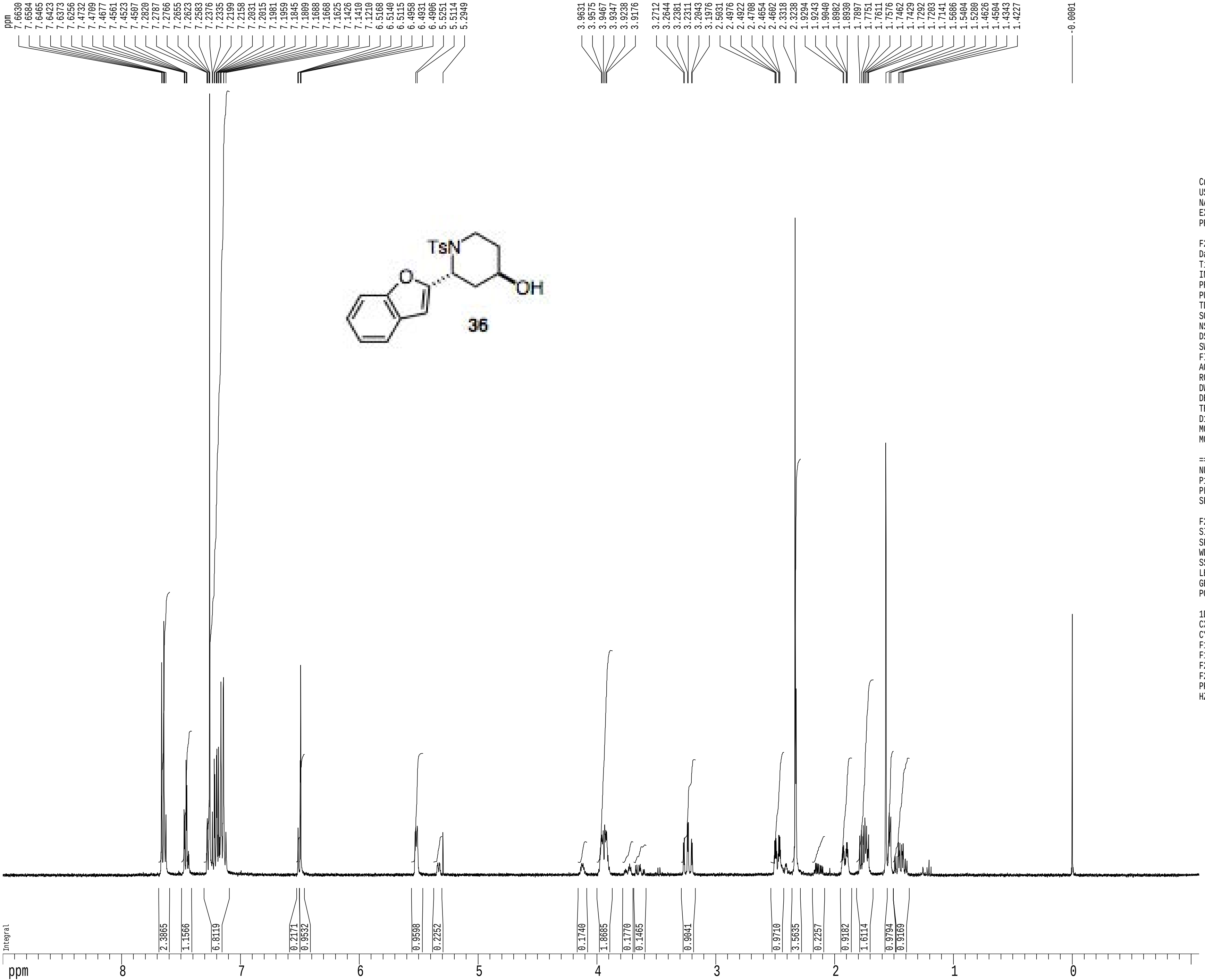

Current Data Parameters
USER caherber
NAME CAH-I-215
EXPNO 2
PROCNO 1

F2 - Acquisition Parameters
Date_ 20210503
Time 13.54
INSTRUM drx400
PROBHD 5 mm QNP H/F/P
PULPROG zg30
TD 65536
SOLVENT CDCl3
NS 8
DS 2
SWH 6410.256 Hz
FIDRES 0.097813 Hz
AQ 5.1118579 sec
RG 512
DW 78.000 usec
DE 4.50 usec
TE 298.0 K
D1 0.10000000 sec
MCREST 0.00000000 sec
MCWRK 0.01500000 sec

======== CHANNEL f1 ========
NUC1 1H
P1 12.00 usec
PL1 -1.60 dB
SFO1 400.1328009 MHz

F2 - Processing parameters
SI 65536
SF 400.1300223 MHz
WDW no
SSB 0
LB 0.00 Hz
GB 0
PC 2.00

1D NMR plot parameters
CX 22.80 cm
CY 15.00 cm
F1P 9.000 ppm
F1 3601.17 Hz
F2P -1.066 ppm
F2 -426.56 Hz
PPMCM 0.44149 ppm/cm
HZCM 176.65485 Hz/cm

1H spectrum

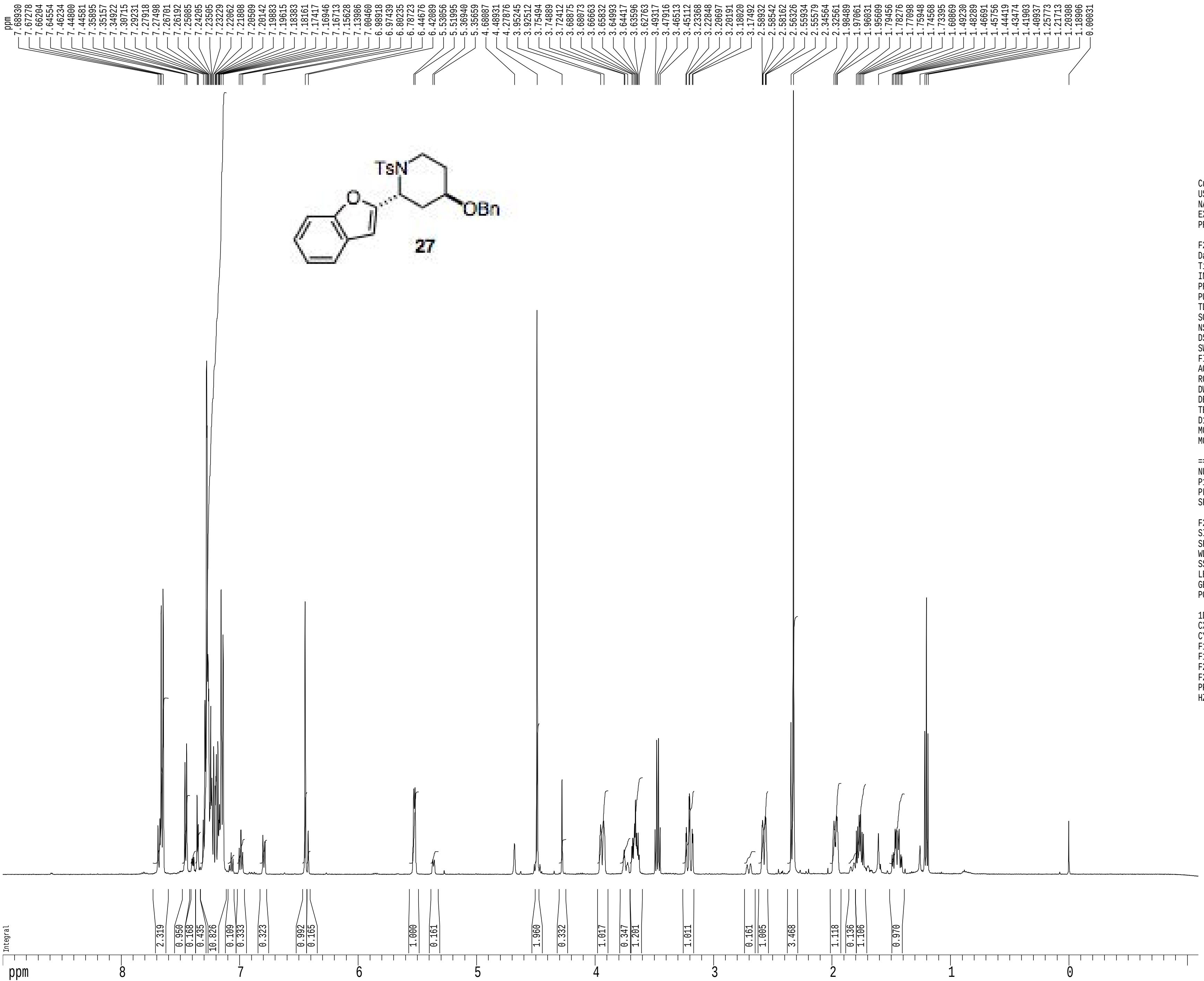

27

Current Data Parameters
USER caherber
NAME CAH-I-220-full
EXPNO 1
PROCNO 1

F2 - Acquisition Parameters
Date_ 20210506
Time 17.24
INSTRUM cryo500
PROBHD 5 mm CPTCI 1H-
PULPROG zg30
TD 48074
SOLVENT CDCl3
NS 8
DS 2
SWH 8012.820 Hz
FIDRES 0.166677 Hz
AQ 2.9998677 sec
RG 4
DW 62.400 usec
DE 6.00 usec
TE 298.0 K
D1 0.10000000 sec
MCREST 0.00000000 sec
MCWRK 0.01500000 sec

======== CHANNEL f1 ========
NUC1 1H
P1 9.75 usec
PL1 1.60 dB
SFO1 500.2235015 MHz

F2 - Processing parameters
SI 65536
SF 500.2200401 MHz
WDW no
SSB 0
LB 0.00 Hz
GB 0
PC 1.00

1D NMR plot parameters
CX 22.80 cm
CY 15.00 cm
F1P 9.000 ppm
F1 4501.98 Hz
F2P -1.089 ppm
F2 -544.94 Hz
PPMCM 0.44252 ppm/cm
HZCM 221.35622 Hz/cm

Z-restored spin-echo 13C spectrum with 1H decoupling

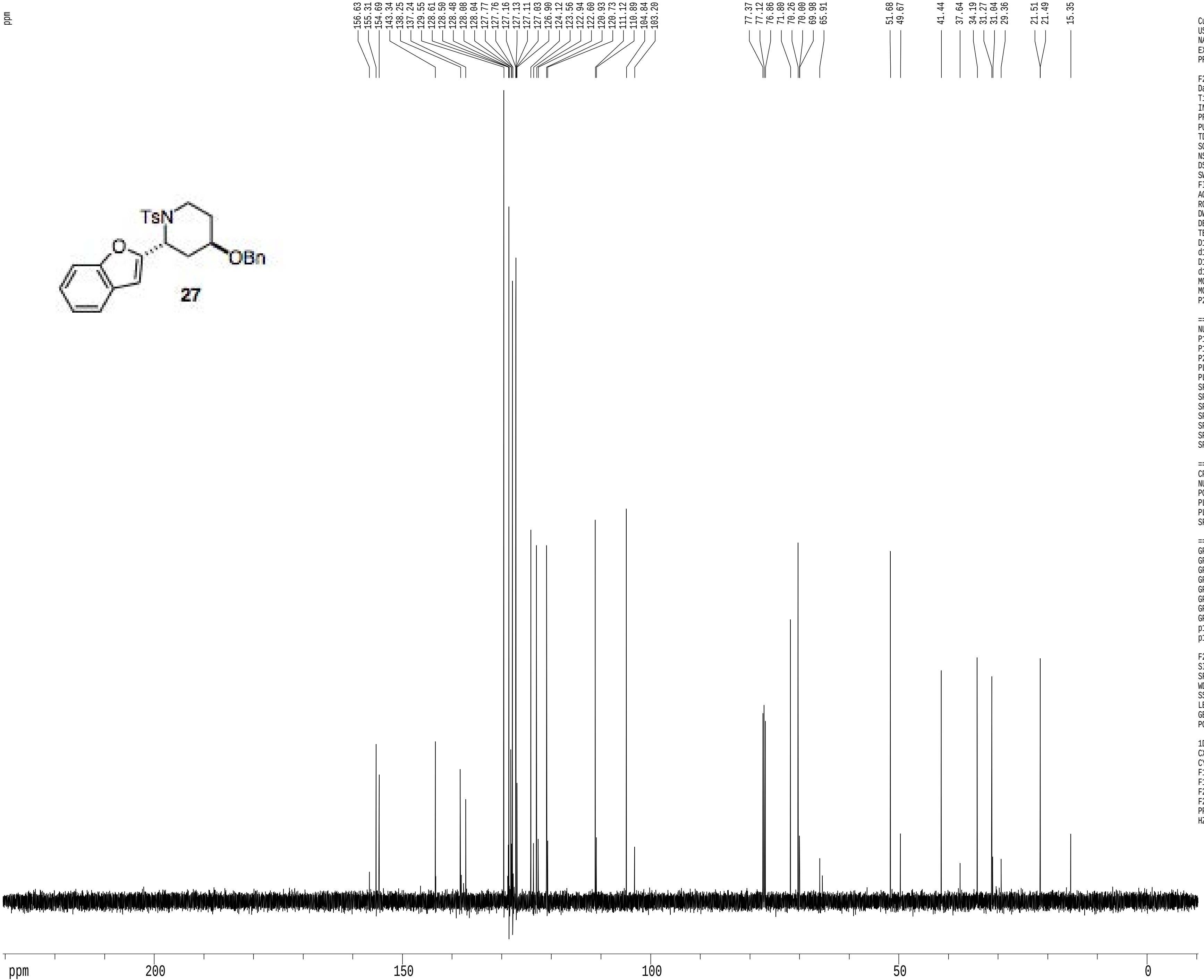


```
Current Data Parameters
USER          caherber
NAME     CAH-I-220-full
EXPNO                5
PROCNO               1

F2 - Acquisition Parameters
Date_         20210506
Time             18.09
INSTRUM        cryo500
PROBHD   5 mm CPTCI 1H-
PULPROG  SpinEchopg30gp2.prd
TD               65536
SOLVENT          CDCl3
NS                  96
DS                  16
SWH          30303.031 Hz
FIDRES        0.462388 Hz
AQ           1.0813940 sec
RG                4096
DW              16.500 usec
DE                6.00 usec
TE               298.0 K
D1          0.25000000 sec
d11         0.03000000 sec
D16         0.00020000 sec
d17         0.00019600 sec
MCREST      0.00000000 sec
MCWRK       0.01500000 sec
P2               37.70 usec

======== CHANNEL f1 ========
NUC1               13C
P1               18.85 usec
P12            2000.00 usec
P20             500.00 usec
PL0             120.00 dB
PL1              -1.00 dB
SFO1       125.7942548 MHz
SP2               1.55 dB
SP4               1.55 dB
SPNAM2     Crp60comp.4
SPNAM4   Crp60,0.5,20.1
SPOFF2            0.00 Hz
SPOFF4            0.00 Hz

======== CHANNEL f2 ========
CPDPRG2        waltz16
NUC2                1H
PCPD2           100.00 usec
PL2               1.60 dB
PL12             22.00 dB
SFO2       500.2225011 MHz

====== GRADIENT CHANNEL =====
GPNAM1        SINE.100
GPNAM2        SINE.100
GPX1              0.00 %
GPX2              0.00 %
GPY1              0.00 %
GPY2              0.00 %
GPZ1             30.00 %
GPZ2             50.00 %
p15             500.00 usec
p16            1000.00 usec

F2 - Processing parameters
SI               65536
SF         125.7804190 MHz
WDW                 no
SSB                  0
LB                0.00 Hz
GB                   0
PC                2.00

1D NMR plot parameters
CX               22.80 cm
CY               15.65 cm
F1P            230.637 ppm
F1            29009.68 Hz
F2P            -10.287 ppm
F2            -1293.96 Hz
PPMCM         10.56688 ppm/cm
HZCM        1329.10706 Hz/cm
```

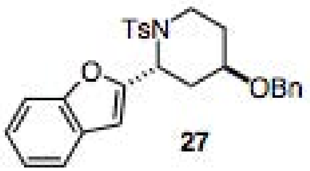

27

gcosy60

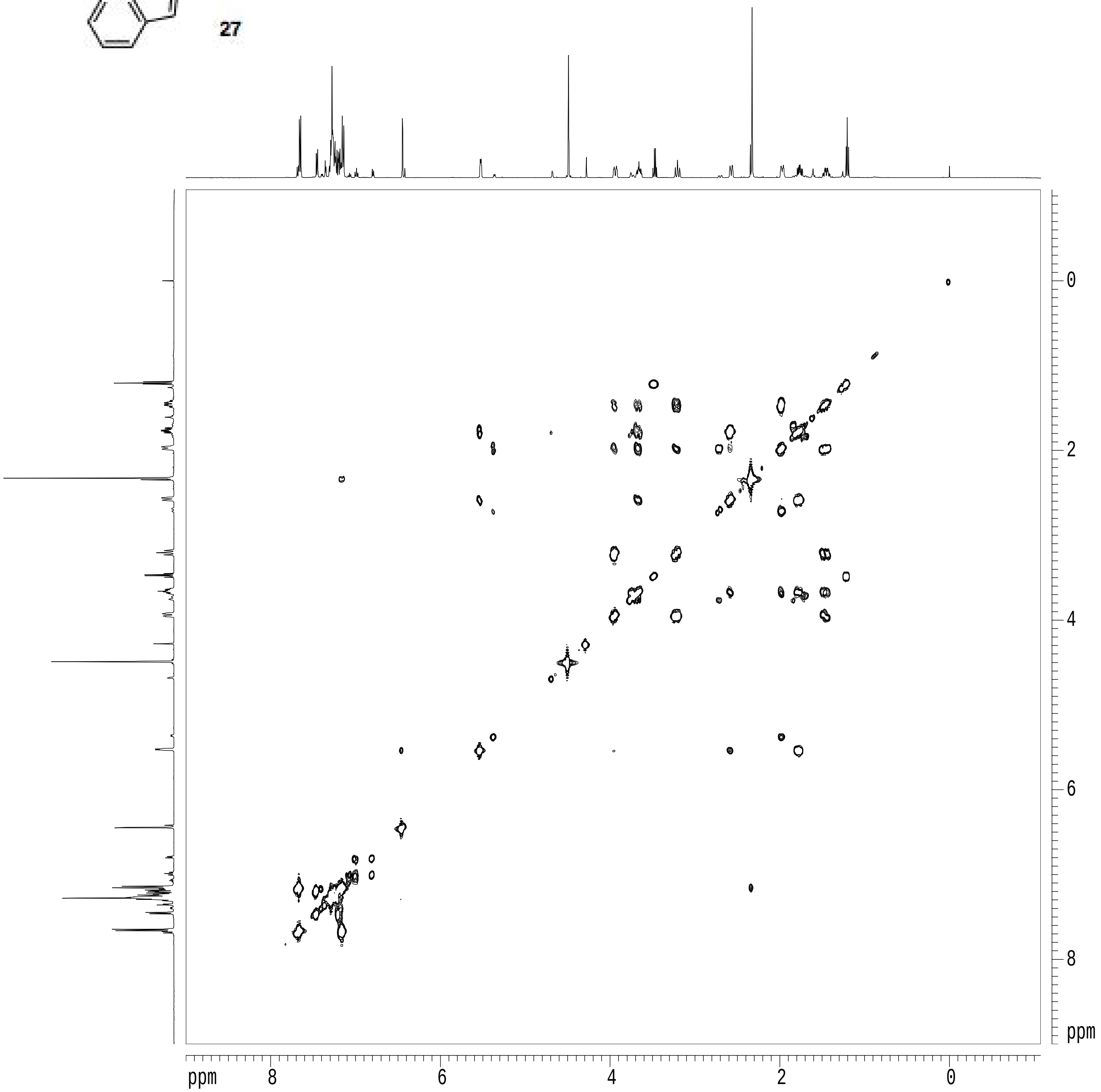

Current Data Parameters

USER	caherber
NAME	CAH-I-220-full
EXPNO	2
PROCNO	1

F2 - Acquisition Parameters

Date_	20210506
Time	17.27
INSTRUM	cryo500
PROBHD	5 mm CPTCI 1H-
PULPROG	cosygp60.prd
TD	2048
SOLVENT	CDCl3
NS	2
DS	16
SWH	8012.820 Hz
FIDRES	3.912510 Hz
AQ	0.1278452 sec
RG	35.9
DW	62.400 usec
DE	6.00 usec
TE	298.0 K
d0	0.00000300 sec
D1	1.00000000 sec
d13	0.00000300 sec
D16	0.00020000 sec
IN0	0.00012480 sec

======== CHANNEL f1 ========

NUC1	1H
P1	9.75 usec
PL1	1.60 dB
SFO1	500.2235015 MHz

====== GRADIENT CHANNEL =====

GPNAM1	SMSQ10.100
GPNAM2	SMSQ10.100
GPX1	0.00 %
GPX2	0.00 %
GPY1	0.00 %
GPY2	0.00 %
GPZ1	17.00 %
GPZ2	17.00 %
P16	1000.00 usec

F1 - Acquisition parameters

ND0	1
TD	269
SFO1	500.2235 MHz
FIDRES	29.787437 Hz
SW	16.018 ppm
FnMODE	QF

F2 - Processing parameters

SI	1024
SF	500.2200329 MHz
WDW	SINE
SSB	0
LB	0.00 Hz
GB	0
PC	1.00

F1 - Processing parameters

SI	1024
MC2	QF
SF	500.2200329 MHz
WDW	SINE
SSB	0
LB	0.00 Hz
GB	0

2D NMR plot parameters

CX2	15.00 cm
CX1	15.00 cm
F2PLO	9.000 ppm
F2LO	4501.98 Hz
F2PHI	-1.075 ppm
F2HI	-537.73 Hz
F1PLO	9.000 ppm
F1LO	4501.98 Hz
F1PHI	-1.075 ppm
F1HI	-537.73 Hz
F2PPMCM	0.67167 ppm/cm
F2HZCM	335.98087 Hz/cm
F1PPMCM	0.67167 ppm/cm
F1HZCM	335.98087 Hz/cm

gnoe

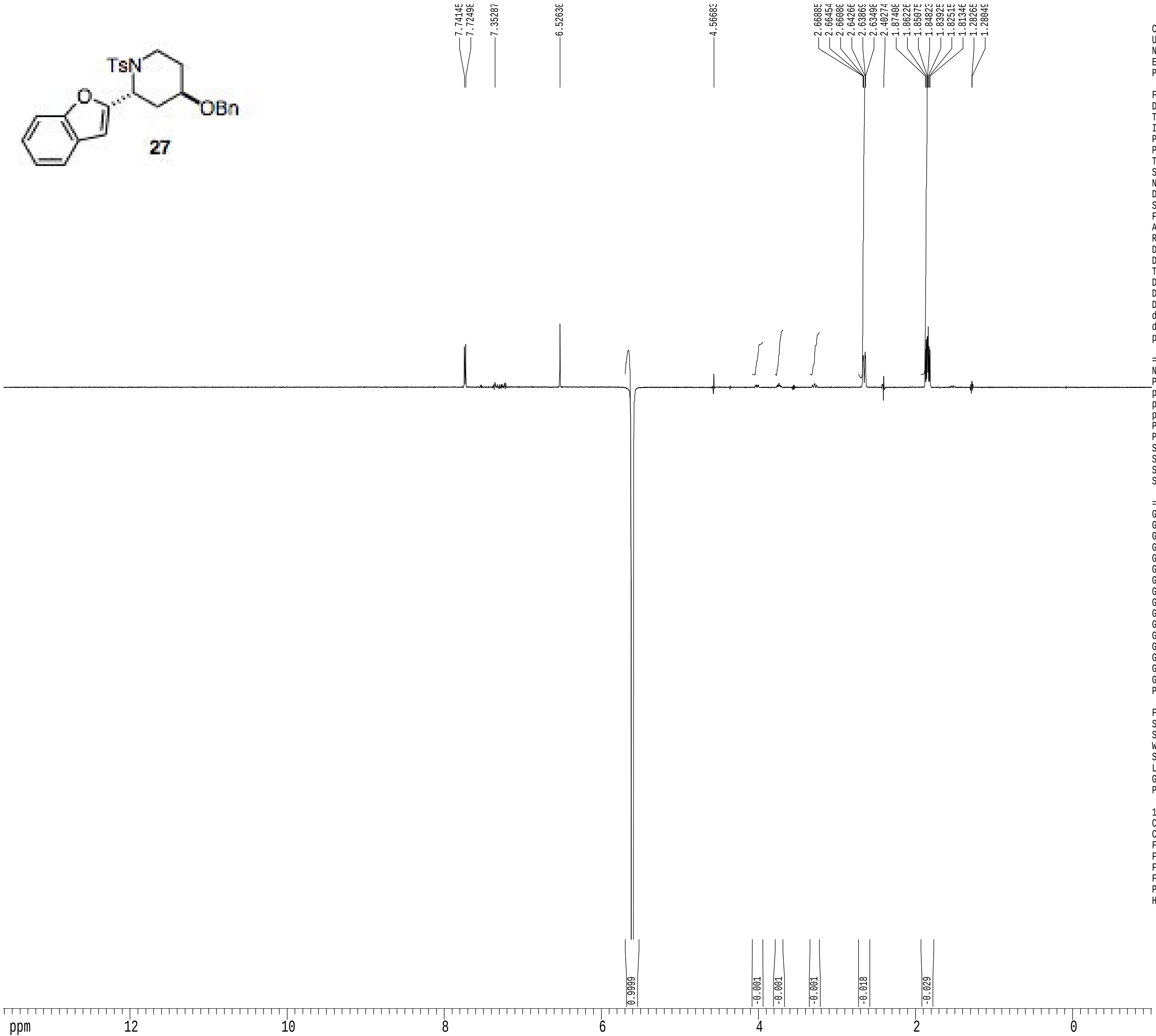


```
Current Data Parameters
USER            caherber
NAME     CAH-I-220-full
EXPNO                  3
PROCNO                 1

F2 - Acquisition Parameters
Date_           20210506
Time               17.52
INSTRUM          cryo500
PROBHD   5 mm CPTCI 1H-
PULPROG   gnoe1cc22.prd
TD                 65536
SOLVENT            CDCl3
NS                   128
DS                     8
SWH             8012.820 Hz
FIDRES          0.122266 Hz
AQ             4.0894966 sec
RG                  90.5
DW                62.400 usec
DE                  6.00 usec
TE                 298.0 K
D1            1.00000000 sec
D8            0.50000000 sec
D16           0.00020000 sec
d21           0.33375451 sec
d22           0.16399699 sec
p2                 19.50 usec

======== CHANNEL f1 ========
NUC1                  1H
P1                  9.75 usec
p3                 29.25 usec
p4                 39.00 usec
p5                 26.00 usec
P29             40000.00 usec
PL1                 1.60 dB
SFO1         500.2228033 MHz
SP9                60.00 dB
SPNAM9        gauss1.512
SPOFF9              0.00 Hz

====== GRADIENT CHANNEL =====
GPNAM1        SMSQ10.100
GPNAM2        SMSQ10.100
GPNAM3        SMSQ10.100
GPNAM4        SMSQ10.100
GPX1                0.00 %
GPX2                0.00 %
GPX3                0.00 %
GPX4                0.00 %
GPY1                0.00 %
GPY2                0.00 %
GPY3                0.00 %
GPY4                0.00 %
GPZ1                7.00 %
GPZ2                3.00 %
GPZ3                2.30 %
GPZ4               -2.30 %
P16              1000.00 usec

F2 - Processing parameters
SI                 65536
SF           500.2200000 MHz
WDW                   no
SSB                    0
LB                  0.00 Hz
GB                     0
PC                  1.00

1D NMR plot parameters
CX                 22.80 cm
CY                 50.00 cm
F1P               15.009 ppm
F1               7507.95 Hz
F2P               -1.009 ppm
F2               -504.87 Hz
PPMCM            0.70257 ppm/cm
HZCM           351.43951 Hz/cm
```
